# Coramitug, a Humanized Monoclonal Antibody for the Treatment of Transthyretin Amyloid Cardiomyopathy: A Phase 2, Randomized, Multicenter, Double-Blind, Placebo-Controlled Trial

**DOI:** 10.1161/CIRCULATIONAHA.125.077304

**Published:** 2025-11-10

**Authors:** Marianna Fontana, Pablo García-Pavía, Martha Grogan, Sanjiv J. Shah, Mads D.M. Engelmann, G. Kees Hovingh, Arnt V. Kristen, Michelle Z. Lim-Watson, Brian Malling, Soumitra Kar, Manjunatha Revanna, Nitasha Sarswat, Kenichi Tsujita, Kevin M. Alexander, Mathew S. Maurer

**Affiliations:** National Amyloidosis Centre, University College London (Royal Free Campus), United Kingdom (M.F.).; Department of Cardiology, Hospital Universitario Puerta de Hierro de Majadahonda, Health Research Institute of the Puerta de Hierro Majadahonda-Segovia de Arana, Centro de Investigación Biomédica en Red Enfermedades Cardiovasculares (CIBER-CV),and Centro Nacional de Investigaciones Cardiovasculares, Madrid, Spain (P.G.-P.).; Department of Cardiology, Mayo Clinic, Rochester, MN (M.G.).; Division of Cardiology, Department of Medicine, Feinberg School of Medicine, Northwestern University, Chicago, IL (S.J.S.).; Novo Nordisk A/S, Søborg, Denmark (M.D.M.E, G.K.H., M.R.).; Department of Cardiology, Angiology, and Pneumology, Medical University of Heidelberg, Germany (A.V.K.).; Novo Nordisk US Research & Development, Lexington, MA (M.L.-W., B.M.).; Novo Nordisk Service Centre Pvt Ltd, Bangalore, India (S.K.).; Biological Sciences Division, University of Chicago Medicine, IL (N.S.).; Department of Cardiovascular Medicine, Graduate School of Medical Sciences, Kumamoto University, Japan (K.T.).; Stanford Amyloid Center, Division of Cardiovascular Medicine, Stanford University School of Medicine, CA (K.M.A.).; Division of Cardiology, Columbia University Medical Center, New York Presbyterian Hospital, New York, NY (M.S.M.).

**Keywords:** amyloidogenic proteins, amyloidosis, cardiomyopathies, clinical study, heart failure, myocardium, walk test

## Abstract

**BACKGROUND:**

Transthyretin amyloidosis with cardiomyopathy is a progressive disease caused by the deposition of transthyretin (TTR) as amyloid in the myocardium. Current therapies may slow disease progression but do not clear existing deposits. Coramitug is a humanized monoclonal antibody that targets misfolded transthyretin, designed to promote clearance of transthyretin amyloid through antibody-mediated phagocytosis.

**METHODS:**

This phase 2, double-blind, placebo-controlled trial randomized participants with transthyretin amyloidosis with cardiomyopathy to receive infusions every 4 weeks of either coramitug at 2 dosages (10 mg/kg or 60 mg/kg) or placebo in a 1:1:1 ratio for 52 weeks. The primary end points were the change from baseline to week 52 in the 6-minute walk test and NT-proBNP (N-terminal pro-B-type natriuretic peptide) levels. Safety was assessed for up to 64 weeks by assessing treatment-emergent adverse events, all-cause mortality, and number of cardiovascular events (comprising hospitalization caused by cardiovascular events or urgent heart failure visits).

**RESULTS:**

In total, 104 participants (median age, 77 years; 93% men; 84% New York Heart Association class II; 13% with variant transthyretin amyloidosis with cardiomyopathy) were randomized and dosed: 34 to 10 mg/kg of coramitug, 35 to 60 mg/kg of coramitug, and 35 to placebo. Median NT-proBNP was 1985 pg/mL (interquartile range, 1224, 3406). In total, 90% of participants were receiving disease-modifying therapy; 84% were treated with tafamidis and 7 (6.7%) with transthyretin silencers (patisiran, n=4; vutrisiran, n=3). From baseline to week 52, 60 mg/kg of coramitug significantly reduced NT-proBNP levels compared with placebo (–48% [95% CI, –65% to –22%]; *P*=0.0017). The change in 6-minute walk test from baseline to week 52 was not statistically different from placebo with either dose. Coramitug (60 mg/kg) was associated with improved functional echocardiographic parameters and was well tolerated.

**CONCLUSIONS:**

This phase 2 trial showed that coramitug, an antibody targeting misfolded transthyretin in transthyretin amyloidosis with cardiomyopathy, was well tolerated and, at a dose of 60 mg/kg, resulted in a statistically significant reduction in NT-proBNP, a validated marker of disease progression, with no statistically significant effect on 6-minute walk test within 52 weeks.

**REGISTRATION:**

URL: https://www.clinicaltrials.gov; Unique identifier: NCT05442047.

**Clinical Perspective**

**What Is New?**

- Coramitug, a humanized monoclonal antibody targeting misfolded transthyretin, was evaluated in a phase 2, randomized, double-blind, placebo-controlled trial in patients with transthyretin amyloid with cardiomyopathy.
- At 60 mg/kg, coramitug significantly reduced NT-proBNP (N-terminal pro-B-type natriuretic peptide), a validated biomarker of disease progression, compared with placebo.
- Coramitug was well tolerated and associated with improvements in multiple echocardiographic parameters, although no statistically significant change in 6-minute walk test was observed within 52 weeks.

**What Are the Clinical Implications?**

- Coramitug offers an innovative therapeutic approach aiming to clear existing amyloid deposits, addressing a key unmet need in transthyretin amyloid with cardiomyopathy, in which currently approved therapies only reduce further deposition but do not reverse the disease.
- NT-proBNP reduction and echocardiographic improvements suggest potential for cardiac remodeling, which, together with the favorable safety profile, warrants further investigation of coramitug in clinical trials.

**Editorial, see p 226**

Transthyretin amyloidosis (ATTR) is a progressive and fatal disease caused by misfolded TTR (transthyretin) protein that accumulates as amyloid fibrils in multiple organs, commonly leading to cardiomyopathy and polyneuropathy.^1^ ATTR with cardiomyopathy (ATTR-CM) occurs with ageing in the absence of predisposing *TTR* variants (wild-type ATTR amyloidosis) or as a result of inherited *TTR* gene variants (variant ATTR [ATTRv] amyloidosis), often with a mixed phenotype that also includes polyneuropathy.^1^ Approved therapies for ATTR-CM reduce new amyloid production either by suppressing hepatic synthesis of the TTR protein through gene-silencing agents or by stabilizing the native tetrameric protein structure of TTR to prevent dissociation and misfolding.^2–4^ Although mechanistically distinct, both approaches aim to slow disease progression by limiting further amyloid deposition.^2–4^ However, despite these advances, no approved therapy to date actively removes existing amyloid deposits from the myocardium, and reversal of cardiac dysfunction remains an unmet therapeutic goal.

Two potential amyloid-depleting antibody therapies have been evaluated in phase 1 to date. In 2023, a phase 1 trial demonstrated that NI006, a recombinant human anti-ATTR IgG1 (immunoglobulin G1) monoclonal antibody, was well tolerated, reduced the extent of cardiac amyloid accumulation, and improved cardiac biomarkers.^5^ Coramitug, a humanized monoclonal antibody targeting a specific TTR epitope found on misfolded and aggregated forms of TTR, was shown in a further phase 1 trial to be well tolerated in participants with ATTRv amyloidosis.^6^ Although coramitug and NI006 are both humanized IgG1 monoclonal antibodies, they target distinct epitopes.

This randomized, double-blind trial in participants with ATTR-CM evaluated the effects of 2 dose levels of coramitug on functional end points, biomarkers, pharmacokinetics, and safety.

## Methods

### Data Sharing

Individual participant data will be shared in data sets in a de-identified format. Data will be available after research completion, approval of the product, and product use in the European Union and the United States. Access request proposals can be found at https://www.novonordisk-trials.com. Data will be shared with bona fide researchers submitting a research proposal approved by the independent review board. Individual participant data will be shared in data sets in a de-identified/anonymized format using a specialized SAS (SAS Institute Inc) data platform. The protocol and statistical analysis plan are provided in the Supplemental Material.

### Study Design

This was an international, phase 2, randomized, multicenter, double-blind trial that included a 52-week placebo-controlled treatment period and a 12-week safety follow-up period. Participants with ATTR-CM were recruited at 30 research sites across 10 countries (Canada, Czech Republic, France, Germany, Italy, Japan, Netherlands, Portugal, Spain, and the United States).

### Role of the Funding Source and Oversight

A steering committee designed the trial in collaboration with the sponsor, Novo Nordisk. An institutional review board or independent ethics committee at each trial center approved the protocol. An independent data and safety monitoring committee reviewed unblinded safety data after the first dose of coramitug or placebo was administered. An independent external events adjudication committee performed blinded adjudication of selected adverse events and deaths. The trial was conducted in accordance with the principles of the Declaration of Helsinki, the International Council for Harmonisation Guideline for Good Clinical Practice, and applicable laws and regulations.^7,8^ The sponsor selected the participating trial centers, oversaw the conduct and monitoring of the trial, received and maintained the trial database, and performed all the data analyses. Cardiac magnetic resonance imaging and echocardiographic images were transferred to a core laboratory (Clario, PA) for analysis. An independent analysis of the primary end points was performed by ICON. The initial draft of the article was written by the first and last authors. The following authors had direct access to the data and vouch for the accuracy and completeness of the data and analyses: S.J.S., B.M., and S.K.

### Patient Population

Eligible participants (Table S1) had confirmed ATTR-CM (either variant or wild-type disease) with an end-diastolic interventricular septal wall thickness of ≥12 mm and New York Heart Association class II or III symptoms with an NT-proBNP (N-terminal pro-B-type natriuretic peptide) ≥650 pg/mL in participants with sinus rhythm and >1000 pg/mL in participants with atrial fibrillation. Eligible participants needed to be able to walk ≥150 m and ≤450 m on a 6-minute walk test (6MWT) and have an estimated glomerular filtration rate ≥25 mL/min per 1.73 m^2^. A full list of inclusion and exclusion criteria is provided in the Supplemental Material. Participants were receiving stable background therapy, including general heart failure medication and disease-modifying treatment (stabilizers and silencers) for ATTR-CM, for at least 6 weeks before randomization. Sex (female/male) was either self- or investigator-reported; race/ethnicity was self-reported. All participants provided written informed consent.

### Randomization and Masking

Participants were centrally screened and randomly assigned to treatment using the randomization and trial supplies management system/interactive web response system. Each participant was assigned a unique 6-digit identification number, ensuring tracking without reassignments. The double-blind design kept participants, care providers, investigators, and outcome assessors unaware of treatment allocations. Unblinded staff managed trial product logistics, including shipment and dispensing, to preserve study integrity. Quality assurance auditors were allowed access to unblinded records for verification.

### Procedures

Eligible participants were stratified by disease type (ATTRv and wild-type ATTR) and were randomized 1:1:1 to 1 of 3 groups: coramitug at a dose of 10 mg/kg body weight, coramitug at a dose of 60 mg/kg body weight, or placebo. Coramitug was supplied as lyophilized powder and reconstituted with sterile water for injection or diluted with normal saline for infusion. Coramitug was prepared by unblinded staff, and a blinding cover was added to the intravenous bag before administration by appointed blinded staff. Coramitug or placebo was administered intravenously every 4 weeks from baseline to week 48. Premedication included antihistamines (25 mg of diphenhydramine or an equivalent dose of an H1 antihistamine) and paracetamol/acetaminophen (650–1000 mg). A sentinel cohort of 9 participants was closely monitored for cardiac safety during the initial 28 days of the study. During screening, participants in the sentinel cohort had Holter electrocardiogram monitoring for at least 48 hours within 2 weeks before randomization, with the cardiac monitoring report reviewed before the participants were randomized. After receiving the first dose of the trial medication, participants were continuously monitored in the hospital for at least 24 hours to detect any immediate adverse cardiac events. After discharge, participants continued to be monitored on an outpatient basis for up to 7 days after the start of infusion using Holter ECG. Safety evaluations were conducted by a medical monitoring group after monitoring the initial participants for 7 days, with additional oversight from the data and safety monitoring committee before further dosing occurred. Efficacy was assessed at baseline and every 4 weeks thereafter for 52 weeks; safety was assessed at screening to week 64. Adverse events were recorded from the time of randomization until the end of the study. Participants who discontinued coramitug or placebo or withdrew consent were asked to return for an end-of-trial visit. Participants who completed the placebo-controlled treatment period and attended the last follow-up visit were invited to enroll in an ongoing 144-week open-label extension study.

### Pharmacokinetic and Immunogenicity Assessments

Samples for antidrug antibody analysis were collected at visits 2 (randomization), 3, 4, 8, 12, 15, and 16 (end of study). Plasma coramitug levels were measured using a validated assay at every visit from randomization.

### End Points

The primary end points were changes from baseline to week 52 in NT-proBNP levels and 6MWT distance with coramitug compared with placebo. Secondary efficacy end points included the change from baseline to week 52 in global longitudinal strain from echocardiography, high-sensitivity troponin I plasma levels, the Kansas City Cardiomyopathy Questionnaire Clinical Summary Score, extracellular volume by cardiac magnetic resonance, and Neuropathy Impairment Score^9^ (only for participants with ATTRv cardiomyopathy). Secondary safety end points were treatment-emergent adverse events, time to occurrence of all-cause mortality, and number of cardiovascular events, including hospitalizations for cardiovascular reasons or urgent heart failure visits, from baseline to week 64. Echocardiography and cardiac magnetic resonance images were centrally read by an imaging core laboratory (Clario, PA), which was blinded to treatment allocation. Standardized echocardiography was performed 5 times before infusion from randomization to end of treatment (week 52). Echocardiography measurements were made across 3 cardiac cycles (or 5 if ectopy or atrial fibrillation was present) and averaged. Cardiac magnetic resonance imaging was performed at the same time points as echocardiography. Measurement of hematocrit for the calculation of extracellular volume was obtained immediately before the magnetic resonance imaging scan, if possible; otherwise, it was obtained within ±3 days of scanning. Detailed imaging methods are provided in the Supplemental Material.

### Statistical Analysis

The sample size calculation was based on the precision of the comparisons of the primary end points. All efficacy and safety analyses were performed in the intention-to-treat population. The primary end points were assessed using an ANCOVA model, with treatment and stratification variable as fixed factors and baseline response as a covariate. Missing data because of death or cardiovascular hospitalizations or urgent heart failure visits were imputed with least possible value (0 m) for 6MWT and highest observed value for NT-proBNP across all visits and all participants. Intermittent missing postbaseline values were imputed in each treatment group using Markov Chain Monte Carlo methods to generate multiple (×1000) copies of monotone missing data. A stepwise procedure sequentially imputed the missing values for the remaining visits according to arm and permanent treatment discontinuation, depending on whether the participant was on the randomized study intervention or had prematurely discontinued. Each complete data set was analyzed separately, and estimates were combined using the Rubin rules. For NT-proBNP values, the analyses were performed after log-transforming the responses and then back-transforming them to the original scale. Nominal *P* values with 95% CIs are presented for all end points. Data were analyzed using SAS version 9.4 and R version 4.3.1.

The trial was registered at https://www.clinicaltrials.gov (Unique identifier: NCT05442047) on June 28, 2022.

## Results

**Figure 1.**
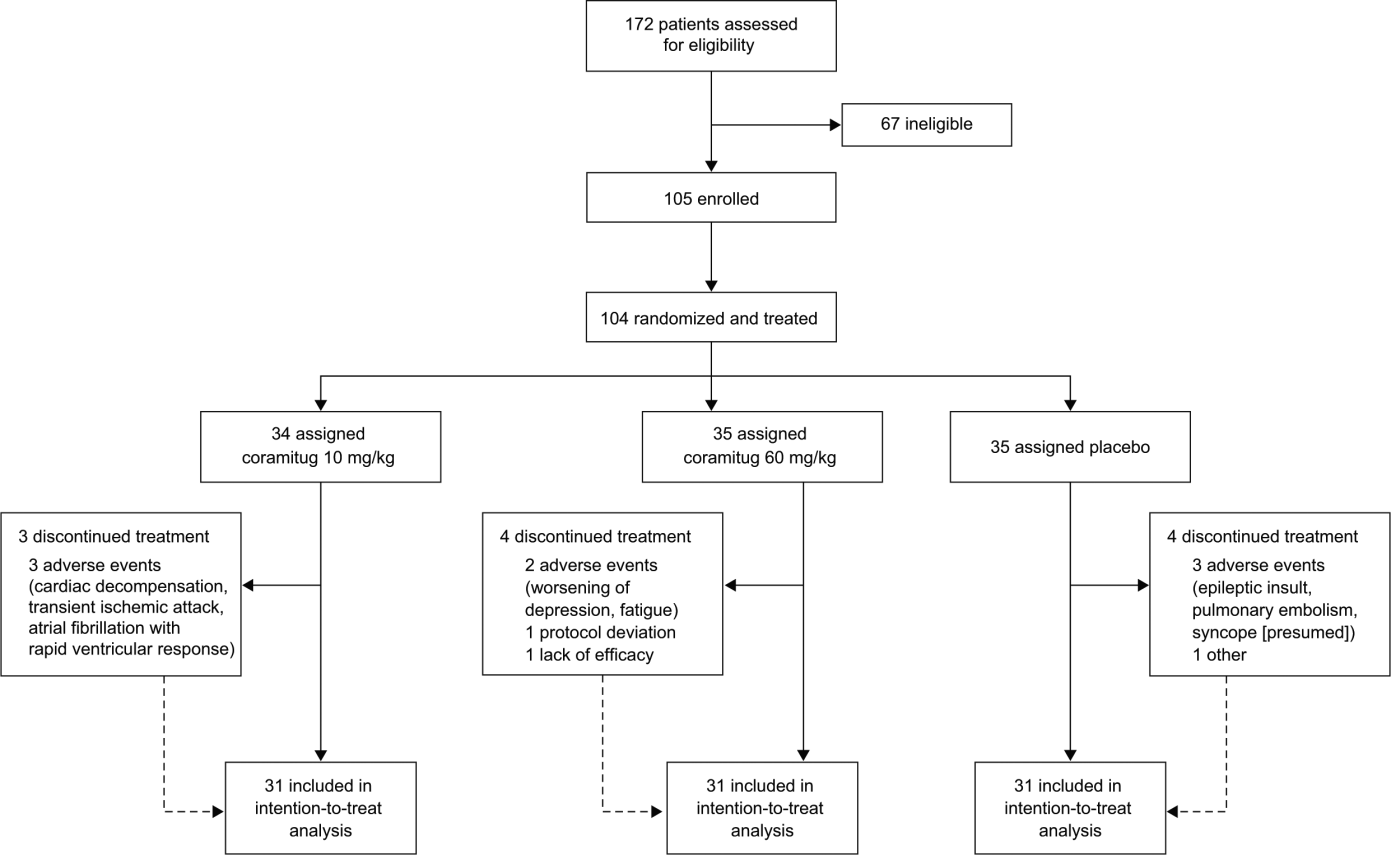
CONSORT diagram.

Between August 2, 2022, and February 6, 2024, 172 participants were screened for eligibility, of whom 105 (61%) were randomized. One participant was randomized in error, so 104 participants were part of the intention-to-treat population (Figure 1): 35 received placebo, 34 received 10 mg/kg of coramitug, and 35 received 60 mg/kg of coramitug (Table 1). The median age was 77.0 years (interquartile range, 73.0, 81.0), and 97 (93.3%) were men. Ninety-one (87.5%) had wild-type ATTR-CM, and 13 (12.5%) had ATTRv-CM; 87 participants (83.7%) were categorized as New York Heart Association class II, and 17 (16.3%) were categorized as New York Heart Association class III. Eighty-seven participants (83.7%) were treated with tafamidis and 7 (6.7%) with TTR silencers (patisiran, n=4; vutrisiran, n=3). Baseline characteristics were similar between cohorts, apart from NT-proBNP levels, which were numerically higher in the placebo group than in the coramitug groups. Adherence to the study protocol was high, with 31 participants in each cohort (88% to 91% across cohorts) on treatment at week 52.

**Table 1.**
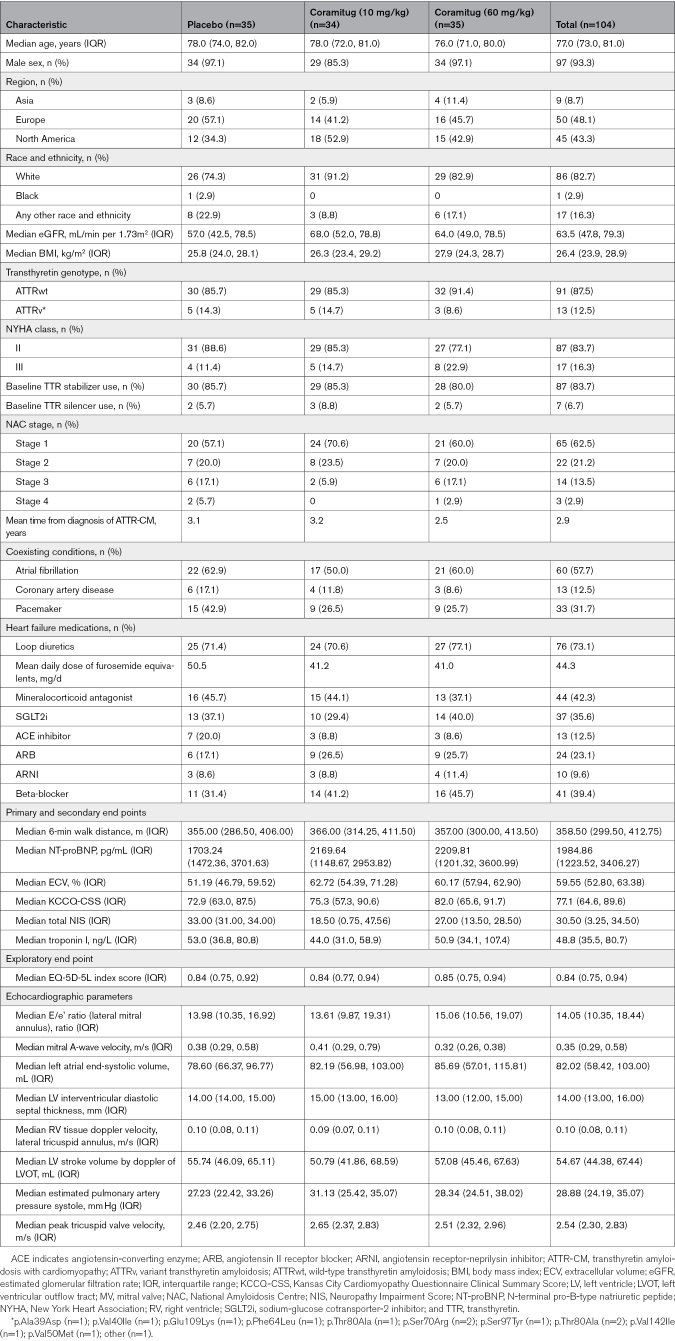
Demographic and Baseline Clinical Characteristics

**Figure 2.**
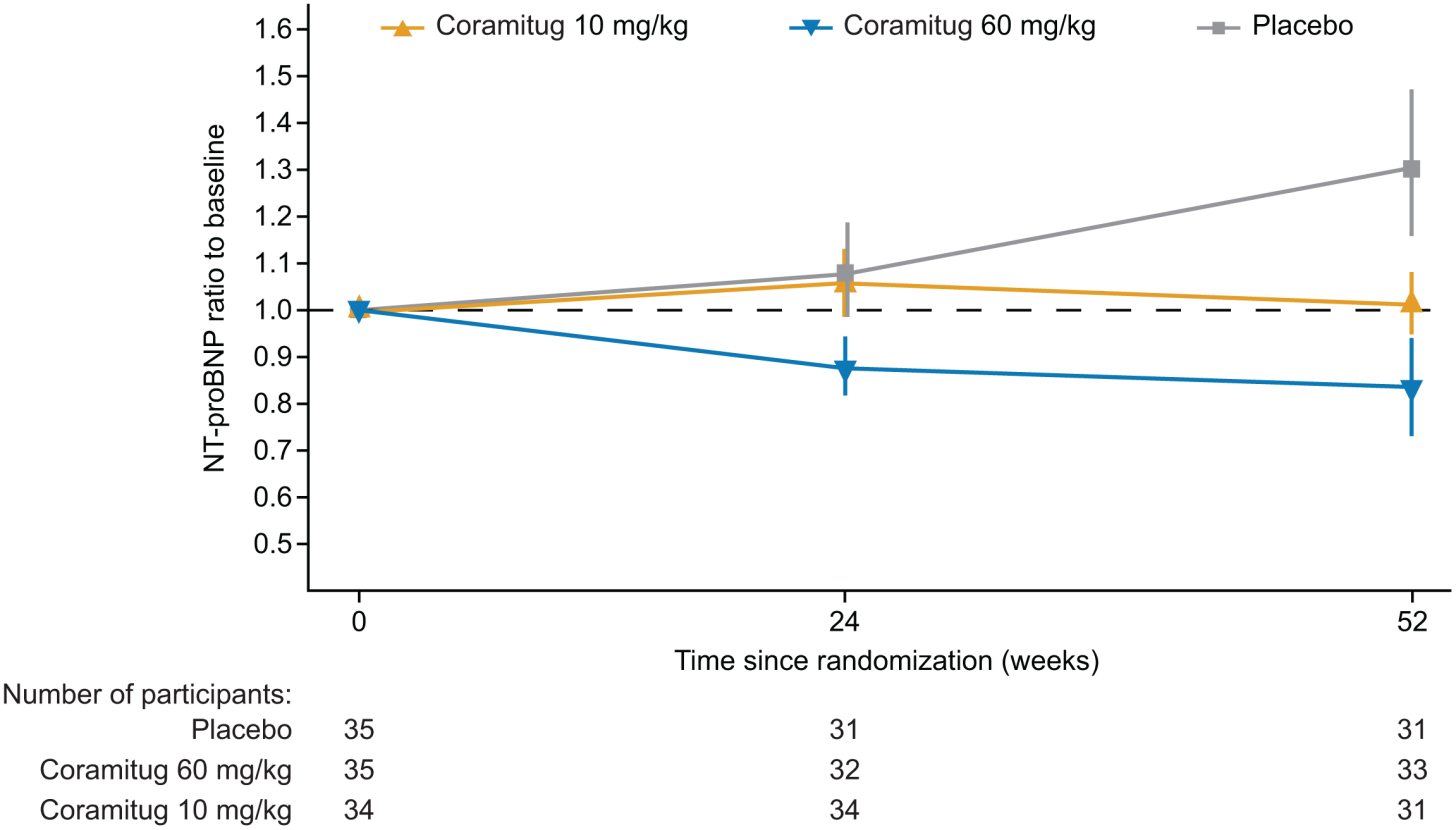
**NT-proBNP ratio to baseline over time.** Data shown are observed geometric mean±SEM on log-scale back-transformed. NT-proBNP indicates N-terminal pro-B-type natriuretic peptide.

Compared with the cohort assigned to placebo, participants receiving coramitug at a dose of 60 mg/kg demonstrated a statistically significant reduction in the ratio to baseline of NT-proBNP at week 52, with a treatment ratio of 0.52 (95% CI, 0.35 to 0.78; *P*=0.0017; Table 2; Figure 2); for observed data, see Table S2, and for data on NT-pro-BNP analyzed using a mixed model for repeated measurements, see Table S3. Participants receiving coramitug at a dose of 10 mg/kg exhibited a ratio to baseline at week 52 compared with placebo that resulted in a treatment ratio of 0.72 (95% CI, 0.49 to 1.07; *P*=0.1043). The treatment differences in 6MWT between coramitug at 60 mg/kg and placebo showed an estimated treatment difference of 13.45 m (95% CI, −29.56 to 56.46), indicating no statistically significant difference. Similarly, the treatment difference between coramitug at 10 mg/kg and placebo resulted in a nonsignificant estimated treatment difference of −0.31 m (95% CI, −43.25 to 42.64).

**Table 2.**
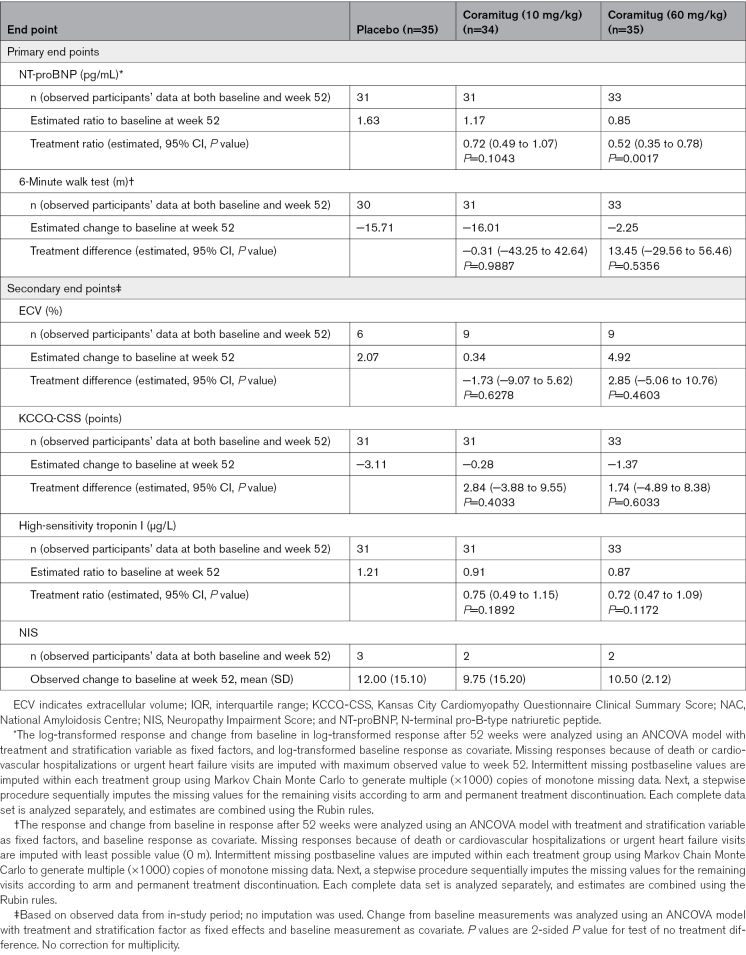
Primary and Secondary Efficacy End Points

No statistically significant effect was observed for the secondary end points from baseline to 52 weeks (Table 2). Myocardial extracellular volume as measured by cardiac magnetic resonance was obtained at baseline and after 52 weeks in 6 participants receiving placebo, in 9 participants receiving 10 mg/kg of coramitug, and in 9 participants receiving 60 mg/kg of coramitug, and did not show any differences between the cohorts. The changes in the results on high-sensitivity troponin I, Neuropathy Impairment Score for those with ATTRv-CM, and the Kansas City Cardiomyopathy Questionnaire Clinical Summary Score at 52 weeks are shown in Table 2.

Coramitug was associated with no apparent drug-related serious adverse events. During this placebo-controlled randomized trial, >90% of participants had an adverse event (Table 3); dyspnea and cardiac failure were reported in >15% of participants. There were 6 infusion-related reactions in the 60 mg/kg of coramitug group, 2 in those receiving 10 mg/kg of coramitug, and 4 in participants receiving placebo. There were no infusion-related adverse events reported on the day of infusion. Hypersensitivity reactions were mild to moderate in severity and did not cause any dose changes or interruptions in study drug administration. No cases of thrombocytopenia were reported. There were numerically fewer treatment-emergent adverse events in those receiving 10 mg/kg of coramitug (257 events) and 60 mg/kg of coramitug (216 events) than in those receiving placebo (311 events). Four deaths occurred in the study; 2 participants were receiving 10 mg/kg of coramitug, and 2 were receiving placebo. Narratives are provided in the Supplemental Material. Deaths were considered to be unrelated to the trial product. The most frequently observed adverse events were fatigue, dyspnea, cough, heart failure, and arrhythmia, which are expected in this patient population (Table S4). The frequency and type of treatment-emergent adverse events appeared to be similar across cohorts. Safety laboratory assessments (biochemistry, hematology, coagulation parameters, lipids, hormones, and urinalysis) did not reveal any clinically significant differences. Safety-related cardiac monitoring ECGs and echocardiography did not show any apparent evidence of treatment-emergent new cardiac dysfunction, pericardial effusion, or an increase in arrhythmias.

**Table 3.**
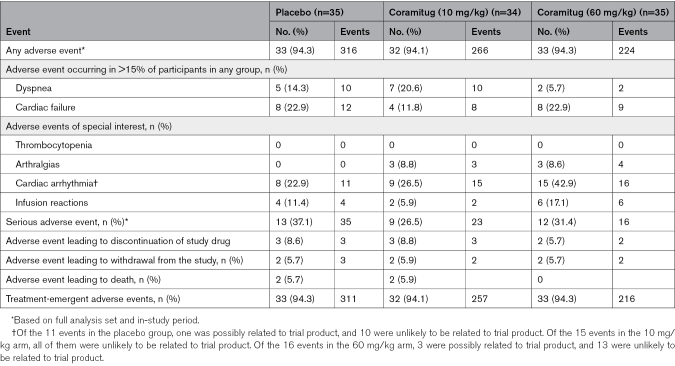
Adverse Events

The pharmacokinetic profile of coramitug was consistent with the characteristics of human immunoglobulin monoclonal antibodies. Exposure to coramitug, which was measured as the maximum concentration and the area under the curve over the dose interval, increased from 10 mg/kg to 60 mg/kg in an approximately dose-proportional manner. Among 69 patients with ATTR-CM tested with antidrug antibody assay, one patient had transient treatment-emergent antidrug antibody at one visit after administration of 60 mg/kg of coramitug.

**Figure 3.**
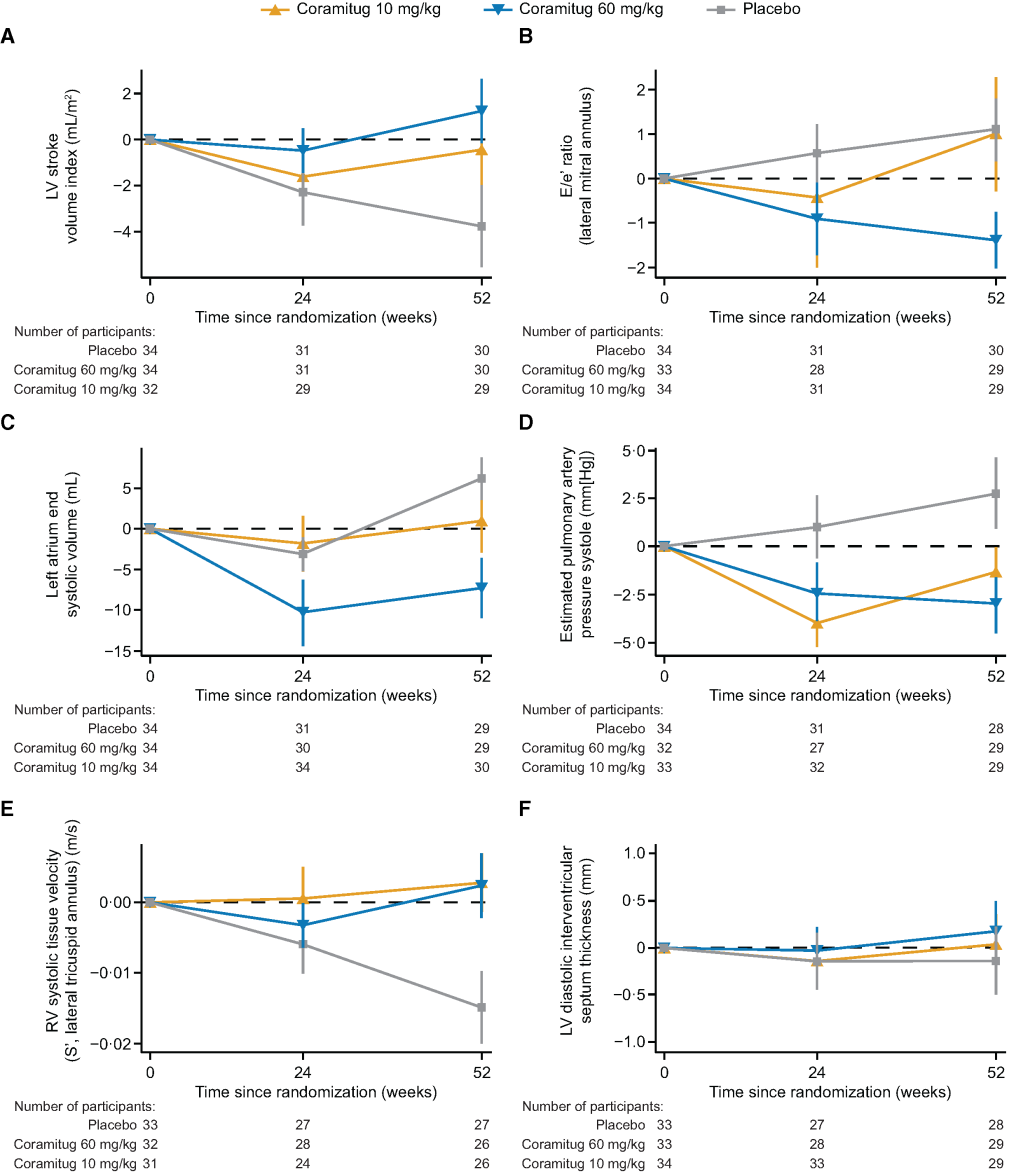
**Change from baseline over time in echocardiographic parameters. A**, LV stroke volume index. **B**, E/e’ ratio (lateral mitral annulus). **C**, Left atrium end-systolic volume. **D**, Estimated pulmonary artery pressure systole. **E**, RV systolic tissue velocity (S’, lateral tricuspid annulus). **F**, LV diastolic interventricular septum thickness (mm). Data shown are mean±SEM. LV indicates left ventricular; and RV, right ventricular.

Across a wide range of exploratory deformation-based and non–deformation-based echocardiographic parameters (Table S5), from baseline to week 52, coramitug 60 mg/kg was associated with an estimated treatment difference compared with placebo in stroke volume index (4.32 mL [95% CI, 0.29 to 8.35]), mitral valve A-wave peak velocity (+0.08 m/s [95% CI, 0.02 to 0.15]), left atrium end-systolic volume (−11.42 mL [95% CI, −20.52 to −2.32]), right ventricular systolic tissue velocity (0.02 m/s [95% CI, 0.01 to 0.03]), and estimated pulmonary artery systolic pressure (−4.06 mm Hg [95% CI, −7.75 to −0.37]; Table S5). There was no observed treatment difference in other echocardiographic parameters (Table S5). Change from baseline over time in echocardiographic parameters is shown in Figure 3.

## Discussion

In this phase 2, randomized, double-blind, placebo-controlled trial, treatment with the humanized monoclonal antibody coramitug at a dose of 60 mg/kg resulted in a statistically significant reduction in NT-proBNP compared with placebo over 52 weeks in participants with ATTR-CM, without a statistically significant improvement in 6MWT results. Coramitug at 10 mg/kg did not show statistical improvements compared with placebo in NT-proBNP or 6MWT. Coramitug had an acceptable safety profile and was generally well tolerated.

The observed improvement in NT-proBNP supports the mechanism of action of coramitug, which targets misfolded forms of TTR and may promote clearance of existing TTR amyloid deposits through immune-mediated mechanisms, such as antibody-dependent phagocytosis.^6,10^ Although coramitug is not the only antibody therapy in development for treatment of ATTR-CM,^5^ it represents a class of disease-modifying therapies aiming to remove existing amyloid rather than solely halting further deposition.^1^ Approved therapies reduce new amyloid production and slow disease progression, but they do not reverse established disease, and clinical events remain common despite treatment. Moreover, many patients are diagnosed at an advanced stage, when these therapies are less effective, highlighting the need for strategies that target existing amyloid deposits and offer the potential for functional recovery.

Patients on coramitug demonstrated a favorable reduction in NT-proBNP, despite the majority of patients (>80%) receiving standard of care (TTR stabilizer). Although no statistically significant difference was observed in functional capacity as measured by the 6MWT, several factors may account for this. First, the study was designed to detect a relatively large change in 6MWT (45 m) over 12 months, which may have limited the ability to capture modest but potentially meaningful improvements, particularly in a cohort where most participants were in New York Heart Association class II and had relatively preserved baseline physical function, similar to another trial in ATTR-CM.^11^ Second, functional changes may follow biomarker improvements and require longer follow-up.^1,3^ Third, 6MWT can be influenced by noncardiac factors and may not fully reflect early therapeutic effects.^12^ Last, almost all participants were receiving disease-modifying therapy, which is likely decreasing the rate of decline in the placebo arm.

Across a wide range of echocardiographic parameters, including left ventricular systolic function (stroke volume index), right ventricular systolic function (right ventricular peak systolic tissue velocity), diastolic function (mitral valve A-wave peak velocity and left atrial volume), and estimated pulmonary arterial pressures (reflective of transmitted left atrial pressures), coramitug at a dose of 60 mg/kg was associated with improvement compared with placebo. These observed changes are consistent with an improvement in cardiac systolic and diastolic function and hemodynamics, which, together with the observed decline in NT-proBNP, supports the proposed mechanism of action of coramitug, and may be indicative of potential cardiac remodeling. High-sensitivity troponin I, a marker of myocardial injury, did not differ significantly between treatment groups; however, a reduction from baseline was observed in the 60 mg/kg arm. Although exploratory, this finding is directionally consistent with the NT-proBNP results and may provide additional supportive evidence for a treatment effect on myocardial stress and injury.

Coramitug demonstrated an acceptable safety profile with no evidence of treatment-related toxicity and numerically fewer treatment-emergent adverse events than placebo. This is reassuring, especially given concerns that amyloid-targeting therapies might exacerbate inflammation or compromise myocardial function in heavily infiltrated hearts.

Notwithstanding the dramatic progress in patients with ATTR-CM, a significant percentage of patients are diagnosed at advanced stages when available therapies are less effective, and in such patients, efficacy takes longer to demonstrate compared with placebo.^13^ Currently approved therapies,^2–4,11^ although they reduce morbidity and mortality, do not reverse the disease but rather either stabilize or slow its progression. Thus, despite current approved therapies, affected patients continue to experience residual morbidity and mortality.^2–4,11^ In addition to facilitating earlier diagnosis, there remains an unmet need for new therapies that reverse the disease^14^ and improve cardiac structure and function and outcomes. Monoclonal antibodies targeting existing amyloid in the myocardium are promising therapies that, if shown to be effective in larger trials, could dramatically change the trajectory of patients with ATTR-CM.

This study has several limitations. The sample size was modest and the exposure time of 52 weeks relatively limited. There were missing data for cardiac magnetic resonance, which limited the likelihood to evaluate differences between treatment arms. Although global longitudinal strain was a prespecified secondary end point, the values reported by the core laboratory were not physiologically consistent with the expected range for ATTR-CM. An independent review by the authors (M.F. and S.J.S.) identified systematic measurement errors, rendering the global longitudinal strain data unreliable. Accordingly, these data were excluded from the present analysis. Although the enrolled population is representative of the diagnosed population with ATTR-CM, our findings may have limited generalizability to women and younger participants; in particular, the inclusion criterion for interventricular septal wall thickness (≥12 mm) was not normalized for sex and body size, which could lead to ascertainment bias against women with ATTR-CM and may explain the high proportion of men included in this study. In addition, the study included very few Black participants or participants with the p.Val142Ile mutation, the most commonly observed mutation in Black patients with ATTRv amyloidosis.

In summary, 60 mg/kg of coramitug significantly reduced NT-proBNP in a patient population in which the vast majority (≈80%) were already receiving standard of care treatment for ATTR-CM. Furthermore, compared with placebo, coramitug was associated with improvements in multiple echocardiographic parameters of cardiac function, and was well tolerated in participants with ATTR-CM. These findings support the potential of coramitug as an amyloid-clearing immunotherapy for ATTR-CM and provide a rationale for additional clinical investigation of coramitug for the treatment of patients with ATTR-CM.

## Article Information

### Acknowledgments

The authors would like to thank Dr Peter-Paul Zwetsloot from the Erasmus Medical Center, Rotterdam, Netherlands, for his critical review of the article, and Dr Bjarke Follin from Novo Nordisk A/S for review and interpretation of study results and imaging parameters. Editorial support was provided by Johanna Scheinost, DPhil, of Oxford PharmaGenesis, Oxford, United Kingdom, in accordance with the 2022 Good Publication Practice (GPP 2022) guidelines, with funding from Novo Nordisk A/S. P.G.-P., K.A., M.G., and S.J.S. were investigators who participated in the conduct of the study. B.M. was responsible for study conduct as sponsor representative. S.K. performed the statistical analyses. S.K., B.M., and S.J.S. directly accessed and verified the patient-level study data. K.A. reviewed and signed off on the clinical study report. M.F. and M.S.M. wrote the initial draft of the article, all authors critically reviewed and revised the article, and all authors had final responsibility for the decision to submit for publication.

### Sources of Funding

This study was funded by Novo Nordisk A/S.

### Disclosures

Dr Fontana reports consultancy for Alexion/Caelum Biosciences, Alnylam, AstraZeneca, Attralus, Bayer, BridgeBio/Eidos, Cardior, Intellia Therapeutics, Ionis Pharmaceuticals, Janssen Pharmaceuticals, Lexeo Therapeutics, MyCardium AI, Novo Nordisk, Pfizer, and Prothena; and research grants from Alnylam, AstraZeneca, BridgeBio, and Pfizer. She has share options in Lexeo Therapeutics and MyCardium AI. Dr Garcia-Pavia reports speaking fees from Alnylam Pharmaceuticals, AstraZeneca, Bayer, BridgeBio, Intellia, Ionis Pharmaceuticals, Novo Nordisk, and Pfizer; consulting fees from Alexion, Alnylam Pharmaceuticals, AstraZeneca, Attralus, Bayer, BridgeBio, Intellia, Ionis Pharmaceuticals, Life Molecular Imaging, Neuroimmune, Novo Nordisk, and Pfizer; and research/educational support to his institution from Alnylam Pharmaceuticals, AstraZeneca, Bayer, BridgeBio, Intellia, Novo Nordisk, and Pfizer. Dr Grogan reports consultancy fees to her institution from Alnylam, AstraZeneca, Attralus, and BridgeBio. She also reports research/educational support to her institution from Alnylam, AstraZeneca, Eidos/BridgeBio, and Intellia. She has participated on a data safety monitoring/advisory board for Janssen. She holds a pending license for an AI algorithm for the electrocardiogram detection of cardiac amyloid, Anumana, with potential future royalties. Dr Shah reports receiving research grants from the National Institutes of Health (Nos. U54 HL160273, R01 HL140731, and X01 HL169712) and American Heart Association (No. 24SFRNPCN1291224); research funding from AstraZeneca, Boston Scientific, Corvia, Pfizer, and Tempus; and consulting fees from 35Pharma, Aditum Bio, Adona, Alleviant, Alnylam, Amgen, AstraZeneca, Axon, BaroPace, Bayer, Boehringer Ingelheim, Boston Scientific, BridgeBio, Bristol Myers Squibb, Corvia, Cytokinetics, Diastol Therapeutics, Edwards Lifesciences, eMyosound, Ensho Health, Gordian, Intellia, Ionis, Lilly, Novo Nordisk, Prothena, Rivus, SalubrisBio, Sardocor, Secretome, Shifamed, Tectonic, Tenax, Ulink, and Ultromics. Drs Engelmann, Lim-Watson, Malling, Hovingh, and Revanna and S. Kar are employees and shareholders of Novo Nordisk. Dr Kristen reports consulting fees and honoraria from Novo Nordisk; he also reports participation on an advisory board for Novo Nordisk. Dr Sarswat reports research grants from the International Society of Amyloidosis and the Amyloidosis Research Consortium. She also reports payment of speaker fees to her institution from Alnylam, AstraZeneca, BridgeBio, and Novo Nordisk as well as meeting support from Alnylam and BridgeBio. She reports leadership roles in the International Society of Amyloidosis and the Heart Failure Society of America. Dr Tsujita reports speaking fees from Abbott Medical, Amgen, Bayer, Daiichi Sankyo, Kowa, Mochida, MSD, Novartis, Otsuka, Pfizer, Takeda, and Novo Nordisk; grant support from Japan Society for the Promotion of Science KAKENHI (Nos. 25K11366 and 22K08209), and research/educational support to his institution from Abbott Medical, Alexion, Amgen, Bayer, Boston Scientific, Daiichi Sankyo, Fidesone, GE HealthCare, ICON Clinical Research, ITI, Kowa, Medtronic, Mochida, MSD, Novartis, Otsuka, Otsuka Medical Devices, Pfizer, PPD-SNBL, Takeda, Novo Nordisk, and Roche Diagnostics. Dr Alexander reports consulting income from Alexion, Alnylam, Arbor Biotechnologies, Novo Nordisk, Pfizer, and Prothena. He also reports participation and personal fees in a data safety monitoring/advisory board for Kriya and stock options in Arbor Biotechnologies.

Dr Maurer reports grant support from the National Institutes of Health (Nos. R01HL177670 and AG081582) and research funding from Alnylam, Attralus, BridgeBio, Intellia, and Ionis; and personal fees from Alnylam, AstraZeneca, Bayer, Novo Nordisk, Intellia, Ionis, and Pfizer.

### Supplemental Material

List of trial sites and investigators

Data and safety monitory committee members

Event adjudication committee members

Supplemental methods, imaging

Narratives of death

Table S1: Inclusion and exclusion criteria

Table S2: Primary and secondary efficacy end points (observed data)

Table S3: NT-proBNP—analysis with mixed model for repeated measurements

Table S4: Adverse events occurring in >5% of patients in any group

Table S5: Echocardiography parameters

Redacted protocol: v1 and final version, with any changes from v1 listed

Redacted statistical analysis plan: v1 (final version)

## Supplementary Material


