# Supplementary material for: Coramitug, a Humanized Monoclonal Antibody for the Treatment of Transthyretin Amyloid Cardiomyopathy: A Phase 2, Randomized, Multicenter, Double-Blind, Placebo-Controlled Trial
Source: Circulation. 2025 Nov 10;153(4):214–25. doi: 10.1161/CIRCULATIONAHA.125.077304 (PMC12851541; doi:10.1161/CIRCULATIONAHA.125.077304)
Supplement: Supplementary file 1 [file cir-153-214-s001.pdf]

SUPPLEMENTAL MATERIAL

Coramitug, a Humanized Monoclonal Antibody for the Treatment of Transthyretin Amyloid Cardiomyopathy: A Phase 2, Randomized, Multicenter, Double-Blind, Placebo-Controlled Trial

Marianna Fontana, Pablo Garcia-Pavia, Martha Grogan, Sanjiv J. Shah, Mads D. M. Engelmann, G. Kees Hovingh, Arnt V. Kristen, Michelle Lim-Watson, Brian Malling, Soumitra Kar, Manjunatha Revanna, Nitasha Sarswat, Kenichi Tsujita, Kevin M. Alexander, Mathew S. Maurer

Contents

SUPPLEMENTAL MATERIAL 1

List of trial sites and investigators 2

Data monitoring committee (DMC) members 3

Event adjudication committee (EAC) members 3

Supplementary Methods. Imaging 4

Narratives of deaths 4

Supplementary tables 6

Table S1. Inclusion and Exclusion Criteria 6

Table S2. Primary and Secondary Efficacy Endpoints (Observed Data; No Imputation) 8

Table S3. NT-proBNP – Analysis with Mixed Model for Repeated Measurements 9

Table S4. Adverse Events Occurring in >5% of Patients in Any Group 10

Table S5. Echocardiography Parameters 11

Study protocol and SAP 12

List of trial sites and investigators

Country	Site	Investigator
Netherlands	UMC Groningen	Peter van der Meer
Netherlands	Universitair Medisch Centrum Utrecht	Marish Oerlemans
Germany	Universitätsklinik Heidelberg Innere Medizin III Kardiologie, Angiologie, Pneumologie	Fabian aus dem Siepen
Germany	Uniklinik Münster, Klinik für Kardiologie I	Ali Yilmaz
Germany	Universitatsklinikum Würzburg - Zentrum für Herzinsuffizienz	Caroline Morbach
Germany	LMU Klinikum München Klinik und Poliklinik 1	Stefan Kääb
Japan	Okayama University Hospital, Cardiovascular Medicine	Kazufumi Nakamura
Japan	Hiroshima University hospital, Cardiovascular Medicine	Yukiko Nakano
Japan	Hospital of the University of Occupational and Environmental Health, Cardiology, Nephrology	Masaharu Kataoka
Japan	Kumamoto University Hospital, Cardiovascular Medicine	Naoto Kuyama
Japan	Nagasaki University Hospital, Cardiovascular Medicine	Hiroaki Kawano
Japan	Shinshu University Hospital, Department of Neurology	Yoshiki Sekijima
Italy	Centro per lo Studio e la Cura delle Amiloidosi Sistemiche Fondazione IRCCS Policlinico San Matteo	Giovanni Palladini
Italy	Fondazione CNR-Regione Toscana Gabriele Monasterio	Michele Emdin
Czech Republic	II. interni klinika VFN - Kardiologie a angiologie	Tomas Palecek
France	CENTRE HOSPITALIER UNIVERSITAIRE DE TOULOUSE-HOPITAL RANGUEIL-1	Olivier Lairez
France	AP-HP-HOPITAL HENRI MONDOR	Thibaud Damy
Portugal	Unidade Local de Saude do Alto Ave, E.P.E.	Olga Azevedo
Portugal	Unidade Local De Saude De Tras-Os-Montes E Alto Douro E.P.E.	Catarina Ferreira
Spain	Hospital Universitario Puerta de Hierro Majadahonda	Pablo García Pavía
Canada	University of Calgary, Cardiology	Nowell Fine
Canada	Ctr for Cardiovascular Innovation	Margot Davis
United States	Oregon Hlth Sci Univ-Portland	Ahmad Masri
United States	Stanford Hlth Cre–Boswell Clin	Kevin Alexander
United States	Cedars-Sinai Medical Center, Los Angeles	Robert Cole
United States	Univ of MD Schl of Med	Stephen Gottlieb
United States	Mayo Clinic Rochester	Martha Grogan
United States	Mayo Clinic Jacksonville	Melissa Lyle
United States	Mayo Clinic Arizona	Julie Rosenthal
United States	NW Univ-Bluhm Cardiovasc Inst	Sanjiv Shah

Data monitoring committee (DMC) members

Function	Member
**DMC chair**	Rodney H. Falk<br>Brigham and Women's Hospital, Harvard Medical School, Boston, US<br>Field of expertise: Cardiology
**DMC statistician**	Kerry L. Lee<br>Department of Biostatistics and Bioinformatics, Duke University, US<br>Field of expertise: Statistics
**DMC member**	Marcia Waddington-Cruz<br>Hospital Universitario Clementino Fraga Filho, Federal University of Rio de Janeiro, Brazil<br>Field of expertise: Neurology
**DMC member**	Francesco Cappelli<br>Azienda Ospedaliero Universitaria Careggi, Italy<br>Field of expertise: Cardiology

Event adjudication committee (EAC) members

Function	Member
**EAC chair**	David Kong, MD<br>Associate Professor of Medicine<br>Duke Clinical Research Institute<br>Area of expertise: Cardiologist
**EAC member**	Christopher Kontos, MD<br>Professor of Medicine<br>Duke University Medical Center<br>Area of expertise: Cardiologist
**EAC member**	Rajendra Mehta, MD<br>Consulting Professor in the Department of Medicine<br>Duke Clinical Research Institute<br>Area of expertise: Cardiologist
**EAC member**	J. Dedrick Jordan, MD, PhD<br>Chair, Department of Neurology, LSU Health Shreveport<br>Area of expertise: Neurologist
**EAC member**	Keith Dombrowski, MD<br>Assistant Professor<br>Department of Neurology and Neurosurgery<br>University of South Florida<br>Area of expertise: Neurologist

Supplementary Methods. Imaging

The procedures for the acquisition, collection, handling, quality review, and analysis of imaging data were standardized and a core imaging lab (Clario, PA, USA) performed a central read of echocardiography and cardiac magnetic resonance imaging to ensure consistency in the assessment of imaging endpoints longitudinally and across patients. Central readers were blinded to treatment and applied consistent methods, software, and expertise to the measurements and assessments. The physicians performing the reading were board certified in cardiology with specialty training within the designated modality being read, and reviewer training occurred prior to starting on-study reads. Sites trained in the imaging manual and then qualified after submitting a test scan to verify the required imaging parameters were correctly set and the image quality was acceptable.

Echocardiography

Comprehensive echocardiography using a standardised, pre-defined protocol was performed prior to infusion 5 times throughout the study, from randomisation to end of treatment (week 52). Participants in the sentinel cohort had additional ECG and echocardiography evaluated by the investigator before the planned second dosing. Echocardiography measurements were made across 3 cardiac cycles (or 5 if ectopy or atrial fibrillation was present) and averaged. If less than 3 evaluable cardiac cycles (or 5 if ectopy/arrhythmia) were present, measurements were made on remaining evaluable cardiac cycles. All echocardiographic measurements were made by readers blinded to all clinical data and treatment assignment using TOMTEC imaging software (TomTec, Munich, Germany).

Cardiac Magnetic Resonance Imaging (MRI)

Cardiac MRI was performed at the same time points for echocardiography unless the participant had a contraindication to MRI according to local standards. The sites used the same approved MRI scanner for all scheduled MRI scans. Measurement of hematocrit for the calculation of ECV was obtained immediately before the MRI scan, if possible; otherwise, it was obtained within ± 3 days of scanning. Centralized reading was performed using cvi42 (Circle Cardiovascular Imaging, AB, Canada). The automated T1 and ECV maps were generated using the 4-chamber view after adding the pre- and post-contrast 4-chamber T1 mapping series.

Narratives of deaths

A total of 5 TEAEs that led to death were reported in 4 participants; 2 participants (5.9%) in the 10 mg/kg group (atrial fibrillation, infection, and ventricular fibrillation), and 2 participants (5.7%) in the placebo group (syncope death due to infection and acute pancreatitis).

Participant ID 905001: A 67-year-old participant with hATTR (NYHA stage II, BMI 29.7 kg/m^2) was on placebo for 6 months before falling backwards from the stairs to the pool, likely due to syncope. After being rescued, resuscitation efforts began, but hospital notes indicated recurrent pulseless electrical activity. Despite advanced cardiac life support and pressor support, the patient suffered profound hypotension and remained unresponsive; life support was withdrawn the next day. This patient also had a significant history of cardiovascular disease and prior syncope episodes.

Participant ID 200005: An 85-year-old participant with ATTRwt (NYHA stage II, BMI 24.2 kg/m^2 was assigned to coramitug 10 mg/kg for 7 months before the event. The participant's medical history contains heart failure, coronary artery disease, hypertension, and aortic, mitral, and tricuspid valve insufficiency. Previously, the participant had left

anterior hemiblock and torsades de pointes. Participant passed away due to refractory ventricular fibrillation. The event was likely due to age and underlying disease.

Participant ID 906006: An 85-year-old participant with ATTRwt (NYHA stage II, BMI 25.2 kg/m^2) was assigned to coramitug 10 mg/kg for 6 months before the event. The participant's medical history contained persistent atrial fibrillation, heart failure, hypertension, and previous smoking. The participant passed away due to atrial fibrillation with rapid ventricular response and unspecified infection. The events were likely due to underlying disease.

Participant ID 301012: An 82-year-old participant with ATTRwt (NYHA stage II, BMI 25.7 kg/m^2) was assigned to placebo for 12 months before the event. The participant's medical history contains diabetes mellitus, hyperuricemia, hypothyroidism, persistent atrial fibrillation, and diverticulosis. The participant was diagnosed with choledochal calculi, signs of cholecystitis and pancreatitis, and rapidly evolved towards septic shock, multiorgan failure, and death. The choledochal calculi were reported to be present explaining the etiology of the disease.

Supplementary tables

Table S1. Inclusion and Exclusion Criteria

**Inclusion criteria**
Participants are eligible to be included in the study only if all the following criteria apply:<br>1. Informed consent obtained before any study-related activities. Study-related activities are any procedures that are carried out as part of the study, including activities to determine suitability for the study.<br>2. Male or female.<br>3. Age ≥18 to <85 years at the time of signing informed consent.<br>4. Have an established diagnosis of ATTR-CM with either wild-type TTR or hereditary TTR genotype as per local standards.[a]<br>5. Expected to be on stable doses of cardiovascular medical therapy 6 weeks prior to the randomization visit.<br>6. Known[b] end-diastolic interventricular septal wall thickness ≥12 mm.<br>7. Presently classified as New York Heart Association Class II–III.<br>8. NT-proBNP concentration ≥650 pg/mL in sinus cardiac rhythm and >1000 pg/mL in atrial fibrillation at screening.<br>9. Completed ≥150 meters to ≤450 meters on the six-minute walk test (6MWT) at screening.<br>10. Absolute neutrophil count $\geq 2.0 \times 10^9$/L; platelet count $\geq 120 \times 10^9$/L at screening.<br>11. Aspartate transaminase (AST) and alanine transaminase (ALT) levels ≤ 2.5 × the upper limit of normal (ULN) and total bilirubin ≤2 × ULN at screening.<br>12. Estimated glomerular filtration rate (eGFR) ≥25 mL/min/1.73 m^2 at screening.
**Exclusion criteria**
Participants are excluded from the study if any of the following criteria apply:<br>1. Known or suspected hypersensitivity to study intervention(s) or related products.<br>2. Previous dosing in this study.<br>3. Female who is pregnant, breast-feeding, or intends to become pregnant or is of childbearing potential and not using highly effective contraceptive method.<br>4. Use of another approved or non-approved investigational medicinal product within 30 days or 5 half-lives of the investigational medicinal product (whichever is longer) before screening.<br>5. Any disorder, which in the investigator's opinion might jeopardize participant's safety or compliance with the protocol.<br>6. Current diagnosis or history of amyloid light chain or other non-ATTR amyloidosis<br>7. Cardiomyopathy not primarily caused by ATTR-CM, for example, cardiomyopathy due to hypertension, valvular heart disease, or ischemic heart disease.<br>8. A prior solid organ transplant.

9. Planned solid organ transplant during the study.
10. Presence or history of malignant neoplasm (other than basal or squamous cell skin cancer, in-situ carcinomas of the cervix, or in-situ/high grade prostatic intraepithelial neoplasia (PIN) or low-grade prostate cancer) within 5 years before screening.
11. Current treatment with calcium channel blockers with conduction system effects (eg, verapamil, diltiazem). The use of dihydropyridine calcium channel blockers is allowed. The use of digoxin will only be allowed if required for management of atrial fibrillation with rapid ventricular response.
12. Acute coronary syndrome, unstable angina, stroke, transient ischemic attack (TIA), coronary revascularization, cardiac valve repair, or major surgery within 3 months of screening.
13. Body weight >120 kg (264.6 lb) at screening.
14. Evidence of current or chronic hepatitis C virus or hepatitis B virus infection.
15. History of or known seropositivity for human immunodeficiency virus (HIV).
16. International normalized ratio (INR) >1.5 (unless participant is on anticoagulant therapy,[c] in which case excluded if INR >3.5).[d]
17. History of contrast allergy or adverse reactions to gadolinium-containing agents.

[a]Non-invasive diagnostic pathways were confirmed by a centralized expert review. [b]Medical history/records are accepted. [c]Vitamin K antagonists (ie, warfarin, acenocoumarol etc.). [d]Criterion not applicable for participants on therapy with direct-acting oral anticoagulants. ATTR-CM indicates transthyretin amyloid cardiomyopathy; NT-proBNP, N-terminal pro B-type natriuretic peptide; and TTR, transthyretin.

Table S2. Primary and Secondary Efficacy Endpoints (Observed Data; No Imputation)

	Placebo (n=35)	Coramitug 10 mg/kg (n=34)	Coramitug 60 mg/kg (n=35)
**Primary endpoints**			
**NT-proBNP (pg/mL)**			
n (observed participants' data at both baseline and week 52)	31	31	33
Observed **ratio** to baseline at week 52	1.31	1.02	0.84
**6-minute walk test (m)**			
n (observed participants' data at both baseline and week 52)	30	31	33
Observed **change** to baseline at week 52	−5.40	13.06	−1.06
**Secondary endpoints**			
**ECV (%)**			
n (observed participants' data at both baseline and week 52)	6	9	9
Observed **change** to baseline at week 52	4.83	0.09	3.32
**KCCQ-CSS (point)**			
n (observed participants' data at both baseline and week 52)	31	31	33
Observed **change** to baseline at week 52	−2.61	−0.13	−1.97
**High-sensitivity troponin I (μg/L)**			
n (observed participants' data at both baseline and week 52)	31	31	33
Observed **ratio** to baseline at week 52	0.063	0.038	0.050
**NIS (score)**			
n (observed participants' data at both baseline and week 52)	3	2	2
Observed **change** to baseline at week 52 (mean [standard deviation])	12.00 (15.10)	9.75 (15.20)	10.50 (2.12)

CI indicates confidence interval; ECV, extracellular volume; eGFR, estimated glomerular filtration rate; IQR, interquartile range; KCCQ-CSS, Kansas City Cardiomyopathy Questionnaire Clinical Summary Score; NAC, National Amyloidosis Centre; NIS, Neuropathy Impairment Score; NT-proBNP, N-terminal pro-brain natriuretic peptide.

Table S3. NT-proBNP – Analysis with Mixed Model for Repeated Measurements

Treatment group	Treatment ratio (95% CI), *P*-value
**Primary analysis**	
10 mg vs placebo	0.72 (0.49; 1.07), *P*=0.1043
60 mg vs placebo	0.52 (0.35; 0.78), *P*=0.0017
**MMRM (with all observed data – treatment policy estimand)**	
10 mg vs placebo	0.75 (0.5593; 0.9993), *P*=0.0495
60 mg vs placebo	0.62 (0.4644; 0.8209), *P*=0.0011

The log-transformed ratio of responses to baseline was analyzed using a MMRM with an unstructured covariance matrix, visit and stratification factors as fixed effects, with the logarithm of the baseline response as a covariate. Additionally, the interactions between visit and log-transformed baseline response, stratification factor has been included in the model.

CI indicates confidence interval; MMRM, mixed model for repeated measurements; NT-proBNP, N-terminal pro-brain natriuretic peptide.

Table S4. Adverse Events Occurring in >5% of Patients in Any Group

	Placebo (n=35)		Coramitug 10 mg/kg (n=34)		Coramitug 60 mg/kg (n=35)	
	n (%)	Events	n (%)	Events	n (%)	Events
**Nasopharyngitis**	3 (8.6)	3	4 (11.8)	6	2 (5.7)	2
**COVID-19**	2 (5.7)	2	1 (2.9)	1	4 (11.4)	5
**Fatigue**	4 (11.4)	4	4 (11.8)	5	5 (14.3)	5
**Edema peripheral**	2 (5.7)	2	4 (11.8)	4	2 (5.7)	2
**Asthenia**	4 (11.4)	9	3 (8.8)	7	1 (2.9)	3
**Dyspnea**	5 (14.3)	10	7 (20.6)	10	2 (5.7)	2
**Cough**	3 (8.6)	5	4 (11.8)	8	4 (11.4)	5
**Cardiac failure**	8 (22.9)	12	4 (11.8)	8	8 (22.9)	9
**Atrial fibrillation**	2 (5.7)	3	3 (8.8)	5	4 (11.4)	4
**Dizziness**	4 (11.4)	7	5 (14.7)	6	3 (8.6)	3
**Paresthesia**	4 (11.4)	4	1 (2.9)	1	1 (2.9)	1
**Diarrhea**	5 (14.3)	7	4 (11.8)	6	2 (5.7)	6
**Fall**	2 (5.7)	2	3 (8.8)	7	4 (11.4)	4
**Hematuria**	3 (8.6)	3	2 (5.9)	5	2 (5.7)	4

Table S5. Echocardiography Parameters

	Placebo (n=35)	Coramitug 10 mg/kg (n=34)	Coramitug 60 mg/kg (n=35)
**Echocardiographic parameters**			
**Left ventricle E/e' ratio**			
n (observed participants' data at both baseline and week 52)	30	29	29
Observed change to baseline at week 52	1.09	0.99	−1.39
Estimated change to baseline at week 52	0.78	1.31	-1.38
Estimated treatment difference (95% CI)		0.53 (−1.79, 2.85)	−2.16 (−4.47, 0.15)
**Mitral valve - A-wave peak velocity (m/s)**			
n (observed participants' data at both baseline and week 52)	22	15	20
Observed change to baseline at week 52	−0.05	−0.09	0.04
Estimated change to baseline at week 52	−0.05	−0.08	0.03
Estimated treatment difference (95% CI)		−0.03 (−0.1, 0.05)	0.08 (0.02, 0.15)
**Left atrium end systolic volume (mL)**			
n (observed participants' data at both baseline and week 52)	29	30	29
Observed change to baseline at week 52	6.16	1.02	−7.27
Estimated change to baseline at week 52	4.76	1.78	−6.66
Estimated treatment difference (95% CI)		−2.97 (−12.03, 6.08)	−11.42 (−20.52, −2.32)
**Left ventricle diastolic interventricular septum thickness (mm)**			
n (observed participants' data at both baseline and week 52)	28	29	29
Observed change to baseline at week 52	−0.14	0.03	0.17
Estimated change to baseline at week 52	−0.08	0.21	−0.07
Estimated treatment difference (95% CI)		0.3 (−0.55, 1.15)	0.02 (−0.84, 0.87)
**Right ventricle systolic tissue velocity (S' lateral tricuspid annulus) (m/s)**			
n (observed participants' data at both baseline and week 52)	27	26	26
Observed change to baseline at week 52	−0.01	0	0
Estimated change to baseline at week 52	−0.01	0	0
Estimated treatment difference (95% CI)		0.01 (0, 0.03)	0.02 (0.01, 0.03)
**Left ventricle stroke volume (mL)**			
n (observed participants' data at both baseline and week 52)	30	29	30
Observed change to baseline at week 52	−6.14	−1.14	−0.04
Estimated change to baseline at week 52	−5.61	−1.29	−0.42
Estimated treatment difference (95% CI)		4.32 (−3.02, 11.67)	5.19 (−2.07, 12.45)
**Estimated pulmonary artery pressure systole (mmHg)**			
n (observed participants' data at both baseline and week 52)	28	29	29
Observed change to baseline at week 52	2.76	−1.33	−2.99
Estimated change to baseline at week 52	1.62	−0.79	−2.43
Estimated treatment difference (95% CI)		−2.42 (−6.11, 1.27)	−4.06 (−7.75, −0.37)
**Tricuspid valve peak velocity (m/s)**			
n (observed participants' data at both baseline and week 52)	28	29	29
Observed change to baseline at week 52	0.14	−0.06	−0.13
Estimated change to baseline at week 52	0.08	−0.03	−0.11
Estimated treatment difference (95% CI)		−0.12 (−0.29, 0.05)	−0.19 (−0.36, −0.02)

Based on observed data from the in-study period. Change from baseline measurements are analyzed using an analysis of covariance model with treatment and stratification factor as fixed effects and baseline measurement as covariate. P-values are two-sided p-values for test of no treatment difference. No correction for multiplicity. These analyses were exploratory and not pre-specified.

CI indicates confidence interval; LV, left ventricle; and MV, mitral valve.

Study protocol and SAP

Redacted protocol: v1 and final version, with any changes from v1 listed

Redacted SAP: v1 (final version)

Protocol

Protocol Title: Efficacy and safety of NNC6019-0001 at two dose levels in participants with transthyretin amyloid cardiomyopathy (ATTR CM)

Substance name: NNC6019-0001

Universal Trial Number: U1111-1271-3861

EudraCT Number: 2021-006226-49

IND Number: 133801

Study phase: 2

In the following, Novo Nordisk A/S and its affiliates will be stated as "Novo Nordisk".

This confidential document is the property of Novo Nordisk. No unpublished information contained herein may be disclosed without prior written approval from Novo Nordisk. Access to this document must be restricted to relevant parties.

Table of Contents

Page

Table of Contents 2

1 Protocol summary 6
1.1 Synopsis 6
1.2 Flowchart 9

2 Introduction 17
2.1 Study rationale 17
2.2 Background 18
2.3 Benefit-risk assessment 19
2.3.1 Risk assessment 19
2.3.2 Benefit assessment 20
2.3.3 Overall benefit-risk conclusion 21

3 Objectives, endpoints and estimands 22

4 Study design 25
4.1 Overall design 25
4.2 Scientific rationale for study design 25
4.3 Justification for dose 27
4.4 End of study definition 27

5 Study population 28
5.1 Inclusion criteria 28
5.2 Exclusion criteria 28
5.3 Lifestyle considerations 29
5.3.1 Activity 29
5.4 Screen failures 29
5.5 Randomisation criteria 30

6 Study interventions and concomitant therapy 31
6.1 Study interventions administered 31
6.2 Preparation, handling, storage and accountability 35
6.3 Measures to minimise bias: Randomisation and blinding 37
6.3.1 Randomisation 37
6.3.2 Blinding 37
6.3.3 Blind Break 37
6.4 Study intervention compliance 38
6.5 Dose modification 38
6.6 Continued access to study intervention after end of study 38
6.7 Treatment of overdose 39
6.8 Concomitant therapy 39

7 Discontinuation of study intervention and participant discontinuation/withdrawal 41
7.1 Discontinuation of study intervention 41
7.1.1 Temporary discontinuation of study intervention 42
7.2 Participant discontinuation/withdrawal from the study 42
7.2.1 Replacement of participants 43
7.3 Lost to follow-up 43

8 Study assessments 44
8.1 Screening 45
8.2 Efficacy assessments 46
8.2.1 Clinical efficacy laboratory assessments 46
8.2.1.1 Urine collection 46

8.2.2 Clinical outcome assessments 46
8.2.2.1 6-minute walk test (6-MWT) 46
8.2.2.2 Neuropathy Impairment Score (NIS) 46
8.2.2.3 Patient reported outcome (PRO) questionnaires 46
8.2.3 Imaging....47
8.2.3.1 Cardiac magnetic resonance imaging (MRI) 47
8.2.3.2 Echocardiography 48
8.3 Safety assessments 48
8.3.1 Physical examinations 49
8.3.2 New York Heart Association (NYHA) classification 49
8.3.3 Body measurements....49
8.3.4 Vital signs....50
8.3.5 Electrocardiograms....50
8.3.6 Cardiac monitoring (only appliable for sentinel participants)....51
8.3.7 Clinical safety laboratory assessments 51
8.3.8 Pregnancy testing....51
8.4 Adverse events and other safety reporting....52
8.4.1 Time period and frequency for collecting AE information 52
8.4.2 Method of detecting AEs....53
8.4.3 Follow-up of AEs 53
8.4.4 Regulatory reporting requirements for SAEs 53
8.4.5 Pregnancy 53
8.4.6 Cardiovascular and death events 53
8.4.7 Technical complaints....54
8.5 Pharmacokinetics and pharmacodynamics 54
8.5.1 Pharmacokinetics....54
8.6 Genetics 54
8.7 Biomarkers....54
8.8 Immunogenicity assessments....55
8.8.1 Anti-NNC6019-0001-antibodies 55
8.8.2 Assessments in case of suspicion of hypersensitivity to trial product....56
8.8.3 Human biosamples for future research....56
8.9 Health economics....57

9 Statistical considerations 58
9.1 Statistical hypotheses....58
9.1.1 Multiplicity adjustment....58
9.2 Analysis sets....58
9.3 Statistical analyses 58
9.3.1 General considerations 58
9.3.2 Primary endpoint analysis 58
9.3.3 Secondary endpoints analysis....59
9.3.3.1 Supportive secondary endpoints 59
9.3.4 Exploratory endpoints analysis....60
9.3.5 Other safety analyses....60
9.3.6 Other analyses 60
9.3.6.1 Pharmacokinetic and pharmacodynamic modelling 60
9.4 Interim analysis....60
9.5 Sample size determination 61

10 Supporting documentation and operational considerations....63
10.1 Appendix 1: Regulatory, ethical, and study oversight considerations....63
10.1.1 Regulatory and ethical considerations....63
10.1.2 Financial disclosure....63
10.1.3 Informed consent process....64
10.1.4 Information to participants during the study 64

10.1.5 Data protection 64
10.1.6 Committee structure 65
10.1.6.1 Novo Nordisk safety committee....65
10.1.6.2 Data monitoring committee....65
10.1.6.3 Steering Committee....65
10.1.6.4 Event adjudication committee....66
10.1.7 Dissemination of clinical study data....66
10.1.8 Data quality assurance....66
10.1.8.1 Case report forms....66
10.1.8.2 Monitoring....67
10.1.8.3 Protocol compliance....68
10.1.9 Source documents....68
10.1.10 Retention of clinical study documentation....68
10.1.11 Study and site closure....69
10.1.12 Responsibilities....69
10.1.13 Indemnity statement....70
10.1.14 Publication policy....70
10.1.14.1 Communication of results....71
10.1.14.2 Authorship....71
10.1.14.3 Site-specific publication(s) by investigator(s)....71
10.1.14.4 Investigator access to data and review of results....72
10.2 Appendix 2: Clinical laboratory tests....73
10.3 Appendix 3: Adverse Events and Serious Adverse Events: Definitions and procedures for recording, evaluating, follow-up, and reporting....75
10.3.1 Definition of AE....75
10.3.2 Definition of an SAE....75
10.3.3 Description of AEs requiring additional data collection....76
10.3.4 Recording and follow-up of AE and/or SAE....77
10.3.4.1 AE and SAE recording....77
10.3.4.2 Assessment of severity....78
10.3.4.3 Assessment of causality....78
10.3.4.4 Final outcome....79
10.3.4.5 Follow-up of AE and SAE....79
10.3.5 Reporting of SAEs....80
10.4 Appendix 4: Contraceptive guidance and collection of pregnancy information....82
10.4.1 Definitions....82
10.4.2 Contraceptive guidance....82
10.4.3 Collection of pregnancy information....83
10.5 Appendix 5: Technical complaints: Definition and procedures for recording, evaluation, follow-up and reporting....85
10.5.1 Definition of technical complaint....85
10.5.2 Recording and follow-up of technical complaints....85
10.5.3 Reporting of technical complaints for products not included in the technical complaint form....86
10.6 Appendix 6: Retention of human biosamples for future research....87
10.6.1 Biosamples for future research....87
10.6.2 Anti-NNC6019-0001-antibodies samples....88
10.6.3 Hypersensitivity reaction samples....88
10.6.4 Pharmacokinetic and pharmacodynamic samples....89
10.7 Appendix 7: Events requiring adjudication....90
10.8 Appendix 8: Country-specific requirements....91
10.9 Abbreviations....94

11 References....97

Protocol attachment I Global list of key staff and relevant departments and suppliers

Protocol attachment II Country list of key staff and relevant departments

1 Protocol summary

1.1 Synopsis

This is an interventional, randomised, multinational, multicentre, three-arm parallel-group, double-blind, placebo-controlled study in participants with hATTR or wtATTR CM.

Rationale

Transthyretin amyloid cardiomyopathy (ATTR CM) is an increasingly recognised cause of heart failure in older adults worldwide, resulting from extracellular deposition of misfolded transthyretin protein (amyloid) in the myocardium.[1] ATTR CM is a progressive chronic disease with a high burden for patients and society, underscoring the need for therapies that reverse disease pathology and lower the risk of worsening of heart failure, hospitalisation and mortality in patients with ATTR CM. NNC6019-0001 is designed to have an amyloid-depleting mechanism of action for patients at high risk of early mortality due to amyloid deposition in the myocardium. The aims of this proof-of-principle study are to compare the effect of NNC6019-0001 versus placebo on functional endpoints, circulating- and imaging biomarkers as well as to evaluate pharmacokinetics, safety, and tolerability of the two dose levels covering the relevant therapeutic levels and based on the totality of data to select the dose to be studied in phase 3.

Objectives, endpoints and estimands

The primary and secondary objectives and endpoints are summarised in the table below.

Objectives	Endpoints		
**Primary**	**Title**	**Time frame**	**Unit**
• To compare the effect of two dose levels of NNC6019-0001 (30 mg/kg and 100 mg/kg) versus placebo on:<br>• change in 6-minute walk test and<br>• change in NT-proBNP<br>from baseline to week 52 in participants with hATTR or wtATTR cardiomyopathy.	*Primary*		
	Change in 6-minute walk test (6-MWT)	From baseline (week 0) to visit 15 (week 52)	Meters
	Change in NT-proBNP	From baseline (week 0) to visit 15 (week 52)	Percentage
**Secondary**	**Title**	**Time frame**	**Unit**
• To compare the effect of two dose levels of NNC6019-0001 (30 mg/kg and 100 mg/kg) versus placebo on:<br>• biomarkers<br>• pharmacodynamic endpoints<br>from baseline to week 52 in participants with hATTR or wtATTR cardiomyopathy.	*Supportive*		
	Change in myocardial extracellular volume (ECV)	From baseline (week 0) to visit 15 (week 52)	%-points
	Change in Kansas City Cardiomyopathy Questionnaire (KCCQ) Clinical Summary Score[a] (CSS)[2]	From baseline (week 0) to visit 15 (week 52)	Score
	Change in neuropathy impairment score[b] (NIS)	From baseline (week 0) to visit 15 (week 52)	Score
	Change in troponin I	From baseline (week 0) to visit 15 (week 52)	ng/mL

Objectives	Endpoints		
	Change in global longitudinal strain (GLS) on echocardiography	From baseline (week 0) to visit 15 (week 52)	%-points
• To compare the effect of two dose levels of NNC6019-0001 (30 mg/kg and 100 mg/kg) versus placebo on:<br>• safety and tolerability<br>from baseline to week 64 in participants with hATTR or wtATTR cardiomyopathy.	Number of treatment emergent adverse events	From baseline (week 0) to visit 16 (week 64)	Count
	Time to occurrence of all-cause mortality	From baseline (week 0) to visit 16 (week 64)	Weeks
	Number of CV events comprising hospitalisation due to CV events or urgent heart failure visits	From baseline (week 0) to visit 16 (week 64)	Count

[a] Clinical Summary Score (CSS) consists of the Symptom domain and the Physical Limitation domain, additional analyses of the remaining domains will be described in the SAP. KCCQ scores range from 0 to 100 and lower scores represent more severe symptoms and/or limitations and scores of 100 indicate no symptoms, no limitations, and excellent quality of life; [b] Only applicable for participants with hATTR CM. The total NIS score is graded on a scale of 0–244, with a higher score indicating greater impairment.
Abbreviations: CV = cardiovascular; hATTR = hereditary ATTR; NT-proBNP = N-terminal-pro brain natriuretic peptide; wtATTR = wild-type ATTR.

Primary estimand

The primary estimand addresses the following question of interest: What is the effect of two dose levels of NNC6019-0001 (30 mg/kg and 100 mg/kg) versus placebo on change in 6-MWT and NT-proBNP from baseline to week 52, or occurrence of death or CV hospitalisation, in participants with hATTR or wtATTR CM, regardless of premature discontinuation of study intervention.

Overall design

This is an interventional, randomised, multinational, multicentre, three-arm parallel-group, double-blind, placebo-controlled study comparing i.v. NNC6019-0001 Q4W at two dose levels (30 mg/kg and 100 mg/kg) versus placebo in participants with hATTR or wtATTR CM.

The study consists of a screening period of up to 8 weeks, followed by a 52-week intervention period. When participants discontinue study intervention according to protocol, an end of treatment visit should be carried out 4 weeks after administration of the last dose and a follow-up visit should be carried out 16 weeks after administration of the last dose.

Study intervention groups, duration, and number of participants

Study intervention groups:
- 30 mg/kg NNC6019-0001 lyophilised powder for solution for i.v. infusion
- 100 mg/kg NNC6019-0001 lyophilised powder for solution for i.v. infusion
- Placebo

Following the screening period, approximately 99 participants will be randomised 1:1:1 to receive i.v. 30 mg/kg NNC6019-0001, 100 mg/kg NNC6019-0001 or placebo Q4W added to standard of care. Randomisation will be stratified by disease type (wtATTR vs hATTR) and maximum 80% of participants randomised will be participants with wtATTR.

The planned study duration for the individual participant will be approximately 64 weeks (excluding screening).

Participant characteristics

Key inclusion criteria:

- Male or female.
- Age ≥ 18 to < 85 years at the time of signing informed consent.
- Have an established diagnosis of ATTR CM with either wild-type TTR or hereditary TTR genotype as per local standards.
- Expected to be on stable doses of cardiovascular medical therapy 6 weeks prior to the randomisation visit.
- Known end-diastolic interventricular septal wall thickness ≥ 12 mm.
- Presently classified as New York Heart Association (NYHA) Class II-III.
- NT-proBNP concentration ≥650 pg/mL in sinus cardiac rhythm and >1000 pg/mL in atrial fibrillation at screening.
- Completed ≥150 meters to ≤450 meters on the 6-MWT at screening.
- Estimated glomerular filtration rate (eGFR) ≥25 mL/min/1.73 m^2 at screening.

Key exclusion criteria:

- Cardiomyopathy not primarily caused by ATTR CM, for example, cardiomyopathy due to hypertension, valvular heart disease, or ischemic heart disease
- A prior solid organ transplant.
- Planned solid organ transplant during the study.
- Presence or history of malignant neoplasm (other than basal or squamous cell skin cancer, in-situ carcinomas of the cervix, or in-situ/high grade prostatic intraepithelial neoplasia (PIN) or low-grade prostate cancer) within 5 years before screening.
- Current treatment with calcium channel blockers with conduction system effects (e.g., verapamil, diltiazem). The use of dihydropyridine calcium channel blockers is allowed. The use of digoxin will only be allowed if required for management of atrial fibrillation with rapid ventricular response.
- Acute coronary syndrome, unstable angina, stroke, transient ischemic attack (TIA), coronary revascularization, cardiac valve repair, or major surgery within 3 months of screening.
- Body weight >120 kg (264.6 lb) at screening.
- History of contrast allergy or adverse reactions to gadolinium-containing agents.

Efficacy and safety data will be collected at regular intervals throughout the study.

Data monitoring committee
Yes.

1.2 Flowchart

Procedure	Protocol Section	Screening		Randomisation	Study intervention period															End of treatment	Follow-up
Visit		V1	V1A[a]	V2	V2A[b]	V2B[b]	V2C[b]	V2D[b]	V3	V4	V5	V6	V7	V8	V9	V10	V11	V12	V13	V14	V15	V16[c]
Timing of Visit (weeks)		up to -8 weeks	up to -2 weeks	0	24h	1	2	3	4	8	12	16	20	24	28	32	36	40	44	48	52	64
Visit Window (Days)				0	0	+2	±2	±2	±4	±4	±4	±4	±4	±4	±4	±4	±4	±4	±4	±4	±4	+7
**PARTICIPANT RELATED INFORMATION AND ASSESSMENTS**																						
Informed Consent and Demography[d]	10.1.3	X																				
Tobacco Use	8.1	X																				
Childbearing Potential	8.3.8	X																				
Medical History/Concomitant Illness	8.3	X	X																			
Concomitant Medication	6.8	X	X	X	X	X	X	X	X	X	X	X	X	X	X	X	X	X	X	X	X	X
NYHA Classification	8.3.2	X	X	X		X	X	X	X		X			X							X	X
Pregnancy Test[e]	8.3.8, 10.4	X		X					X	X	X	X	X	X	X	X	X	X	X	X	X	X
Eligibility Criteria	5	X	X	X																		
Inclusion Criteria	5.1	X	X																			
Exclusion Criteria	5.2	X	X																			
Randomisation Criteria	5.5			X																		
Randomisation	6.3.1			X																		

Procedure	Protocol Section	Screening		Randomisation	Study intervention period																End of treatment	Follow-up
Visit		V1	V1A[a]	V2	V2A[b]	V2B[b]	V2C[b]	V2D[b]	V3	V4	V5	V6	V7	V8	V9	V10	V11	V12	V13	V14	V15	V16[c]
Timing of Visit (weeks)		up to -8 weeks	up to -2 weeks	0	24h	1	2	3	4	8	12	16	20	24	28	32	36	40	44	48	52	64
Visit Window (Days)				0	0	+2	±2	±2	±4	±4	±4	±4	±4	±4	±4	±4	±4	±4	±4	±4	±4	+7
Upload 99mTc PYP/DPD/HMDP cardiac scintigraphy including SPECT[f]	8.1	X																				
**SAFETY**																						
Vital Signs	8.3.4, Table 6-2	X	X	X	X	X	X	X	X	X	X	X	X	X	X	X	X	X	X	X	X	X
Physical Examination	8.3.1	X		X	X	X	X	X	X		X			X						X	X	
Body Measurements	8.3.3	X	X	X		X	X	X	X	X	X	X	X	X	X	X	X	X	X	X	X	X
Body weight	8.3.3	X	X	X		X	X	X	X	X	X	X	X	X	X	X	X	X	X	X	X	X
Height	8.3.3			X																		
ECG	8.3.5	X	X	X	X	X	X	X	X		X			X							X	X
Safety Monitoring[b]	8.3.6	X	X	X	X																	
24-hours Cardiac Monitoring In-Patient[b, h]	8.3.6			X																		
Cardiac Monitoring Out-Patient[b]	8.3.6	X[i]	X[i]		X[j, h]																	
Adverse Event	8.4			X	X	X	X	X	X	X	X	X	X	X	X	X	X	X	X	X	X	X
Laboratory Assessments	10.2	X	X	X	X	X	X	X	X	X	X			X							X	
Serology Reminder	10.2	X																				

**Procedure**	**Protocol Section**	**Screening**		**Randomisation**	**Study intervention period**																**End of treatment**	**Follow-up**
Visit		V1	V1A[a]	V2	V2A[b]	V2B[b]	V2C[b]	V2D[b]	V3	V4	V5	V6	V7	V8	V9	V10	V11	V12	V13	V14	V15	V16[c]
Timing of Visit (weeks)		up to -8 weeks	up to -2 weeks	0	24h	1	2	3	4	8	12	16	20	24	28	32	36	40	44	48	52	64
Visit Window (Days)				0	0	+2	±2	±2	±4	±4	±4	±4	±4	±4	±4	±4	±4	±4	±4	±4	±4	+7
Coagulation Parameter[k]	10.2	X	X	X	X	X		X	X	X	X			X							X	
Biochemistry	10.2	X	X	X	X	X	X	X	X	X	X			X							X	
Urinalysis[l]	10.2			X					X		X			X							X	
Haematology	10.2	X	X	X	X	X	X	X	X	X	X			X							X	
**OTHER ASSESSMENTS**																						
Clinical Outcome Assessments		X	X	X					X		X			X							X	
6-MWT	8.2.2.1	X	X	X					X		X			X							X	
Patient Global Impression of Status (PGI-S) for 6-MWT	8.2.2.3			X										X							X	
Patient Global Impression of Change (PGI-C) for 6-MWT	8.2.2.3													X							X	
KCCQ	8.2.2.3			X					X		X			X							X	
Patient Global Impression of Status (PGI-S) for KCCQ	8.2.2.3			X										X							X	
Patient Global Impression of Change (PGI-C) for KCCQ	8.2.2.3													X							X	
EQ-5D-5L	8.2.2.3			X					X		X			X							X	

Procedure	Protocol Section	Screening		Randomisation	Study intervention period																End of treatment	Follow-up
Visit		V1	V1A[a]	V2	V2A[b]	V2B[b]	V2C[b]	V2D[b]	V3	V4	V5	V6	V7	V8	V9	V10	V11	V12	V13	V14	V15	V16[c]
Timing of Visit (weeks)		up to -8 weeks	up to -2 weeks	0	24h	1	2	3	4	8	12	16	20	24	28	32	36	40	44	48	52	64
Visit Window (Days)				0	0	+2	±2	±2	±4	±4	±4	±4	±4	±4	±4	±4	±4	±4	±4	±4	±4	+7
NIS[m]	8.2.2.2			X										X							X	
**EFFICACY**																						
Cardiac MRI	8.2.3.1			X					X		X			X							X	
Echocardiography	8.2.3.2			X					X		X			X							X	
PK/PD	8.5			X	X	X	X	X	X	X	X	X	X	X	X	X	X	X	X	X	X	X
PK[n]	8.5.1			X	X	X	X	X	X	X	X	X	X	X	X	X	X	X	X	X	X	X
Misfolded transthyretin (misTTR)	8.7			X					X		X			X							X	
Anti-drug antibodies	8.8.1			X			X		X	X	X			X				X			X	X
Inflammatory Biomarkers	8.7			X	X	X	X	X	X		X			X							X	
Cardiac biomarkers	8.7	X	X	X	X	X	X	X	X		X			X							X	
**REMINDERS**																						
Hand Out ID Card		X																				
Pre-medication	6.1			X					X	X	X	X	X	X	X	X	X	X	X	X		
RTSM/IWRS	6.2	X		X					X	X	X	X	X	X	X	X	X	X	X	X	X	
Samples for future analysis[g]	8.8.3			X					X		X			X							X	

Procedure	Protocol Section	Screening		Randomisation	Study intervention period																End of treatment	Follow-up
Visit		V1	V1A[a]	V2	V2A[b]	V2B[b]	V2C[b]	V2D[b]	V3	V4	V5	V6	V7	V8	V9	V10	V11	V12	V13	V14	V15	V16[c]
Timing of Visit (weeks)		up to -8 weeks	up to -2 weeks	0	24h	1	2	3	4	8	12	16	20	24	28	32	36	40	44	48	52	64
Visit Window (Days)				0	0	+2	±2	±2	±4	±4	±4	±4	±4	±4	±4	±4	±4	±4	±4	±4	±4	+7
Biosamples for Future Analysis Taken	8.9			X					X		X			X							X	
Biosamples for Genetic Analysis Taken	8.9			X																	X	
Urine samples for future Analysis Taken	8.9			X																	X	
Hand Out Urine Kit	8.2.1.1	X		X						X			X							X		
**STUDY MATERIAL**																						
Administration of Trial Product	6.2			X					X	X	X	X	X	X	X	X	X	X	X	X		
Drug Dispensing	6.2			X					X	X	X	X	X	X	X	X	X	X	X	X		

a: Only applicable when the randomisation visit is more than 2 weeks after start of screening procedures.

b: Only applicable for participants in the sentinel cohort.

c: When participants discontinue study intervention prematurely, a follow-up visit should be carried out 16 weeks after last dosing. If this coincides with another scheduled visit, assessments pertaining to Visit 16 should be done in addition to the assessments pertaining to the other scheduled visit.

d: Demography consists of date of birth, age, sex, ethnicity, and race (according to local regulation). Race and ethnicity must be self-reported by the participant.

e: Only applicable for women of childbearing potential.

f: Only applicable if the ATTR CM diagnosis is based on non-invasive diagnostic criteria; transfer the most recent 99mTc PYP/DPD/HMDP cardiac scintigraphy including SPECT available for central review.

g: A separate consent is needed for the collection of these samples (optional).

h: Only applicable for the first 6 randomised Japanese participants.

i: 48-hour out-patient cardiac monitoring (before randomisation).

j: 6 days out-patient cardiac monitoring after discharge at V2A.

k: For participants on anticoagulants requiring laboratory monitoring the monitoring should take place as per local standards independent of study participation or similar.

l: Measured from two first morning void urine samples, 1 day before the visit and on the day of the visit.

m: Only applicable for participants with hATTR CM.

n: PK samples to be taken according to Table 1-1.

The PK sampling scheme is provided in Table 1-1.

Table 1-1 Sampling scheme for PK sampling

Visit	Week no. (visit window)	Dose	PK sample		Time relative to dose [a]	Time window allowance
V2	Week 0		X	Pre-dose sample	Before start of infusion	-30 min
		X				
			X	Post-dose sample	After end of infusion	+30 min
V2A[b]	Week 0 +24h		X	PK sample	24 hours	±3 h
V2B[b]	Week 1 (+2 days)		X	PK sample	7 days	
V2C[b]	Week 2 (± 2 days)		X	PK sample	14 days	
V2D[b]	Week 3 (± 2 days)		X	PK sample	21 days	
V3	Week 4		X	Pre-dose sample	Before start of infusion	-30 min
		X				
			X	Post-dose sample	After end of infusion	+30 min
V4	Week 8		X	Pre-dose sample	Before start of infusion	-30 min
		X				

Visit	Week no. (visit window)	Dose	PK sample		Time relative to dose [a]	Time window allowance
			X	Post-dose sample	After end of infusion	+30 min
V5	Week 12		X	Pre-dose sample	Before start of infusion	-30 min
		X				
			X	Post-dose sample	After end of infusion	+30 min
V6	Week 16		X	Pre-dose sample	Before start of infusion	-30 min
		X				
			X	Post-dose sample	After end of infusion	+30 min
V7	Week 20		X	Pre-dose sample	Before start of infusion	-30 min
		X				
			X	Post-dose sample	After end of infusion	+30 min
V8	Week 24		X	Pre-dose sample	Before start of infusion	-30 min
		X				
			X	Post-dose sample	After end of infusion	+30 min
V9	Week 28		X	Pre-dose sample	Before start of infusion	-30 min
		X				
			X	Post-dose sample	After end of infusion	+30 min
V10	Week 32		X	Pre-dose sample	Before start of infusion	-30 min
		X				
			X	Post-dose sample	After end of infusion	+30 min
V11	Week 36		X	Pre-dose sample	Before start of infusion	-30 min
		X				
			X	Post-dose sample	After end of infusion	+30 min
			X	Pre-dose sample	Before start of infusion	-30 min

Visit	Week no. (visit window)	Dose	PK sample		Time relative to dose [a]	Time window allowance
V12	Week 40	X				
			X	Post-dose sample	After end of infusion	+30 min
V13	Week 44		X	Pre-dose sample	Before start of infusion	-30 min
		X				
			X	Post-dose sample	After end of infusion	+30 min
V14	Week 48		X	Pre-dose sample	Before start of infusion	-30 min
		X				
			X	Post-dose sample	After end of infusion	+30 min
V15	Week 52		X	PK sample	4 weeks	±4 days
V16	Week 64		X	PK sample	16 weeks	+7 days

[a] From start of dosing for the pre-dose sampling and from end of dosing for the post-dose sampling; [b] Only applicable for participants in the sentinel cohort.

2 Introduction

2.1 Study rationale

Transthyretin amyloid cardiomyopathy (ATTR CM) is an increasingly recognised cause of heart failure in older adults worldwide, resulting from extracellular deposition of misfolded transthyretin protein (amyloid) in the myocardium.[1] ATTR CM is a progressive chronic disease with a high burden for patients and society, underscoring the need for therapies that reverse disease pathology and lower the risk of worsening of heart failure, hospitalisation and mortality in patients with ATTR CM.

ATTR CM can be hereditary (hATTR) due to rare genetic variants or occur sporadically as wild-type (wtATTR).[3] Patients with deposition of amyloid in the myocardium often present with symptoms and signs suggestive of chronic heart failure (i.e. dyspnea on exertion, peripheral edema, fatigue, elevated jugular venous pressure, hepatojugular reflux) and/or arrhythmias (i.e. palpitations, light-headedness, syncope).[4]

Due to advances in diagnostic strategies and the possibility of achieving non-invasive diagnosis, ATTR CM is a more frequent disease than previously thought.[5] Both the prevalence and incidence of ATTR CM has been reported to increase over the past decade.[6, 7] ATTR CM is a rare disease with an unknown global prevalence. Local studies, which were conducted in various regions with differing methods, suggest that at least 0.3–30 in 100,000 people live with ATTR CM.[6-10] Wild type ATTR CM is more frequently reported than hATTR CM[8-11], but data on the proportional distribution of hATTR CM vs wtATTR CM are not available. It is further estimated that up to 13.3% of patients with heart failure with preserved ejection fraction (HFpEF) may have ATTR CM, indicating that it is a more frequent cause of HFpEF than previously anticipated.[8, 9, 12] Once diagnosed, prognosis is particularly poor, dependent on sub-type median survival in untreated patients is approximately 2.5 (hATTR) to 5 years (wtATTR).[3]

Currently available treatment options are limited and include supportive treatment of cardiac involvement with medications (i.e. diuretics), implantable devices (i.e. pacemakers) or organ (heart and/or liver) transplantation.[13] More recently, a transthyretin tetramer stabiliser (tafamidis[14, 15]) for ATTR CM and silencers that reduce expression of mutant TTR (inotersen[16, 17] and patisiran[18, 19]) for hATTR polyneuropathy (PN) have been approved in the US and EU.

In a global randomised clinical study, known as the ATTR-ACT study,[20] the transthyretin tetramer stabiliser tafamidis was tested in patients with ATTR CM. Tafamidis was associated with a 30% reduction in all-cause mortality vs placebo (HR 0.70 $[0.51; 0.96]_{95\%CI}$), and a 32% reduction in cardiovascular related hospitalisations (RR 0.68 $[0.56; 0.81]_{95\%CI}$). The residual risk, however, still appears to be high, and especially so in more advanced disease (NYHA III at baseline). There was still disease progression in the tafamidis arm (decline in 6-minute walk test [6-MWT], increase in NT-proBNP and decreasing KCCQ-OS score), however significantly slower than in the placebo arm.[20] In addition, transthyretin tetramer stabilisers and silencers do not target the removal of amyloid already deposited in the tissues.

Hence, there is a major unmet medical need to improve the treatment of patients with ATTR CM. NNC6019-0001 (previously known as PRX004) is a humanised IgG1κ monoclonal antibody designed to specifically target a unique epitope, amino acid residues 89-97 (EHAEVVFTA) of TTR that is exposed only on monomeric, misfolded, and aggregated forms of TTR such as found in wtATTR and hATTR but hidden in the native tetramer conformation. NNC6019-0001 is administered every 4 weeks (Q4W) as an intravenous (i.v.) infusion.

Currently available non-clinical and clinical data indicate that NNC6019-0001 may deplete accumulated amyloid from the myocardium and consequently may reverse disease pathology and improve functional outcomes in patients with ATTR CM. In the FHD study (study NN6019-4965), NNC6019-0001 was associated with a mean change of –1.21% in global longitudinal strain (GLS) indicating a possible benefit in cardiac systolic function and NNC6019-0001 was safe and well tolerated supporting further development.[21] Taken together, the clinical findings suggest that NNC6019-0001's amyloid-depleting mechanism of action can result in clinical benefits and thus, NNC6019-0001 may provide a new treatment option for patients at high risk of early mortality due to amyloid deposition in the heart. The aims of this proof-of-principle study are to compare the effect of NNC6019-0001 versus placebo on functional endpoints, circulating- and imaging biomarkers as well as to evaluate pharmacokinetics, safety, and tolerability of the two dose levels covering the relevant therapeutic levels and based on the totality of data to select the dose to be studied in phase 3.

2.2 Background

Transthyretin amyloid cardiomyopathy (ATTR CM)

Transthyretin amyloid (ATTR) amyloidosis is a rare and progressive disease characterised by deposition of aggregates of misfolded transthyretin protein (amyloid). Transthyretin (TTR) is a naturally occurring protein, which may misfold to form toxic soluble monomers that subsequently may aggregate and form fibrils with resultant TTR amyloid deposition into tissues (e.g., heart, nerves, gastrointestinal tract) with disrupted organ structure and function as the consequence.[22] The TTR protein is produced primarily in the liver and in its normal tetrameric form serves as a carrier for thyroxin and vitamin A, the latter via the binding of retinol binding protein.

ATTR amyloidosis can be hereditary (hATTR) due to rare genetic variants or occur sporadically as wild type (wtATTR).[3]

In hATTR amyloidosis, the body makes a mutant form of the TTR protein. There are more than 100 reported types of TTR mutations that promote amyloid fibril formation. The predominant organ involvement for hATTR amyloidosis is either the nervous system (hATTR PN amyloidosis) or the heart (hATTR CM amyloidosis), although other organ systems are also often involved.[3, 23, 24] Depending on the specific mutation, some patients predominantly have cardiac symptoms, some predominantly have symptoms from the nervous system, and some have a combination of both. In one study of approximately 500 patients with the hATTR V30M mutation, one third of patients had clinical nephropathy based on elevated levels of proteinuria, and 10% progressed to end-stage renal disease.[25, 26] A significant number of TTR mutations associated with a clinical phenotype cause a restrictive cardiomyopathy.[24]

Wild-type ATTR is similar to hATTR except that the protein that is deposited is the misfolded, non-mutated transthyretin protein. The misfolding is thought to be caused by age-related impaired proteostasis.[27] The predominant effect of wtATTR amyloidosis is on the heart.

2.3 Benefit-risk assessment

The main benefits and risks related to participation in the study are described in the below sections. More detailed information about the known and expected benefits and risks of NNC6019-0001 may be found in the current edition of the investigator's brochure[28] or updates thereof.

2.3.1 Risk assessment

The risk assessment is presented in Table 2-1.

Table 2-1 Risk assessment

Potential risk of clinical significance	Summary of data/rationale for risk	Mitigation strategy
**Study intervention: NNNC6019-0001**		
**Potential risk**: Hypersensitivity	As expected for a protein-based drug, participants treated with NNC6019-0001 may develop localised (to the infusion site) or generalised hypersensitivity reactions.	To mitigate risk of hypersensitivity reactions, participants will be treated with histamine (H1) blockers and acetaminophen (or paracetamol) prior to receiving NNC6019-0001.<br>As a precaution, participants with known or suspected hypersensitivity to NNC6019-0001 or related products are excluded. Participants and investigators will be instructed to detect signs and symptoms of hypersensitivity reactions. In addition, participants will be instructed to contact the site staff as soon as possible for further guidance if suspicion of a hypersensitivity reaction to the NNC6019-0001 occurs.
**Potential risk**: Myocardial Inflammation	The intended action of the NNC6019-0001 in clearing amyloid through macrophage activation and phagocytosis may result in a theoretical risk of myocardial inflammation.	Participants will be followed closely and carefully by qualified medical staff.<br>To minimise the risk, standard safety surveillance activities and medical monitoring will be performed by Novo Nordisk.
**Potential risk**: Pro-arrhythmic risk	Based on the potential mechanism of action of NNC6019-0001 and the underlying disease pathophysiology of ATTR cardiomyopathy, there is a theoretical risk of arrhythmogenicity.	Participants will be followed closely and carefully by qualified medical staff.<br>To minimise the risk, standard safety surveillance activities and medical monitoring will be performed by Novo Nordisk.
**Study procedures**		
**COVID-19:** Risk of COVID-19 infection in relation to participation in the study	Participants may be exposed to the risk of COVID-19 transmission and infection in relation to site visits if an outbreak is ongoing in the given country.	The risk of COVID-19 transmission in relation to site visits is overall considered to be low, however this may vary over time and between geographical areas.<br>Where relevant, to minimise the risk as much as possible, the following measures will be taken:<br>• Cautious participant recruitment planning to ensure controlled participant enrolment in countries where the COVID-19 pandemic is evaluated to be sufficiently under control, and at sites where health care resources are evaluated to be adequate.

Potential risk of clinical significance	Summary of data/rationale for risk	Mitigation strategy
		• On-site visits are planned to be as short as possible. Physical contact between participants and site staff is limited to the extent possible, and protective measures are implemented (e.g., use of masks, sanitisers, no aerosol-generating procedures etc.) according to local practice.
**Cardiac MRI**	All medical imaging examinations are carried out using a scanner where the participant lies on a bed that is moved into the scanner so that the head is inside the scanner tunnel. This procedure may be perceived as unpleasant for people suffering from claustrophobia. Further, the MRI examination involves the use of strong magnetic field but does not involve radiation exposure.	To minimise the risk of claustrophobia and risks associated with the magnetic field, local guidelines will be followed (such as excluding participants with certain implanted devices or other incompatible metallic objects in their body to having a cardiac MRI performed).<br>A gadolinium contrast agent will be applied intravenously during the cardiac MRI and it normally does not cause side effects. To minimize the rare risk of adverse reactions to gadolinium, people with advanced kidney disease (such as eGFR<30 ml/min/1.73m2) according to local guidelines will not undergo cardiac MRI.
**Other**		
**Pregnancy and fertility**	Studies in animals have not shown reproductive toxicity. There are limited data from the use of NNC6019-0001 in pregnant women.	NNC6019-0001 should not be used during pregnancy. Women of childbearing potential are required to use highly effective contraceptive methods when participating in this study (Appendix 4 [Section 10.4]) If a female participant wishes to become pregnant, or pregnancy occurs during the study, treatment with study intervention should be discontinued immediately (please refer to Section 7.1 for further guidance). The effect of NNC6019-0001 on fertility in humans is unknown.

2.3.2 Benefit assessment

Preliminary efficacy results from the FHD study (study NN6019-4965) for 7 evaluable participants treated with NNC6019-0001 were favourable (please see the current IB[28] for more details). NNC6019-0001 was associated with a mean change of – 1.21% in GLS from baseline to 9 months indicating a possible benefit in cardiac systolic function. Six (6) of the 7 efficacy evaluable participants had no change in their baseline New York Heart Association (NYHA) class at month 9. NNC6019-0001 demonstrated a mean change of 1.29 in Neuropathy Impairment Score (NIS) from baseline to 9 months indicating a stable peripheral nerve function. Participation in this study is contributing to the process of developing a new therapy option for patients with ATTR CM with a proposed amyloid-depleting mode of action. Expected benefits associated with treatment with NNC6019-0001 include those associated with removal of amyloid in tissues, specifically in the myocardium.

It is expected that all participants will benefit from participation through frequent and close contact with investigators and other site staff who will ensure that the participants are treated to recommended standard of care for their conditions, including ATTR CM, and disease development and progression will be closely monitored and treated.

2.3.3 Overall benefit-risk conclusion

Taking into account the measures taken to minimise risk and burden to participants participating in this study, the potential risks identified in association with NNC6019-0001 are justified by the anticipated benefits that may be afforded to participants with ATTR CM.

3 Objectives, endpoints and estimands

The objectives and endpoints are listed in Table 3-1.

Table 3-1 Objectives and endpoints

**Objectives**	**Endpoints**		
**Primary**	**Title**	**Time frame**	**Unit**
• To compare the effect of two dose levels of NNC6019-0001 (30 mg/kg and 100 mg/kg) versus placebo on:<br>• change in 6-minute walk test and<br>• change in NT-proBNP<br>from baseline to week 52 in participants with hATTR or wtATTR cardiomyopathy.	*Primary*		
	Change in 6-minute walk test (6-MWT)	From baseline (week 0) to visit 15 (week 52)	Meters
	Change in NT-proBNP	From baseline (week 0) to visit 15 (week 52)	Percentage
**Secondary**	**Title**	**Time frame**	**Unit**
• To compare the effect of two dose levels of NNC6019-0001 (30 mg/kg and 100 mg/kg) versus placebo on:<br>• biomarkers<br>• pharmacodynamic endpoints<br>from baseline to week 52 in participants with hATTR or wtATTR cardiomyopathy.	*Supportive*		
	Change in myocardial extracellular volume (ECV)	From baseline (week 0) to visit 15 (week 52)	%-points
	Change in Kansas City Cardiomyopathy Questionnaire (KCCQ) Clinical Summary Score[a] (CSS)[2]	From baseline (week 0) to visit 15 (week 52)	Score
	Change in neuropathy impairment score[b] (NIS)	From baseline (week 0) to visit 15 (week 52)	Score
	Change in troponin I	From baseline (week 0) to visit 15 (week 52)	ng/mL
	Change in global longitudinal strain (GLS) on echocardiography	From baseline (week 0) to visit 15 (week 52)	%-points
• To compare the effect of two dose levels of NNC6019-0001 (30 mg/kg and 100 mg/kg) versus placebo on:<br>• safety and tolerability<br>from baseline to week 64 in participants with hATTR or wtATTR cardiomyopathy.	Number of treatment emergent adverse events	From baseline (week 0) to visit 16 (week 64)	Count
	Time to occurrence of all-cause mortality	From baseline (week 0) to visit 16 (week 64)	Weeks
	Number of CV events comprising hospitalisation due to CV events or urgent heart failure visits	From baseline (week 0) to visit 16 (week 64)	Count
**Exploratory**	**Title**	**Time frame**	**Unit**
	Exploratory		
• To compare the effect of two dose levels of NNC6019-0001 (30 mg/kg and 100 mg/kg) versus placebo on depletion of plasma misTTR from baseline to week 52 in participants with hATTR or wtATTR cardiomyopathy.	Change in misfolded transthyretin[c] (misTTR)	From baseline (week 0) to visit 15 (week 52)	Percentage

Objectives	Endpoints		
• To compare the effect of two dose levels of NNC6019-0001 (30 mg/kg and 100 mg/kg) versus placebo on change in EQ-5D-5L from baseline to week 52 in participants with hATTR or wtATTR cardiomyopathy.	Change in EQ-5D-5L	From baseline (week 0) to visit 15 (week 52)	Score

[a] Clinical Summary Score (CSS) consists of the Symptom domain and the Physical Limitation domain, additional analyses of the remaining domains will be described in the statistical analysis plan (SAP). Scores range from 0 to 100 and lower scores represent more severe symptoms and/or limitations and scores of 100 indicate no symptoms, no limitations, and excellent quality of life; [b] Only applicable for participants with hATTR CM. The total NIS score is graded on a scale of 0–244, with a higher score indicating greater impairment; [c] Including fragmented TTR depending on assay development.

Abbreviations: CV = cardiovascular; hATTR = hereditary ATTR; misTTR = misfolded transthyretin; NT-proBNP = N-terminal-pro brain natriuretic peptide; wtATTR = wild-type ATTR.

Primary estimand

The primary estimand addresses the following question of interest: What is the effect of two dose levels of NNC6019-0001 (30 mg/kg and 100 mg/kg) versus placebo on change in 6-MWT and NT-proBNP from baseline to week 52, or occurrence of death or CV hospitalisation, in participants with hATTR or wtATTR cardiomyopathy, regardless of premature discontinuation of study intervention.

The primary estimand is defined with the five attributes as defined in ICH E9(R1) addendum[29]:

- Treatment condition: The treatment regimen evaluated is i.v. infusion of NNC6019-0001 Q4W at two dose levels (30 mg/kg and 100 mg/kg) vs placebo, irrespective of use of concomitant medications indicated for ATTR CM or ATTR PN (TTR silencers and stabilisers).
- Population: The treatment effect is assessed for the target population of patients with hATTR or wtATTR CM.
- Variable: The treatment effect is assessed by change in 6-MWT and relative change in NT-proBNP from baseline to week 52.
- Remaining intercurrent events (see Table 3-2):
  Premature discontinuation of randomised study intervention will be handled by a treatment policy strategy including all post-discontinuation observations of 6-MWT and NT-proBNP in the analysis and conditional multiple imputation from the placebo arm of missing values.
  - Missing values will be handled as follows:
    - Missing 6-MWT values due to all-cause death or CV hospitalisation or urgent heart failure visit will be handled by a composite strategy assigning a single value of 0 meters
    - The risk of missing 6-MWT due to fractures or fall-related injuries is assumed unrelated to treatment and missing values will be handled by a hypothetical strategy depending on whether the participant is on the randomised study intervention or has prematurely discontinued.
    - Missing 6-MWT values for other than the above reasons will be handled by a hypothetical strategy depending on whether the participant is on the randomised study intervention or has prematurely discontinued.
    - Missing NT-proBNP values due to all-cause death or CV hospitalisation or urgent heart failure visit will be handled by a composite strategy assigning a single value

corresponding to the highest observed NT-proBNP value across all participant and visits (including baseline).
    - Missing NT-proBNP values for other reasons will be handled by a hypothetical strategy depending on whether the participant is on the randomised study intervention or has prematurely discontinued.
- Population-level summary:
    - Difference in mean change in 6-MWT from baseline between NNC6019-0001 (30 mg/kg or 100 mg/kg) vs placebo.
    - Difference in mean change in NT-proBNP (log-scale) from baseline between NNC6019-0001 (30 mg/kg or 100 mg/kg) vs placebo. The difference will be back transformed to original scale and reported as a ratio of geometric mean ratios.

Table 3-2 Handling of premature discontinuation of randomised study intervention and missing values for various reasons

		**Premature discontinuation of randomised study intervention**	
**Value**	Endpoint	No	Yes
**Observed**	6-MWT<br>NT-proBNP	Use value	Use value
**Missing due to death or CV hospitalisation or urgent heart failure visit**	6-MWT<br>NT-proBNP	Assign 0 meters<br>Assign highest observed	Assign 0 meters<br>Assign highest observed
**Missing due to fracture or other reason**	6-MWT<br>NT-proBNP	Impute from own group	Impute from placebo group

Abbreviations: 6-MWT = 6-minute walk test; NT-proBNP = N-terminal-pro brain natriuretic peptide.

Rationale for the estimand

The estimand for the two primary endpoints is chosen to best quantify a potential effect on the planned phase 3 primary endpoint considering both efficacy and tolerability. To penalise the phase 3 primary endpoint events as much as possible within sensible limits, it is chosen to assign a single worst-case value of 0 meters for 6-MWT and the highest observed value of NT-proBNP, acknowledging that such values may not be directly interpretable.

4 Study design

4.1 Overall design

This is an interventional, randomised, multinational, multicentre, three-arm parallel-group, double-blind, placebo-controlled study comparing i.v. NNC6019-0001 Q4W at two dose levels (30 mg/kg and 100 mg/kg) versus placebo in participants with hATTR or wtATTR CM.

Approximately 99 participants will be randomised 1:1:1 to receive i.v. 30 mg/kg NNC6019-0001, 100 mg/kg NNC6019-0001 or placebo Q4W added to standard of care. Randomisation will be stratified by disease type (wtATTR vs hATTR) and maximum 80% of participants randomised will be participants with wtATTR.

The study consists of a screening period of up to 8 weeks, followed by a 52-week intervention period. For participants with a screening period lasting more than 2 weeks, a pre-randomisation visit should be carried out maximum 2 weeks prior to the randomisation visit to verify safety related eligibility criteria that tend to fluctuate over time. When participants discontinue study intervention according to protocol, an end of treatment visit should be carried out 4 weeks after administration of the last dose and a follow-up visit should be carried out 16 weeks after administration of the last dose. For participants who discontinue study intervention prematurely, please refer to Section 7.1. The planned study duration for the individual participant will be approximately 64 weeks (excluding screening). The study design is illustrated in Figure 4-1.

Sentinel dosing and intensified safety monitoring will be carried out for minimum 9 participants (3 participants per intervention group), see details on sentinel dosing in Section 6.1. Participants in the sentinel cohort will continue to be dosed Q4W and be part of the main cohort. Stratification by disease type will not be applied for participants in the sentinel cohort.

Figure 4-1 Study design

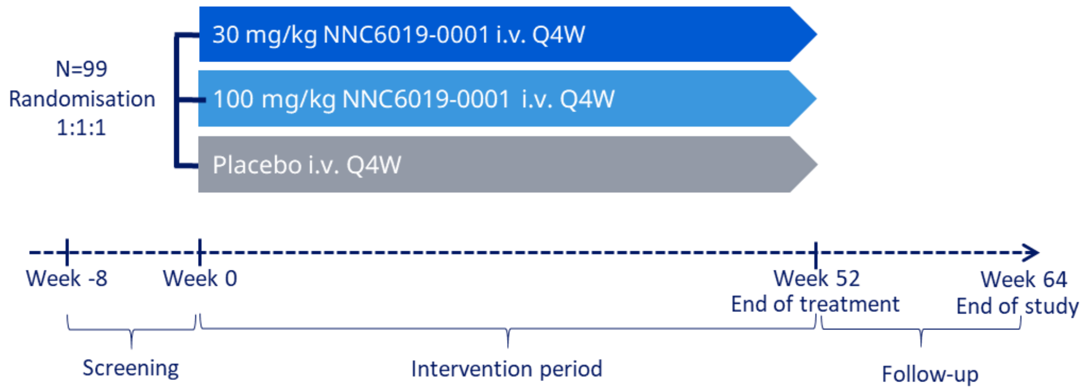

Abbreviations: i.v. = intravenous; N = number of randomised participants; Q4W = every 4 weeks.

4.2 Scientific rationale for study design

The study is designed as a 3-armed study (30 mg/kg NNC6019-0001, 100 mg/kg NNC6019-0001 and placebo) in accordance with the study objectives and to minimise bias. Randomised and double-blinded intervention with NNC6019-0001 or placebo offers a robust method for assessment of the effects of NNC6019-0001.

A placebo-controlled design is chosen to ensure scientific rigour of the study. The placebo control will facilitate evaluation of efficacy as well as safety and tolerability of NNC6019-0001 by allowing adverse events associated with NNC6019-0001 to be distinguished from symptoms of the underlying disease. Established standard of care will be allowed throughout the study, therefore, assignment to placebo will not place participants at increased risk compared to patients not participating in the study.

The present study will include a population of patients with established ATTR CM. To ensure representative inclusion in all intervention groups, randomisation will be stratified by disease type (wtATTR vs hATTR). Randomisation will not be stratified by disease type for participants in the sentinel cohort.

To safeguard the most vulnerable populations, the age limit is set to <85 years at screening. The participants should be classified as NYHA class at II-III and the participants should be able to complete ≥150 meters to ≤450 meters on the 6-MWT at screening. The study population is chosen to optimise the likelihood of achieving a clinical benefit of the treatment and it will be ensured that participants with both wtATTR and hATTR are included by randomising a maximum of 80% participants with wtATTR. Cardiac involvement will be ensured with left ventricular wall thickness of ≥12 mm[5] and with NT-proBNP minimum values (NT-proBNP ≥650 in sinus rhythm and >1000 pg/mL in atrial fibrillation). Participants are required to have an eGFR ≥25 mL/min/1.73m^2 at screening to minimise the risk of development of nephrogenic systemic fibrosis (NSF)[30] related to MRI imaging procedure for the majority of the included population while still allowing entry for participants with more advanced disease. Participants should be on stable doses of cardiovascular medical therapy for at least 6 weeks prior to the randomisation visit to reduce possible confounding bias.

The study is a non-confirmatory study with 2 primary endpoints; change in 6-MWT and NT-proBNP from baseline to week 52. NT-proBNP is an important diagnostic and prognostic biomarker used to evaluate cardiac severity in ATTR CM patients and increased levels indicate more severe cardiac involvement and poorer prognosis.[31] The 6-MWT is a standardised field test to evaluate functional exercise performance, and both NT-proBNP and 6-MWT have important prognostic value for death and CV hospitalisation in patients with chronic heart failure[32] and ATTR CM[31, 33]. To support the primary endpoints, a supportive secondary endpoint, change in ECV will be of relevance as it is a robust marker of cardiac interstitium infiltration and extracellular volume and remains an independent predictor of prognosis in ATTR CM after adjusting for known predictors and is the earliest disease marker to track amyloid regression.[34] Additional context will be provided by other supportive secondary endpoints including safety and tolerability, functional endpoints (KCCQ and change in NIS), all-cause mortality and cardiovascular events (CV hospitalisations and urgent heart failure visits), complemented with troponin I and an additional imaging endpoint (global longitudinal strain on echocardiography).

An intervention period of 52 weeks will provide robust data to evaluate the full effect of NNC6019-0001. An interim evaluation is planned at week 24 to possibly enable early selection of the dose to be investigated in phase 3 (see Section 9.4).

In the FHD study (study NN6019-4965), i.v. infusions of NNC6019-0001 administered Q4W were generally safe and well-tolerated at all dose levels tested (doses of 0.1, 0.3, 1.0, 3.0, 10 and

30 mg/kg). No treatment related serious TEAEs, life-threatening events, deaths, or dose limiting toxicities were reported. Due to the introduction of the 100 mg/kg dose in the present study (see Section 4.3) and the serious nature of the disease, a sentinel cohort is applied for participant safety. The sentinel cohort allows for assessments of acute safety of minimum 3 participants per intervention group to ensure the safety of participants before randomising the remaining participants into the main cohort.

4.3 Justification for dose

Doses of 30 and 100 mg/kg NNC6019-0001 are selected for this study. Study intervention will be infused intravenously Q4W. The $t_{½}$ of NNC6019-0001 is approximately 1 month, supporting Q4W dosing.

The doses for this study were selected based on the reduction in circulating levels of misTTR from previous clinical data with NNC6019-0001 (previously known as PRX004). NNC6019-0001 is expected to bind to and reduce circulating levels of misTTR, as well as to bind and deplete accumulated amyloid in the myocardium (see Section 2.1).

In the FHD study (study NN6019-4965), i.v. infusions of NNC6019-0001 Q4W at dose levels of 0.1, 0.3, 1.0, 3.0, 10 and 30 mg/kg were tested in patients with hATTR and circulating misTTR levels were measured (n=3 for each dose up to 10 mg/kg, and n=6 for the 30 mg/kg dose). There was no clear effect of the lower doses (0.1, 0.3, 1.0 and 3.0 mg/kg) on the relative reduction in circulating levels of misTTR during month 3 compared to baseline.[35] However, an effect was observed for the 10 and 30 mg/kg doses, with a greater reduction in misTTR in participants treated with 30 mg/kg compared to 10 mg/kg. The 30 mg/kg dose level was selected for further study. There was no indication that the maximum level of misTTR reduction with NNC6019-0001 had been reached, and doses above 30 mg/kg may have a larger effect. No dose-limiting toxicities were observed in the FHD study, and there was no apparent relationship between dose and AEs. The dose with the optimal benefit/risk may not have been reached in the FHD study, and an additional, higher dose of 100 mg/kg was therefore selected for investigation in this study accommodating pre-clinical exposure limits. The selected dose is below what was defined as no observed adverse-effect level (NOAEL) which was 300 mg/kg. Modelling and simulation based on FHD data support that participants treated with 100 mg/kg can expect a near maximal reduction in circulating misTTR levels across the dosing interval when at steady-state of NNC6019-0001.

4.4 End of study definition

The end of the study is defined as the date of the last visit of the last participant in the study globally.

A participant is considered to have completed the study if he/she has completed all periods of the study including the last visit. If a randomised participant has died during study, ‘date of study completion’ is the date of death.

The primary endpoints are evaluated at visit 15 (week 52). The primary completion date (PCD) is defined as the date of visit 15 (week 52) on which the last participant in the clinical study has an assessment for the primary endpoints. If the last participant is withdrawn early, the PCD is considered the date when the last participant would have completed visit 15.

5 Study population

Prospective approval of protocol deviations to recruitment and enrolment criteria, also known as protocol waivers or exemptions, is not permitted.

Pre-screening is defined as review of the patient medical records, including handing out participant information, as well as database review. Any pre-screening activities must be documented on site by the investigator.

All eligibility criteria related to laboratory assessments should be based on central laboratory data, unless otherwise stated.

5.1 Inclusion criteria

Participants are eligible to be included in the study only if all the following criteria apply:

1. Informed consent obtained before any study-related activities. Study-related activities are any procedures that are carried out as part of the study, including activities to determine suitability for the study.
2. Male or female.
3. Age ≥ 18 to < 85 years at the time of signing informed consent.
4. Have an established diagnosis of ATTR CM with either wild-type TTR or hereditary TTR genotype as per local standards[a].[5]
5. Expected to be on stable doses of cardiovascular medical therapy 6 weeks prior to the randomisation visit.
6. Known[b] end-diastolic interventricular septal wall thickness ≥12 mm.
7. Presently classified as New York Heart Association (NYHA) Class II-III.
8. NT-proBNP concentration ≥650 pg/mL in sinus cardiac rhythm and >1000 pg/mL in atrial fibrillation at screening.
9. Completed ≥150 meters to ≤450 meters on the 6-MWT at screening.
10. Absolute neutrophil count $\geq 2.0 \times 10^9$/L; platelet count $\geq 120 \times 10^9$/L at screening.
11. Aspartate transaminase (AST) or alanine transaminase (ALT) levels ≤ 2.5 × the upper limit of normal (ULN) or total bilirubin ≤2 × ULN at screening.
12. Estimated glomerular filtration rate (eGFR) ≥25 mL/min/1.73 m^2 at screening.

5.2 Exclusion criteria

Participants are excluded from the study if any of the following criteria apply:

1. Known or suspected hypersensitivity to study intervention(s) or related products.
2. Previous dosing in this study
3. Female who is pregnant, breast-feeding or intends to become pregnant or is of childbearing potential and not using highly effective contraceptive method, as defined in Appendix 4. UK, Spain: For country-specific requirements, please refer to Appendix 8 (Section 10.8).
4. Use of another approved or non-approved investigational medicinal product within 30 days or 5 half-lives of the investigational medicinal product (whichever is longer) before screening.
5. Any disorder, which in the investigator's opinion might jeopardise participant's safety or compliance with the protocol.
6. Current diagnosis or history of amyloid light chain or other non-ATTR amyloidosis

7. Cardiomyopathy not primarily caused by ATTR CM, for example, cardiomyopathy due to hypertension, valvular heart disease, or ischemic heart disease.
8. A prior solid organ transplant.
9. Planned solid organ transplant during the study.
10. Presence or history of malignant neoplasm (other than basal or squamous cell skin cancer, in-situ carcinomas of the cervix, or in-situ/high grade prostatic intraepithelial neoplasia (PIN) or low-grade prostate cancer) within 5 years before screening.
11. Current treatment with calcium channel blockers with conduction system effects (e.g., verapamil, diltiazem). The use of dihydropyridine calcium channel blockers is allowed. The use of digoxin will only be allowed if required for management of atrial fibrillation with rapid ventricular response.
12. Acute coronary syndrome, unstable angina, stroke, transient ischemic attack (TIA), coronary revascularisation, cardiac valve repair, or major surgery within 3 months of screening.
13. Body weight > 120 kg (264.6 lb) at screening.
14. Evidence of current or chronic hepatitis C virus[c] or hepatitis B virus infection.
15. History of or known seropositivity for human immunodeficiency virus (HIV).
16. International normalised ratio (INR)>1.5 (unless participant is on anticoagulant therapy, in which case excluded if INR>3.5).
17. History of contrast allergy or adverse reactions to gadolinium-containing agents.

Definitions:

[a] Non-invasive diagnostic pathway will be confirmed by a centralised expert review, see Section 8.1; [b] Medical history/records are accepted; [c] As per current medical standards i.e. hepatitis C virus RNA testing needed only if the anti-hepatitis C virus screening is positive.

5.3 Lifestyle considerations

5.3.1 Activity

For participants, especially in the sentinel cohort, the quantity or types of physical activity may be limited during the cardiac monitoring periods (holter ECG) pre- and post-dosing, as per local requirements (with regards to potential electrode displacement and water resistance of the device used). Dosing will take place at the site as an infusion and physical activity may be limited during the infusion and subsequent observation time (Section 6.1).

5.4 Screen failures

A screen failure occurs when a participant who consents to participate in the clinical study is not subsequently eligible for participation according to the inclusion/exclusion and randomisation criteria. Visit 1A is not allowed if an eligibility criterion is failed at Visit 1. A screen failure must be registered in the system (Randomisation and Trial Supplies Management System [RTSM] / Interactive Web Response System [IWRS]).

If participants withdraw their consent prior to randomisation or do not return for randomisation, a screen failure must be registered in the RTSM/IWRS. The reason for failure will in all cases be captured in the electronic case report forms (eCRF).

A minimal set of screen failure information is required to ensure transparent reporting of screen failure participants to meet requirements from regulatory authorities. Minimal information includes informed consent date, demography, screen failure details, and eligibility criteria.

Individuals who do not meet the criteria for participation in this study may be rescreened if the investigator assesses it is reasonable to expect that potential changeable or fluctuating in- or exclusion criteria may change, e.g., biochemical parameters. However, previously dosed participants cannot be rescreened.

If the participant has failed one of the inclusion criteria or fulfilled one of the exclusion criteria related to laboratory parameters, re-sampling is not allowed, unless participant is re-screened. In case of technical issues with laboratory sample(s) collected for eligibility assessment (e.g., haemolysed or lost) re-sampling is allowed for the affected laboratory parameter(s). This is not considered rescreening.

Individuals who are rescreened are required to sign a new informed consent form and provided with a new subject ID. A new screening must be registered in the RTSM/IWRS.

5.5 Randomisation criteria

For all participants: To be randomised, the following randomisation criterion must be answered "yes":

- On stable doses of cardiovascular medical therapy within the last 6 weeks prior to randomisation.

Sentinel cohort

For participants in the sentinel cohort, the following randomisation criteria must be answered "no":

1. Sinus pauses >3 seconds in the day or sinus pauses >5 seconds at night during the 48 hours outpatient cardiac monitoring prior to the randomisation visit.
2. Arrhythmia requiring treatment diagnosed during the 48 hours out-patient cardiac monitoring prior to the randomisation visit.

6 Study interventions and concomitant therapy

Study intervention is defined as any investigational intervention(s), marketed product(s), placebo, or medical device(s) intended to be administered to a study participant according to the study protocol.

Trial product comprise investigational medicinal products (IMPs), including placebo and comparators, non-investigational medicinal products (NIMPs) and/or investigational medical devices.
In this protocol:

- Trial products consist of IMPs (NNC6019-0001 and placebo), but NIMPs are not considered trial products.
- In situations where trial product is referring only to vials with NNC6019-0001, e.g., when discussing trial product accountability, this is specified.

6.1 Study interventions administered

Table 6-1 provides an overview of the study interventions in the study.

Table 6-1 Study interventions

**Study intervention name**	**30 mg/kg NNC6019-0001**	**100 mg/kg NNC6019-0001**	**Placebo**	**Other interventions**	
Intervention name	NNC6019-0001		Placebo (saline[a])	Pre-medication	Post-dose flushing (saline[a])
Intervention type	IMP, test product		IMP, reference therapy	NIMP, pre-medication	NIMP, post-dose flushing
Pharmaceutical form	Lyophilised powder for solution for infusion<br>Reconstituted with SWFI[b] and diluted with normal saline[a]		Sterile solution for infusion	Tablet or capsule	Sterile solution
Route of administration	Intravenous			Oral	Intravenous
Trial product strength	250 mg NNC6019-0001 supplied as a sterile, lyophilised dosage form in 20 mL vials		0.9% sodium chloride solution	25 mg diphenhydramine (or an equivalent dose of an H1 antihistamine) and 650 mg acetaminophen (or an equivalent paracetamol dose)	0.9% sodium chloride solution
Dose and dose frequency	Every 4 weeks (Q4W)			30 to 90 minutes prior to start of infusion with NNC6019-0001 or placebo	Post-dose flushing
Dosing instructions and administration	See description of Dosing instructions/administration below			As per site standard	As per site standard
Sourcing	Lyophilised powder is supplied by Novo Nordisk A/S		Provided locally by study site	Provided locally by study site	
Packaging and labelling	Lyophilised powder is labelled and packaged by Novo Nordisk A/S. Labelled in accordance with EU GMP, Volume 4, Annex 13,[36] local regulations and study requirements		250 mL infusion bags for diluent containers as per standards defined in the TMM	No protocol specific requirements	

[a] Normal Saline: USP, Ph.Eur., BP or as per local pharmacopeia specification is provided locally by study site.

[b] SFWI = Sterile water for injection USP, Ph. Eur, BP or local pharmacopeia specification. Sterile water for injection is provided locally by study site in glass or plastic containers for single use as per site standards for reconstitution of products. Preparation of doses will be described in the Trial Master Manual (TMM).

Investigational medicinal products (IMP)

The IMPs are listed in Table 6-1.

Dosing instructions/administration

Trial product should only be administered in settings where emergency resuscitative equipment and personnel trained in the management of anaphylaxis are immediately available to treat systemic reactions under the direct supervision of a physician. Trial product will be administered Q4W (±4-day window allowed starting with second dose) as a 2 or 3-hour i.v. infusion. A minimum of 20 days is required between infusions. Each participant's initial dose will be delivered over 3 hours (±10 minutes), followed by a ~30 mL saline flush (Table 6-2). If the first dose is tolerated without a hypersensitivity-associated AE, subsequent infusions may be delivered over 2 hours (±10 minutes), followed by a ~30 mL saline flush. At each dosing visit, participants will be premedicated with 25 mg diphenhydramine (or an equivalent dose of an H1 antihistamine) and 650 mg acetaminophen (or an equivalent paracetamol dose) 30 to 90 minutes prior to start of infusion with trial product.

Table 6-2 Timing of infusion and measurement of vital signs

	Infusion time	Observation time	Measurement of vital signs during observation time
**Sentinel cohort**[a]			
1st dose	3 hours<br>(±10 minutes)	24 h (± 4h) in-house/admitted[b]	Before infusion<br>Halfway through infusion<br>At end of infusion<br>After infusion: 10 min (±1 min), 30 min (±10 min), 60 min (±10 min), 2 hours (±15 min) , 3 hours (±15 min), 4 hours (±20 min), 8 hours (±20 min), and 24 hours (±4 hours)
2nd dose and on	2 hours<br>(±10 minutes)	90 minutes<br>(±10min)	Before infusion<br>Halfway through infusion<br>At end of infusion<br>After infusion: 10 min (±1 min), 90 min (±10 min)
**Main cohort**			
1st dose	3 hours<br>(±10 minutes)	4 hours (±20min)	Before infusion<br>Halfway through infusion<br>At end of infusion<br>After infusion: 10 min (±1 min), 2 hours (±15 min), and 4 hours (±20 min)
2nd dose and on	2 hours<br>(±10 minutes)	90 minutes<br>(±10min)	Before infusion<br>Halfway through infusion<br>At end of infusion<br>After infusion: 10 min (±1 min), 90 min (±10 min)

[a]Also applicable for the first 6 randomised Japanese participants; Japan: For country specific requirements, please refer to Appendix 8 (Section 10.8). [b] Discharge participant from clinic if no immediate safety concerns and/or hypersensitivities are present after the post-dose assessments and 24-hour cardiac monitoring period. In the event of any clinical concerns or suspicious signs or symptoms after the infusion, the participant will remain with the Investigator and study staff for further observation until the Investigator deems the participant can safely leave the clinic.

Sentinel cohort

Due to the introduction of the 100 mg/kg dose (see Section 4.3) and the serious nature of the disease, a sentinel cohort is applied for participant safety.

The sentinel cohort, consisting of minimum 9 participants, will be subjected to intensified cardiac monitoring during the first 28 days of the study. Participants in the sentinel cohort will undergo cardiac monitoring on an out-patient basis for at least 48 hours within 2 weeks prior to the randomisation visit to ensure that the eligibility criteria for participation in the study are met.

After randomisation, 1 participant in each of the intervention groups will receive the first dose of study intervention (1 participant will receive 30 mg/kg NNC6019-0001, 1 participant will receive 100 mg/kg NNC6019-0001, and 1 participant will receive placebo) on an in-patient basis and will be observed under continuous cardiac monitoring for at least 24 hours after start of infusion. On discharge, participants will be monitored using continuous cardiac monitoring on an out-patient basis until 7 days after start of infusion.

Dosing of the remaining 6 participants in the sentinel cohort will be initiated following safety evaluation of blinded relevant safety data including AEs, vital signs, ECGs and cardiac monitoring evaluated by investigator, and laboratory parameters for the initial 3 participants. The evaluation of blinded safety data and decision to continue dosing in the sentinel cohort will be performed by an internal Novo Nordisk medical monitoring group at a dedicated meeting after completing 7 days of post-dose cardiac monitoring for the initial 3 participants. The sentinel cohort design is illustrated in Figure 6-1.

Figure 6-1 Sentinel cohort design

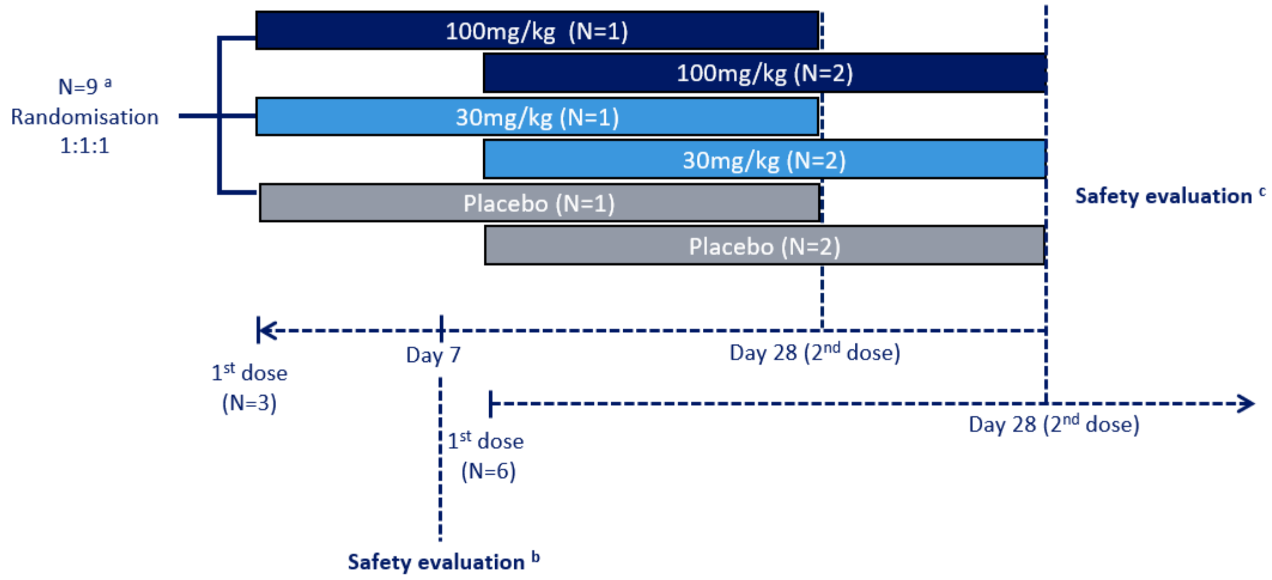

[a] The sentinel cohort will consist of minimum 9 participants, [b] Evaluation of blinded safety data and decision to continue dosing in the sentinel cohort will be performed by an internal Novo Nordisk medical monitoring group, [c] Evaluation of unblinded safety data for minimum 9 participants in the sentinel cohort will be performed by an external Data Monitoring Committee.

In case of reporting of an SAE with a possible or probable relation to the trial product, the Novo Nordisk NNC6019-0001 Safety Committee will be informed immediately and will discuss at a separate meeting if the dosing should be paused, and any action on individual or study level should be taken towards current ongoing treatment or follow-up investigations.

In addition, an external Data Monitoring Committee (see Appendix 1, Section 10.1.6) will evaluate unblinded data for the minimum 9 participants in the sentinel cohort at a separate meeting and will give their recommendation for the continued conduct of study. Minimum 9 participants in the sentinel cohort must have attended visit 3 before the Data Monitoring Committee evaluation can take place and before randomising further participants into the main cohort. The Data Monitoring Committee will continue to monitor the study based on unblinded data until study closure.

Participants in the sentinel cohort who do not attend visit 3 will be replaced, and a new participant will be allocated to the same treatment. Additionally, participants in the sentinel cohort must have an ECG and echocardiography evaluated by the investigator before the planned second dosing.

Continuous cardiac monitoring may be performed at subsequent dosing visit as clinically indicated or at the discretion of the investigator.

Japan: For country-specific requirements, please refer to Appendix 8 (Section 10.8).

Non-investigational medicinal products (NIMP)

The NIMPs are listed in Table 6-1.

Auxiliary supplies including medical device(s) not under investigation

Auxiliaries needed for preparation and administration of each dose will not be provided by Novo Nordisk.

6.2 Preparation, handling, storage and accountability

Only participants enrolled in the study may use study intervention and only delegated site staff may administer study intervention.

Each site will be supplied with sufficient trial product (vials with NNC6019-0001) for the study on an ongoing basis according to recruitment and randomisation.

Receipt of trial product (vials with NNC6019-0001) shipments, storage, and preparation including blinding of trial product, accountability and disposition records will be maintained by investigator´s unblinded designee.

Each vial with NNC6019-0001 will be reconstituted with 4.9 mL sterile water for injection (SWFI) to a concentration of 50 mg/mL. The reconstituted solution will be diluted in normal saline for infusion (total volume 250 mL). The total volume infused will be ~280 mL including the saline flush volume of ~30 mL. Details on the preparation procedures are described in the Trial Materials Manual (TMM).

Body weight is measured at every visit and the latest available body weight is used for calculating the dose. Number of vials with NNC6019-0001 needed for each dose will be calculated based on

body weight. Dose must be calculated based on a body weight of maximum 120 kg (264.6 lb). If a participant's body weight increases above that during the study, the dose calculation must remain based on 120 kg (264.6 lb).

Administration of blinded trial product to study participants will be done by blinded site staff. Blinded site staff will document in source if the fully prepared dose was administrated to the participant.

Acceptable temperature ranges and conditions for storage and handling of trial product (vials with NNC6019-0001) when not in use and when in use are described in the TMM.

The investigator's unblinded designee must confirm that appropriate temperature conditions have been maintained during transit for all trial product (vials with NNC6019-0001) received, and that any discrepancies are reported and resolved before use of the trial product (vials with NNC6019-0001).

All trial product (vials with NNC6019-0001) must be stored in a secure, controlled, and monitored (manual or automated) area in accordance with the labelled storage conditions with access limited to investigator´s unblinded designee.

The investigator's unblinded designee must inform Novo Nordisk immediately if any trial product (vials with NNC6019-0001) has been stored outside specified conditions. The trial product (vials with NNC6019-0001) must not be dispensed to any participant before it has been evaluated and approved for further use by Novo Nordisk. Additional details regarding handling of temperature deviations can be found in the TMM.

The investigator's unblinded designee is responsible for trial product (vials with NNC6019-0001) accountability and record maintenance (i.e., receipt, accountability, and final disposition records). Trial product (vials with NNC6019-0001) accountability must be performed by investigator´s unblinded designee using a trial product accountability log and must be registered in the RTSM/IWRS. To avoid accidental site/pharmacy staff exposure to trial product, accountability of infusion bags will not be done as these containers are single-use and not intended for longer-term storage. Infusion bags should be discarded immediately after use together with the infusion kit.

Destruction of trial products (vials with NNC6019-0001) can be performed on an ongoing basis and will be done according to local procedures after accountability is finalised by the investigator´s unblinded designee and reconciled by the unblinded monitor.

All expired or damaged trial products (vials with NNC6019-0001) including package (for technical complaint samples, see Appendix 5 [Section 10.5]) must be stored separately from non-allocated trial product (vials with NNC6019-0001) by the investigator´s unblinded designee. No temperature monitoring is required.

Non-allocated trial product (vials with NNC6019-0001), including expired or damaged products, must be accounted as unused, at the latest at closure of the site by the investigator´s unblinded designee.

Instructions for auxiliary supplies are described in the TMM.

Japan: For country-specific requirements, please refer to Appendix 8 (Section 10.8).

6.3 Measures to minimise bias: Randomisation and blinding

6.3.1 Randomisation

This is a randomised, placebo-controlled study. Participants will be randomised 1:1:1 to intervention groups as illustrated in Figure 4-1. Participants will be stratified by disease type (wtATTR vs hATTR) and maximum 80% of participants randomised will be participants with wtATTR. Within each stratum, each participant will be randomly allocated to one of the intervention groups.

Sentinel cohort: Stratification by disease type will not be applied for participants in the sentinel cohort. This is in order to ensure the 1:1:1 distribution to the different intervention groups with the small participant number. Japan: For country-specific requirements, please refer to Appendix 8 (Section 10.8).

All participants will be screened and centrally randomised using the RTSM/IWRS and assigned to the next available treatment according to the randomisation schedule. Trial product will be allocated by the RTSM/IWRS and dispensed and prepared for administration by investigator´s unblinded designee at the study visits summarised in the flowchart (Section 1.2).

At screening, each participant will be assigned a unique 6-digit number which will remain the same throughout the study. Each site is assigned a 3-digit number and all subject IDs will start with the site number. Subject IDs must not be re-assigned.

6.3.2 Blinding

This is a double-blind study in which participants, care providers, investigators and outcome assessors are blinded to trial product allocation. To preserve the blinding of the study in the event of interim evaluation, only a minimum number of Novo Nordisk personnel are allowed to see the randomisation table and intervention assignments before the study is completed (see Section 9.4 for further details).

Investigators and other site staff with participant contact will remain blinded throughout the course of the study. In order to maintain this blind, unblinded delegated site staff or pharmacy staff not otherwise involved in the study procedures will be responsible for the shipment receipt, storage, returns, accountability and destruction of trial product (vials with NNC6019-0001) and dispensing, preparation, and blinding of trial product. See further details in Section 6.2 and in the TMM.

In the event of a Quality Assurance audit, the auditor(s) will be allowed access to unblinded trial product records at the site/pharmacy to verify that randomisation/dispensing has been done accurately.

6.3.3 Blind Break

The RTSM/IWRS is used for blind-breaking. In case of an emergency, the investigator has the sole responsibility for determining if unblinding of a participant’s trial product is warranted. Participant safety must always be the first consideration in making such a determination. If the investigator

decides that unblinding is warranted, the investigator should make every effort to contact Novo Nordisk prior to unblinding a participant's study intervention unless this could delay emergency treatment of the participant.

If a participant's trial product is unblinded, Novo Nordisk (Global Safety department) must be notified within 24 hours after breaking the blind. The date and reason that the blind was broken must be recorded in the source documentation. The person breaking the blind must print the blind break confirmation notification generated by the RTSM/IWRS, sign and date the document. If RTSM/IWRS is not accessible at the time of blind break, the RTSM/IWRS helpdesk should be contacted. Contact details are listed in Attachment I.

If there are no safety concerns at the discretion of the investigator, the participant may continue trial product.

Trial product allocation will also be accessible to:

- The laboratory responsible for analysis of NNC6019-0001 anti-drug antibodies, and the responsible analytical scientist at special lab.
- The Novo Nordisk laboratory responsible for analysis of NNC6019-0001 PK and the responsible analytical scientist.
- The Novo Nordisk unblinded monitor.

6.4 Study intervention compliance

Drug treatment compliance and compliance with other interventions

Throughout the study, the investigator will remind the participants to follow the study procedures and requirements to encourage participant compliance.

The participants are dosed at the site. They will receive trial product directly from the investigator or designee, under medical supervision. The date and start and stop time of each dose administered at the site will be recorded in the source documents and eCRF. For guidance in the event of overdose, please refer to Section 6.7.

Site staff will observe ingestion of the pre-medication as per Table 6-1. If in doubt whether the pre-medication has been swallowed, site staff will enter into a dialogue with the participant, acknowledging non-compliance can happen and addressing barriers to compliance. Pre-medication should be entered as concomitant medication in the eCRF.

6.5 Dose modification

Not applicable for this study.

6.6 Continued access to study intervention after end of study

When discontinuing study intervention, the participant should be transferred to a suitable marketed product at the discretion of the investigator.

6.7 Treatment of overdose

There is no previous experience of an overdose with NNC6019-0001 in humans. Any dose of NNC6019-0001 greater than i.v. 30 mg/kg or 100 mg/kg Q4W, depending on the treatment group, and/or less than 20 days apart will be considered an overdose.

Treatment of overdose with NNC6019-0001 should consist of general supportive measures, if applicable. There is no known specific antidote for overdose with NNC6019-0001.

Accidental overdose must be reported as a medication error. Intentional overdose must be reported as misuse and abuse, please refer to Section 8.4 and Appendix 3 (Section 10.3.3) for further details.

In the event of an overdose, the investigator should closely monitor the participant for overdose-related AEs/SAEs The length of observation and treatment should be guided by the clinical picture and medical judgment.

Decisions regarding dose interruptions will be made by the investigator based on the clinical evaluation of the participant.

For more information on overdose, also consult the current version of the NNC6019-0001 investigator's brochure (IB)[28] or updates thereof.

6.8 Concomitant therapy

NNC6019-0001 will be used on top of standard-of-care treatment. Changes in standard-of-care treatment should be avoided and increase in dose or addition of concomitant medication with TTR-targeted treatment such as patisiran or tafamidis after randomisation will generally not be allowed, unless a change in standard-of-care treatment is indicated based on a deterioration in the clinical condition of the participant as evaluated by the investigator. A worsening of the pre-existing condition will be required to be registered in the AE form.

Additional medications to treat participants´ conditions may be added or changed during the study at the discretion of the investigator and in accordance with local treatment guidelines and policies. Standard-of care treatment is considered background treatment and will not be provided by Novo Nordisk A/S.

Treatment with calcium channel blockers with conduction system effects (e.g., verapamil, diltiazem) or digoxin not prescribed for management of atrial fibrillation with rapid ventricular response is contraindicated in ATTR CM and is not allowed during study conduct (see Sections 5.2 and 7.1).

Any medication or vaccine (including over the counter or prescription medicines, that the participant is receiving at the time of the first visit or receives until end of study must be recorded along with:

- Trade name or generic name
- Primary indication
- Dates of administration including start and stop dates
- Dose and unit, frequency, route of administration

Changes in concomitant therapy must be recorded at each visit. If a change is due to an AE, then this must be reported according to Section 8.4.

7 Discontinuation of study intervention and participant discontinuation/withdrawal

Discontinuation of specific sites or of the study as a whole is detailed in Appendix 1 (Section 10.1.11).

7.1 Discontinuation of study intervention

Study intervention may be discontinued at any time during the study at the discretion of the participant or at the discretion of the investigator for safety, behavioural, compliance or administrative reasons.

Efforts must be made to have participants who discontinue study intervention continue the planned visit schedule and assessments. As a minimum, efforts must be made to have those participants attend the scheduled milestone visits (Visit 3, Visit 5, Visit 8 and Visit 15) on site to ensure continued counselling and data collection. Participants should be informed about the continued scientific importance of their data, even if they discontinue study intervention. Only participants who withdraw consent will be considered as withdrawn from the study. The follow-up visit (Visit 16) should be scheduled 16 weeks after last dose of study intervention. If this coincides with a scheduled visit, assessments pertaining to visit 16 should be performed in addition to the visit assessments. See the flowchart for data to be collected at the milestone visits and follow-up visit and for any further evaluations that need to be completed (Section 1.2).

If the participant does not wish to attend the scheduled visits, efforts should be made to have remaining visits converted to remote contacts, carrying out the protocol assessments deemed feasible to conduct remotely. If a participant is unwilling to attend remaining visits, information about the attempts to follow up with the participant should be documented in the participant's medical record.

The study intervention must be discontinued, if any of the following applies for the participant:
1. Pregnancy
2. Intention of becoming pregnant
3. Initiation of treatment with calcium channel blockers with conduction system effects (e.g., verapamil, diltiazem) or digoxin not required for management of atrial fibrillation with rapid ventricular response
4. Solid organ transplant during the study
5. Simultaneous use of an approved or non-approved investigational medicinal product in another clinical study
6. Safety concern as judged by the investigator

If a participant meets a discontinuation criterion judged by the investigator as reversible, study intervention can be resumed when the discontinuation criterion is no longer met, unless any of the following becomes applicable for the participant in the meantime:
- participant met a discontinuation criterion that is judged by the investigator as irreversible
- participant missed 3 consecutive doses of study intervention

The primary reason for discontinuation of study intervention must be specified in the eCRF, and final trial product (vials with NNC6019-0001) accountability must be performed once the participant has permanently discontinued study intervention. Treatment discontinuation must be registered in the RTSM/IWRS.

7.1.1 Temporary discontinuation of study intervention

If a participant discontinued study intervention temporarily, study intervention can be resumed if considered safe at the investigator's discretion and if the participant does not meet any of the discontinuation criteria (Section 7.1). Similarly, a participant who discontinues study intervention on their own initiative should be encouraged to resume the study intervention if deemed safe by the investigator (see Section 6.1).

A treatment discontinuation and treatment resume must be registered in RTSM/IWRS when a participant discontinues or resumes study intervention. Missed doses are recorded in the eCRF.

7.2 Participant discontinuation/withdrawal from the study

A participant may withdraw consent at any time at his/her own request.

If a participant withdraws consent prior to randomisation, the participant will not be asked to have any follow-up assessments performed. The following data must be collected: Demography, eligibility criteria, date of informed consent, date of screening and the date when participant's participation ended. The end of study form must be completed in eCRF.

If a participant withdraws consent as registered in the RTSM/IWRS between randomisation and prior to first dosing administration, the participant will not be asked to have any follow-up assessments performed. This withdrawal of consent should be captured in the first dose after randomisation form and the end of study form in eCRF.

If a participant withdraws consent after receipt of study intervention, the investigator must ask the participant if he/she is willing, as soon as possible, to have assessments pertaining to safety performed according to Visit 16. See the flowchart for data to be collected.

Discontinuation of study intervention must be registered in the RTSM/IWRS.

If the participant withdraws consent, Novo Nordisk may retain and continue to use any data collected before such a withdrawal of consent for the purpose of the study or scientific research.

If a participant withdraws from the study, the participant may request destruction of any samples taken and not tested, and the investigator must document this in the medical record and inform Novo Nordisk that samples should be destroyed.

Although a participant is not obliged to give his/her reason(s) for withdrawing, the investigator must make a reasonable effort to ascertain the reason(s), while fully respecting the participant's rights. Where the reasons are obtained, the primary reason for withdrawal must be specified in the eCRF.

7.2.1 Replacement of participants

Sentinel cohort: If a sentinel participant discontinues study intervention or withdraws consent before attending visit 3, the participant will be replaced unless procedures described in Appendix 3 (Section 10.3) are applicable. Sentinel participants will not be replaced if they discontinue study intervention/withdraws consent at or after Visit 3.

If a non-sentinel participant discontinues study intervention or withdraws consent after first dose (Visit 2), the participant will not be replaced.

If a non-sentinel participant withdraws consent or is lost to follow up prior to first dose administration (Visit 2), the participant will be replaced.

7.3 Lost to follow-up

A participant will be considered lost to follow-up if he/she repeatedly fails to return for scheduled visits and is unable to be contacted by the site.

The following actions must be taken if a participant fails to return to the site for a required visit:

- The site must attempt to contact the participant and reschedule the missed visit as soon as possible and counsel the participant on the importance of maintaining the assigned visit schedule and ascertain whether the participant wishes to and/or should continue in the study.
- Before a participant is deemed lost to follow-up, the investigator or designee must make every effort to regain contact with the participant (where possible, at least three telephone calls and, if necessary, a certified letter to the participant’s last known mailing address or local equivalent methods). These contact attempts should be documented in the participant’s source document.
- Should the participant continue to be unreachable when visit 15 is due, the participant will be considered to have withdrawn from the study with a primary reason of ‘lost to follow-up’.
- Site personnel, or an independent third party, will attempt to collect the vital status of the participant within legal and ethical boundaries for all participants randomised. Public sources may be searched for vital status information. If vital status is determined as deceased, this will be documented, and the participant will not be considered lost to follow-up. Sponsor personnel will not be involved in any attempts to collect vital status information.

If a participant is lost to follow-up as registered in the RTSM/IWRS between randomisation and prior to first dosing administration, the above listed actions are not required. The lost to follow-up should be captured in the first dose after randomisation form and the end of study form in eCRF.

8 Study assessments

The following sections describe the assessments and procedures, while their timing is summarised in the flowchart.

The following general assessments and procedures must be followed in the study:

- Informed consent must be obtained before any study-related activity, see Appendix 1 (Section 10.1.3).
- All screening evaluations must be completed and reviewed to confirm that potential participants meet all inclusion criteria and none of the exclusion criteria. Note: results from both Visit 1 and Visit 1A, when applicable, should fulfil the eligibility criteria.
- The investigator will maintain a screening log to record details of all participants screened and rescreened and to confirm eligibility or record reason for screen failure, as applicable.
- At screening, participants will be provided with a card stating that they are participating in a study and giving contact details of relevant site staff that can be contacted in case of emergency.
- Adherence to the study design requirements, including those specified in the flowchart, is essential and required for study conduct.
- Assessments should be carried out according to the standard of care unless otherwise specified in the current section. Efforts should be made to limit bias between assessments. Assessments should be caried out in the following order:
  1. Clinical outcome assessments; the following patient reported outcome (PRO) questionnaires are suggested to be performed in the following order:
     - EQ-5D-5L
     - PGI-S for KCCQ
     - PGI-C for KCCQ (when applicable)
     - KCCQ
  2. Electrocardiogram (ECG) and vital signs
  3. Blood sampling
  4. Clinical outcome assessments; the following assessments are suggested to be performed in the following order:
     - 6-MWT (must be performed after ECG, first vital sign assessment and blood sampling)
     - PGI-S for walking
     - PGI-C for walking (when applicable)
     - NIS (when applicable)
  5. Other assessments
- First dose must only be administered after assessments related to primary and/or secondary endpoints are completed (e.g., blood samples taken, echocardiography, cardiac MRI and PRO questionnaires completed).
- Review of PRO instruments, ECG, laboratory reports, etc., must be documented in the source documents or the participant’s medical record. If clarification of entries or discrepancies in the PRO instruments is needed, the participant must be questioned, and a conclusion made in the participant’s source documents. Care must be taken not to bias the participant.
- Repeat samples may be taken for technical issues and unscheduled samples or assessments may be taken for safety reasons. Please refer to Appendix 2 (Section 10.2) for further details on laboratory samples.

- Participants are not required to attend any visits in a fasting state. The time since last meal is collected when blood samples are taken.

8.1 Screening

Demography

The following information must be recorded after informed consent at the screening visit:

- Date of birth, unless not permitted by local regulations
- Year of birth
- Age (should be recorded in the RTSM/IWRS)
- Sex
- Race, unless not permitted by local regulations
- Ethnicity, unless not permitted by local regulations

Germany, France, Netherlands: For country-specific requirements, please refer to Appendix 8 (Section 10.8).

Tobacco use

Details of tobacco use must be recorded at screening (visit 1). Smoking is defined as smoking at least one cigarette or equivalent daily. The collected information should include whether the participant smokes or has smoked.

Smoking status information to be collected:

- Never smoked
- Previous smoker (smoking stop date)
- Current smoker

Childbearing Potential

The assessment of women's childbearing potential should be performed at the screening visit and recorded as specified in the flowchart (Section 1.2) and Appendix 4 (Section 10.4).

Documentation can come from the site staff's review of participant's medical records, medical examination, or medical history interview. Documentation of women's child-bearing potential must be recorded in the eCRF.

Central review of imaging diagnostic criteria

Information regarding the specific assessments justifying the diagnostic conclusion of ATTR CM should be reported in the eCRF in a customised ATTR CM medical history form. If the diagnosis is based on non-invasive diagnostic criteria, it will be required to upload technetium-labelled PYP/DPD/HMDP cardiac scintigraphy including SPECT imaging[37] (confirming that cardiac radiotracer uptake corresponds to the myocardium). Approximately 1 to 2 appointed external medical experts will review the criteria and evaluate the scintigraphy/SPECT imaging. In case a participant does not fulfil the pre-defined centralised diagnostic criteria as assessed by the expert, the participant will not be eligible for the study.

8.2 Efficacy assessments

Planned time points for all efficacy assessments are provided in the flowchart (Section 1.2).

8.2.1 Clinical efficacy laboratory assessments

All protocol-required laboratory assessments, as defined in Appendix 2 (Section 10.2), must be conducted in accordance with the flowchart (Section 1.2) and the laboratory manual.

8.2.1.1 Urine collection

Participants must collect first morning void urine samples in accordance with the flowchart. Urine must be collected in the containers which are to be provided at the previous visit. The investigator should remind the participant to collect first morning void urine samples e.g., via a telephone call or a text message.

Participants must collect two first morning void urine samples:

- one day before the visit
- on the day of the visit

If the participant has not collected the required first morning void urine samples, the participant must be asked to provide new samples (within the visit window) to replace the missed samples.

8.2.2 Clinical outcome assessments

The 6-MWT must be performed in accordance with the manual provided by Novo Nordisk. The below clinical outcome assessments test will be performed.

8.2.2.1 6-minute walk test (6-MWT)

The 6-MWT assesses the distance a participant can walk in six minutes. It is a direct and timed measure of walking ability, which is technically simple, reproducible, and when administrators are well trained, readily standardised. The goal is for the participant to walk as far as possible in six minutes without running. The 6-MWT must be performed in accordance with the manual provided by Novo Nordisk.

If it is identified at Visit 1 that Visit 1A will be required, the 6-MWT may be omitted at Visit 1.

8.2.2.2 Neuropathy Impairment Score (NIS)

Neuropathy impairment score (NIS) is a clinical assessment that tests muscle strength, reflex activity, and sensation of toes and fingers, and can be used to assess neurologic function in hATTR polyneuropathy over time. The NIS assessment is only applicable for participants with hATTR CM and will be performed by a trained neurologist or a physician trained in using the NIS. The NIS assessment should be evaluated, signed and dated by the investigator and filed on the participant’s medical record. The assessment should be recorded in the eCRF.

8.2.2.3 Patient reported outcome (PRO) questionnaires

Participants should be given the opportunity to complete the questionnaires by themselves without interruption. Review of completed PROs must be documented in the source document. The review

must be performed by an investigator. The questionnaires take approximately 10 minutes to complete. The below PRO questionnaires will be used.

EuroQoL five dimensions five level (EQ-5D-5L)

The EQ-5D-5L will be used to estimate the impact on participants' health-related quality of life and provides a description of participants' problems by dimensions (descriptive system), a score for overall self-rated health (visual analogue scale (VAS) as well as an index score (EQ-5D-5L index). EQ-5D index score range: 0 to 1 and EQ-5D-VAS: range 0 to 100. A higher score indicates better self-reported health status. If clarification of the test is needed, care should be taken not to bias the participant.

Kansas City Cardiomyopathy Questionnaire (KCCQ)

The KCCQ is a disease-specific health status instrument composed of 23 items that quantify the domains of physical limitation, symptoms, self-efficacy, social limitation, and health-related quality of life limitation from heart failure. The overall summary score and all domains have been independently demonstrated to be valid, reliable, and responsive to clinical change.

Patient Global Impression of Status (PGI-S) and Patient Global Impression of Change (PGI-C)

PGI-S and PGI-C are single-item global rating PRO measures that are used to evaluate the responder threshold. The following PGI-S and PGI-C measures are included:

- Patient Global Impression of Status (PGI-S) for KCCQ version 1.0.
- Patient Global Impression of Change (PGI-C) for KCCQ version 1.0.
- Patient Global Impression of Status (PGI-S) for 6-MWT version 1.0.
- Patient Global Impression of Change (PGI-C) for 6-MWT version 1.0.

8.2.3 Imaging

The images (cardiac MRI and echocardiography) will be performed at study selected imaging units and images will be analysed by a centralised imaging core laboratory. For standardisation purposes the imaging core laboratory will train the study specific imaging units, as applicable, and the imaging core laboratory will do centralised blinded (blinded to treatment allocation) image analysis/interpretation. The acquisition, display, interpretation, and archiving process of images will be described in an imaging charter and in an imaging manual that will be finalised as supplementary documents before study start. Results from the imaging analyses will be transferred electronically directly from the centralised imaging core laboratory to Novo Nordisk and not shared with site.

8.2.3.1 Cardiac magnetic resonance imaging (MRI)

Cardiac MRI should be performed at the timepoints outlined in the flowchart (Section 1.2) except if a participant has a contraindication to MRI according to local standards. If a participant has a contraindication to MRI, the participant will continue in the study without MRI assessments.

The imaging site should use the same approved MR scanner for all scheduled MR scans. The cardiac MRI scans can be performed ± 2 weeks of the respective visit, except for the first dosing visit where it can be performed up to two weeks in advance (full eligibility needs to be confirmed beforehand). In order to standardise the examination, an MRI site instruction manual will be

provided to guide investigators on the image acquisition and the analysis of the planned and optional assessments.

The investigator should ensure that a standard assessment of MR images is performed by a qualified cardiologist/radiologist. In case of any incidental findings, the investigator should be informed and assess if any AEs are to be reported. It is the responsibility of the investigator to refer the participant to further examination and treatment based on the incidental findings as medically indicated. Continued study participation or discontinuation should be considered by the investigator.

To calibrate the MRI, the imaging core laboratory may test the imaging protocol in healthy volunteers. This test will check the MRI settings and the quality of the images generated. Novo Nordisk will not have access to any data generated from the MRI examination on the healthy volunteers and these participants are therefore not considered part of the study.

8.2.3.2 Echocardiography

A standard echocardiographic examination should be performed at the timepoints outlined in the flowchart (Section 1.2). Echocardiographic examination should be performed pre-infusion and the echocardiogram must be assessed locally prior to infusion of trial product. The echocardiography should be interpreted (categorised as normal or abnormal, and, if abnormal, furthermore indicate whether the finding was clinically relevant), signed and dated by the investigator and filed on the participant's medical record. The echocardiography overall evaluation and corresponding outcomes should be documented in source notes and recorded in the eCRF.

Acquisition of echocardiographic images including the analysis should be performed locally by personnel trained in echocardiography. To standardise the examination, an echocardiography site instruction manual will be provided to guide investigators on the image acquisition and the analysis of the planned and optional assessments.

All echocardiography assessments should be performed in accordance with the site instruction manual.

8.3 Safety assessments

Planned time points for all safety assessments are provided in the flowchart (Section 1.2).

Concomitant illness and medical history

A **concomitant illness** is any illness that is already present at the time point from which AEs are collected or found as a result of a screening procedure or other study procedures performed before exposure to study intervention under clinical investigation.

Medical history is a medical event that the participant has experienced in the past. Only relevant medical history should be reported prior to the time point from which AEs are collected.

In case of an abnormal and clinically significant finding fulfilling the definition of medical history or concomitant illness, the investigator must record the finding on the medical history/concomitant illness form.

The following Medical History/Concomitant Illness should be reported in the eCRF:

- History of heart failure
- History of heart rhythm and conduction disturbances
- History of hypotension
- History of peripheral neuropathy
- History of ATTR CM and the exact genetic type if applicable

Any change to a concomitant illness should be recorded during the study. A clinically significant worsening of a concomitant illness should be reported as an AE.

8.3.1 Physical examinations

A physical examination will include assessments of:

- General appearance
- Head, ears, eyes, nose, throat, neck
- Respiratory system
- Cardiovascular system
- Gastrointestinal system incl. mouth
- Musculoskeletal system
- Central and peripheral nervous system
- Skin
- Lymph node palpation
- Endocrine system
- Genitourinary system

Investigators should pay special attention to clinical signs related to previous serious illnesses.

Any abnormal, clinically significant findings prior to start of first dose should be recorded as concomitant illness. Any clinically significant worsening from dosing should be reported as an AE (see Section 8.4).

8.3.2 New York Heart Association (NYHA) classification

The investigator should assess the functional status of the participant based on New York Heart Association (NYHA) classification as specified in the flowchart (Section 1.2). Documentation of the assessment should be recorded in the eCRF.

8.3.3 Body measurements

Body measurements (e.g., height and weight) will also be measured and recorded as specified in the flowchart (Section 1.2).

Body weight

Body weight should be measured with an empty bladder, without shoes and only wearing light clothing on a calibrated scale. Body weight is recorded in kilograms (kg) or pounds (lb).

Height

Height is measured without shoes in centimetres (cm) or inches (in).

8.3.4 Vital signs

Ear temperature, pulse rate, as well as systolic and diastolic blood pressure will be assessed and recorded as specified in the flowchart (Section 1.2) and Table 6-2.

Blood pressure and pulse rate

Blood pressure and pulse rate measurements should be preceded by at least 5 minutes of rest for the participant in a quiet setting without distractions (e.g., no use of television, cell phones).

The initial blood pressure and pulse rate measurements at each visit should be assessed sitting. The subsequent measurements at each visit can be assessed sitting or lying. Please see Table 6-2 for timing of measurements.

Blood pressure and pulse rate measurements will be assessed with a completely automated device. Manual techniques must be used only if an automated device is not available.

Blood pressure and pulse rate are collected as specified in the flowchart (Section 1.2).

Blood pressure will consist of 3 systolic and diastolic blood pressure measurements with intervals of at least 1-2 minutes. An additional fourth blood pressure measurement must be performed if the first two readings on systolic or diastolic blood pressure differ by >10 mmHg. No more than four measurements should be performed.

- The last 2 systolic and last 2 diastolic blood pressure measurements should be recorded in the eCRF.

Pulse rate will be measured in connection to the blood pressure measurements.

- The pulse rate for the last 2 measurements should be recorded in the eCRF.

Body temperature

Body temperature should be measured in the ear with a calibrated thermometer. Body temperature is recorded in degree Celsius (°C) or Fahrenheit (°F) with a precision of one decimal. For details on timepoints for body temperature measurements (Table 6-2). The body temperature measures should be recorded in the eCRF.

8.3.5 Electrocardiograms

12-lead ECG will be obtained as outlined in the flowchart using an ECG machine that automatically calculates the heart rate and measures PR, QRS, QT and QT_c intervals.

The ECG should be interpreted (categorised as normal or abnormal, and, if abnormal, furthermore indicate whether the finding was clinically relevant with a short description, signed and dated by the investigator and filed on the participant's medical record. The ECG measures and corresponding outcomes should be recorded in the eCRF.

Any abnormal clinically relevant findings revealing baseline conditions are to be reported as concomitant illness/medical history in the eCRF. Any clinically significant worsening of a pre-existing condition as well as any new clinically relevant signs, symptoms or disease found as a

result of the ECGs conducted after randomisation are to be reported as AEs (please refer to Section 8.4).

Additional ECG recordings can be performed at the investigator's discretion, in which case the reason is to be documented, and an AE reported if applicable.

8.3.6 Cardiac monitoring (only appliable for sentinel participants)

All sentinel participants will be cardiac monitored on both in- and outpatient basis. Cardiac monitoring should be performed at the timepoints outlined in the flowchart (Section 1.2). The cardiac monitoring results should be interpreted (categorised as normal or abnormal, and, if abnormal, furthermore indicate whether the finding was clinically significant), signed and dated by the investigator and filed on the participant's medical record. The cardiac monitoring report over all interpretation and corresponding outcomes should be recorded in the eCRF.
Japan: For country-specific requirements, please refer to Appendix 8 (Section 10.8).

Outpatient 48-hours cardiac monitoring (before randomisation)

During screening, participants will undergo cardiac monitoring (Holter ECG) on an outpatient basis for at least 48-hours within 2 weeks prior to the randomisation visit. Interpretation and review of the cardiac monitoring report is required prior to randomisation of participants (see Section 5.5).

Inpatient 24-hours cardiac monitoring

When receiving the first dose, participants will be observed under continuous bedside cardiac monitoring for at least 24-hours prior to discharge. Japan: For country-specific requirements, please refer to Appendix 8 (Section 10.8).

Outpatient 6-day cardiac monitoring

On discharge, participants will be monitored using cardiac monitoring (Holter ECG) on an outpatient basis until 7 days after start of infusion. Japan: For country-specific requirements, please refer to Appendix 8 (Section 10.8).

8.3.7 Clinical safety laboratory assessments

All protocol-required laboratory assessments, as defined in Appendix 2 (Section 10.2), must be conducted in accordance with the laboratory manual and the protocol flowchart.

8.3.8 Pregnancy testing

Woman of childbearing potential (WOCBP) should only be included after a negative, highly sensitive urine pregnancy test (see Appendix 2 [Section 10.2]).

Pregnancy testing should be performed whenever a menstruation is missed or when pregnancy is otherwise suspected.

Additional pregnancy testing should be performed during the treatment period, if required locally, refer to Appendix 8 (Section 10.8).

8.4 Adverse events and other safety reporting

The investigator is responsible for detecting, documenting, recording, and following up on events that meet the definition of an AE or SAE.

The definition of AEs and SAEs can be found in Appendix 3 (Section 10.3), along with a description of AEs requiring additional data collection. The definition and description of events for adjudication can be found in Appendix 7 (Section 10.7).

Some AEs require additional data collection on a specific event form. The relevant event(s) are listed below in Table 8-1, together with event(s) for adjudication

Events for adjudication require completion of an adjudication form, please refer to Appendix 7 (Section 10.7).

Table 8-1 AEs requiring additional data collection and events for adjudication

**Event type**	**AE requiring additional data collection**	**Event for adjudication**
Medication error, misuse and abuse	X	
Hypersensitivity reactions	X	
Myocardial inflammation	X	
Pro-arrhythmic risk	X	
Death		X
Cardiovascular hospitalisation[a]		X
Urgent heart failure visit not requiring hospitalisation		X

[a] All hospitalisations will be adjudicated

Definitions and reporting timelines for the events mentioned in the above table can be found in Appendix 3 (Section 10.3) and Appendix 7 (Section 10.7) for events requiring adjudication.

8.4.1 Time period and frequency for collecting AE information

All AEs and SAEs must be collected from first administration of trial product under clinical investigation (randomisation visit) and until the follow-up visit in accordance with the flowchart (Section 1.2) or whenever, within the above time period, the site becomes aware of an AE or SAE.

AEs and SAEs are collected from first administration of trial product under clinical investigation (randomisation visit) as no invasive procedures which can give AEs/SAEs are performed during the screening period.

Conditions present prior to the timepoint from which AEs are collected and anticipated day-to-day fluctuations of these conditions, including those identified during screening or during other study-related procedures performed before exposure to study intervention under clinical investigation, will be recorded as medical history/concomitant illness.

AE and SAE reporting timelines can be found in Appendix 3 (Section 10.3). All SAEs must be recorded and reported to Novo Nordisk within 24 hours, and the investigator must submit any updated SAE data to Novo Nordisk within 24 hours of it being available.

Investigators are not obligated to actively seek for AE or SAE in former study participants. However, if the investigator learns of any SAE, including a death, at any time after a participant has discontinued from/completed the study, and the investigator considers the event to be related to the IMP or related to study participation, the investigator must promptly notify Novo Nordisk.

8.4.2 Method of detecting AEs

The method of recording, evaluating, and assessing causality of AE and SAE and the procedures for completing and transmitting SAE reports are provided in Appendix 3 (Section 10.3).

Care should be taken not to introduce bias when detecting AEs and/or SAEs. Open-ended and non-leading verbal questioning of the participant is the preferred method to inquire about events.

8.4.3 Follow-up of AEs

After the initial AE/SAE report, the investigator is required to proactively follow each participant at subsequent visits/contacts. All SAEs should be followed until final outcome of the event or until the participant is lost to follow-up as described in Section 7.3. Further information on follow-up and final outcome of events is given in Appendix 3 (Section 10.3).

8.4.4 Regulatory reporting requirements for SAEs

Prompt notification by the investigator to Novo Nordisk of an SAE is essential so that legal obligations and ethical responsibilities towards the safety of participants and the safety of a study intervention under clinical investigation are met.

Novo Nordisk has a legal responsibility to notify both the local regulatory authority and other regulatory agencies about the safety of a study intervention under clinical investigation. Novo Nordisk will comply with country-specific regulatory requirements relating to safety reporting to the regulatory authority, IRB/IEC, and investigators. This also includes suspected unexpected serious adverse reactions (SUSAR)

An investigator who receives an investigator safety report describing an SAE or other specific safety information (e.g., summary or listing of SAEs) from Novo Nordisk will review and then file it along with the investigator's brochure and will notify the IRB/IEC, if appropriate according to local requirements.

8.4.5 Pregnancy

Details of pregnancies in female participants will be collected after first exposure to IMP and until pregnancy outcome. For details regarding collection and reporting of pregnancy information, please refer to Appendix 4 (Section 10.4).

8.4.6 Cardiovascular and death events

Cardiovascular and death events will be handled and reported according to Section 8.4.

8.4.7 Technical complaints

Technical complaints will be collected for all products listed on the technical complaint form.

Instructions for reporting technical complaints can be found in Appendix 5 (Section 10.5).

In order for Novo Nordisk to perform a complete investigation of reported SAEs, Novo Nordisk might ask the investigator to complete a technical complaint form.

8.5 Pharmacokinetics and pharmacodynamics

8.5.1 Pharmacokinetics

The purpose of measuring plasma NNC6019-0001 levels is to conduct population PK and exposure-response analyses. Single blood samples for measuring plasma concentration of NNC6019-0001 will be drawn on visits specified in the flowchart (Section 1.2). The exact timing (date and time) of obtaining the pharmacokinetic (PK) sample should be recorded in the eCRF.

Blood samples for PK assessments should be collected, handled, stored, labelled, and shipped according to the description in the laboratory manual supplied by the central laboratory. The bioanalysis of NNC6019-0001 PK will be performed by Novo Nordisk laboratory. NNC6019-0001 PK samples will be stored at the laboratory responsible until final Clinical Study Report (CSR) in case further analysis of the PK samples is required. Details of the bioanalysis will be outlined in a bioanalytical study plan issued by the special laboratory. Bioanalysis of plasma samples for NNC6019-0001 will be carried out using a validated immunoassay.

Residual PK samples should be retained according to Appendix 6 (Section 10.6.4).

8.6 Genetics

Not applicable for this study.

8.7 Biomarkers

Collection of samples for biomarker research is part of this study. The following samples are required and will be collected from all participants in this study:

- blood samples

The detailed sampling regimen is described in the flowchart (Section 1.2) and defined in Appendix 2 (Section 10.2). Biomarkers evaluated in the study include both circulating and imaging biomarkers, sampled at multiple time points throughout the study. The relevant biomarkers directly reflect cardiac status as well as systemic effects related to the target biology.

Some of the biomarkers are also defined as endpoints (Section 3) or assessments (Section 10.2) in this study, and, thus, used for the efficacy and safety evaluation. These include biomarkers related to cardiac status (NT-proBNP and troponin I, GLS, and ECV), inflammation, (hsCRP, IL-6, IL-8, TNF-α, complement C3 and C4), and target biology (misTTR). These pre-defined biomarkers will be analysed prior to database lock (DBL) and reported in the CSR.

Additionally, non-cardiac specific biomarkers related to the target biology, include tetrameric TTR assessments, and levels of retinol binding protein 4 (RBP4) which in patients with hATTR[38] may indirectly reflect degree of stable tetrameric TTR.

Misfolded TTR serves as a potential treatment response biomarker, this has been shown in the FHD study (study NN6019-4965) and in published studies[39], all in participants with hATTR amyloidosis. In relation to the assessment of misfolded TTR, this biomarker holds potential to be both diagnostic and prognostic. In addition, misfolded TTR could be used to monitor treatment response.

Jointly, the abovementioned biomarkers serve as an important part of demonstrating the efficacy, safety, and mode of action of the drug.

The assessment of misfolded TTR will be performed by Novo Nordisk or a special laboratory contracted by Novo Nordisk. The laboratory will provide instructions on sampling, handling of samples, labelling and shipment of samples, which will be detailed in the laboratory manual provided by central lab. Details of the biomarker analyses will be outlined in a biomarker study plan provided by the analysing laboratory.

Residual biomarker samples for misfolded TTR assessment should be retained according to Appendix 6 (Section 10.6.4).

In addition, biosamples are collected for future biomarker analysis. Refer to Section 8.8.3 for further details and Appendix 6 (Section 10.6) for retention.

8.8 Immunogenicity assessments

8.8.1 Anti-NNC6019-0001-antibodies

Anti-drug-antibody samples will be collected according to the flowchart (Section 1.2). All samples must be drawn prior to trial product administration if trial product administration is planned on the sampling day.

Assessment of antibodies against NNC6019-0001 in plasma will be performed by a special laboratory contracted by Novo Nordisk (please refer to Attachment I).

For details on blood sampling, serum preparation and storage, please refer to the laboratory manual.

Analysis for binding anti-NNC6019-0001-antibodies will be performed using a validated anti-drug antibody assay. Confirmed antibody positive samples will be titrated to evaluate the level of the antibody response. Neutralising effect of the antibodies will be evaluated by correlating binding antibody data to PK and PD. Detailed description of the assay methods will be included in an analytical report. Antibody assays will be validated according to international guidelines and recommendations.

Results from the binding anti-drug antibody analysis will be available for the investigator at the end of study upon request.

At the end of the study, the following data will be electronically transferred to the Novo Nordisk database:

- Anti-NNC6019-0001 binding antibodies (positive/negative)
- Anti-NNC6019-0001-antibody titre (numerical)

The investigator will not be able to review the results of antibody measurements in relation to AEs as these are often analysed after LPLV.

For retention of remaining and residual antibody samples, please refer to Appendix 6 (Section 10.6.2).

8.8.2 Assessments in case of suspicion of hypersensitivity to trial product

Participants and investigators will be instructed to detect signs and symptoms of hypersensitivity reactions:

- Local reactions
- Systemic reactions, including anaphylaxis.
- In the event of a hypersensitivity reaction:
- The participant should contact the site for advice on further action as soon as possible.
- Additional data collection will be performed on the event
- Treatment should be provided by the investigator according to local clinical practice.

Additional blood samples and other tests

In the event of an acute severe **systemic** hypersensitivity reaction (i.e., not local reactions), as judged by the investigator, the participant should be called in as soon as possible to have additional blood samples taken in order to analyse the following parameters:

- Tryptase (optimal 0.5 – 2 hours after the hypersensitivity reaction)
- Complement 50
- Anti-NNC6019-0001 binding antibodies
- Anti-NNC6019-0001 IgE antibodies
- Total IgE

Analysis of tryptase and anti-NNC6019-0001 IgE antibodies will be performed by Novo Nordisk, complement 50 and total IgE will be performed by central laboratory, and anti-NNC6019-0001 binding antibodies will be performed by a special laboratory contracted by Novo Nordisk (please refer to Attachment I).

Data from the additional blood samples and tests will be reported in an analytical report and attached to the clinical study report. Furthermore, the results will be included in the narratives of the clinical study report.

For retention of residual hypersensitivity samples, please refer to Appendix 6 (Section 10.6.3).

8.8.3 Human biosamples for future research

Collection of biosamples for future analysis is a component of this study. The samples will be stored in a biobank and allow for future analyses when new knowledge or improved testing

technologies may have become available during or after the study. Participation is optional, and participants must sign a separate informed consent to indicate their participation in the biobank component(s) of the study. Participants who do not wish to participate in the biobank component(s) may still participate in the study. Blood and urine samples will be collected according to Appendix 6 (Section 10.6.1) and stored for future use.

Genetic analyses may include analysis of selected genes or genetic markers throughout the genome with the purpose of understanding and predicting response to NNC6019-0001 as well as to understand ATTR CM or other related conditions.

Analyses of circulating biomarkers will measure proteins, lipids, peptides, hormones, metabolites or other non-genetic entities with the purpose of understanding and predicting response to NNC6019-0001 as well as understanding ATTR CM or other related conditions.

The samples may be analysed as part of a multi-study assessment. Results will not be reported to the investigator for assessments of AEs nor will they be part of the clinical study report. The primary objective of the analysis is to investigate on a population level and results are very unlikely to have clinical utility on an individual level. Furthermore, the analyses will be done on pseudonymised data. Therefore, any outcome of the analyses will not be reported directly to participants or sites. The result may be reported in publications, at scientific conferences or to authorities.

The human biosamples for future research will be stored for up to 15 years after end of study at a central laboratory or appropriate storage facility (see Appendix 6 [Section 10.6]).

8.9 Health economics

Not applicable for this study.

9 Statistical considerations

The statistical analysis plan (SAP) will be finalised prior to any interim evaluation, and it will include a more technical and detailed description of the statistical analyses and interim analysis than described in this section.

9.1 Statistical hypotheses

No confirmatory statistical hypothesis testing will be done in this study.

9.1.1 Multiplicity adjustment

As no confirmatory hypothesis will be tested statistically, no adjustment for multiplicity will be done for the two primary endpoints.

9.2 Analysis sets

The following participant analysis sets are defined:

Participant analysis set (PAS)	Description
Full analysis set (FAS)	All randomised participants, except participants who initiate the randomisation registration in RTSM/IWRS but withdraw consent, is withdrawn by the investigator, or is lost to follow-up prior to first dosing administration. Participants will be included in the analyses according to the planned intervention.
Safety analysis set (SAS)	All participants who are exposed to study intervention. Participants will be included in the analyses according to the intervention they actually received.

Abbreviations: FAS = full analysis set; PAS = participant analysis set; SAS = safety analysis set; RTMS/IWRS = Randomisation and Trial Supplies Management System / Interactive Web Response System.

9.3 Statistical analyses

9.3.1 General considerations

Estimated treatment effects will be presented with a 95% confidence interval and a two-sided p-value.

9.3.2 Primary endpoint analysis

The primary endpoints are change in 6-MWT and change in NT-proBNP from baseline to week 52.

Analysis addressing the primary estimand

The effect of interest in the primary estimand is regardless of premature discontinuation of study intervention. The primary analysis will be based on the FAS. The following statistical analysis and imputation method is used to address the primary estimand.

Discontinuation of randomised study intervention will be handled by a treatment policy strategy including all post-discontinuation observations. Missing values of 6-MWT and NT-proBNP (log-transformed) will be imputed (single or multiple) as described in the estimand section (Table 3-2). Subsequently, values of change from baseline to week 52 will be calculated based on the observed and imputed post-baseline values. The procedure is described in detail below:

First missing values due to death or CV hospitalisation or urgent heart failure visit will be assigned according to Table 3-2.

Thereafter, missing values due to fracture or other reason (Table 3-2) of 6-MWT or NT-proBNP (log-transformed) will be multiple imputed sequentially:

- First, intermittent missing post-baseline values are imputed separately for each intervention group using Markov Chain Monte Carlo to generate multiple (1000) copies of the dataset with monotone missing data patterns.
- Next, a stepwise procedure sequentially imputes the missing values for the remaining visits containing missing values.
- At the first visit containing missing values, models are fitted for each copy of the dataset to the observed values for:
    - Pattern 1: Placebo group
    - Pattern 2: NNC6019-0001 30 mg/kg on randomised study intervention
    - Pattern 3: NNC6019-0001 100 mg/kg on randomised study intervention

  The models will include the stratification variable as a factor and as covariates baseline 6-MWT or NT-proBNP (log-transformed) and the observed post-baseline assessments for visits prior to the one in question. The estimated parameters, and their variances, are used to impute missing post-baseline values for the visit in question. For 6-MWT, a minimum of zero will be specified to prevent imputations below zero.
    - Placebo group: Impute from pattern 1
    - NNC6019-0001 30 mg/kg group and prematurely discontinued randomised study intervention: Impute from pattern 1
    - NNC6019-0001 100 mg/kg group and prematurely discontinued randomised study intervention: Impute from pattern 1
    - NNC6019-0001 30 mg/kg on randomised study intervention: Impute from pattern 2
    - NNC6019-0001 100 mg/kg on randomised study intervention: Impute from pattern 3

  The stepwise procedure is repeated sequentially for Visits 3, Visit 5, Visit 8, and Visit 15 to impute the missing values. If no intermittent missing values exist, multiple copies of the dataset will be generated at the first visit where missing values are present.
  Values of change from baseline to Visit 15 (week 52) will be calculated based on the observed and imputed post-baseline values.
- For each of the complete data sets, change in 6-MWT or NT-proBNP (log-transformed) from baseline to Visit 15 (week 52) is analysed using an analysis of variance model with randomised study intervention (NNC6019-0001 (30 mg/kg or 100 mg/kg) vs placebo) and the stratification variable as factors and baseline 6-MWT or NT-proBNP (log-transformed) as a covariate.
- The estimates and standard deviations for the dataset copies are pooled to one estimate and associated standard deviation using Rubin's rule. For NT-proBNP, the mean difference on the logarithmic scale will be back-transformed to original scale and reported as a ratio of geometric mean ratios.

9.3.3 Secondary endpoints analysis

9.3.3.1 Supportive secondary endpoints

For details on analyses of additional supportive secondary endpoints, please refer to the SAP.

9.3.4 Exploratory endpoints analysis

For details on analyses of exploratory endpoints, please refer to the SAP.

9.3.5 Other safety analyses

All safety analyses will be made on the safety analysis set. The standard safety assessments (AEs, safety laboratory parameters, vital signs, etc.) will be reported descriptively, including any notable changes of clinical interest in laboratory parameters.

9.3.6 Other analyses

Potential analyses on additional outcomes from the cardiac MRI scans and echocardiographic parameters will be described in the SAP. For other analyses, please also refer to the SAP.

9.3.6.1 Pharmacokinetic and pharmacodynamic modelling

Population PK and exposure-response analysis based on drug concentration and response data from the study will be performed.

The objective of the population PK analysis is to evaluate the effects of pre-specified covariates on drug exposure. The objective of the exposure-response analysis is to investigate the relationship between drug exposure and response and to evaluate the effects of pre-specified covariates on this relationship. A more technical and detailed elaboration of the population PK analysis and exposure-response analysis will be given in a modelling analysis plan (MAP), which will be prepared before DBL.

The population PK and exposure-response analysis will be reported in a separate modelling report, which will not be part of the clinical study report. The individual drug concentration data will be tabulated in the bioanalytical report.

9.4 Interim analysis

An interim evaluation and a partial DBL are pre-planned but may be reconsidered during the study period. It is not considered a protocol deviation if one or more interim evaluation is not performed. An interim evaluation is planned when all participants still in the study have reached the week 24 visit based on all efficacy, safety, and PK data supporting a preliminary selection of the phase 3 dose. The purpose is to obtain advice from regulatory agencies on the overall phase 3 design and the preliminary selected dose to be investigated. The evaluation will primarily be based on the endpoints 6-MWT, NT-proBNP, ECV and KCCQ, while simultaneously considering PK data, exposure-response relationships, and the overall safety profile. No change in study design can occur as a consequence of the interim evaluation and the study will not be stopped for either positive efficacy or futility.

A minimal number of Novo Nordisk personnel in an Unblinded Interim Team will be unblinded to perform the interim analyses and interpret the results. From unblinding at the interim evaluation until database lock for the partial DBL, the Unblinded Interim Team cannot be involved in the daily study activities, including but not limited to data cleaning, medical monitoring, safety surveillance, or involved in protocol amendments, updates to existing endpoints, or definition of new endpoints and analyses. Thereafter, the Unblinded Interim Team can again be involved in the study

evaluation. To avoid inducing bias to data collected after the interim evaluation and maintain the integrity of the study, results will only be shared with regulatory authorities and kept confidential to participants, investigators and Novo Nordisk personnel who is not member of the Unblinded Interim Team.

A partial DBL is planned when all participants have completed the end of treatment visit (prior to completion of the follow-up visit for all participants) based on the available efficacy, safety and PK data to confirm the phase 3 dose selected at the interim evaluation. If the evaluation of the selected dose changes from the interim evaluation (if conducted), another dose may be selected. The evaluation will primarily be based on the statistical comparisons of 6-MWT, NT-proBNP, ECV and KCCQ specified in the protocol and the SAP, while simultaneously considering PK data, exposure-response relationships, and the overall safety profile. No change in study design can occur as a consequence of this partial DBL evaluation. The regular study team will perform the evaluation, as only follow-up data collection is ongoing.

Further information will be specified in an interim charter or SAP before unblinding.

9.5 Sample size determination

As no confirmatory hypothesis will be tested statistically, the sample size calculation is based on the precision of the comparisons of the primary endpoints.

In the ATTR-ACT[20] study (Figure 4A), a change from baseline of approximately -23 meters was observed for the pooled tafamidis group and -55 meters for the placebo group. With up to 30% of participants expected to be on tafamidis in the present study, a change from baseline in the placebo group of -23*0.3 + -55*0.7 = -45 meters can be expected. Therefore, a 95% confidence interval for the difference between NNC6019-0001 and placebo with a half-width of 45 meters is considered appropriate. With 27 participants in each intervention group and an SD of 75 there is more than 85% probability for obtaining such a confidence interval. The probability of obtaining a half-width of 45 meters on the confidence interval for 6-MWT for various sample sizes and standard deviations is shown in Table 9-1.

Based on relative change in NT-proBNP compared to placebo after 52 weeks from the studies ATTR-ACT[20] (approximate 18% reduction), and APOLLO[40] (approximate 44% reduction), a 95% confidence interval with a half-width of 30% reduction between NNC6019-0001 and placebo is considered appropriate. Assuming a coefficient of variation of approximately 0.65 observed in APOLLO[40] it requires 27 participants in each intervention group to obtain such a confidence interval with 80% probability.

Allowing for approximately 20% dropout, it is planned to randomise 99 participants 1:1:1 with 33 participants in each intervention group.

Table 9-1 Probability of obtaining a half-width of 45 meters on the confidence interval for 6-MWT for various sample sizes and standard deviations (SD)

	**Participants per intervention group allowing 20% dropout**		
SD	30	33	36
70	85%	96%	100%
75	64%	85%	96%

Abbreviations: SD = standard deviation.

10 Supporting documentation and operational considerations

10.1 Appendix 1: Regulatory, ethical, and study oversight considerations

10.1.1 Regulatory and ethical considerations

This study will be conducted in accordance with the protocol and with the following:

- Consensus ethical principles derived from international guidelines including the Declaration of Helsinki[41] and applicable ICH Good Clinical Practice (GCP) Guideline[42]
- Applicable laws and regulations

The protocol, informed consent form, investigator's brochure (as applicable) and other relevant documents (e.g., advertisements) must be submitted to an IRB/IEC and reviewed and approved by the IRB/IEC before the study is initiated.

Regulatory authorities will receive the clinical trial application, protocol amendments, reports on SAEs, and the CSR according to national requirements.

Any amendments to the protocol will require IRB/IEC approval before implementation of changes made to the study design, except for changes necessary to eliminate an immediate safety hazard to study participants.

Before a site is allowed to start screening participants, written notification from Novo Nordisk must be received.

The investigator will be responsible for:

- providing written summaries of the status of the study annually or more frequently in accordance with the requirements, policies, and procedures established by the IRB/IEC and/or regulatory authorities
- notifying the IRB/IEC of SAEs or other significant safety findings as required by IRB/IEC procedures
- providing oversight of the conduct of the study at the site and adherence to requirements of ICH guidelines, the IRB/IEC, and all other applicable local regulations
- ensuring submission of the CSR synopsis to the IRB/IEC
- reporting any potential serious breaches to the sponsor immediately after discovery

US: For country-specific requirements, please refer to Appendix 8 (Section 10.8).

10.1.2 Financial disclosure

Investigators and sub-investigators will provide Novo Nordisk with sufficient, accurate financial information as requested to allow Novo Nordisk to submit complete and accurate financial certification or disclosure statements to the appropriate regulatory authorities. Investigators are responsible for providing information on financial interests during the course of the study and one year after completion of the study.
Verification under disclosures per Code of Federal Regulations (CFR) of Financial Conflict of Interest.

10.1.3 Informed consent process

The investigator or his/her representative will explain the nature of the study, including the risks and benefits, to the participant and answer all questions regarding the study. This includes the use of an impartial witness where required according to local requirements.

The investigator must ensure the participant ample time to come to a decision whether or not to participate in the study.

Participants must be informed that their participation is voluntary. Participants will be required to sign and date a statement of informed consent that meets the requirements of local regulations, ICH GCP[42] guidelines, Declaration of Helsinki,[41] privacy and data protection requirements, where applicable, and the IRB/IEC or site.

The medical record must include a statement that written informed consent was obtained before any study-related activity and the date when the written consent was obtained. The authorised person obtaining the informed consent must also sign and date the informed consent form before any study-related activity.

The responsibility of seeking informed consent must remain with the investigator, but the investigator may delegate the task to a medically qualified person, in accordance with local requirements.

Participants must be re-consented to the most current version of the informed consent form(s) during their participation in the study.

A copy of the informed consent form(s) must be provided to the participant.

Czech Republic: For country-specific requirements, please refer to Appendix 8 (Section 10.8).

10.1.4 Information to participants during the study

The site will be offered a communication package for the participant during the conduct of the study. The package content is issued by Novo Nordisk. The communication package will contain written information intended for distribution to the participants. The written information will be translated and adjusted to local requirements and distributed to the participant at the discretion of the investigator. The participant may receive a “thank you for your participation letter” after completion of the study. Further, the participant may receive other written information during the study.

All written information to participants must be sent to IRB/IEC for approval/favourable opinion and to regulatory authorities for approval or notification according to local regulations.

10.1.5 Data protection

Participants will be assigned a 6-digit unique identifier, a subject ID. Any participant records or datasets that are transferred to Novo Nordisk will contain the identifier only. No direct identifiers from the participant are transferred to Novo Nordisk.

The participant and any biological material obtained from the participant will be identified by subject ID, visit number and study ID. Appropriate measures such as encryption or leaving out certain identifiers will be enforced to protect the identity of participants as required by local, regional and national requirements.

The participant must be informed about his/her privacy rights, including that his/her personal study-related data will be used by Novo Nordisk in accordance with local data protection law. The disclosure of the data must also be explained to the participant.

The participant must be informed that his/her medical records may be examined by auditors or other authorised personnel appointed by Novo Nordisk, by appropriate IRB/IEC members, and by inspectors from regulatory authorities.

Personal data may be collected from participants due to process requirements from Novo Nordisk's suppliers. This data is needed to ensure that the relevant data analysis for the study can be performed, but will not be part of the data transferred to Novo Nordisk, the assessment of the study endpoints or the clinical study report. A list of any such data values must be kept as part of the study documentation along with an explanation of why it was required.

Spain, UK: For country-specific requirements, please refer to Appendix 8 (Section 10.8).

10.1.6 Committee structure

10.1.6.1 Novo Nordisk safety committee

Novo Nordisk will perform ongoing safety surveillance. If new safety signals are identified, these will be evaluated by an internal safety committee. The safety committee may recommend unblinding of any data for further analysis, and in this case an internal study-independent ad hoc group may be established in order to maintain the blinding of the study personnel.

10.1.6.2 Data monitoring committee

The DMC is an independent, external committee composed of members whose expertise covers relevant specialties including statistics. The DMC is established to review and evaluate accumulated data from the study at predefined time points as well as *ad hoc*. This is done in order to protect the safety of the participants and to evaluate the benefit-risk balance. The DMC will have access to unblinded data, and will provide recommendations on study continuation, modification or termination.

Information regarding responsibilities, procedures and workflow to be used by the DMC are specified in the DMC charter.

10.1.6.3 Steering Committee

A steering committee will provide scientific and operational leadership for the study. The committee will consist of experts from outside Novo Nordisk, and designated Novo Nordisk employees. The committee will operate under a charter agreed with Novo Nordisk.

10.1.6.4 Event adjudication committee

An independent external EAC is established to perform ongoing blinded adjudication of selected AEs and deaths (see Table 8-1 and Appendix 7 [Section *10.7*]).

The EAC will evaluate events sent for adjudication using pre-defined definitions and guidelines in accordance with the EAC charter. The evaluation is based on review of pre-defined clinical data collected by the sites. The EAC is composed of permanent members covering all required medical specialities. EAC members must disclose any potential conflicts of interest and must be independent of Novo Nordisk. The EAC will have no authority to impact study conduct, study protocol or amendments. The assessments made by both the event adjudication committee and the investigator will be evaluated and included in the CSR.

10.1.7 Dissemination of clinical study data

Study information will be disclosed at clinicaltrials.gov and novonordisk-trials.com and, if applicable, also on other national or regional study registries. It will be disclosed according to applicable requirements, relevant recommendations or regulations, such as the Declaration of Helsinki,[41] the International Committee of Medical Journal Editors (ICMJE),[43] the Food and Drug Administration Amendment Act (FDAAA),[44] European Commission Requirements[45-47] and in accordance with Novo Nordisk commitment to clinical transparency. If a participant requests to be included in the study via the Novo Nordisk e-mail contact at these web sites, Novo Nordisk may disclose the investigator's contact details to the participant. As a result of increasing requirements for transparency, some countries require public disclosure of investigator names and their affiliations.

Japan: For country-specific requirements, please refer to Appendix 8 (Section 10.8).

10.1.8 Data quality assurance

10.1.8.1 Case report forms

Novo Nordisk or designee is responsible for the data management of this study including quality checking of the data.

To demonstrate his/her oversight of the collected data, the investigator should sign the eCRF on a regular basis during the conduct of the study as well as at the end of the study, as described in the eCRF completion guideline.

All participant data relating to the study will be recorded on eCRFs unless transmitted electronically to Novo Nordisk or designee (e.g., laboratory data). The investigator is responsible for verifying that data entries are accurate and correct by physically or electronically signing the CRF.

The following will be provided as paper CRFs:

- Pregnancy forms
- Technical complaint forms

The following will be provided as paper CRFs to be used when access to the CRF is revoked or the CRF is temporarily unavailable:

- AE forms
- Safety information forms

Corrections to the CRF data may be made by the investigator or the investigator's delegated staff. An audit trail will be maintained in the CRF application containing as a minimum: the old and the new data, identification of the person entering the data, date and time of the entry and reason for the correction. If corrections are made by the investigator's delegated staff after the date when the investigator signed the CRF, the CRF must be signed and dated again by the investigator.

The investigator must ensure that data is recorded in the CRF as soon as possible, preferably within 5 working days after the visit. Once data has been entered, it will be available to Novo Nordisk for data verification and validation purposes.

10.1.8.2 Monitoring

The investigator must permit study-related monitoring, audits, IRB/IEC review, and regulatory agency inspections and provide direct access to source data documents (original documents, data and records). Direct access includes permission to examine, analyse, verify and reproduce any record(s) and report(s) that are important to the evaluation of the study. If the electronic source data does not have a visible audit trail, the investigator must provide the monitor with signed and dated printouts. In addition, the relevant site staff should be available for discussions at monitoring visits and between monitoring visits (e.g., by telephone).

Study monitors will perform ongoing source data verification of critical data points to confirm that data entered into the eCRF by authorised site personnel are accurate, complete and verifiable from source documents. Study monitors will perform ongoing source data review to ensure that the study is being conducted in accordance with the current approved protocol and any other study agreements, ICH GCP[42], and all applicable regulatory requirements, evaluating the adequacy of critical processes at site for the execution of the protocol, collection of study data, to ensure that the safety and rights of participants are being protected.

Monitoring will be conducted using a risk-based approach including risk assessment, monitoring plans, centralised monitoring (remote assessment of data by Novo Nordisk) and visits to sites.

Quality tolerance limits (QTLs) will be predefined in the relevant monitoring plan to identify systematic issues that can impact participant safety and/or reliability of study results. These predefined parameters will be monitored during the study, and important deviations from the QTLs and remedial actions taken will be summarised in the clinical study report.

An unblinded monitor will visit the study site to ensure that drug handling procedures are adhered to (e.g., that the unblinded pharmacy binder and drug accountability has been completed correctly) and will reconcile trial product accountability.

10.1.8.3 Protocol compliance

Deviations from the protocol should be avoided. If deviations do occur, the investigator must inform the monitor without delay and the implications of the deviation must be reviewed and discussed.

Deviations must be documented and explained in a protocol deviation by stating the reason, date, and the action(s) taken. Some deviations, for which corrections are not possible, can be acknowledged and confirmed via edit checks in the eCRF or via listings from the study database.

10.1.9 Source documents

All data entered in the eCRF must be verifiable in source documentation other than the eCRF.

If source data is entered directly in a paper CRF, each data entry or clear series of data entries must be signed and dated separately by the study staff making the entry.

The original of the completed PROs must not be removed from the site.

Source documents provide evidence for the existence of the participant and substantiate the integrity of the data collected. Source documents are filed at the site. Any source data generated by investigator's subcontractors must be archived and accessible by the site.

Data that is transcribed into the eCRF from source documents must be consistent with the source documents, or the discrepancies must be explained. The investigator may need to request previous medical records or transfer records. Also, current medical records must be available.

It must be possible to verify participant's medical history in source documents, such as participant's medical record.

The investigator must document any attempt to obtain external medical information by noting the date(s) when information was requested, and who was contacted.

Definition of what constitutes source data can be found in a source document agreement at each site. There will only be one source document defined at any time for any data element.

10.1.10 Retention of clinical study documentation

Records and documents, including signed informed consent forms, pertaining to the conduct of this study must be retained by the investigator for 25 years after end of study unless local regulations or institutional policies require a longer retention period. No records may be destroyed during the retention period without the written approval of Novo Nordisk. No records may be transferred to another location or party without written notification to Novo Nordisk.

The investigator must be able to access his/her study documents without involving Novo Nordisk in any way. If applicable, electronic CRF (eCRF) and other participant data will be provided in an electronic readable format to the investigator before access is revoked to the systems supplied by Novo Nordisk. Site-specific CRFs and other participant data (in an electronic readable format or as paper copies or prints) must be retained by the site. A copy of all data will be stored by Novo Nordisk.

Participant's medical records must be kept for the maximum period permitted by the hospital, institution or private practice.

US: For country-specific requirements, please refer to Appendix 8 (Section 10.8).

10.1.11 Study and site closure

Novo Nordisk reserves the right to close the site or terminate the study at any time for any reason at the sole discretion of Novo Nordisk. If the study is suspended or terminated, the investigator must inform the participants promptly and ensure appropriate therapy and follow-up. The investigator and/or Novo Nordisk must also promptly inform the regulatory authorities and IRBs/IECs and provide a detailed written explanation.

Sites will be closed upon study completion. A site is considered closed when all required documents and study supplies have been collected and a site closure visit has been performed.

The investigator may initiate site closure at any time, provided there is reasonable cause and sufficient notice is given in advance of the intended termination.

Reasons for the early closure of a site by Novo Nordisk or investigator may include but are not limited to:

- failure of the investigator to comply with the protocol, the requirements of the IRB/IEC or local health authorities, Novo Nordisk procedures or GCP guidelines
- inadequate recruitment of participants by the investigator
- discontinuation of further study intervention development.

10.1.12 Responsibilities

The investigator is accountable for the conduct of the study at his/her site and must ensure adequate supervision of the conduct of the study at the site. If any tasks are delegated, the investigator must maintain a log of appropriately qualified persons to whom he/she has delegated specified study-related duties. The investigator must ensure that there is adequate and documented training for all staff participating in the conduct of the study. It is the investigator's responsibility to supervise the conduct of the study and to protect the rights, safety, and well-being of the participants.

A qualified physician, who is an investigator or a sub investigator for the study, must be responsible for all study-related medical decisions.

The investigator is responsible for filing essential documents (i.e., those documents which individually and collectively permit evaluation of the conduct of a study and the quality of the data produced) in the investigator trial master file. The documents, including the participant identification code list must be kept in a secure locked facility so that no unauthorised persons can get access to the data.

The investigator will take all necessary technical and organisational safety measures to prevent accidental or wrongful destruction, loss or deterioration of data. The investigator will prevent any unauthorised access to data or any other processing of data against applicable law. This also includes ensuring that no indirect sharing of user credentials for IT systems used in this study takes place (e.g., by not sharing IT equipment with others in a way where user credentials have the

possibility of being shared). The investigator must be able to provide the necessary information or otherwise demonstrate to Novo Nordisk that such technical and organisational safety measures have been taken.

During any period of unavailability, the investigator must delegate responsibility for medical care of participants to a specific qualified physician who will be readily available to participants during that time.

If the investigator is no longer able to fulfil the role as investigator (e.g., if he/she moves or retires), a new investigator will be appointed in consultation with Novo Nordisk.

The investigator and other site personnel must have sufficient English skills according to their assigned task(s).

10.1.13 Indemnity statement

Novo Nordisk carries product liability for its products, and liability as assumed under the special laws, acts and/or guidelines for conducting clinical studies in any country, unless others have shown negligence.

Novo Nordisk assumes no liability in the event of negligence or any other liability of the sites or investigators conducting the study or by persons for whom the said site or investigator are responsible.

Novo Nordisk accepts liability in accordance with country-specific laws, acts and guidelines. France, Spain: For any country specific indemnity requirements supplementing the above, please refer to Appendix 8 (Section 10.8).

10.1.14 Publication policy

The information obtained during the conduct of this study is considered confidential and may be used by or on behalf of Novo Nordisk for regulatory purposes as well as for the general development of the study intervention. All information supplied by Novo Nordisk in connection with this study shall remain the sole property of Novo Nordisk and is to be considered confidential information.

No confidential information shall be disclosed to others without prior written consent from Novo Nordisk. Such information shall not be used except in the performance of this study.

The information obtained during this study may be made available to other investigators who are conducting other clinical studies with the study intervention, if deemed necessary by Novo Nordisk. Provided that certain conditions are fulfilled, Novo Nordisk may grant access to information obtained during this study to researchers who require access for research projects studying the same or related diseases and/or study intervention studied in this study.

Novo Nordisk may publish on its clinical studies website a redacted CSR for this study.

One investigator will be appointed by Novo Nordisk to review and sign the CSR (signatory investigator) on behalf of all participating investigators.

10.1.14.1 Communication of results

Novo Nordisk commits to communicate and disclose results of studies regardless of outcome. Disclosure includes publication of a manuscript in a peer-reviewed scientific journal, abstract submission with a poster or oral presentation at a scientific meeting or disclosure by other means.

The results of this study will be subject to public disclosure on external web sites according to international and national regulations. Novo Nordisk reserves the right to defer the release of data until specified milestones are reached, for example when the CSR is available. This includes the right not to release the results of interim analyses, because the release of such information may influence the results of the entire study.

At the end of the study, one or more scientific publications may be prepared collaboratively by the investigator(s) and Novo Nordisk. Novo Nordisk reserves the right to postpone publication and/or communication for up to 60 days to protect intellectual property.

In all cases, the study results will be reported in an objective, accurate, balanced and complete manner, with a discussion of the strengths and limitations. In the event of any disagreement on the content of any publication, both the investigators’ and Novo Nordisk opinions will be fairly and sufficiently represented in the publication.

10.1.14.2 Authorship

Novo Nordisk will work with one or more investigator(s) and other experts who have contributed to the study concept or design, acquisition, analysis or interpretation of data to report the results in one or more publications.

Authorship of publications should be in accordance with the Recommendations for the Conduct, Reporting, Editing and Publication of Scholarly Work in Medical Journals by the International Committee of Medical Journal Editors.[48]

All authors will be provided with the relevant statistical tables, figures, and reports needed to evaluate the planned publication.

Where required by the journal, the investigator from each site will be named in an acknowledgement or in the supplementary material, as specified by the journal.

10.1.14.3 Site-specific publication(s) by investigator(s)

For a multicentre clinical study, analyses based on single-site data usually have significant statistical limitations and frequently do not provide meaningful information for healthcare professionals or participants, and therefore may not be supported by Novo Nordisk. Thus, Novo Nordisk may deny a request or ask for deferment of the publication of individual site results until the primary manuscript is accepted for publication. In line with Good Publication Practice, such individual reports should not precede the primary manuscript and should always reference the primary manuscript of the study.

10.1.14.4 Investigator access to data and review of results

As owner of the study database, Novo Nordisk has the discretion to determine who will have access to the database. Individual investigators will have their own research participants' data and will be provided with the randomisation code after results are available.

10.2 Appendix 2: Clinical laboratory tests

The tests detailed in Table 10-1 and Table 10-2 will be performed by the central laboratory unless otherwise noted.

Additional tests may be performed at any time during the study as determined necessary by the investigator or required by local regulations. Only laboratory samples specified in the protocol should be sent to the central laboratory for analysis; if additional laboratory sampling is needed, e.g., to follow up on AEs, this must be done at a local laboratory.

The central lab will communicate to the investigator abnormal values of parameters not requested in the protocol but identified by the laboratory equipment and/or their processes according to their laboratory standard operating procedures (SOPs). These data will not be transferred to the study database. The investigator should review such values for AEs and report these according to this protocol.

The investigator must review all laboratory results for concomitant illnesses and AEs.

The investigator must keep an overview, e.g., a log, of laboratory samples not handled according to the laboratory manual. In addition, the investigator must keep an overview, e.g., a log, of laboratory samples stored at site.

Human biosamples for future research will be stored as described in Appendix 6 (Section 10.6.1).

US: For country-specific requirements, please refer to Appendix 8 (Section 10.8).

Table 10-1 Protocol-required efficacy laboratory assessments

Laboratory assessments	Parameters
Cardiac biomarkers	• N-terminal pro-brain natriuretic peptide (NT-proBNP)<br>• Troponin I
Inflammatory biomarkers	• High Sensitive C-Reactive Protein (CRP)<br>• IL-6<br>• IL-8<br>• TNF-α<br>• Complement C3<br>• Complement C4
Pharmacokinetics[a]	• NNC6019-0001 plasma concentrations
Other tests	• Misfolded transthyretin[a, b] (misTTR)<br>• Retinol binding protein 4[a] (RBP4)

[a] Results from PK, RBP4 and misTTR will not be made available to investigators during study conduct; [b] misTTR or fragments thereof depending on assay availability and the analysis of misTTR will be performed by Novo Nordisk A/S.

Table 10-2 Protocol-required safety laboratory assessments

Laboratory assessments	Parameters
Haematology	• Lymphocytes<br>• Eosinophils<br>• Basophils<br>• Monocytes

Laboratory assessments	Parameters
	• Neutrophils<br>• Haematocrit<br>• Haemoglobin<br>• Erythrocytes<br>• Leukocytes<br>• Thrombocytes
Biochemistry[a]	• Alanine Aminotransferase (ALT)<br>• Alkaline phosphatase<br>• Aspartate Aminotransferase (AST)<br>• Bilirubin<br>• Creatinine<br>• Potassium<br>• Sodium<br>• Urea<br>• Gamma-Glutamyl Transferase (GGT)<br>• Creatinin Kinase<br>• Glucose<br>• Albumin<br>• Calcium
Coagulation Parameters	• INR<br>• Prothrombin Time<br>• Partiel Tromboplastin Time
Lipids	• Cholesterol<br>• High density lipoprotein (HDL) cholesterol<br>• Low density lipoprotein (LDL) cholesterol<br>• Triglycerides
Hormones	• Thyrotropin (TSH)
Serology	• HIV antibody<br>• Hepatitis B surface antigen (HBsAg)<br>• Hepatitis C virus antibody
Pregnancy Testing[b]	• Highly sensitive urine human chorionic gonadotropin (hCG) pregnancy test
Urinalysis	• Urine-Albumin to Creatinine Ratio
Other tests	• eGFR calculated by the central laboratory based on the creatinine value using the CKD-EPI equation<br>• Transthyretin (TTR)[c]<br>• In case of systemic hypersensitivity (Section 8.8.2): anti-NNC6019-0001-antibodies[c], anti-NNC6019-0001 IgE[c], total IgE, tryptase and complement 50
Antibodies[c]	• Anti-NNC6019-0001-antibodies<br>• Anti-NNC6019-0001-antibody titre
Biosamples for future research	• Whole blood for genetic analysis<br>• Serum and plasma (for analyses of circulating biomarkers)<br>• Urine spot samples (for analyses of filtered circulating biomarkers)

[a] Details of required actions and follow-up assessments for increased liver parameters including any discontinuation criteria are given in Appendix 3 (Section 10.3) (Hy's Law) and Section 7.1; [b] For women of childbearing potential, as needed, local urine testing will be standard unless serum testing is required by local regulation or IRB/IEC, see Appendix 4 (Section 10.4)[c] Results from anti-drug antibodies and TTR, will not be made available to investigators during study conduct;

10.3 Appendix 3: Adverse Events and Serious Adverse Events: Definitions and procedures for recording, evaluating, follow-up, and reporting

10.3.1 Definition of AE

An AE is any untoward medical occurrence in a clinical study participant that is temporally associated with the use of IMP, whether or not considered related to the IMP. An AE can therefore be any unfavourable and unintended sign (including an abnormal laboratory finding), symptom or disease (new or exacerbated) temporally associated with the use of an IMP.

Events to be reported as AEs:

- Any abnormal laboratory test results or safety assessments considered clinically significant in the medical and scientific judgment of the investigator, including events that have worsened from prior to the time point from which AEs are collected
- Conditions detected or diagnosed after IMP administration even though it may have been present prior to the time point from which AEs are collected
- Exacerbation/worsening of a chronic or intermittent condition including either an increase in frequency and/or intensity of the condition
- Signs, symptoms or the clinical sequelae of a suspected drug-drug interaction
- Signs, symptoms or the clinical sequelae of a suspected overdose of IMP regardless of intent

A ‘lack of efficacy’ or ‘failure of expected pharmacological action’ per se will not be reported as an AE or SAE. Such instances will be captured in the efficacy assessments. However, the signs, symptoms and/or clinical sequelae resulting from lack of efficacy will be reported as AE or SAE if they fulfil the definition.

Events NOT to be reported as AEs:

- Conditions present prior to the time point from which AEs are collected and anticipated day-to-day fluctuations of these conditions. This includes those conditions identified during screening or identified during other study procedures performed before exposure to IMP.
  Note: Conditions present or occurring prior to the time point from which AEs are collected should be recorded as concomitant illness/medical history.
- Medical or surgical procedures (e.g., endoscopy, appendectomy). The condition that leads to the procedure is the AE.
- Medical or surgical procedures not preceded by an AE or worsening of a known condition.

10.3.2 Definition of an SAE

An SAE is any untoward medical occurrence that fulfils at least one of the following criteria:

- **Results in death**
- **Is life-threatening**
  - The term ‘life-threatening’ refers to an event in which the participant was at risk of death at the time of the event. It does not refer to an event which hypothetically might have caused death, if it were more severe.
- **Requires inpatient hospitalisation or prolongation of existing hospitalisation**
  - Hospitalisation signifies that the participant has been admitted at the hospital or emergency ward for observation and/or treatment that would not have been appropriate in the

physician's office or outpatient setting. Complications that occur during hospitalisation are AEs. If a complication prolongs hospitalisation or fulfils any other seriousness criteria, the event is serious. When in doubt as to whether 'hospitalisation' occurred or was necessary, the AE should be considered serious.

- Hospitalisation for elective treatment (e.g., elective medical or surgical procedures) of a condition that was present prior to the time point from which AEs are collected, and that did not worsen, is not considered an AE.

  Note: Hospitalisations for administrative, study-related, social and convenience reasons do not constitute AEs and should therefore not be reported as AEs or SAEs. Hospital admissions for medical or surgical procedures, planned before study inclusion, are not considered AEs or SAEs

- **Results in persistent or significant disability/incapacity**
  - The term 'disability' means a substantial disruption of a person's ability to conduct normal life functions. This definition is not intended to include experience of relatively minor medical significance, such as uncomplicated headache, nausea, vomiting, diarrhoea, influenza, and accidental trauma (e.g., sprained ankle), that may interfere with or prevent everyday life functions but do not constitute a substantial disruption.
- **Is a congenital anomaly/birth defect**
- **Important medical event:**
  - Medical or scientific judgment should be exercised by the investigator in deciding whether SAE reporting is appropriate in other situations. This includes important medical events that may not be immediately life-threatening or result in death or hospitalisation but may jeopardise the participant or may require medical or surgical intervention to prevent one of the other outcomes listed in the above definition. These events should usually be considered serious and reported as SAEs using the important medical event criterion.
  - The following must be reported as an SAE using the important medical event criterion if no other seriousness criteria are applicable:
    - Suspicion of transmission of infectious agents via IMP
    - Risk of liver injury defined as alanine aminotransferase (ALT) or asparate aminotransferase (AST) >3x UNL and total bilirubin >2x UNL where no alternative aetiology exists (Hy's law)

10.3.3 Description of AEs requiring additional data collection

Adverse events requiring additional data collection (on specific event form)

An AE requiring additional data collection is an AE where Novo Nordisk has evaluated that additional data is needed in the evaluation of safety.

Hypersensitivity reactions

Hypersensitivity is defined as episodes of objectively reproducible symptoms or signs initiated by exposure to a defined stimulus at a dose tolerated by normal persons. Hypersensitivity includes:

- local reactions
- Systemic reactions, including anaphylaxis.

Anaphylaxis is defined as serious hypersensitivity reactions that is rapid in onset and may cause death.

Myocardial inflammation
Newly onset of non-infectious myocarditis.

Pro-arrhythmic risk
Cardiac rhythm disturbances, including atrial and/or ventricular tachy- and/or bradyarrhythmia will be collected to assess the pro-arrhythmic risk, including:

1. A change or worsening of current arrhythmia
2. The development of a new arrhythmia
3. The development of new/a change of worsening of the sinoatrial and atrioventricular conduction disorders.

Medication error

- A medication error is an unintended failure in the IMP treatment process that leads to, or has the potential to lead to, harm to the participant, such as:
  - administration of wrong drug.
    Note: Use of wrong DUN is not considered a medication error unless it results in administration of wrong drug.
  - wrong route of administration, such as intramuscular instead of subcutaneous
  - accidental administration of a lower or higher dose than intended (including faster or slower infusion time). The administered dose must deviate from the intended dose to an extent where clinical consequences for the study participant were likely to happen as judged by the investigator, although they did not necessarily occur.

Misuse and abuse

- Situations where the IMP is intentionally and inappropriately used not in accordance with the protocol (e.g., overdose to maximise effect)
- Persistent or sporadic, intentional excessive use of an IMP which is accompanied by harmful physical or psychological effects (e.g., overdose with the intention to cause harm)

Note: Medication error, misuse and abuse must always be reported on an AE form and a specific event form must be completed. The AE diagnosis on the AE form must reflect what occurred (e.g., accidental overdose, intentional overdose or other). If the medication error and/or misuse and abuse resulted in a clinical consequence, this must be reported on an additional AE form.

10.3.4 Recording and follow-up of AE and/or SAE

10.3.4.1 AE and SAE recording

The investigator will record all relevant AE/SAE information in the eCRF.

The investigator will attempt to establish a diagnosis of the event based on signs, symptoms, and/or other clinical information. In such cases, the diagnosis (not the individual signs/symptoms) will be documented as the AE/SAE.

When an AE/SAE occurs, it is the responsibility of the investigator to review all documentation (e.g., hospital progress notes, laboratory and diagnostics reports) related to the event.

There may be instances when copies of source documents (e.g., medical records) for certain cases are requested by Novo Nordisk. In such cases, all participant identifiers, with the exception of the subject ID, must be redacted on the copies of the source documents before submission to Novo Nordisk.

For all non-serious AEs, the applicable forms should be signed when the event is resolved or at the end of the study at the latest. For sign-off of SAE-related forms, refer to “AE and SAE reporting via paper CRF” later in this section.

Novo Nordisk products used as concomitant medication: if an AE is considered to have a causal relationship with a Novo Nordisk marketed product used as concomitant medication in the study, it is important that the suspected relationship is reported to Novo Nordisk, e.g., in the alternative aetiology section on the safety information form. Novo Nordisk may need to report this adverse event to relevant regulatory authorities

10.3.4.2 Assessment of severity

The investigator will assess severity for each event reported during the study and assign it to one of the following categories:

- **Mild**: An event that is easily tolerated by the participant, causing minimal discomfort and not interfering with everyday activities.
- **Moderate**: An event that causes sufficient discomfort and interferes with normal everyday activities.
- **Severe**: An event that prevents normal everyday activities.
  Note: An AE that is assessed as severe should not be confused with an SAE. Both AEs and SAEs can be assessed as severe.

10.3.4.3 Assessment of causality

The investigator is obligated to assess the relationship between IMP and the occurrence of each AE/SAE. The investigator will use clinical judgment to determine the relationship.

Relationship between an AE/SAE and the relevant IMP should be assessed as:

- **Probable** - Good reason and sufficient documentation to assume a causal relationship.
- **Possible** - A causal relationship is conceivable and cannot be dismissed.
- **Unlikely** - The event is most likely related to aetiology other than the IMP.

Alternative aetiology, such as underlying disease(s), concomitant medication, and other risk factors, as well as the temporal relationship of the event to IMP administration, should be considered and investigated.

The investigator should use the investigator’s brochure for the assessment. For each AE/SAE, the investigator must document in the medical records that he/she has reviewed the AE/SAE and has provided an assessment of causality.

There may be situations in which an SAE has occurred, and the investigator has minimal information to include in the initial report. However, **it is important that the investigator always**

makes an assessment of causality for every event before the initial transmission of the SAE data.

The investigator may change his/her opinion of causality, in light of follow-up information, and update the causality assessment in the eCRF.

The causality assessment is one of the criteria used when determining regulatory reporting requirements

10.3.4.4 Final outcome

The investigator will select the most appropriate outcome:

- **Recovered/resolved**: The participant has fully recovered, or by medical or surgical treatment the condition has returned to the level observed when first documented
- **Recovering/resolving**: The condition is improving, and the participant is expected to recover from the event. This term may also be applicable for AEs ongoing at the time of death (where death was due to another AE).
  Note: For SAEs, this term is only applicable if the participant has completed the follow-up period and is expected to recover.
- **Recovered/resolved with sequelae**: The participant has recovered from the condition but with lasting effect due to a disease, injury, treatment or procedure. If a sequela meets an SAE criterion, the AE must be reported as an SAE.
- **Not recovered/not resolved**: The condition of the participant has not improved, and the symptoms are unchanged, or the outcome is not known. This term may be applicable in cases of chronic conditions, cancer or AEs ongoing at time of death (where death is due to another AE).
- **Fatal**: This term is only applicable if the participant died from a condition related to the reported AE. Outcomes of other reported AEs in a participant before he/she died should be assessed as 'recovered/resolved', 'recovering/resolving', 'recovered/resolved with sequelae' or 'not recovered/not resolved'. An AE with a fatal outcome must be reported as an SAE.
- **Unknown**: This term is only applicable if the participant is lost to follow-up

10.3.4.5 Follow-up of AE and SAE

The investigator is obligated to perform or arrange for the conduct of supplemental measurements and/or evaluations as medically indicated or as requested by Novo Nordisk to elucidate the nature and/or causality of the AE or SAE as fully as possible (e.g., severe hypersensitivity reactions, Hy's law). This may include additional laboratory tests or investigations, histopathological examinations, or consultation with other health care professionals.

If a participant dies during participation in the study or during a recognised follow-up period, the investigator should if possible and upon request, provide Novo Nordisk with a redacted copy of the autopsy report including histopathology.

New or updated information should be recorded in the eCRF.

10.3.5 Reporting of SAEs

AE and SAE reporting via CRF

Relevant forms must be completed in the CRF.

For SAEs, initial notification via telephone is acceptable, although it does not replace the need for the investigator to complete the AE and safety information forms within the designated reporting timelines (see Figure 10-1):

- AE form within 24 hours.
- Safety information form within 5 calendar days.
- Both forms should be signed within 7 calendar days after first knowledge by the investigator.
- Specific event form within 14 calendar days.
- For timelines related to events for adjudication, refer also to Appendix 7 (Section 10.7).

If the eCRF is unavailable for more than 24 hours, then the sites will use the paper AE form, and if the eCRF is unavailable for more than 5 calendar days, then the site will use the paper safety information form. The site should enter the SAE data in the eCRF as soon as it becomes available.

The relevant CRF forms (AE and safety information forms) must be forwarded to Novo Nordisk in accordance with Section 10.1.5.

After the study is completed, the study database will be locked, and the CRF will be decommissioned to prevent the entry of new data or changes to existing data. If a site receives a report of a new SAE from a participant or receives updated information on a previously reported SAE after CRF decommission, the site can report this information on a paper AE and safety information form (see below) or to Novo Nordisk by telephone.

Figure 10-1 Decision tree for determining the event type and the respective forms to complete with associated timelines

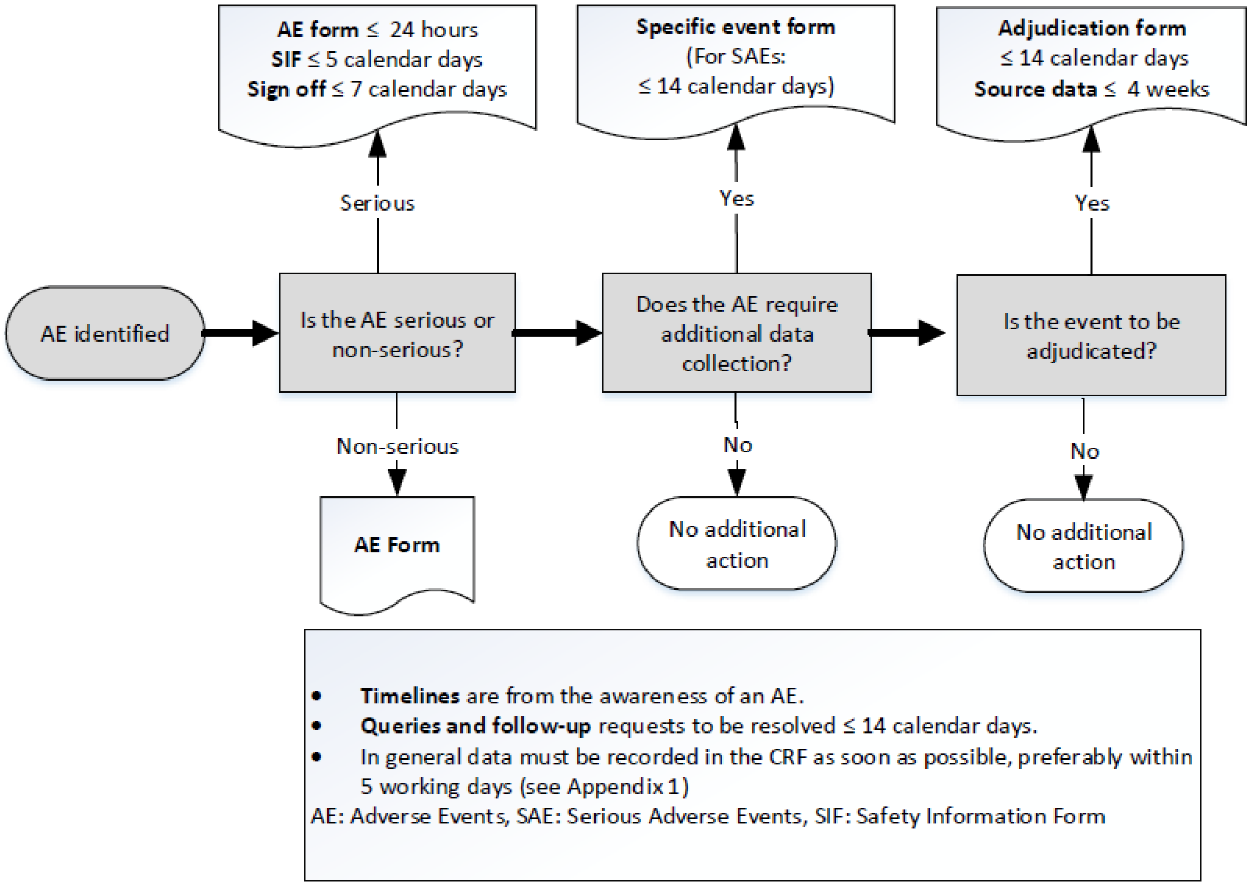

For further information on events for adjudication, refer to Appendix 7 (Section 10.7).
If the event adjudication system (EAS) is not available for document upload, the investigator should ensure that the relevant source documents are collected and saved locally until the EAS is available again.

Contact details for SAE reporting can be found in the investigator trial master file.

10.4 Appendix 4: Contraceptive guidance and collection of pregnancy information

10.4.1 Definitions

Woman of childbearing potential (WOCBP)

A woman is considered fertile following menarche and until becoming postmenopausal unless permanently sterile.
If fertility is unclear (e.g., amenorrhea in adolescents or athletes), and a menstrual cycle cannot be confirmed before first dose of study intervention, additional evaluation should be considered.
It must be recorded in the eCRF whether female participants are of childbearing potential.

Females in the following categories are not considered WOCBP

1. Premenarcheal
2. Females with one or more of the following:
   - Documented total hysterectomy
   - Documented bilateral salpingectomy
   - Documented bilateral oophorectomy

   For females with permanent infertility due to an alternate medical cause other than the above (e.g., Müllerian agenesis, androgen insensitivity), investigator discretion should be applied in determining study enrolment.
3. Postmenopausal female:
   - A postmenopausal state is defined as amenorrhoea for at least 12 months without an alternative medical cause in a female > 45 years of age. Alternative medical causes for amenorrhoea include, but are not limited to, hormonal contraception or hormonal replacement therapy.
   - Females ≥ 60 years of age can be considered postmenopausal.

Females on HRT and whose menopausal status is in doubt are considered of childbearing potential and will be required to use one of the highly effective contraception methods.

Note: Documentation regarding categories 1-3 can come from the site staff's review of participant's medical records, medical examination or medical history interview.

10.4.2 Contraceptive guidance

Male participants

No contraception measures are needed for male participants as the risk of teratogenicity/fetotoxicity caused by transfer of NNC6019-0001 in seminal fluid is unlikely.

Female participants

Female participants of childbearing potential are eligible to participate if they agree to use methods of contraception consistently and correctly. Table 10-3 lists the highly effective methods of contraception allowed. Local regulations may apply.

UK, Portugal, Spain: For country-specific requirements, please refer to Appendix 8 (Section *10.8*).

Highly effective contraception should be utilised for a least 16 weeks after last dose of IMP (corresponding to time during treatment and until the end of relevant systemic exposure).

Table 10-3 Highly effective contraceptive methods allowed[49]

**Highly effective methods[a] (Failure rate of <1% per year when used consistently and correctly):**<br>• Combined (estrogen- and progestogen-containing) hormonal contraception associated with inhibition of ovulation[b]<br>• oral<br>• intravaginal<br>• transdermal<br>• Progestogen-only hormone contraception associated with inhibition of ovulation<br>• oral<br>• injectable<br>• implantable<br>• Intrauterine device (IUD)<br>• Intrauterine hormone-releasing system (IUS)<br>• Bilateral tubal occlusion<br>• Vasectomized partner<br>Vasectomized partner is a highly effective contraceptive method provided that the partner is the sole sexual partner of the woman of childbearing potential, and the absence of sperm has been confirmed. If not, an additional highly effective method of contraception should be used. Spermatogenesis cycle is approximately 90 days.<br>• Sexual abstinence<br>Sexual abstinence is considered a highly effective method only if defined as refraining from heterosexual intercourse during the entire period of risk associated with the study intervention. The reliability of sexual abstinence needs to be evaluated in relation to the duration of the study and the preferred and usual lifestyle of the participant.

[a]Contraceptive use by men or women should comply with local regulations regarding the use of contraceptive methods for those participating in clinical studies; [b]If locally required, in accordance with Clinical Trial Facilitation Group (CTFG) guidelines, acceptable contraceptive methods are limited to those which inhibit ovulation as the primary mode of action.

The following methods are not acceptable methods of contraception: Periodic abstinence (calendar, symptothermal, post-ovulation methods), withdrawal (coitus interruptus), spermicides only, and lactational amenorrhoea method (LAM).

10.4.3 Collection of pregnancy information

Female participants who become pregnant

Investigator will collect pregnancy information on any female participant who becomes pregnant while participating in this study.

Information will be recorded on the appropriate form and submitted to Novo Nordisk within 14 calendar days of learning of a participant's pregnancy (see Figure 10-2).

The participant will be followed to determine the outcome of the pregnancy. The investigator will collect follow-up information on participant and neonate which will be forwarded to Novo Nordisk within 14 calendar days. Generally, follow-up will not be required for longer than 1 month beyond the delivery date.

Any termination of pregnancy will be reported, regardless of foetal status (presence or absence of anomalies) or indication for procedure.

While pregnancy itself is not considered to be an AE or SAE, any adverse event in connection with pregnancy or elective termination of a pregnancy for medical reasons will be reported as an AE or SAE. If relevant, consider adding 'gestational', 'pregnancy-related' or a similar term when reporting the AE/SAE.

Pregnancy outcome should be documented in the participant's medical record. Abnormal pregnancy outcome (e.g., spontaneous abortion, foetal death, stillbirth, congenital anomalies and ectopic pregnancy) is considered an SAE. In case of abnormal pregnancy outcome, paternal information should be recorded in the appropriate form after obtaining the necessary signed paternal informed consent.

If the investigator learns of an SAE occurring as a result of a post-study pregnancy which is considered related to the IMP by the investigator, the SAE should be reported to Novo Nordisk as described in Appendix 3 (Section 10.3.)

Figure 10-2 Decision tree for determining the forms to complete for collection of pregnancy information and timelines for reporting – For female participants

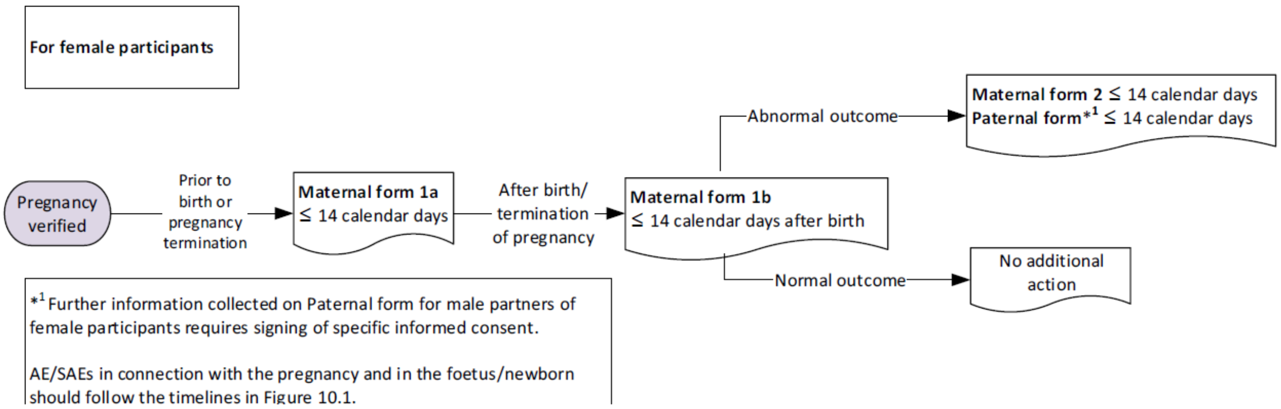

Any female participant who becomes pregnant while participating in the study will discontinue study intervention. Furthermore, study intervention must be discontinued if the female participant intends to become pregnant.

10.5 Appendix 5: Technical complaints: Definition and procedures for recording, evaluation, follow-up and reporting

10.5.1 Definition of technical complaint

A technical complaint is any written, electronic or oral communication that alleges product (medicine or device) defects. The technical complaint may be associated with an AE but does not concern the AE itself.

Examples of technical complaints:

- Problems with the physical or chemical appearance of study interventions (e.g., discoloration, particles or contamination).
- Problems with packaging material including labelling.

Time period for detecting technical complaints

All technical complaints which occur from the time of receipt of the product at site until the time of the last usage of the product must be collected for products predefined on the technical complaint form.

10.5.2 Recording and follow-up of technical complaints

Reporting of technical complaints to Novo Nordisk

For contact details for Customer Complaint Center, please refer to Attachment I.

Technical complaints on products allocated to a participant must be reported on a separate technical complaint form:

1. For products with DUN: One technical complaint form must be completed for each affected DUN.
2. For products without DUN: One technical complaint form must be completed for each batch, code or lot number.

DUN is the same as the term Kit IDs in the systems and manuals.

Timelines for reporting technical complaints to Novo Nordisk

The investigator or the investigator´s unblinded designee must complete the technical complaint form and ensure it is forwarded to Customer Complaint Center, Novo Nordisk, within:

- 24 hours if related to an SAE
- 5 days calendar for all other technical complaints

Follow-up of technical complaints

The investigator is responsible for ensuring that new or updated information will be recorded on the originally completed form.

Collection, storage and shipment of technical complaint samples

The unblinded personnel must collect the technical complaint sample and all associated parts and notify the unblinded monitor within 5 calendar days of obtaining the sample at site. The sample and

all associated parts must be sent as soon as possible to Customer Complaint Center, Novo Nordisk, together with a copy of the completed technical complaint form.

The technical complaint sample should contain the batch, code or lot number and, if available, the DUN (DUN is the same as the term Kit IDs in the systems and manuals). If the technical complaint sample is unobtainable, the reason must be stated on the technical compliant form.

If several samples are shipped in one shipment, the sample and the corresponding technical complaint form should be kept together.

Storage for the technical complaint sample must be done in accordance with the conditions prescribed for the trial product.

10.5.3 Reporting of technical complaints for products not included in the technical complaint form

Technical complaints on products not included in the technical complaint form should be reported to manufacturing holder.

10.6 Appendix 6: Retention of human biosamples for future research

Human biosamples (also in some cases known as human biospecimen or human biological materials) are samples that have been taken from the human body during life or after death. It includes:

- Primary cells, tissues, organs or cell containing fluids of human origin (for example, whole blood, urine, saliva, synovial fluid)
- Cell free fluids of primary human origin (for example, serum and plasma)
- Extracts or derivatives of the above, when derived by purification (for example, DNA, RNA, proteins, membranes, microsomes and other cellular substructures).

10.6.1 Biosamples for future research

Participants who do not wish to contribute with biosamples for storage may still participate in the study. Participants must sign and date a separate informed consent form before biosamples are collected to be stored for future analysis.

In countries where allowed, the study will involve collection of human biosamples for future research to be stored in a central laboratory facility for future use. Serum, plasma, whole blood and urine will be stored, and timing of sampling and amount of material to be stored are specified in Table 10-4.

Table 10-4 Type of material, timing of sampling and amount of material to be stored for future research

Type of material	Time of sampling[a]	Material to be stored
Serum[b]	Randomisation, V3, V5, V8, V15	5 x 0.5 mL aliquots per sample
Plasma[b]	Randomisation, V3, V5, V8, V15	5 x 0.5 mL aliquots per sample
Whole blood[c]	Randomisation, V15	1 x 2.5 mL aliquot per sample
Urine spot samples[d]	Randomisation, V15	2 x 1 mL aliquot per sample

[a] Timing of sampling is also specified in the flowchart (Section 1.2) and Appendix 2 (Section10.2).
[b] Sampled for non-genetic analyses (e.g. hormones, metabolites or similar)
[c] Whole blood collected for analysis of DNA and RNA
[d] For assessment of biomarkers or similar accumulating in urine.
Abbreviations: V = visit

The biosamples will be stored at a central laboratory, at a central storage facility or an analysing laboratory contracted by Novo Nordisk for up to 15 years after end of study. Only relevant Novo Nordisk, consultants, auditors, research organisations or laboratories working for or collaborating with Novo Nordisk as well as storage facility employees will be able to access the stored biosamples and associated data. The biosamples may be transferred to other countries for analysis and will be destroyed at the latest 15 years after end of study.

The analyses of the biosamples for future research are not intended to identify participant-specific findings, but to understand and predict response to NNC6019-0001 and related conditions on a population level.

Analysis will be done on the biosamples and associated data (data relating to the test results or results from the main study).

Novo Nordisk will ensure that third party collaborators live up to the regulations on data protection, see Appendix 1 (Section 10.1.5).

The participant may request the stored biosamples for future research to be destroyed by withdrawing the designated informed consent at any timepoint during and after the study. For samples that have already been analysed, the results can still be used for scientific research and will not be removed from the datafile.

10.6.2 Anti-NNC6019-0001-antibodies samples

Remaining and residual antibody samples (see Section 8.8.1) already collected may be retained after end of study.

- The samples will be stored at Novo Nordisk or a biorepository assigned by Novo Nordisk after end of study and until marketing authorisation approval or until the research project terminates, but no longer than 15 years from the end of study after which they will be destroyed.
- Only relevant Novo Nordisk staff and consultants, auditors, research organisations or laboratories working for Novo Nordisk and biorepository personnel will have access to the stored samples and associated data.
- The samples may be transferred to other countries for analysis, if not prohibited by local regulations, and will be destroyed at the latest 15 years after end of study.
- The identity of study participants will remain confidential, and the samples will be identified only by subject ID, visit number and study identification number. No direct identification of the participant will be stored together with the samples.

The retained samples may be used to:

- Evaluate safety or efficacy aspects that address concerns arising during or after the study.
- Further characterise the antibody responses towards the drug, if required by health authorities or for safety reasons.
- Conduct further analytical method development and validation of antibody assays.
- Genetic analyses will not be performed on these samples.

10.6.3 Hypersensitivity reaction samples

In order to comply with any future requests from health authorities to further characterise the antibody response, antibody samples collected in relation to suspicion of a severe immediate systemic hypersensitivity reaction[50] (Section 8.8.2) may be retained.

The samples will be stored at Novo Nordisk or a Novo Nordisk designated referral central biorepository. The samples might be transferred to other countries, if not prohibited by local regulations. Only Novo Nordisk staff and bio-repository personnel will have access to the stored samples. The samples may be shipped to a contract research organisation (CRO) for analysis.

The samples will be anonymised (identified only by subject ID, visit number, study identification number and sampling date). Confidentiality and personal data protection will be ensured during storage after the end of study and no direct identification of the participant will be stored together with the samples.

Potential further analyses of the samples will not have any consequences for the participant and their relatives. Participants can contact the investigator if they wish to be informed about results derived from stored antibody samples obtained from their own body.

The samples will be stored after end of study and until marketing authorisation approval or until the research project terminates, but no longer than 15 years from end of study after which they will be destroyed.

10.6.4 Pharmacokinetic and pharmacodynamic samples

Residual plasma samples used for bioanalysis of NNC6019-0001 as well as for misfolded TTR biomarker analysis may be stored at Novo Nordisk after finalisation of the CSR. The residual PK samples will only be used for potential NNC6019-0001 assay investigation/validation and exploratory metabolite analysis, if deemed relevant for interpretation of the results within this study. Residual biomarker samples for misTTR analysis may be used for potential further characterisation of biomarkers and assay investigations. Samples will be stored for up to 5 years from end of study, after which the samples will be destroyed. Only Novo Nordisk A/S or relevant special laboratories will have access to the samples.

The samples will be pseudonymized (identified only by a unique sample ID, visit number, study identification number and sampling date). Confidentiality and personal data protection will be ensured during storage after the end of study and no direct identification of the participant will be stored together with the samples.

If an assay investigation or exploratory metabolite investigation is performed using residual PK, and/or biomarker samples, the data derived from this investigation will be documented independently from the CSR.

10.7 Appendix 7: Events requiring adjudication

Event adjudication will be performed in randomised participants. An event for adjudication is a selected AE or death evaluated by an independent external Event adjudication committee (EAC) in a blinded manner, please refer to Table 10-5 for event types in scope.

For details on the EAC, refer to Appendix 1 (Section 10.1.6.4).

Table 10-5 AEs requiring event adjudication

Event type<br>(serious and non-serious AEs)	Description
Death	All cause death
Cardiovascular hospitalisation	All cause hospitalisation
Urgent heart failure visit not requiring hospitalisation	New episode or worsening of existing heart failure leading to an urgent, unscheduled clinic/office/emergency department visit

There are five ways to identify events relevant for adjudication as described below:

1. Investigator-reported events for adjudication: investigator selects the appropriate event type relevant for adjudication (see Table 10-5).
2. AEs reported with fatal outcome
3. AEs reported as requiring hospitalisation
4. AE search (standardised screening): All AEs not reported with an event type relevant for adjudication will undergo screening to identify potential urgent heart failure visit not requiring hospitalisation events for adjudication. Investigators will be notified of these events in the eCRF.
5. EAC-identified events: Unreported events relevant for adjudication identified by the EAC during review of source documents provided for another event for adjudication. Investigators will be notified of these events in the eCRF and has the option to report the EAC-identified event.

For each event relevant for adjudication, an event type specific adjudication form should be completed in the eCRF within 14 days (Figure 10-1).

Copies of source documents should be uploaded to the event adjudication system (EAS) as soon as possible and preferably within 4 weeks (Figure 10-1). In cases where the EAS is not accessible for document upload, the investigator should ensure that the relevant source documents are collected and saved locally until the EAS is available. If no, or insufficient source documents are provided to the adjudication supplier, the investigator can be asked to complete a clinical narrative to be uploaded to the EAS.

If new information becomes available for an event sent for adjudication, it is the responsibility of the investigator to ensure the new information is uploaded to the EAS.

An Event Adjudication Site Manual will be provided to each site detailing which source documents are relevant and how these should be provided to the adjudication supplier. The anonymisation and labelling requirements are also described in the event adjudication site manual.

10.8 Appendix 8: Country-specific requirements

Czech Republic:

- Appendix 1 (Section 10.1.3). Informed consent process: Participant's electronic signature is not permitted.

France:

- Sections 1.2 and 8.1: Race and ethnic origin can only be collected if purpose of the research is justified. Therefore, in this trial for the central laboratory calculation of the eGFR, information on race (black/white/other) and year of birth will be collected on the laboratory requisition form only.
- Sections 1.2 and 8.1. Date of birth: Only year is collected for the date of birth.
- Appendix 1 (Section 10.1.13). Indemnity statement: The French Public Health Code article L 1121-10 (law n° 2004-806 of 9 August 2004 art. 88 I,IX, Journal Official of 11 August 2004. "The sponsor is responsible for identification of the harmful consequences of the biomedical the research for the person lending himself thereto and for indemnification of his beneficiaries, except in case of proof, incumbent on it, that the prejudice is not attributable to his fault of the fault of any intervening party, without the sponsor's being entitled to call on acts by a third party or the voluntary withdrawal of the person who had initially consented to cooperating in the research".

Germany:

- Sections 1.2 and 8.1, Participant's full date of birth is not allowed to be collected and must be shortened to year of birth.

Japan:

- Section 6.3.1: Stratification by disease type will not be applied for Japanese participants.
- Sections 6.1 and 8.3.6: Additional safety measures will be applied for Japanese participants:
  - Japanese participants will be dosed after the safety confirmation based on unblinded data evaluation by the external, independent Data Monitoring Committee (DMC) for at least 9 participants completing the 28-days sentinel phase. In addition, intensified safety monitoring will be instituted for the first 6 randomised Japanese participants as outlined below:
  - The first 6 randomised Japanese participants will be observed on site under continuous cardiac monitoring for at least 24 hours after start of their first dose. On discharge, participants will be monitored using continuous cardiac monitoring on an out-patient basis until 7 days after start of the infusion. In addition, the first 6 randomised Japanese participants will have weekly visits with safety assessment as defined in the flowchart for Visit 2A, 2B, 2C and 2D during the first 28 days. Thereafter, the first 6 randomised Japanese participants will continue to be dosed Q4W for the full duration of the study.
  - Throughout the study, a comprehensive, risk-based medical monitoring will evaluate blinded safety data on a regular basis. In addition, a DMC will review and evaluate accumulated unblinded data (including the Japanese participants) at predefined time intervals as well as ad hoc. The DMC will continue to monitor the trial based on unblinded data until trial closure and will give their recommendation on trial continuation, modification, or termination.

- Section 6.2: Preparation/Handling/Storage/Accountability: The head of the study site or the trial product storage manager assigned by the head of the study site (a pharmacist in principle) is responsible for control and accountability of the trial products.
- Appendix 1 (Section 10.1.7). Dissemination of clinical study data: The study will be registered at www.jrct.niph.go.jp.

Netherlands:

- Sections 1.2 and 8.1: Date of birth: participant's full day of birth is not allowed to be collected as part of demography and must be shortened to year of birth.

Portugal:

- Appendix 4 (Section 10.4). Contraception requirements based on the Recommendations related to contraception and pregnancy testing in clinical trials from Clinical Trial Facilitation Group (CTFG).

Spain:

- Appendix 1 (Section 10.1.5). Data protection: This study will be conducted in line with European Regulation (EU) 2016/679 of the European Parliament and of the Council of 27 April 2016 on data protection GDPR.
- Appendix 1 (Section 10.1.13). Indemnity statement: Novo Nordisk accepts liability in accordance with Article 10 "Liability regime" of the Royal Decree 1090/2015 of 4 December.
- Appendix 4 (Section 10.4) and Section 5.2. Contraception requirements based on the Recommendations related to contraception and pregnancy testing in clinical trials from Clinical Trial Facilitation Group (CTFG).

United Kingdom:

- Appendix 1 (Section 10.1.5). Data protection: "The participant must be informed that his/her medical records may be examined by auditors or other authorised personnel appointed by Novo Nordisk, by appropriate IRB/IEC members, and by inspectors from regulatory authorities." In the UK the IRB/IEC do not have access to the participants' medical records.
- Appendix 4 (Section 10.4) and Section 5.2. Contraceptive guidance and collection of pregnancy information: Comply with the EU CTFG guidelines. When highly effective contraception is required the use of double barrier methods is not allowed under any circumstance.

United States:

- Appendix 1 (Section 10.1.1). Regulatory, ethical, and study oversight considerations: FDA form 1572:
  For US sites:
  - Intended for US sites
  - Conducted under the IND
  - All US investigators, as described above, will sign FDA Form 1572

  For sites outside the US:
  - Intended for participating sites outside of the US
  - Not conducted under the IND
  - All investigators outside of the US will not sign FDA form 1572

Novo Nordisk will analyse and report data from all sites together if more than one site is involved in the trial.

- Appendix 1 (Section 10.1.10). Retention of clinical study documentation: In the United States, 21 CFR 312.62(c) and 21 CFR 812.140(d) require 2 years following the date a marketing application is approved for the drug for the indication for which it is being investigated; or, if no application is to be filed or if the application is not approved for such indication, until 2 years after the investigation is discontinued and FDA is notified'.
- Appendix 2 (Section 10.2): Clinical laboratory tests: For haematology samples (differential count) where the test result is not normal, then a part of the sample may be kept for up to two years or according to local regulations.

10.9 Abbreviations

ADA	anti-drug antibodies
AE	adverse event
ALT	alanine aminotransferase
AST	aspartate aminotransferase
ATTR	transthyretin amyloid
AV	atrioventricular
COA	clinical outcome assessment
COVID 19	Corona virus disease 2019
CM	cardiomyopathy
CRF	case report form
CSR	clinical study report
CTFG	clinical trial facilitation group
CV	cardiovascular
DBL	database lock
DMC	Data Monitoring Committee
DNA	deoxyribonucleic acid
DPD	3,3-diphosphono-1,2-propanodicarboxylic acid
DPS	data points set
DUN	dispensing unit number
EAC	Event Adjudication Committee
EAS	event adjudication system
ECG	electrocardiogram
eCRF	electronic case report form
ECV	extracellular volume
eGFR	estimated glomerular filtration rate
FAS	full analysis set
FDA	U.S. Food and Drug Administration
FDAAA	FDA Amendments Act
FHD	first human dose
GCP	Good Clinical Practice
GLS	global longitudinal strain
hATTR	hereditary ATTR
HFpEF	heart failure with preserved ejection fraction
HIV	Human Immunodeficiency Virus
HMDP	hydroxymethylene diphosphonate

HR	hazard ratio
HRT	hormone replacement therapy
IB	investigator’s brochure
ICH	International Council for Harmonisation
IEC	independent ethics committee
IMP	investigational medicinal product
IND	investigational new drug
INR	international normalised ratio
IRB	institutional review board
KCCQ	Change in Kansas City Cardiomyopathy Questionnaire
LPLV	last participant last visit
MAP	modelling analysis plan
MR	magnetic resonance
MRI	magnetic resonance imaging
6-MWT	6 Minute Walk Test
NIMP	non-investigational medicinal product
NIS	neuropathy impairment score
NSF	nephrogenic systemic fibrosis
NT-proBNP	N-terminal-pro brain natriuretic peptide
NYHA	New York Heart Association
PAS	participant analysis set
PCD	primary completion date
PD	pharmacodynamics
PGI-S	Patient Global Impression of Status
PIN	prostatic intraepithelial neoplasia
PK	pharmacokinetics
PN	polyneuropathy
PRO	patient reported outcome
PYP	pyrophosphate
RNA	ribonucleic acid
RR	relative risk
RTSM/IWRS	randomisation and trial supplies management system / interactive web response system
SAE	serious adverse event
SAP	statistical analysis plan
SPECT	single-photon emission computed tomography
SUSAR	suspected unexpected serious adverse reaction
TEAE	treatment emergent adverse event

TMM	Trial Materials Manual
TTR	transthyretin
VAS	visual analogue scale
WOCBP	woman of childbearing potential
wtATTR	wild-type ATTR

11 References

1. Ruberg FL, Grogan M, Hanna M, Kelly JW, Maurer MS. Transthyretin Amyloid Cardiomyopathy: JACC State-of-the-Art Review. J Am Coll Cardiol. 2019;73(22):2872-91.
2. Spertus JA, Jones PG, Sandhu AT, Arnold SV. Interpreting the Kansas City Cardiomyopathy Questionnaire in Clinical Trials and Clinical Care: JACC State-of-the-Art Review. J Am Coll Cardiol. 2020;76(20):2379-90.
3. Hawkins PN, Ando Y, Dispenzeri A, Gonzalez-Duarte A, Adams D, Suhr OB. Evolving landscape in the management of transthyretin amyloidosis. Ann Med. 2015;47(8):625-38.
4. Emdin M, Aimo A, Rapezzi C, Fontana M, Perfetto F, Seferović PM, et al. Treatment of cardiac transthyretin amyloidosis: an update. Eur Heart J. 2019;40(45):3699-706.
5. Garcia-Pavia P, Rapezzi C, Adler Y, Arad M, Basso C, Brucato A, et al. Diagnosis and treatment of cardiac amyloidosis: a position statement of the ESC Working Group on Myocardial and Pericardial Diseases. Eur Heart J. 2021;42(16):1554-68.
6. Lauppe RE, Liseth Hansen J, Gerdesköld C, Rozenbaum MH, Strand AM, Vakevainen M, et al. Nationwide prevalence and characteristics of transthyretin amyloid cardiomyopathy in Sweden. Open Heart. 2021;8(2).
7. Damy T, Bourel G, Slama M, de Neuville B, Rault C, Charrong P. PCV67 Epidemiology of Transthyretin Amyloid Cardiomyopathy (ATTR-CM) in France: EPACT, a Study Based on the French Nationwide Claims Database Snds. Value in Health. 2020;23:S498-9.
8. Winburn I, Ishii T, Sumikawa T, Togo K, Yasunaga H. Estimating the Prevalence of Transthyretin Amyloid Cardiomyopathy in a Large In-Hospital Database in Japan. Cardiol Ther. 2019;8(2):297-316.
9. Auer-Grumbach M, Rettl R, Ablasser K, Agis H, Beetz C, Duca F, et al. Hereditary ATTR Amyloidosis in Austria: Prevalence and Epidemiological Hot Spots. J Clin Med. 2020;9(7).
10. Lindmark K, Pilebro B, Sundström T, Lindqvist P. Prevalence of wild type transtyrethin cardiac amyloidosis in a heart failure clinic. ESC Heart Fail. 2021;8(1):745-9.
11. Lane T, Fontana M, Martinez-Naharro A, Quarta CC, Whelan CJ, Petrie A, et al. Natural History, Quality of Life, and Outcome in Cardiac Transthyretin Amyloidosis. Circulation. 2019;140(1):16-26.
12. Maurer MS, Bokhari S, Damy T, Dorbala S, Drachman BM, Fontana M, et al. Expert Consensus Recommendations for the Suspicion and Diagnosis of Transthyretin Cardiac Amyloidosis. Circ Heart Fail. 2019;12(9):e006075.
13. Yamamoto H, Yokochi T. Transthyretin cardiac amyloidosis: an update on diagnosis and treatment. ESC Heart Fail. 2019;6(6):1128-39.
14. Pfizer. Vyndaqel® (tafamidis), US Prescribing Information (PI). June 2021.

15. Pfizer. Vyndaqel ® (tafamidis), EU Summary of product characteristics (SmPC). 2019.
16. Ionis Pharmaceuticals. Tegsedi™ (inotersen), US prescribing information (PI). Oct 2018.
17. Ionis Pharmaceutical. Tegsedi™(inotersen), EU summary of product characteristics (SmPC). 2018.
18. Alnylam Pharmaceuticals Inc. Onpattro™ (patisiran), US prescribing information (PI). Aug 2018.
19. Alnylam Pharmaceuticals Inc. Onpattro™ (patisiran), EU Summary of product characteristics (SmPC). 2018.
20. Maurer MS, Schwartz JH, Gundapaneni B, Elliott PM, Merlini G, Waddington-Cruz M, et al. Tafamidis Treatment for Patients with Transthyretin Amyloid Cardiomyopathy. N Engl J Med. 2018;379(11):1007-16.
21. Prothena Biosciences Limited. Clinical trial report (PRX004-101). A phase 1, open-label, dose escalation study of intravenous PRX004 in subjects with amyloid transthyretin (ATTR) amyloidosis. 18 March 2021.
22. Merlini G, Bellotti V. Molecular mechanisms of amyloidosis. N Engl J Med. 2003;349(6):583-96.
23. Benson MD, Kincaid JC. The molecular biology and clinical features of amyloid neuropathy. Muscle Nerve. 2007;36(4):411-23.
24. Coelho T, Maurer MS, Suhr OB. THAOS - The Transthyretin Amyloidosis Outcomes Survey: initial report on clinical manifestations in patients with hereditary and wild-type transthyretin amyloidosis. Curr Med Res Opin. 2013;29(1):63-76.
25. Lobato L. Portuguese-type amyloidosis (transthyretin amyloidosis, ATTR V30M). J Nephrol. 2003;16(3):438-42.
26. Lobato L, Rocha A. Transthyretin amyloidosis and the kidney. Clin J Am Soc Nephrol. 2012;7(8):1337-46.
27. Hofmann C, Katus HA, Doroudgar S. Protein Misfolding in Cardiac Disease. Circulation. 2019;139(18):2085-8.
28. Novo Nordisk A/S. Investigator's Brochure, NNC6019-0001, project NN6019, (edition 4). 31 Jan 2022.
29. European Medicines Agency. ICH E9 (R1) addendum on estimands and sensitivity analysis in clinical trials to the guideline on statistical principles for clinical trials. Step 5 (EMA/CHMP/ICH/436221/2017). 17 Feb 2020.
30. American College of Radiology. Nephrogenic systemic fibrosis. ACR Manual On Contrast Media: ACR Committee on Drugs and Contrast Media; 2021. p. 80-8.
31. Oghina S, Josse C, Bézard M, Kharoubi M, Delbarre MA, Eyharts D, et al. Prognostic Value of N-Terminal Pro-Brain Natriuretic Peptide and High-Sensitivity Troponin T Levels in the Natural History of Transthyretin Amyloid

Cardiomyopathy and Their Evolution after Tafamidis Treatment. J Clin Med. 2021;10(21).

32. Uszko-Lencer NHMK, Mesquita R, Janssen E, Werter C, Brunner-La Rocca HP, Pitta F, et al. Reliability, construct validity and determinants of 6-minute walk test performance in patients with chronic heart failure. Int J Cardiol. 2017;240:285-90.
33. Law S, Petrie A, Chacko L, Cohen OC, Ravichandran S, Gilbertson JA, et al. Change in N-terminal pro-B-type natriuretic peptide at 1 year predicts mortality in wild-type transthyretin amyloid cardiomyopathy. Heart. 2021.
34. Fontana M, Ćorović A, Scully P, Moon JC. Myocardial Amyloidosis: The Exemplar Interstitial Disease. JACC Cardiovasc Imaging. 2019;12(11 Pt 2):2345-56.
35. Prothena Biosciences. PK/PD report (PRX001-101): A phase 1, open-label, dose escalation study of intravenous PRX004 in subjects with amyloid transthyretin (ATTR) amyloidosis: Pharmacokinetic, phamacodynamic, immunogenicity report. 13 Jan 2021.
36. European Commission. The Rules Governing Medicinal Products in the European Union, Volume 4, Annex 13, Investigational Medicinal Products (ENTR/F/2/AM/an D(2010) 3374). 03 Feb 2010.
37. Hanna M, Ruberg FL, Maurer MS, Dispenzieri A, Dorbala S, Falk RH, et al. Cardiac Scintigraphy With Technetium-99m-Labeled Bone-Seeking Tracers for Suspected Amyloidosis: JACC Review Topic of the Week. J Am Coll Cardiol. 2020;75(22):2851-62.
38. Arvanitis M, Koch CM, Chan GG, Torres-Arancivia C, LaValley MP, Jacobson DR, et al. Identification of Transthyretin Cardiac Amyloidosis Using Serum Retinol-Binding Protein 4 and a Clinical Prediction Model. JAMA Cardiol. 2017;2(3):305-13.
39. Jiang X, Labaudinière R, Buxbaum JN, Monteiro C, Novais M, Coelho T, et al. A circulating, disease-specific, mechanism-linked biomarker for ATTR polyneuropathy diagnosis and response to therapy prediction. Proc Natl Acad Sci U S A. 2021;118(9).
40. Solomon SD, Adams D, Kristen A, Grogan M, González-Duarte A, Maurer MS, et al. Effects of Patisiran, an RNA Interference Therapeutic, on Cardiac Parameters in Patients With Hereditary Transthyretin-Mediated Amyloidosis. Circulation. 2019;139(4):431-43.
41. World Medical Association. WMA Declaration of Helsinki - Ethical Principles for Medical Research Involving Human Subjects. Last amended by the 64th WMA General Assembly, Fortaleza, Brazil. Oct 2013.
42. ICH Harmonised Tripartite Guideline. Guideline for Good Clinical Practice E6(R2), Current step 4 version. 09 Nov 2016.
43. De Angelis C, Drazen JM, Frizelle FA, Haug C, Hoey J, Horton R, et al. Clinical trial registration: a statement from the International Committee of Medical Journal Editors. N Engl J Med. 2004;351(12):1250-1.

44. U.S. Department of Health and Human Services, Food and Drug Administration. Food and Drug Administration Amendments Act of 2007 as amended by the Final Rule "Clinical Trials Registration and Results Information Submission". 21 September 2016.
45. The European Parliament and the Council of the European Council. Directive 2001/20/EC of the European Parliament and of the Council of 4 April 2001 on the approximation of the laws, regulations and administrative provisions of the member states relating to the implementation of good clinical practice in the conduct of clinical trials on medicinal products for human use. 2001.
46. The European Parliament and the Council of the European Council. Regulation (EC) No 726/2004 of the European Parliament and of the Council of 31 March 2004 laying down Community procedures for the authorisation and supervision of medicinal products for human and veterinary use and establishing a European Medicines Agency, article 57. 30 April 2004.
47. The European Parliament and the Council of the European Council. Regulation (EC) No 1901/2006 of the European Parliament and of the Council of 12 December 2006 on medicinal products for paediatric use and amending Regulation (EEC) No 1768/92, Directive 2001/20/EC, Directive 2001/83/EC and Regulation (EC) No 726/2004, article 41. Official Journal of the European Communities. 27 Dec 2006.
48. International Committee of Medical Journal Editors. Recommendations for the Conduct, Reporting, Editing and Publication of Scholarly Work in Medical Journals; current version available at www.icmje.org.
49. Clinical Trial Facilitation Group (CTFG), Heads of Medicines Agency. Recommendations related to contraception and pregnancy testing in clinical trials. 21 Sep 2020.
50. Food and Drug Administration. Guidance for Industry:Immunogenicity Assessment for Therapeutic Protein Products. 8/2015 2015.

Protocol

Protocol Title: Efficacy and safety of NNC6019-0001 at two dose levels in participants with transthyretin amyloid cardiomyopathy (ATTR CM)

Substance name: NNC6019-0001

Universal Trial Number: U1111-1271-3861

EU CT Number: 2023-506824-96

IND Number: 133801

Study phase: 2

In the following, Novo Nordisk A/S and its affiliates will be stated as “Novo Nordisk”.

This ~~confidential~~ document is the property of Novo Nordisk. ~~No unpublished information contained herein may be disclosed without prior written approval from Novo Nordisk. Access to this document must be restricted to relevant parties.~~

Protocol amendment summary of changes table

DOCUMENT HISTORY		
Document version	Date	Applicable in country(-ies) and/or site(s)
Protocol version 6.0	07 February 2024	All
Protocol version 5.0	28 April 2023	All
Protocol version 4.0	09 September 2022	All
Protocol version 3.0	22 July 2022	All
Protocol version 2.0	13 July 2022	For Czech Republic only
Original protocol version 1.0	01 March 2022	All

Protocol version 6.0 (07 February 2024)

This amendment is considered to be non-substantial based on the criteria set forth in Article 2(13) of Regulation (EU) No 536/2014 of the European Parliament and the Council of 16 April 2014[1]; because it neither substantially impacts the safety or rights of the participants nor the reliability or robustness of the data generated in the study.

Overall rationale for preparing protocol, version 6.0:

The overall rationale for preparing protocol version 6.0 is to replace the EudraCT number with the EU CT number.

Section # and name	Description of change	Brief rationale
Cover page	EudraCT number has been replaced with the EU CT number	To comply with EU CTR

Table of Contents

Protocol amendment summary of changes table **2**

Table of Contents **3**

1 Protocol summary **7**
1.1 Synopsis 7
1.2 Flowchart 10

2 Introduction **18**
2.1 Study rationale 18
2.2 Background 19
2.3 Benefit-risk assessment 20
2.3.1 Risk assessment 20
2.3.2 Benefit assessment 21
2.3.3 Overall benefit-risk conclusion 22

3 Objectives, endpoints and estimands **23**

4 Study design **26**
4.1 Overall design 26
4.2 Scientific rationale for study design 26
4.3 Justification for dose 28
4.4 End of study definition 29

5 Study population **30**
5.1 Inclusion criteria 30
5.2 Exclusion criteria 30
5.3 Lifestyle considerations 31
5.3.1 Activity 31
5.4 Screen failures 31
5.5 Randomisation criteria 32

6 Study interventions and concomitant therapy **33**
6.1 Study interventions administered 33
6.2 Preparation, handling, storage and accountability 37
6.3 Measures to minimise bias: Randomisation and blinding 39
6.3.1 Randomisation 39
6.3.2 Blinding 39
6.3.3 Blind Break 40
6.4 Study intervention compliance 40
6.5 Dose modification 41
6.6 Continued access to study intervention after end of study 41
6.7 Treatment of overdose 41
6.8 Concomitant therapy 41

7 Discontinuation of study intervention and participant discontinuation/withdrawal **43**
7.1 Discontinuation of study intervention 43
7.1.1 Temporary discontinuation of study intervention 44
7.2 Participant discontinuation/withdrawal from the study 44
7.2.1 Replacement of participants 45
7.3 Lost to follow-up 45

8 Study assessments **46**
8.1 Screening 47
8.2 Efficacy assessments 48
8.2.1 Clinical efficacy laboratory assessments 48
8.2.1.1 Urine collection 48
8.2.2 Clinical outcome assessments 48

8.2.2.1 6-minute walk test (6MWT) 48
8.2.2.2 Neuropathy Impairment Score (NIS) 48
8.2.2.3 Patient reported outcome (PRO) questionnaires 49
8.2.3 Imaging 49
8.2.3.1 Cardiac magnetic resonance imaging (MRI) 50
8.2.3.2 Echocardiography 50
8.3 Safety assessments 51
8.3.1 Physical examinations 51
8.3.2 New York Heart Association (NYHA) classification 52
8.3.3 Body measurements 52
8.3.4 Vital signs 52
8.3.5 Electrocardiograms 53
8.3.6 Cardiac monitoring (only appliable for sentinel participants) 53
8.3.7 Clinical safety laboratory assessments 54
8.3.8 Pregnancy testing 54
8.4 Adverse events and other safety reporting 54
8.4.1 Time period and frequency for collecting AE information 55
8.4.2 Method of detecting AEs 56
8.4.3 Follow-up of AEs 56
8.4.4 Regulatory reporting requirements for SAEs 56
8.4.5 Pregnancy 56
8.4.6 Cardiovascular and death events 56
8.4.7 Technical complaints 56
8.5 Pharmacokinetics and pharmacodynamics 57
8.5.1 Pharmacokinetics 57
8.6 Genetics 57
8.7 Biomarkers 57
8.8 Immunogenicity assessments 58
8.8.1 Anti-NNC6019-0001-antibodies 58
8.8.2 Assessments in case of suspicion of hypersensitivity to trial product 59
8.8.3 Human biosamples for future research 59
8.9 Health economics 60

9 Statistical considerations 61
9.1 Statistical hypotheses 61
9.1.1 Multiplicity adjustment 61
9.2 Analysis sets 61
9.3 Statistical analyses 61
9.3.1 General considerations 61
9.3.2 Primary endpoint analysis 61
9.3.3 Secondary endpoints analysis 62
9.3.3.1 Supportive secondary endpoints 62
9.3.4 Exploratory endpoints analysis 63
9.3.5 Other safety analyses 63
9.3.6 Other analyses 63
9.3.6.1 Pharmacokinetic and pharmacodynamic modelling 63
9.4 Interim analysis 63
9.5 Sample size determination 64

10 Supporting documentation and operational considerations 66
10.1 Appendix 1: Regulatory, ethical, and study oversight considerations 66
10.1.1 Regulatory and ethical considerations 66
10.1.2 Financial disclosure 66
10.1.3 Informed consent process 67
10.1.4 Information to participants during the study 67
10.1.5 Data protection 67

10.1.6 Committee structure 68
10.1.6.1 Novo Nordisk safety committee....68
10.1.6.2 Data monitoring committee....68
10.1.6.3 Steering Committee....68
10.1.6.4 Event adjudication committee....69
10.1.7 Dissemination of clinical study data....69
10.1.8 Data quality assurance....69
10.1.8.1 Case report forms....69
10.1.8.2 Monitoring....70
10.1.8.3 Protocol compliance....71
10.1.9 Source documents....71
10.1.10 Retention of clinical study documentation....71
10.1.11 Study and site closure....72
10.1.12 Responsibilities....72
10.1.13 Indemnity statement....73
10.1.14 Publication policy....73
10.1.14.1 Communication of results....74
10.1.14.2 Authorship....74
10.1.14.3 Site-specific publication(s) by investigator(s)....74
10.1.14.4 Investigator access to data and review of results....75
10.2 Appendix 2: Clinical laboratory tests....76
10.3 Appendix 3: Adverse Events and Serious Adverse Events: Definitions and procedures for recording, evaluating, follow-up, and reporting....79
10.3.1 Definition of AE....79
10.3.2 Definition of an SAE....79
10.3.3 Description of AEs requiring additional data collection....80
10.3.4 Recording and follow-up of AE and/or SAE....81
10.3.4.1 AE and SAE recording....81
10.3.4.2 Assessment of severity....82
10.3.4.3 Assessment of causality....82
10.3.4.4 Final outcome....83
10.3.4.5 Follow-up of AE and SAE....83
10.3.5 Reporting of SAEs....84
10.4 Appendix 4: Contraceptive guidance and collection of pregnancy information....86
10.4.1 Definitions....86
10.4.2 Contraceptive guidance....86
10.4.3 Collection of pregnancy information....87
10.5 Appendix 5: Technical complaints: Definition and procedures for recording, evaluation, follow-up and reporting....89
10.5.1 Definition of technical complaint....89
10.5.2 Recording and follow-up of technical complaints....89
10.5.3 Reporting of technical complaints for products not included in the technical complaint form....90
10.6 Appendix 6: Retention of human biosamples for future research....91
10.6.1 Biosamples for future research....91
10.6.2 Anti-NNC6019-0001-antibodies samples....92
10.6.3 Hypersensitivity reaction samples....92
10.6.4 Pharmacokinetic and pharmacodynamic samples....93
10.7 Appendix 7: Events requiring adjudication....94
10.8 Appendix 8: Country-specific requirements....95
10.9 Appendix 9: Abbreviations....98
10.10 Appendix 10: Protocol amendment history....101

11 References....111

Protocol attachment I Global list of key staff and relevant departments and suppliers

Protocol attachment II Country list of key staff and relevant departments

1 Protocol summary

1.1 Synopsis

This is an interventional, randomised, multinational, multicentre, three-arm parallel-group, double-blind, placebo-controlled study in participants with hereditary ATTR (hATTR) or wild-type ATTR (wtATTR) CM.

Rationale

Transthyretin amyloid cardiomyopathy (ATTR CM) is an increasingly recognised cause of heart failure in older adults worldwide, resulting from extracellular deposition of misfolded transthyretin protein (amyloid) in the myocardium.[3] ATTR CM is a progressive chronic disease with a high burden for patients and society, underscoring the need for therapies that reverse disease pathology and lower the risk of worsening of heart failure, hospitalisation and mortality in patients with ATTR CM. NNC6019-0001 is designed to have an amyloid-depleting mechanism of action for patients at high risk of early mortality due to amyloid deposition in the myocardium. The aims of this proof-of-principle study are to compare the effect of NNC6019-0001 versus placebo on functional endpoints, circulating- and imaging biomarkers as well as to evaluate pharmacokinetics, safety, and tolerability of the two dose levels covering the relevant therapeutic levels and based on the totality of data to select the dose to be studied in phase 3.

Objectives, endpoints and estimands

The primary and secondary objectives and endpoints are summarised in the table below.

**Objectives**	**Endpoints**		
Primary	Title	Time frame	Unit
• To compare the effect of two dose levels of NNC6019-0001 (10 mg/kg and 60 mg/kg) versus placebo on:<br>• change in 6-minute walk test and<br>• change in NT-proBNP<br>from baseline to week 52 in participants with hATTR or wtATTR cardiomyopathy.	*Primary*		
	Change in 6-minute walk test (6MWT)	From baseline (week 0) to visit 15 (week 52)	Meters
	Change in NT-proBNP	From baseline (week 0) to visit 15 (week 52)	Percentage
**Secondary**	**Title**	**Time frame**	**Unit**
• To compare the effect of two dose levels of NNC6019-0001 (10 mg/kg and 60 mg/kg) versus placebo on:<br>• biomarkers<br>• pharmacodynamic endpoints<br>from baseline to week 52 in participants with hATTR or wtATTR cardiomyopathy	*Supportive*		
	Change in myocardial extracellular volume (ECV)	From baseline (week 0) to visit 15 (week 52)	%-points
	Change in Kansas City Cardiomyopathy Questionnaire (KCCQ) Clinical Summary Score[a] (CSS)[4]	From baseline (week 0) to visit 15 (week 52)	Score
	Change in neuropathy impairment score[b] (NIS)	From baseline (week 0) to visit 15 (week 52)	Score
	Change in troponin I	From baseline (week 0) to visit 15 (week 52)	ng/mL

Objectives	Endpoints		
	Change in global longitudinal strain (GLS) on echocardiography	From baseline (week 0) to visit 15 (week 52)	%-points
• To compare the effect of two dose levels of NNC6019-0001 (10 mg/kg and 60 mg/kg) versus placebo on: • safety and tolerability from baseline to week 64 in participants with hATTR or wtATTR cardiomyopathy.	Number of treatment emergent adverse events	From baseline (week 0) to visit 16 (week 64)	Count
	Time to occurrence of all-cause mortality	From baseline (week 0) to visit 16 (week 64)	Weeks
	Number of CV events comprising hospitalisation due to CV events or urgent heart failure visits	From baseline (week 0) to visit 16 (week 64)	Count

[a] Clinical Summary Score (CSS) consists of the Symptom domain and the Physical Limitation domain, additional analyses of the remaining domains will be described in the SAP. KCCQ scores range from 0 to 100 and lower scores represent more severe symptoms and/or limitations and scores of 100 indicate no symptoms, no limitations, and excellent quality of life; [b] Only applicable for participants with hATTR CM. The total NIS score is graded on a scale of 0–244, with a higher score indicating greater impairment.
Abbreviations: CV = cardiovascular; hATTR = hereditary ATTR; NT-proBNP = N-terminal-pro brain natriuretic peptide; wtATTR = wild-type ATTR.

Primary estimand

The primary estimand addresses the following question of interest: What is the effect of two dose levels of NNC6019-0001 (10 mg/kg and 60 mg/kg) versus placebo on change in 6MWT and NT-proBNP from baseline to week 52, or occurrence of death or CV hospitalisation, in participants with hATTR or wtATTR CM, regardless of premature discontinuation of study intervention.

Overall design

This is an interventional, randomised, multinational, multicentre, three-arm parallel-group, double-blind, placebo-controlled study comparing i.v. NNC6019-0001 Q4W at two dose levels (10 mg/kg and 60 mg/kg) versus placebo in participants with hATTR or wtATTR CM.

The study consists of a screening period of up to 8 weeks, followed by a 52-week intervention period. When participants discontinue study intervention according to protocol, an end of treatment visit should be carried out 4 weeks after administration of the last dose and a follow-up visit should be carried out 16 weeks after administration of the last dose.

Study intervention groups, duration, and number of participants

Study intervention groups:

- 10 mg/kg NNC6019-0001 lyophilised powder for solution for i.v. infusion
- 60 mg/kg NNC6019-0001 lyophilised powder for solution for i.v. infusion
- Placebo

Following the screening period, approximately 99 participants will be randomised 1:1:1 to receive i.v. 10 mg/kg NNC6019-0001, 60 mg/kg NNC6019-0001 or placebo Q4W added to standard of care. Randomisation will be stratified by disease type (wtATTR vs hATTR) and maximum 80% of participants randomised will be participants with wtATTR.

The planned study duration for the individual participant will be approximately 64 weeks (excluding screening).

Participant characteristics

Key inclusion criteria:

- Male or female.
- Age ≥ 18 to < 85 years at the time of signing informed consent.
- Have an established diagnosis of ATTR CM with either wild-type TTR or hereditary TTR genotype as per local standards.
- Expected to be on stable doses of cardiovascular medical therapy 6 weeks prior to the randomisation visit.
- Known end-diastolic interventricular septal wall thickness ≥ 12 mm.
- Presently classified as New York Heart Association (NYHA) Class II-III.
- NT-proBNP concentration ≥650 pg/mL in sinus cardiac rhythm and >1000 pg/mL in atrial fibrillation at screening.
- Completed ≥150 meters to ≤450 meters on the 6MWT at screening.
- Estimated glomerular filtration rate (eGFR) ≥25 mL/min/1.73 m^2 at screening.

Key exclusion criteria:

- Cardiomyopathy not primarily caused by ATTR CM, for example, cardiomyopathy due to hypertension, valvular heart disease, or ischemic heart disease
- A prior solid organ transplant.
- Planned solid organ transplant during the study.
- Presence or history of malignant neoplasm (other than basal or squamous cell skin cancer, in-situ carcinomas of the cervix, or in-situ/high grade prostatic intraepithelial neoplasia (PIN) or low-grade prostate cancer) within 5 years before screening.
- Current treatment with calcium channel blockers with conduction system effects (e.g., verapamil, diltiazem). The use of dihydropyridine calcium channel blockers is allowed. The use of digoxin will only be allowed if required for management of atrial fibrillation with rapid ventricular response.
- Acute coronary syndrome, unstable angina, stroke, transient ischemic attack (TIA), coronary revascularization, cardiac valve repair, or major surgery within 3 months of screening.
- Body weight >120 kg (264.6 lb) at screening.
- History of contrast allergy or adverse reactions to gadolinium-containing agents.

Efficacy and safety data will be collected at regular intervals throughout the study.

Data monitoring committee
Yes.

1.2 Flowchart

Procedure	Protocol Section	Screening		Randomisation	Study intervention period																End of treatment	Follow-up
Visit		V1	V1A[a]	V2	V2A[b]	V2B[b]	V2C[b]	V2D[b]	V3	V4	V5	V6	V7	V8	V9	V10	V11	V12	V13	V14	V15	V16[c]
Timing of Visit (weeks)		up to -8 weeks	up to -2 weeks	0	24h	1	2	3	4	8	12	16	20	24	28	32	36	40	44	48	52	64
Visit Window (Days)				0	0	+2	±2	±2	±4	±4	±4	±4	±4	±4	±4	±4	±4	±4	±4	±4	±4	+7
**PARTICIPANT RELATED INFORMATION AND ASSESSMENTS**																						
Informed Consent and Demography[d]	8.1, 10.1.3	X																				
Tobacco Use	8.1	X																				
Childbearing Potential	8.3.8	X																				
Medical History/Concomitant Illness	8.3	X	X																			
Concomitant Medication	6.8	X	X	X	X	X	X	X	X	X	X	X	X	X	X	X	X	X	X	X	X	X
NYHA Classification	8.3.2	X	X	X		X	X	X	X		X			X							X	X
Pregnancy Test[e]	8.3.8, 10.4	X		X					X	X	X	X	X	X	X	X	X	X	X	X	X	X
Eligibility Criteria	5	X	X	X																		
Inclusion Criteria	5.1	X	X																			
Exclusion Criteria	5.2	X	X																			
Randomisation Criteria	5.5			X																		
Randomisation	6.3.1			X																		

Procedure	Protocol Section	Screening		Randomisation	Study intervention period															End of treatment	Follow-up
Visit		V1	V1A[a]	V2	V2A[b]	V2B[b]	V2C[b]	V2D[b]	V3	V4	V5	V6	V7	V8	V9	V10	V11	V12	V13	V14	V15	V16[c]
Timing of Visit (weeks)		up to -8 weeks	up to -2 weeks	0	24h	1	2	3	4	8	12	16	20	24	28	32	36	40	44	48	52	64
Visit Window (Days)				0	0	+2	±2	±2	±4	±4	±4	±4	±4	±4	±4	±4	±4	±4	±4	±4	±4	+7
Upload 99mTc PYP/DPD/HMDP cardiac scintigraphy including SPECT[f]	8.1	X																				
**SAFETY**																						
Vital Signs	8.3.4, Table 6-2	X	X	X	X	X	X	X	X	X	X	X	X	X	X	X	X	X	X	X	X	X
Physical Examination	8.3.1	X		X	X	X	X	X	X		X			X						X	X	
Body Measurements	8.3.3	X	X	X		X	X	X	X	X	X	X	X	X	X	X	X	X	X	X	X	X
Body weight	8.3.3	X	X	X		X	X	X	X	X	X	X	X	X	X	X	X	X	X	X	X	X
Height	8.3.3			X																		
ECG	8.3.5	X	X	X	X	X	X	X	X		X			X							X	X
Safety Monitoring[b]	8.3.6	X	X	X	X																	
24-hours Cardiac Monitoring In-Patient[b, h]	8.3.6			X																		
Cardiac Monitoring Out-Patient[b]	8.3.6	X[i]	X[i]		X[j, h]																	
Adverse Event	8.4			X	X	X	X	X	X	X	X	X	X	X	X	X	X	X	X	X	X	X
Laboratory Assessments	10.2	X	X	X	X	X	X	X	X	X	X			X							X	
Serology Reminder	10.2	X																				

Procedure	Protocol Section	Screening		Randomisation	Study intervention period																End of treatment	Follow-up
Visit		V1	V1A[a]	V2	V2A[b]	V2B[b]	V2C[b]	V2D[b]	V3	V4	V5	V6	V7	V8	V9	V10	V11	V12	V13	V14	V15	V16[c]
Timing of Visit (weeks)		up to -8 weeks	up to -2 weeks	0	24h	1	2	3	4	8	12	16	20	24	28	32	36	40	44	48	52	64
Visit Window (Days)				0	0	+2	±2	±2	±4	±4	±4	±4	±4	±4	±4	±4	±4	±4	±4	±4	±4	+7
Coagulation Parameter[k]	10.2	X	X	X	X	X		X	X	X	X			X							X	
Biochemistry	10.2	X	X	X	X	X	X	X	X	X	X			X							X	
Urinalysis[l]	10.2			X					X		X			X							X	
Haematology	10.2	X	X	X	X	X	X	X	X	X	X			X							X	
**OTHER ASSESSMENTS**																						
Clinical Outcome Assessments		X	X	X					X		X			X							X	
6MWT	8.2.2.1	X	X	X					X		X			X							X	
Patient Global Impression of Status (PGI-S) for 6MWT	8.2.2.3			X										X							X	
Patient Global Impression of Change (PGI-C) for 6MWT	8.2.2.3													X							X	
KCCQ	8.2.2.3			X					X		X			X							X	
Patient Global Impression of Status (PGI-S) for KCCQ	8.2.2.3			X										X							X	
Patient Global Impression of Change (PGI-C) for KCCQ	8.2.2.3													X							X	
EQ-5D-5L	8.2.2.3			X					X		X			X							X	

Procedure	Protocol Section	Screening		Randomisation	Study intervention period																End of treatment	Follow-up
Visit		V1	V1A[a]	V2	V2A[b]	V2B[b]	V2C[b]	V2D[b]	V3	V4	V5	V6	V7	V8	V9	V10	V11	V12	V13	V14	V15	V16[c]
Timing of Visit (weeks)		up to -8 weeks	up to -2 weeks	0	24h	1	2	3	4	8	12	16	20	24	28	32	36	40	44	48	52	64
Visit Window (Days)				0	0	+2	±2	±2	±4	±4	±4	±4	±4	±4	±4	±4	±4	±4	±4	±4	±4	+7
NIS[m]	8.2.2.2			X										X							X	
**EFFICACY**																						
Cardiac MRI	8.2.3.1			X					X[b]		X			X							X	
Echocardiography	8.2.3.2			X					X		X			X							X	
PK/PD	8.5			X	X	X	X	X	X	X	X	X	X	X	X	X	X	X	X	X	X	X
PK[n]	8.5.1			X	X	X	X	X	X	X	X	X	X	X	X	X	X	X	X	X	X	X
Misfolded transthyretin (misTTR)	8.7			X					X		X			X							X	
Anti-drug antibodies	8.8.1			X			X		X	X	X			X				X			X	X
Inflammatory Biomarkers	8.7			X	X	X	X	X	X		X			X							X	
Cardiac biomarkers	8.7	X	X	X	X	X	X	X	X		X			X							X	
**REMINDERS**																						
Hand Out ID Card		X																				
Pre-medication	6.1			X					X	X	X	X	X	X	X	X	X	X	X	X		
RTSM/IWRS	6.2	X		X					X	X	X	X	X	X	X	X	X	X	X	X	X	
Samples for future analysis[g]	8.8.3			X					X		X			X							X	

Procedure	Protocol Section	Screening		Randomisation	Study intervention period																End of treatment	Follow-up
Visit		V1	V1A[a]	V2	V2A[b]	V2B[b]	V2C[b]	V2D[b]	V3	V4	V5	V6	V7	V8	V9	V10	V11	V12	V13	V14	V15	V16[c]
Timing of Visit (weeks)		up to -8 weeks	up to -2 weeks	0	24h	1	2	3	4	8	12	16	20	24	28	32	36	40	44	48	52	64
Visit Window (Days)				0	0	+2	±2	±2	±4	±4	±4	±4	±4	±4	±4	±4	±4	±4	±4	±4	±4	+7
Biosamples (blood) for Future Analysis Taken	8.8.3			X					X		X			X							X	
Biosamples (blood) for Genetic Analysis Taken	8.8.3			X																	X	
Urine samples for future Analysis Taken	8.8.3			X																	X	
Hand Out Urine Kit	8.2.1.1	X		X						X			X							X		
**STUDY MATERIAL**																						
Administration of Trial Product	6.2			X					X	X	X	X	X	X	X	X	X	X	X	X		
Drug Dispensing	6.2			X					X	X	X	X	X	X	X	X	X	X	X	X		

a: Only applicable when the randomisation visit is more than 2 weeks after start of screening procedures.

b: Only applicable for participants in the sentinel cohort.

c: When participants discontinue study intervention prematurely, a follow-up visit should be carried out 16 weeks after last dosing. If this coincides with another scheduled visit, assessments pertaining to Visit 16 should be done in addition to the assessments pertaining to the other scheduled visit.

d: Demography consists of date of birth, age, sex, ethnicity, and race (according to local regulation). Race and ethnicity must be self-reported by the participant.

e: Only applicable for women of childbearing potential.

f: Only applicable if the ATTR CM diagnosis is based on non-invasive diagnostic criteria; transfer the most recent 99mTc PYP/DPD/HMDP cardiac scintigraphy including SPECT available for central review.

g: A separate consent is needed for the collection of these samples (optional).

h: Only applicable for the first 6 randomised Japanese participants.

i: 48-hour out-patient cardiac monitoring (before randomisation).

j: 6 days out-patient cardiac monitoring after discharge at V2A.

k: For participants on anticoagulants requiring laboratory monitoring the monitoring should take place as per local standards independent of study participation or similar.

l: Measured from two first morning void urine samples, 1 day before the visit and on the day of the visit.

m: Only applicable for participants with hATTR CM.

n: PK samples to be taken according to Table 1-1.

The PK sampling scheme is provided in Table 1-1.

Table 1-1 Sampling scheme for PK sampling

Visit	Week no. (visit window)	Dose	PK sample		Time relative to dose [a]	Time window allowance
V2	Week 0		X	Pre-dose sample	Before start of infusion	-30 min
		X				
			X	Post-dose sample	After end of infusion	+30 min
V2A[b]	Week 0 +24h		X	PK sample	24 hours	±3 h
V2B[b]	Week 1 (+2 days)		X	PK sample	7 days	
V2C[b]	Week 2 (± 2 days)		X	PK sample	14 days	
V2D[b]	Week 3 (± 2 days)		X	PK sample	21 days	
V3	Week 4		X	Pre-dose sample	Before start of infusion	-30 min
		X				
			X	Post-dose sample	After end of infusion	+30 min
V4	Week 8		X	Pre-dose sample	Before start of infusion	-30 min
		X				

Visit	Week no. (visit window)	Dose	PK sample		Time relative to dose [a]	Time window allowance
			X	Post-dose sample	After end of infusion	+30 min
V5	Week 12		X	Pre-dose sample	Before start of infusion	-30 min
		X				
			X	Post-dose sample	After end of infusion	+30 min
V6	Week 16		X	Pre-dose sample	Before start of infusion	-30 min
		X				
			X	Post-dose sample	After end of infusion	+30 min
V7	Week 20		X	Pre-dose sample	Before start of infusion	-30 min
		X				
			X	Post-dose sample	After end of infusion	+30 min
V8	Week 24		X	Pre-dose sample	Before start of infusion	-30 min
		X				
			X	Post-dose sample	After end of infusion	+30 min
V9	Week 28		X	Pre-dose sample	Before start of infusion	-30 min
		X				
			X	Post-dose sample	After end of infusion	+30 min
V10	Week 32		X	Pre-dose sample	Before start of infusion	-30 min
		X				
			X	Post-dose sample	After end of infusion	+30 min
V11	Week 36		X	Pre-dose sample	Before start of infusion	-30 min
		X				
			X	Post-dose sample	After end of infusion	+30 min
			X	Pre-dose sample	Before start of infusion	-30 min

Visit	Week no. (visit window)	Dose	PK sample		Time relative to dose [a]	Time window allowance
V12	Week 40	X				
			X	Post-dose sample	After end of infusion	+30 min
V13	Week 44		X	Pre-dose sample	Before start of infusion	-30 min
		X				
			X	Post-dose sample	After end of infusion	+30 min
V14	Week 48		X	Pre-dose sample	Before start of infusion	-30 min
		X				
			X	Post-dose sample	After end of infusion	+30 min
V15	Week 52		X	PK sample	4 weeks	±4 days
V16	Week 64		X	PK sample	16 weeks	+7 days

[a] From start of dosing for the pre-dose sampling and from end of dosing for the post-dose sampling; [b] Only applicable for participants in the sentinel cohort.

2 Introduction

2.1 Study rationale

Transthyretin amyloid cardiomyopathy (ATTR CM) is an increasingly recognised cause of heart failure in older adults worldwide, resulting from extracellular deposition of misfolded transthyretin protein (amyloid) in the myocardium.[3] ATTR CM is a progressive chronic disease with a high burden for patients and society, underscoring the need for therapies that reverse disease pathology and lower the risk of worsening of heart failure, hospitalisation and mortality in patients with ATTR CM.

ATTR CM can be hereditary (hATTR) due to rare genetic variants or occur sporadically as wild-type (wtATTR).[5] Patients with deposition of amyloid in the myocardium often present with symptoms and signs suggestive of chronic heart failure (i.e. dyspnea on exertion, peripheral edema, fatigue, elevated jugular venous pressure, hepatojugular reflux) and/or arrhythmias (i.e. palpitations, light-headedness, syncope).[6]

Due to advances in diagnostic strategies and the possibility of achieving non-invasive diagnosis, ATTR CM is a more frequent disease than previously thought.[7] Both the prevalence and incidence of ATTR CM has been reported to increase over the past decade.[8, 9] ATTR CM is a rare disease with an unknown global prevalence. Local studies, which were conducted in various regions with differing methods, suggest that at least 0.3–30 in 100,000 people live with ATTR CM.[8-12] Wild type ATTR CM is more frequently reported than hATTR CM[10-13], but data on the proportional distribution of hATTR CM vs wtATTR CM are not available. It is further estimated that up to 13.3% of patients with heart failure with preserved ejection fraction (HFpEF) may have ATTR CM, indicating that it is a more frequent cause of HFpEF than previously anticipated.[10, 11, 14] Once diagnosed, prognosis is particularly poor, dependent on sub-type median survival in untreated patients is approximately 2.5 (hATTR) to 5 years (wtATTR).[5]

Currently available treatment options are limited and include supportive treatment of cardiac involvement with medications (i.e. diuretics), implantable devices (i.e. pacemakers) or organ (heart and/or liver) transplantation.[15] More recently, a transthyretin tetramer stabiliser (tafamidis[16, 17] for ATTR CM and silencers that reduce expression of mutant TTR (inotersen[18, 19] and patisiran[20, 21] for hATTR polyneuropathy (PN) have been approved in the US and EU.

In a global randomised clinical study, known as the ATTR-ACT study,[22] the transthyretin tetramer stabiliser tafamidis was tested in patients with ATTR CM. Tafamidis was associated with a 30% reduction in all-cause mortality vs placebo (HR 0.70 $[0.51; 0.96]_{95\%CI}$), and a 32% reduction in cardiovascular related hospitalisations (RR 0.68 $[0.56; 0.81]_{95\%CI}$). The residual risk, however, still appears to be high, and especially so in more advanced disease (NYHA III at baseline). There was still disease progression in the tafamidis arm (decline in 6-minute walk test [6MWT], increase in NT-proBNP and decreasing KCCQ-OS score), however significantly slower than in the placebo arm.[22] In addition, transthyretin tetramer stabilisers and silencers do not target the removal of amyloid already deposited in the tissues.

Hence, there is a major unmet medical need to improve the treatment of patients with ATTR CM. NNC6019-0001 (previously known as PRX004) is a humanised IgG1κ monoclonal antibody

designed to specifically target a unique epitope, amino acid residues 89-97 (EHAEVVFTA) of TTR that is exposed only on monomeric, misfolded, and aggregated forms of TTR such as found in wtATTR and hATTR but hidden in the native tetramer conformation. NNC6019-0001 is a recombinant product produced using a Chinese hamster ovary-derived cell line. NNC6019-0001 is administered every 4 weeks (Q4W) as an intravenous (i.v.) infusion.

Currently available non-clinical and clinical data indicate that NNC6019-0001 may deplete accumulated amyloid from the myocardium and consequently may reverse disease pathology and improve functional outcomes in patients with ATTR CM. In the FHD study (study NN6019-4965), NNC6019-0001 was associated with a mean change of –1.21% in global longitudinal strain (GLS) indicating a possible benefit in cardiac systolic function and NNC6019-0001 was safe and well tolerated supporting further development.[23] Taken together, the clinical findings suggest that NNC6019-0001's amyloid-depleting mechanism of action can result in clinical benefits and thus, NNC6019-0001 may provide a new treatment option for patients at high risk of early mortality due to amyloid deposition in the heart. The aims of this proof-of-principle study are to compare the effect of NNC6019-0001 versus placebo on functional endpoints, circulating- and imaging biomarkers as well as to evaluate pharmacokinetics, safety, and tolerability of the two dose levels covering the relevant therapeutic levels and based on the totality of data to select the dose to be studied in phase 3.

In the future NN6019 development programme, the term hereditary ATTR will be updated to variant ATTR (ATTRv) and wild-type ATTR will be abbreviated to ATTRwt.[24, 25] These updated terms will be used in the reporting (CSR) of the current study, NN6019-4940.

2.2 Background

Transthyretin amyloid cardiomyopathy (ATTR CM)

Transthyretin amyloid (ATTR) amyloidosis is a rare and progressive disease characterised by deposition of aggregates of misfolded transthyretin protein (amyloid). Transthyretin (TTR) is a naturally occurring protein, which may misfold to form toxic soluble monomers that subsequently may aggregate and form fibrils with resultant TTR amyloid deposition into tissues (e.g., heart, nerves, gastrointestinal tract) with disrupted organ structure and function as the consequence.[26] The TTR protein is produced primarily in the liver and in its normal tetrameric form serves as a carrier for thyroxin and vitamin A, the latter via the binding of retinol binding protein.

ATTR amyloidosis can be hereditary (hATTR) due to rare genetic variants or occur sporadically as wild type (wtATTR).[5]

In hATTR amyloidosis, the body makes a mutant form of the TTR protein. There are more than 100 reported types of TTR mutations that promote amyloid fibril formation. The predominant organ involvement for hATTR amyloidosis is either the nervous system, termed hATTR polyneuropathy (hATTR PN), or the heart, hATTR cardiomyopathy (hATTR CM), although other organ systems are also often involved.[5, 27, 28] Depending on the specific mutation, some patients predominantly have cardiac symptoms, some predominantly have symptoms from the nervous system, and some have a combination of both. In one study of approximately 500 patients with the hATTR V30M mutation, one third of patients had clinical nephropathy based on elevated levels of proteinuria, and

10% progressed to end-stage renal disease.[29, 30] A significant number of TTR mutations associated with a clinical phenotype cause a restrictive cardiomyopathy.[28]

Wild-type ATTR is similar to hATTR except that the protein that is deposited is the misfolded, non-mutated transthyretin protein. The misfolding is thought to be caused by age-related impaired proteostasis.[31] The predominant effect of wtATTR amyloidosis is on the heart, wtATTR cardiomyopathy (wtATTR CM).

2.3 Benefit-risk assessment

The main benefits and risks related to participation in the study are described in the below sections. More detailed information about the known and expected benefits and risks of NNC6019-0001 may be found in the current edition of the investigator's brochure[32] or updates thereof.

2.3.1 Risk assessment

The risk assessment is presented in Table 2-1.

Table 2-1 Risk assessment

Potential risk of clinical significance	Summary of data/rationale for risk	Mitigation strategy
**Study intervention: NNC6019-0001**		
**Potential risk**: Hypersensitivity	As expected for a protein-based drug, participants treated with NNC6019-0001 may develop localised (to the infusion site) or generalised hypersensitivity reactions.	To mitigate risk of hypersensitivity reactions, participants will be treated with histamine (H1) blockers and acetaminophen/paracetamol prior to receiving NNC6019-0001.<br>As a precaution, participants with known or suspected hypersensitivity to NNC6019-0001 or related products are excluded. Participants and investigators will be instructed to detect signs and symptoms of hypersensitivity reactions. In addition, participants will be instructed to contact the site staff as soon as possible for further guidance if suspicion of a hypersensitivity reaction to the NNC6019-0001 occurs.
**Potential risk**: Myocardial Inflammation	The intended action of the NNC6019-0001 in clearing amyloid through macrophage activation and phagocytosis may result in a theoretical risk of myocardial inflammation.	Participants will be followed closely and carefully by qualified medical staff.<br>To minimise the risk, standard safety surveillance activities and medical monitoring will be performed by Novo Nordisk.
**Potential risk**: Cardiac arrhythmia	Based on the potential mechanism of action of NNC6019-0001 and the underlying disease pathophysiology of ATTR cardiomyopathy, there is a theoretical risk of arrhythmogenicity.	Participants will be followed closely and carefully by qualified medical staff.<br>To minimise the risk, standard safety surveillance activities and medical monitoring will be performed by Novo Nordisk.
**Study procedures**		
**COVID-19:** Risk of COVID-19 infection in relation to participation in the study	Participants may be exposed to the risk of COVID-19 transmission and infection in relation to site visits if an outbreak is ongoing in the given country.	The risk of COVID-19 transmission in relation to site visits is overall considered to be low, however this may vary over time and between geographical areas.<br>Where relevant, to minimise the risk as much as possible, the following measures will be taken:

Potential risk of clinical significance	Summary of data/rationale for risk	Mitigation strategy
		• Cautious participant recruitment planning to ensure controlled participant enrolment in countries where the COVID-19 pandemic is evaluated to be sufficiently under control, and at sites where health care resources are evaluated to be adequate.<br>• On-site visits are planned to be as short as possible. Physical contact between participants and site staff is limited to the extent possible, and protective measures are implemented (e.g., use of masks, sanitisers, no aerosol-generating procedures etc.) according to local practice.
**Cardiac MRI**	All medical imaging examinations are carried out using a scanner where the participant lies on a bed that is moved into the scanner so that the head is inside the scanner tunnel. This procedure may be perceived as unpleasant for people suffering from claustrophobia. Further, the MRI examination involves the use of strong magnetic field but does not involve radiation exposure.	To minimise the risk of claustrophobia and risks associated with the magnetic field, local guidelines will be followed (such as excluding participants with certain implanted devices or other incompatible metallic objects in their body to having a cardiac MRI performed).<br>A gadolinium contrast agent will be applied intravenously during the cardiac MRI and it normally does not cause side effects. To minimize the rare risk of adverse reactions to gadolinium, people with advanced kidney disease (such as eGFR<30 ml/min/1.73m2) according to local guidelines will not undergo cardiac MRI.
**Other**		
**Pregnancy and fertility**	Studies in animals have not shown reproductive toxicity. There are limited data from the use of NNC6019-0001 in pregnant women.	NNC6019-0001 should not be used during pregnancy. Women of childbearing potential are required to use highly effective contraceptive methods when participating in this study (Appendix 4 [Section 10.4]) If a female participant wishes to become pregnant, or pregnancy occurs during the study, treatment with study intervention should be discontinued immediately (please refer to Section 7.1 for further guidance). The effect of NNC6019-0001 on fertility in humans is unknown.

2.3.2 Benefit assessment

Preliminary efficacy results from the FHD study (study NN6019-4965) for 7 evaluable participants treated with NNC6019-0001 were favourable (please see the current IB[32] for more details). NNC6019-0001 was associated with a mean change of – 1.21% in GLS from baseline to 9 months indicating a possible benefit in cardiac systolic function. Six (6) of the 7 efficacy evaluable participants had no change in their baseline New York Heart Association (NYHA) class at month 9. NNC6019-0001 demonstrated a mean change of 1.29 in Neuropathy Impairment Score (NIS) from baseline to 9 months indicating a stable peripheral nerve function. Participation in this study is contributing to the process of developing a new therapy option for patients with ATTR CM with a proposed amyloid-depleting mode of action. Expected benefits associated with treatment with NNC6019-0001 include those associated with removal of amyloid in tissues, specifically in the myocardium.

It is expected that all participants will benefit from participation through frequent and close contact with investigators and other site staff who will ensure that the participants are treated to

recommended standard of care for their conditions, including ATTR CM, and disease development and progression will be closely monitored and treated.

2.3.3 Overall benefit-risk conclusion

Taking into account the measures taken to minimise risk and burden to participants participating in this study, the potential risks identified in association with NNC6019-0001 are justified by the anticipated benefits that may be afforded to participants with ATTR CM.

3 Objectives, endpoints and estimands

The objectives and endpoints are listed in Table 3-1.

Table 3-1 Objectives and endpoints

**Objectives**	**Endpoints**		
Primary	Title	Time frame	Unit
• To compare the effect of two dose levels of NNC6019-0001 (10 mg/kg and 60 mg/kg) versus placebo on: • change in 6-minute walk test and • change in NT-proBNP from baseline to week 52 in participants with hATTR or wtATTR cardiomyopathy.	*Primary*		
	Change in 6-minute walk test (6MWT)	From baseline (week 0) to visit 15 (week 52)	Meters
	Change in NT-proBNP	From baseline (week 0) to visit 15 (week 52)	Percentage
**Secondary**	**Title**	**Time frame**	**Unit**
• To compare the effect of two dose levels of NNC6019-0001 (10 mg/kg and 60 mg/kg) versus placebo on: • biomarkers • pharmacodynamic endpoints from baseline to week 52 in participants with hATTR or wtATTR cardiomyopathy.	*Supportive*		
	Change in myocardial extracellular volume (ECV)	From baseline (week 0) to visit 15 (week 52)	%-points
	Change in Kansas City Cardiomyopathy Questionnaire (KCCQ) Clinical Summary Score[a] (CSS)[4]	From baseline (week 0) to visit 15 (week 52)	Score
	Change in neuropathy impairment score[b] (NIS)	From baseline (week 0) to visit 15 (week 52)	Score
	Change in troponin I	From baseline (week 0) to visit 15 (week 52)	ng/mL
	Change in global longitudinal strain (GLS) on echocardiography	From baseline (week 0) to visit 15 (week 52)	%-points
• To compare the effect of two dose levels of NNC6019-0001 (10 mg/kg and 60 mg/kg) versus placebo on: • safety and tolerability from baseline to week 64 in participants with hATTR or wtATTR cardiomyopathy.	Number of treatment emergent adverse events	From baseline (week 0) to visit 16 (week 64)	Count
	Time to occurrence of all-cause mortality	From baseline (week 0) to visit 16 (week 64)	Weeks
	Number of CV events comprising hospitalisation due to CV events or urgent heart failure visits	From baseline (week 0) to visit 16 (week 64)	Count
**Exploratory**	**Title**	**Time frame**	**Unit**
	Exploratory		
• To compare the effect of two dose levels of NNC6019-0001 (10 mg/kg and 60 mg/kg) versus placebo on depletion of plasma misTTR from baseline to week 52 in participants with hATTR or wtATTR cardiomyopathy.	Change in misfolded transthyretin[c] (misTTR)	From baseline (week 0) to visit 15 (week 52)	Percentage

Objectives	Endpoints		
• To compare the effect of two dose levels of NNC6019-0001 (10 mg/kg and 60 mg/kg) versus placebo on change in EQ-5D-5L from baseline to week 52 in participants with hATTR or wtATTR cardiomyopathy.	Change in EQ-5D-5L	From baseline (week 0) to visit 15 (week 52)	Score

[a] Clinical Summary Score (CSS) consists of the Symptom domain and the Physical Limitation domain, additional analyses of the remaining domains will be described in the statistical analysis plan (SAP). Scores range from 0 to 100 and lower scores represent more severe symptoms and/or limitations and scores of 100 indicate no symptoms, no limitations, and excellent quality of life; [b] Only applicable for participants with hATTR CM. The total NIS score is graded on a scale of 0–244, with a higher score indicating greater impairment; [c] Including fragmented TTR depending on assay development.

Abbreviations: CV = cardiovascular; hATTR = hereditary ATTR; misTTR = misfolded transthyretin; NT-proBNP = N-terminal-pro brain natriuretic peptide; wtATTR = wild-type ATTR.

Primary estimand

The primary estimand addresses the following question of interest: What is the effect of two dose levels of NNC6019-0001 (10 mg/kg and 60 mg/kg) versus placebo on change in 6MWT and NT-proBNP from baseline to week 52, or occurrence of death or CV hospitalisation, in participants with hATTR or wtATTR cardiomyopathy, regardless of premature discontinuation of study intervention.

The primary estimand is defined with the five attributes as defined in ICH E9(R1) addendum[33]

- Treatment condition: The treatment regimen evaluated is i.v. infusion of NNC6019-0001 Q4W at two dose levels (10 mg/kg and 60 mg/kg) vs placebo, irrespective of use of concomitant medications indicated for ATTR CM or ATTR PN (TTR silencers and stabilisers).
- Population: The treatment effect is assessed for the target population of patients with hATTR or wtATTR CM.
- Variable: The treatment effect is assessed by change in 6MWT and relative change in NT-proBNP from baseline to week 52.
- Remaining intercurrent events (see Table 3-2):
  Premature discontinuation of randomised study intervention will be handled by a treatment policy strategy including all post-discontinuation observations of 6MWT and NT-proBNP in the analysis and conditional multiple imputation from the placebo arm of missing values.
  - Missing values will be handled as follows:
    - Missing 6MWT values due to all-cause death or CV hospitalisation or urgent heart failure visit will be handled by a composite strategy assigning a single value of 0 meters
    - The risk of missing 6MWT due to fractures or fall-related injuries is assumed unrelated to treatment and missing values will be handled by a hypothetical strategy depending on whether the participant is on the randomised study intervention or has prematurely discontinued.
    - Missing 6MWT values for other than the above reasons will be handled by a hypothetical strategy depending on whether the participant is on the randomised study intervention or has prematurely discontinued.
    - Missing NT-proBNP values due to all-cause death or CV hospitalisation or urgent heart failure visit will be handled by a composite strategy assigning a single value

corresponding to the highest observed NT-proBNP value across all participant and visits (including baseline).
    - Missing NT-proBNP values for other reasons will be handled by a hypothetical strategy depending on whether the participant is on the randomised study intervention or has prematurely discontinued.
- Population-level summary:
    - Difference in mean change in 6MWT from baseline between NNC6019-0001 (10 mg/kg or 60 mg/kg) vs placebo.
    - Difference in mean change in NT-proBNP (log-scale) from baseline between NNC6019-0001 (10 mg/kg or 60 mg/kg) vs placebo. The difference will be back transformed to original scale and reported as a ratio of geometric mean ratios.

Table 3-2 Handling of premature discontinuation of randomised study intervention and missing values for various reasons

		**Premature discontinuation of randomised study intervention**	
**Value**	Endpoint	No	Yes
**Observed**	6MWT<br>NT-proBNP	Use value	Use value
**Missing due to death or CV hospitalisation or urgent heart failure visit**	6MWT<br>NT-proBNP	Assign 0 meters<br>Assign highest observed	Assign 0 meters<br>Assign highest observed
**Missing due to fracture or other reason**	6MWT<br>NT-proBNP	Impute from own group	Impute from placebo group

Abbreviations: 6MWT = 6-minute walk test; NT-proBNP = N-terminal-pro brain natriuretic peptide.

Rationale for the estimand

The estimand for the two primary endpoints is chosen to best quantify a potential effect on the planned phase 3 primary endpoint considering both efficacy and tolerability. To penalise the phase 3 primary endpoint events as much as possible within sensible limits, it is chosen to assign a single worst-case value of 0 meters for 6MWT and the highest observed value of NT-proBNP, acknowledging that such values may not be directly interpretable.

4 Study design

4.1 Overall design

This is an interventional, randomised, multinational, multicentre, three-arm parallel-group, double-blind, placebo-controlled study comparing i.v. NNC6019-0001 Q4W at two dose levels (10 mg/kg and 60 mg/kg) versus placebo in participants with hATTR or wtATTR CM.

Approximately 99 participants will be randomised 1:1:1 to receive i.v. 10 mg/kg NNC6019-0001, 60 mg/kg NNC6019-0001 or placebo Q4W added to standard of care. Randomisation will be stratified by disease type (wtATTR vs hATTR) and maximum 80% of participants randomised will be participants with wtATTR.

The study consists of a screening period of up to 8 weeks, followed by a 52-week intervention period. For participants with a screening period lasting more than 2 weeks, a pre-randomisation visit should be carried out maximum 2 weeks prior to the randomisation visit to verify safety related eligibility criteria that tend to fluctuate over time. When participants discontinue study intervention according to protocol, an end of treatment visit should be carried out 4 weeks after administration of the last dose and a follow-up visit should be carried out 16 weeks after administration of the last dose. For participants who discontinue study intervention prematurely, please refer to Section 7.1. The planned study duration for the individual participant will be approximately 64 weeks (excluding screening). The study design is illustrated in Figure 4-1.

Sentinel dosing and intensified safety monitoring will be carried out for minimum 9 participants (3 participants per intervention group), see details on sentinel dosing in Section 6.1. Participants in the sentinel cohort will continue to be dosed Q4W and be part of the main cohort. Stratification by disease type will not be applied for participants in the sentinel cohort.

Figure 4-1 Study design

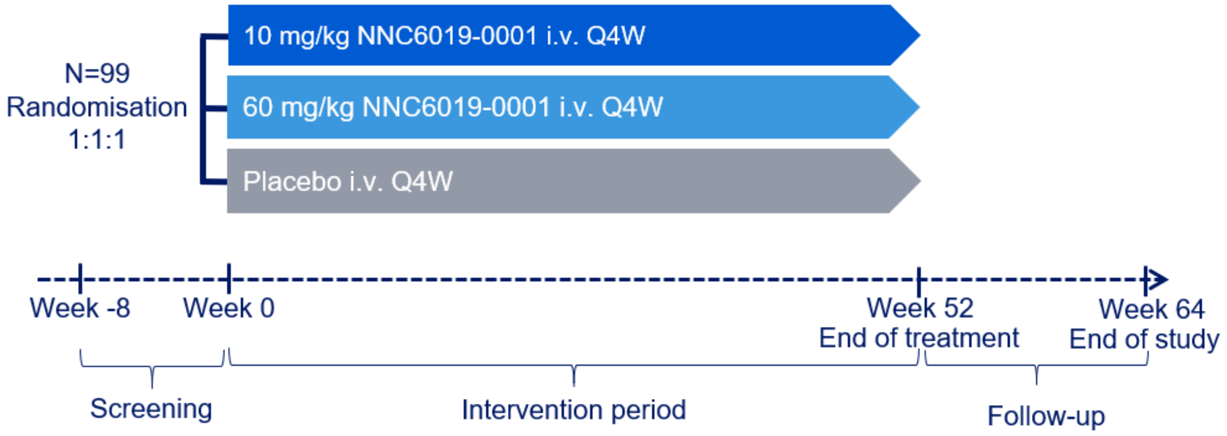

Abbreviations: i.v. = intravenous; N = number of randomised participants; Q4W = every 4 weeks.

4.2 Scientific rationale for study design

The study is designed as a 3-armed study (10 mg/kg NNC6019-0001, 60 mg/kg NNC6019-0001 and placebo) in accordance with the study objectives and to minimise bias. Randomised and

double-blinded intervention with NNC6019-0001 or placebo offers a robust method for assessment of the effects of NNC6019-0001.

A placebo-controlled design is chosen to ensure scientific rigour of the study. The placebo control will facilitate evaluation of efficacy as well as safety and tolerability of NNC6019-0001 by allowing adverse events associated with NNC6019-0001 to be distinguished from symptoms of the underlying disease. Established standard of care will be allowed throughout the study, therefore, assignment to placebo will not place participants at increased risk compared to patients not participating in the study.

The present study will include a population of patients with established ATTR CM. To ensure representative inclusion in all intervention groups, randomisation will be stratified by disease type (wtATTR vs hATTR). Randomisation will not be stratified by disease type for participants in the sentinel cohort.

To safeguard the most vulnerable populations, the age limit is set to <85 years at screening. The participants should be classified as NYHA class at II-III and the participants should be able to complete ≥150 meters to ≤450 meters on the 6MWT at screening. The study population is chosen to optimise the likelihood of achieving a clinical benefit of the treatment and it will be ensured that participants with both wtATTR and hATTR are included by randomising a maximum of 80% participants with wtATTR. Cardiac involvement will be ensured with left ventricular wall thickness of ≥12 mm[7] and with NT-proBNP minimum values (NT-proBNP ≥650 in sinus rhythm and >1000 pg/mL in atrial fibrillation). Participants are required to have an eGFR ≥25 mL/min/1.73m^2 at screening to minimise the risk of development of nephrogenic systemic fibrosis (NSF)[34] related to MRI imaging procedure for the majority of the included population while still allowing entry for participants with more advanced disease. Participants should be on stable doses of cardiovascular medical therapy for at least 6 weeks prior to the randomisation visit to reduce possible confounding bias.

The study is a non-confirmatory study with 2 primary endpoints; change in 6MWT and NT-proBNP from baseline to week 52. NT-proBNP is an important diagnostic and prognostic biomarker used to evaluate cardiac severity in ATTR CM patients and increased levels indicate more severe cardiac involvement and poorer prognosis.[35] The 6MWT is a standardised field test to evaluate functional exercise performance, and both NT-proBNP and 6MWT have important prognostic value for death and CV hospitalisation in patients with chronic heart failure[36] and ATTR CM[35, 37]. To support the primary endpoints, a supportive secondary endpoint, change in ECV will be of relevance as it is a robust marker of cardiac interstitium infiltration and extracellular volume and remains an independent predictor of prognosis in ATTR CM after adjusting for known predictors and is the earliest disease marker to track amyloid regression.[38] Additional context will be provided by other supportive secondary endpoints including safety and tolerability, functional endpoints (KCCQ and change in NIS), all-cause mortality and cardiovascular events (CV hospitalisations and urgent heart failure visits), complemented with troponin I and an additional imaging endpoint (global longitudinal strain on echocardiography).

An intervention period of 52 weeks will provide robust data to evaluate the full effect of NNC6019-0001. An interim evaluation is planned at week 24 to possibly enable early selection of the dose to be investigated in phase 3 (see Section 9.4).

In the FHD study (study NN6019-4965), i.v. infusions of NNC6019-0001 administered Q4W were generally safe and well-tolerated at all dose levels tested (doses of 0.1, 0.3, 1.0, 3.0, 10 and 30 mg/kg). No treatment related serious TEAEs, life-threatening events, deaths, or dose limiting toxicities were reported. Due to the introduction of the 60 mg/kg dose in the present study (see Section 4.3) and the serious nature of the disease, a sentinel cohort is applied for participant safety. The sentinel cohort allows for assessments of acute safety of minimum 3 participants per intervention group to ensure the safety of participants before randomising the remaining participants into the main cohort.

4.3 Justification for dose

Doses of 10 mg/kg and 60 mg/kg are selected for this study. Study intervention will be infused intravenously Q4W. The $t_{½}$ of NNC6019-0001 is approximately 1 month, supporting Q4W dosing.

In the FHD study (study NN6019-4965), i.v. infusions of NNC6019-0001 Q4W at dose levels of 0.1, 0.3, 1.0, 3.0, 10 and 30 mg/kg were tested in patients with hATTR and circulating misTTR levels were measured (n=3 for each dose up to 10 mg/kg, and n=6 for the 30 mg/kg dose). There was no clear effect of the lower doses (0.1, 0.3, 1.0 and 3.0 mg/kg) on the relative reduction in circulating levels of misTTR during month 3 compared to baseline.[39] However, an effect was observed for the 10 and 30 mg/kg doses. There was no clear indication that the maximum level of misTTR reduction with NNC6019-0001 had been reached, and doses above 30 mg/kg may have a larger effect. No dose-limiting toxicities were observed in the FHD study, and there was no apparent relationship between dose and AEs, indicating that doses above 30 mg/kg may be well tolerated.

NNC6019-0001 is expected to bind to and reduce both circulating misTTR in plasma and accumulated TTR amyloid fibrils in the myocardium (see Section 2.1). The effect on TTR amyloid fibrils in the myocardium is anticipated to be the most important, disease-modifying effect of NNC6019-0001. However, this effect is difficult to measure, and was not directly measured in the FHD study. The doses for this study were instead selected based on a PK/PD model using the reduction in circulating levels of misTTR (from the first human dose study of NNC6019-001), and two correcting factors. The two correcting factors are applied to translate the effect on circulating misTTR to a predicted effect on accumulated TTR amyloid fibrils in the myocardium.

The first correcting factor is applied to adjust for the much greater apparent binding affinity of NNC6019-0001 to aggregated misTTR compared to monomeric misTTR. The greater apparent binding affinity appears to be driven primarily by avidity effects. This means that lower concentrations are required to have an effect on the aggregated misTTR compared to what a model would predict without this factor. For further details, please refer to current IB[32] and any updates hereof.

The second correcting factor is applied to adjust for the lower expected concentration of NNC6019-0001 in cardiac tissue versus plasma (expected to be ~10% of plasma concentration).[40] The 60 mg/kg dose is selected to ensure maximum effect is reached. The dose is below what was defined as no observed adverse-effect level (NOAEL) which was 300 mg/kg. The 10 mg/kg dose is selected to further explore the dose-response.

4.4 End of study definition

The end of the study is defined as the date of the last visit of the last participant in the study globally.

A participant is considered to have completed the study if he/she has completed all periods of the study including the last visit. If a randomised participant has died during study, ‘date of study completion’ is the date of death.

The primary endpoints are evaluated at visit 15 (week 52). The primary completion date (PCD) is defined as the date of visit 15 (week 52) on which the last participant in the clinical study has an assessment for the primary endpoints. If the last participant is withdrawn early, the PCD is considered the date when the last participant would have completed visit 15.

5 Study population

Prospective approval of protocol deviations to recruitment and enrolment criteria, also known as protocol waivers or exemptions, is not permitted.

Pre-screening is defined as review of the patient medical records, including handing out participant information, as well as database review. Any pre-screening activities must be documented on site by the investigator.

All eligibility criteria related to laboratory assessments should be based on central laboratory data, unless otherwise stated.

5.1 Inclusion criteria

Participants are eligible to be included in the study only if all the following criteria apply:

1. Informed consent obtained before any study-related activities. Study-related activities are any procedures that are carried out as part of the study, including activities to determine suitability for the study.
2. Male or female.
3. Age ≥ 18 to < 85 years at the time of signing informed consent.
4. Have an established diagnosis of ATTR CM with either wild-type TTR or hereditary TTR genotype as per local standards[a].[7]
5. Expected to be on stable doses of cardiovascular medical therapy 6 weeks prior to the randomisation visit.
6. Known[b] end-diastolic interventricular septal wall thickness ≥12 mm.
7. Presently classified as New York Heart Association (NYHA) Class II-III.
8. NT-proBNP concentration ≥650 pg/mL in sinus cardiac rhythm and >1000 pg/mL in atrial fibrillation at screening.
9. Completed ≥150 meters to ≤450 meters on the 6MWT at screening.
10. Absolute neutrophil count $\geq 2.0 \times 10^9$/L; platelet count $\geq 120 \times 10^9$/L at screening.
11. Aspartate transaminase (AST) and alanine transaminase (ALT) levels ≤ 2.5 × the upper limit of normal (ULN) and total bilirubin ≤2 × ULN at screening.
12. Estimated glomerular filtration rate (eGFR) ≥25 mL/min/1.73 m^2 at screening.

5.2 Exclusion criteria

Participants are excluded from the study if any of the following criteria apply:

1. Known or suspected hypersensitivity to study intervention(s) or related products.
2. Previous dosing in this study
3. Female who is pregnant, breast-feeding or intends to become pregnant or is of childbearing potential and not using highly effective contraceptive method, as defined in Appendix 4 (Section 10.4).
   Spain: For country-specific requirements, please refer to Appendix 8 (Section 10.8).
4. Use of another approved or non-approved investigational medicinal product within 30 days or 5 half-lives of the investigational medicinal product (whichever is longer) before screening.
5. Any disorder, which in the investigator's opinion might jeopardise participant's safety or compliance with the protocol.
6. Current diagnosis or history of amyloid light chain or other non-ATTR amyloidosis

7. Cardiomyopathy not primarily caused by ATTR CM, for example, cardiomyopathy due to hypertension, valvular heart disease, or ischemic heart disease.
8. A prior solid organ transplant.
9. Planned solid organ transplant during the study.
10. Presence or history of malignant neoplasm (other than basal or squamous cell skin cancer, in-situ carcinomas of the cervix, or in-situ/high grade prostatic intraepithelial neoplasia (PIN) or low-grade prostate cancer) within 5 years before screening.
11. Current treatment with calcium channel blockers with conduction system effects (e.g., verapamil, diltiazem). The use of dihydropyridine calcium channel blockers is allowed. The use of digoxin will only be allowed if required for management of atrial fibrillation with rapid ventricular response.
12. Acute coronary syndrome, unstable angina, stroke, transient ischemic attack (TIA), coronary revascularisation, cardiac valve repair, or major surgery within 3 months of screening.
13. Body weight > 120 kg (264.6 lb) at screening.
14. Evidence of current or chronic hepatitis C virus or hepatitis B virus infection.
15. History of or known seropositivity for human immunodeficiency virus (HIV).
16. International normalised ratio (INR)>1.5 (unless participant is on anticoagulant therapy[c], in which case excluded if INR>3.5)[d].
17. History of contrast allergy or adverse reactions to gadolinium-containing agents.

Definitions:
[a] Non-invasive diagnostic pathway will be confirmed by a centralised expert review, see Section 8.1; [b] Medical history/records are accepted; [c] Vitamin K antagonists i.e. warfarin, acenocoumarol etc.; [d] Criterion not applicable for participants on therapy with direct-acting oral anticoagulants (DOACs).

5.3 Lifestyle considerations

5.3.1 Activity

For participants, especially in the sentinel cohort, the quantity or types of physical activity may be limited during the cardiac monitoring periods (Holter ECG) pre- and post-dosing, as per local requirements (with regards to potential electrode displacement and water resistance of the device used). Dosing will take place at the site as an infusion and physical activity may be limited during the infusion and subsequent observation time (Section 6.1).

5.4 Screen failures

A screen failure occurs when a participant who consents to participate in the clinical study is not subsequently eligible for participation according to the inclusion/exclusion and randomisation criteria. Visit 1A is not allowed if an eligibility criterion is failed at Visit 1. A screen failure must be registered in the system (Randomisation and Trial Supplies Management System [RTSM] / Interactive Web Response System [IWRS]).

If participants withdraw their consent prior to randomisation or do not return for randomisation, a screen failure must be registered in the RTSM/IWRS. The reason for failure will in all cases be captured in the electronic case report forms (eCRF).

A minimal set of screen failure information is required to ensure transparent reporting of screen failure participants to meet requirements from regulatory authorities. Minimal information includes informed consent date, demography, screen failure details, and eligibility criteria.

Individuals who do not meet the criteria for participation in this study may be rescreened if the investigator assesses it is reasonable to expect that potential changeable or fluctuating in- or exclusion criteria may change, e.g., biochemical parameters. However, previously dosed participants cannot be rescreened.

If the participant has failed one of the inclusion criteria or fulfilled one of the exclusion criteria related to laboratory parameters, re-sampling is not allowed, unless participant is re-screened. In case of technical issues with laboratory sample(s) collected for eligibility assessment (e.g., haemolysed or lost) re-sampling is allowed for the affected laboratory parameter(s). This is not considered rescreening.

Individuals who are rescreened are required to sign a new informed consent form and provided with a new subject ID. A new screening must be registered in the RTSM/IWRS.

5.5 Randomisation criteria

For all participants: To be randomised, the following randomisation criterion must be answered "yes":

- On stable doses of cardiovascular medical therapy within the last 6 weeks prior to randomisation.

Sentinel cohort

For participants in the sentinel cohort, the following randomisation criteria must be answered "no":

1. Sinus pauses >3 seconds in the day or sinus pauses >5 seconds at night during the 48 hours outpatient cardiac monitoring prior to the randomisation visit.
2. Arrhythmia requiring treatment diagnosed during the 48 hours out-patient cardiac monitoring prior to the randomisation visit.

6 Study interventions and concomitant therapy

Study intervention is defined as any investigational intervention(s), marketed product(s), placebo, or medical device(s) intended to be administered to a study participant according to the study protocol.

Trial product comprise investigational medicinal products (IMPs), including placebo and comparators, non-investigational medicinal products (NIMPs) and/or investigational medical devices.
In this protocol:

- Trial products consist of IMPs (NNC6019-0001 and placebo), but NIMPs are not considered trial products.
- In situations where trial product is referring only to vials with NNC6019-0001, e.g., when discussing trial product accountability, this is specified.

6.1 Study interventions administered

Table 6-1 provides an overview of the study interventions in the study.

Table 6-1 Study interventions

**Study intervention name**	**10 mg/kg NNC6019-0001**	**60 mg/kg NNC6019-0001**	**Placebo**	**Other interventions**	
Intervention name	NNC6019-0001		Placebo (saline[a])	Pre-medication	Post-dose flushing (saline[a])
Intervention type	IMP, test product		IMP, reference therapy	NIMP, pre-medication	NIMP, post-dose flushing
Pharmaceutical form	Lyophilised powder for solution for infusion<br>Reconstituted with SWFI[b] and diluted with normal saline[a]		Sterile solution for infusion	Tablet or capsule	Sterile solution
Route of administration	Intravenous			Oral	Intravenous
Trial product strength	250 mg NNC6019-0001 supplied as a sterile, lyophilised dosage form in 20 mL vials		0.9% sodium chloride solution	25 mg diphenhydramine (or an equivalent dose of an H1 antihistamine) and 650-1000 mg acetaminophen/paracetamol [c]	0.9% sodium chloride solution
Dose and dose frequency	Every 4 weeks (Q4W)			30 to 90 minutes prior to start of infusion with NNC6019-0001 or placebo	Post-dose flushing
Dosing instructions and administration	See description of Dosing instructions/administration below			As per site standard	As per site standard
Sourcing	Lyophilised powder is supplied by Novo Nordisk A/S		Provided locally by study site	Provided locally by study site	
Packaging and labelling	Lyophilised powder is labelled and packaged by Novo Nordisk A/S. Labelled in accordance with EU GMP, Volume 4, Annex 13,[41] local regulations and study requirements		250 mL infusion bags for diluent containers as per standards defined in the TMM	No protocol specific requirements	

[a] Normal Saline: USP, Ph.Eur., BP or as per local pharmacopeia specification is provided locally by study site.
[b] SFWI = Sterile water for injection USP, Ph. Eur, BP or local pharmacopeia specification. Sterile water for injection is provided locally by study site in glass or plastic containers for single use as per site standards for reconstitution of products. Preparation of doses will be described in the Trial Master Manual (TMM). [c]Japan: For country specific requirements, please refer to Appendix 8 (Section 10.8).

Investigational medicinal products (IMP)

The IMPs are listed in Table 6-1.

Dosing instructions/administration

Trial product should only be administered in settings where emergency resuscitative equipment and personnel trained in the management of anaphylaxis are immediately available to treat systemic reactions under the direct supervision of a physician. Trial product will be administered Q4W (±4-day window allowed starting with second dose) as a 2 or 3-hour i.v. infusion. A minimum of 20 days is required between infusions. Each participant's initial dose will be delivered over 3 hours (±10 minutes), followed by a ~30 mL saline flush (Table 6-2). If the first dose is tolerated without a hypersensitivity-associated AE, subsequent infusions may be delivered over 2 hours (±10 minutes), followed by a ~30 mL saline flush. At each dosing visit, participants will be premedicated with 25 mg diphenhydramine (or an equivalent dose of an H1 antihistamine) and 650-1000 mg acetaminophen/paracetamol 30 to 90 minutes prior to start of infusion with trial product.

Table 6-2 Timing of infusion and measurement of vital signs

	Infusion time	Observation time	Measurement of vital signs during observation time
**Sentinel cohort[a]**			
1st dose	3 hours<br>(±10 minutes)	24 h (± 4h) in-house/admitted[b]	Before infusion<br>Halfway through infusion<br>At end of infusion<br>After infusion: 10 min (±1 min), 30 min (±10 min), 60 min (±10 min), 2 hours (±15 min) , 3 hours (±15 min), 4 hours (±20 min), 8 hours (±20 min), and 24 hours (±4 hours)
2nd dose and on	2 hours<br>(±10 minutes)	90 minutes<br>(±10min)	Before infusion<br>Halfway through infusion<br>At end of infusion<br>After infusion: 10 min (±1 min), 90 min (±10 min)
**Main cohort**			
1st dose	3 hours<br>(±10 minutes)	4 hours (±20min)	Before infusion<br>Halfway through infusion<br>At end of infusion<br>After infusion: 10 min (±1 min), 2 hours (±15 min), and 4 hours (±20 min)
2nd dose and on	2 hours<br>(±10 minutes)	90 minutes<br>(±10min)	Before infusion<br>Halfway through infusion<br>At end of infusion<br>After infusion: 10 min (±1 min), 90 min (±10 min)

Note: Czech Republic and Japan: For country specific requirements, please refer to Appendix 8 (Section 10.8).

[a]Also applicable for the first 6 randomised Japanese participants. [b] Discharge participant from clinic if no immediate safety concerns and/or hypersensitivities are present after the post-dose assessments and 24-hour cardiac monitoring period. In the event of any clinical concerns or suspicious signs or symptoms after the infusion, the participant will remain with the Investigator and study staff for further observation until the Investigator deems the participant can safely leave the clinic.

Sentinel cohort

Due to the introduction of the 60 mg/kg dose (see Section 4.3) and the serious nature of the disease, a sentinel cohort is applied for participant safety.

The sentinel cohort, consisting of minimum 9 participants, will be subjected to intensified cardiac monitoring during the first 28 days of the study. Participants in the sentinel cohort will undergo cardiac monitoring on an out-patient basis for at least 48 hours within 2 weeks prior to the randomisation visit to ensure that the eligibility criteria for participation in the study are met.

After randomisation, 1 participant in each of the intervention groups will receive the first dose of study intervention (1 participant will receive 10 mg/kg NNC6019-0001, 1 participant will receive 60 mg/kg NNC6019-0001, and 1 participant will receive placebo) on an in-patient basis and will be observed under continuous cardiac monitoring for at least 24 hours after start of infusion. On discharge, participants will be monitored using continuous cardiac monitoring on an out-patient basis until 7 days after start of infusion.

Dosing of the remaining 6 participants in the sentinel cohort will be initiated following safety evaluation of blinded relevant safety data including AEs, vital signs, ECGs and cardiac monitoring evaluated by investigator, and laboratory parameters for the initial 3 participants. The evaluation of blinded safety data and decision to continue dosing in the sentinel cohort will be performed by an internal Novo Nordisk medical monitoring group at a dedicated meeting after completing 7 days of post-dose cardiac monitoring for the initial 3 participants. The sentinel cohort design is illustrated in Figure 6-1.

Figure 6-1 Sentinel cohort design

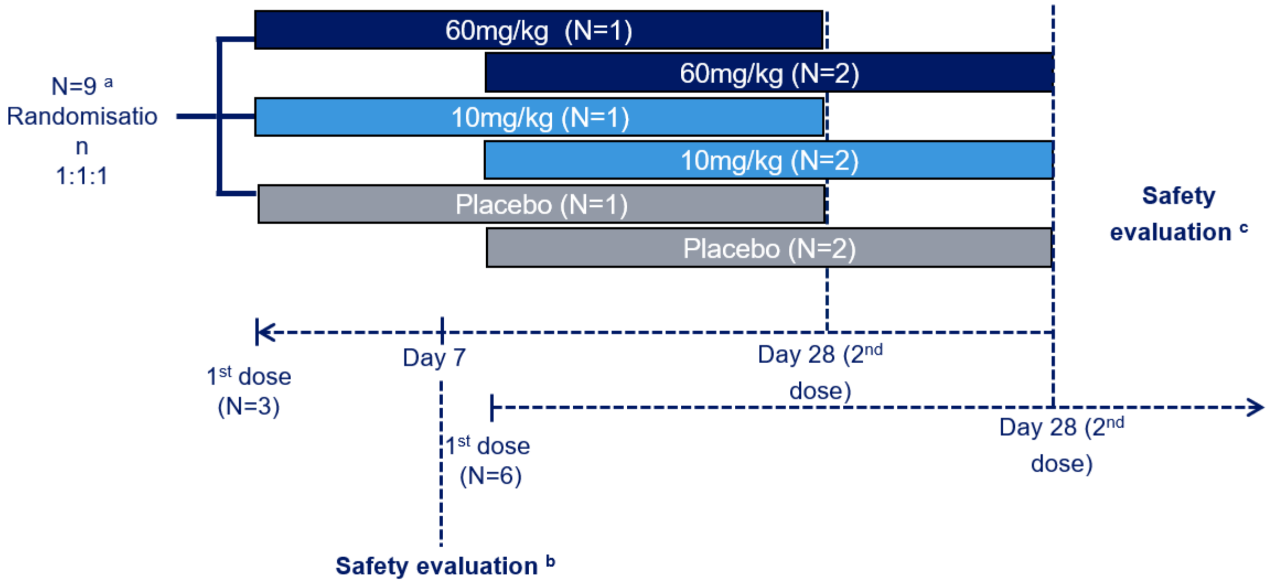

[a] The sentinel cohort will consist of minimum 9 participants, [b] Evaluation of blinded safety data and decision to continue dosing in the sentinel cohort will be performed by an internal Novo Nordisk medical monitoring group, [c] Evaluation of unblinded safety data for minimum 9 participants in the sentinel cohort will be performed by an external Data Monitoring Committee.

In case of reporting of an SAE with a possible or probable relation to the trial product, the Novo Nordisk NNC6019-0001 Safety Committee will be informed immediately and will discuss at a separate meeting if the dosing should be paused, and any action on individual or study level should be taken towards current ongoing treatment or follow-up investigations.

In addition, an external Data Monitoring Committee (see Appendix 1, Section 10.1.6) will evaluate unblinded data for the minimum 9 participants in the sentinel cohort at a separate meeting and will give their recommendation for the continued conduct of study. Minimum 9 participants in the sentinel cohort must have attended visit 3 before the Data Monitoring Committee evaluation can take place and before randomising further participants into the main cohort. The Data Monitoring Committee will continue to monitor the study based on unblinded data until study closure.

Participants in the sentinel cohort who do not attend visit 3 will be replaced, and a new participant will be allocated to the same treatment. Additionally, participants in the sentinel cohort must have an ECG and echocardiography evaluated by the investigator before the planned second dosing.

Continuous cardiac monitoring may be performed at subsequent dosing visit as clinically indicated or at the discretion of the investigator.

Japan: For country-specific requirements, please refer to Appendix 8 (Section 10.8).

Non-investigational medicinal products (NIMP)

The NIMPs are listed in Table 6-1.

Auxiliary supplies including medical device(s) not under investigation

Novo Nordisk will provide infusion/blinding cover bags to EU countries.

Other auxiliaries needed for preparation and administration of each dose will not be provided by Novo Nordisk.

6.2 Preparation, handling, storage and accountability

Only participants enrolled in the study may use study intervention and only delegated site staff may administer study intervention.

Each site will be supplied with sufficient trial product (vials with NNC6019-0001) for the study on an ongoing basis according to recruitment and randomisation.

Receipt of trial product (vials with NNC6019-0001) shipments, storage, and preparation including blinding of trial product, accountability and disposition records will be maintained by investigator´s unblinded designee.

Each vial with NNC6019-0001 will be reconstituted with 4.9 mL sterile water for injection (SWFI) to a concentration of 50 mg/mL. The reconstituted solution will be diluted in normal saline for infusion (total volume 250 mL). The total volume infused will be ~280 mL including the saline flush volume of ~30 mL. Details on the preparation procedures are described in the Trial Materials Manual (TMM).

Body weight is measured at every visit and the latest available body weight is used for calculating the dose. Number of vials with NNC6019-0001 needed for each dose will be calculated based on body weight. Dose must be calculated based on a body weight of maximum 120 kg (264.6 lb). If a participant's body weight increases above that during the study, the dose calculation must remain based on 120 kg (264.6 lb).

Administration of blinded trial product to study participants will be done by blinded site staff. Blinded site staff will document in source if the fully prepared dose was administrated to the participant.

Acceptable temperature ranges and conditions for storage and handling of trial product (vials with NNC6019-0001) when not in use and when in use are described in the TMM.

The investigator's unblinded designee must confirm that appropriate temperature conditions have been maintained during transit for all trial product (vials with NNC6019-0001) received, and that any discrepancies are reported and resolved before use of the trial product (vials with NNC6019-0001).

All trial product (vials with NNC6019-0001) must be stored in a secure, controlled, and monitored (manual or automated) area in accordance with the labelled storage conditions with access limited to investigator´s unblinded designee.

The investigator's unblinded designee must inform Novo Nordisk immediately if any trial product (vials with NNC6019-0001) has been stored outside specified conditions. The trial product (vials with NNC6019-0001) must not be dispensed to any participant before it has been evaluated and approved for further use by Novo Nordisk. Additional details regarding handling of temperature deviations can be found in the TMM.

The investigator's unblinded designee is responsible for trial product (vials with NNC6019-0001) accountability and record maintenance (i.e., receipt, accountability, and final disposition records). Trial product (vials with NNC6019-0001) accountability must be performed by investigator´s unblinded designee using a trial product accountability log and must be registered in the RTSM/IWRS. To avoid accidental site/pharmacy staff exposure to trial product, accountability of infusion bags will not be done as these containers are single-use and not intended for longer-term storage. Infusion bags should be discarded immediately after use together with the infusion kit.

Destruction of trial products (vials with NNC6019-0001) can be performed on an ongoing basis and will be done according to local procedures after accountability is finalised by the investigator´s unblinded designee and reconciled by the unblinded monitor.

All vials should be saved to allow for trial product reconciliation by the unblinded monitor, unless not permissible per site standard operating procedure (SOP). The site SOP should outline the local process for accountability documentation and destruction policy. The unblinded monitor should perform the reconciliation of the trial product based on the site accountability records.

All expired or damaged trial products (vials with NNC6019-0001) including package (for technical complaint samples, see Appendix 5 [Section 10.5]) must be stored separately from non-allocated

trial product (vials with NNC6019-0001) by the investigator´s unblinded designee. No temperature monitoring is required.

Non-allocated trial product (vials with NNC6019-0001), including expired or damaged products, must be accounted as unused, at the latest at closure of the site by the investigator´s unblinded designee.

Instructions for auxiliary supplies are described in the TMM.

Japan: For country-specific requirements, please refer to Appendix 8 (Section 10.8).

6.3 Measures to minimise bias: Randomisation and blinding

6.3.1 Randomisation

This is a randomised, placebo-controlled study. Participants will be randomised 1:1:1 to intervention groups as illustrated in Figure 4-1. Participants will be stratified by disease type (wtATTR vs hATTR) and maximum 80% of participants randomised will be participants with wtATTR. Within each stratum, each participant will be randomly allocated to one of the intervention groups.

Sentinel cohort: Stratification by disease type will not be applied for participants in the sentinel cohort. This is in order to ensure the 1:1:1 distribution to the different intervention groups with the small participant number. Japan: For country-specific requirements, please refer to Appendix 8 (Section 10.8).

All participants will be screened and centrally randomised using the RTSM/IWRS and assigned to the next available treatment according to the randomisation schedule. Trial product will be allocated by the RTSM/IWRS and dispensed and prepared for administration by investigator´s unblinded designee at the study visits summarised in the flowchart (Section 1.2).

At screening, each participant will be assigned a unique 6-digit number which will remain the same throughout the study. Each site is assigned a 3-digit number and all subject IDs will start with the site number. Subject IDs must not be re-assigned.

6.3.2 Blinding

This is a double-blind study in which participants, care providers, investigators and outcome assessors are blinded to trial product allocation. To preserve the blinding of the study in the event of interim evaluation, only a minimum number of Novo Nordisk personnel are allowed to see the randomisation table and intervention assignments before the study is completed (see Section 9.4 for further details).

Investigators and other site staff with participant contact will remain blinded throughout the course of the study. In order to maintain this blind, unblinded delegated site staff or pharmacy staff not otherwise involved in the study procedures will be responsible for the shipment receipt, storage, returns, accountability and destruction of trial product (vials with NNC6019-0001) and dispensing, preparation, and blinding of trial product. See further details in Section 6.2 and in the TMM.

In the event of a Quality Assurance audit, the auditor(s) will be allowed access to unblinded trial product records at the site/pharmacy to verify that randomisation/dispensing has been done accurately.

6.3.3 Blind Break

The RTSM/IWRS is used for blind-breaking. In case of an emergency, the investigator has the sole responsibility for determining if unblinding of a participant's trial product is warranted. Participant safety must always be the first consideration in making such a determination. If the investigator decides that unblinding is warranted, the investigator should make every effort to contact Novo Nordisk prior to unblinding a participant's study intervention unless this could delay emergency treatment of the participant.

If a participant's trial product is unblinded, Novo Nordisk (Global Safety department) must be notified within 24 hours after breaking the blind. The date and reason that the blind was broken must be recorded in the source documentation. The person breaking the blind must print the blind break confirmation notification generated by the RTSM/IWRS, sign and date the document. If RTSM/IWRS is not accessible at the time of blind break, the RTSM/IWRS helpdesk should be contacted. Contact details are listed in Attachment I.

If there are no safety concerns at the discretion of the investigator, the participant may continue trial product.

Trial product allocation will also be accessible to:
- The laboratory responsible for analysis of NNC6019-0001 anti-drug antibodies, and the responsible analytical scientist at special lab.
- The Novo Nordisk laboratory responsible for analysis of NNC6019-0001 PK and the responsible analytical scientist.
- The Novo Nordisk unblinded monitor.

6.4 Study intervention compliance

Drug treatment compliance and compliance with other interventions

Throughout the study, the investigator will remind the participants to follow the study procedures and requirements to encourage participant compliance.

The participants are dosed at the site. They will receive trial product directly from the investigator or designee, under medical supervision. The date and start and stop time of each dose administered at the site will be recorded in the source documents and eCRF. For guidance in the event of overdose, please refer to Section 6.7.

Site staff will observe ingestion of the pre-medication as per Table 6-1. If in doubt whether the pre-medication has been swallowed, site staff will enter into a dialogue with the participant, acknowledging non-compliance can happen and addressing barriers to compliance. Pre-medication should be entered as concomitant medication in the eCRF.

6.5 Dose modification

Not applicable for this study.

6.6 Continued access to study intervention after end of study

When discontinuing study intervention, the participant should be transferred to a suitable marketed product at the discretion of the investigator.

6.7 Treatment of overdose

There is no previous experience of an overdose with NNC6019-0001 in humans. Any dose of NNC6019-0001 greater than i.v. 10 mg/kg or 60 mg/kg Q4W, depending on the treatment group, and/or less than 20 days apart will be considered an overdose.

Treatment of overdose with NNC6019-0001 should consist of general supportive measures, if applicable. There is no known specific antidote for overdose with NNC6019-0001.

Accidental overdose must be reported as a medication error. Intentional overdose must be reported as misuse and abuse, please refer to Section 8.4 and Appendix 3 (Section 10.3.3) for further details.

In the event of an overdose, the investigator should closely monitor the participant for overdose-related AEs/SAEs The length of observation and treatment should be guided by the clinical picture and medical judgment.

Decisions regarding dose interruptions will be made by the investigator based on the clinical evaluation of the participant.

For more information on overdose, also consult the current version of the NNC6019-0001 investigator's brochure (IB)[32] or updates thereof.

6.8 Concomitant therapy

NNC6019-0001 will be used on top of standard-of-care treatment. Changes in standard-of-care treatment should be avoided and increase in dose or addition of concomitant medication with TTR-targeted treatment such as patisiran or tafamidis after randomisation will generally not be allowed, unless a change in standard-of-care treatment is indicated based on a deterioration in the clinical condition of the participant as evaluated by the investigator. A worsening of the pre-existing condition will be required to be registered in the AE form.

Additional medications to treat participants´ conditions may be added or changed during the study at the discretion of the investigator and in accordance with local treatment guidelines and policies. Standard-of care treatment is considered background treatment and will not be provided by Novo Nordisk A/S.

Treatment with calcium channel blockers with conduction system effects (e.g., verapamil, diltiazem) or digoxin not prescribed for management of atrial fibrillation with rapid ventricular response is contraindicated in ATTR CM and is not allowed during study conduct (see Sections 5.2 and 7.1).

Any medication or vaccine (including over the counter or prescription medicines, that the participant is receiving at the time of the first visit or receives until end of study must be recorded along with:

- Trade name or generic name
- Primary indication
- Dates of administration including start and stop dates
- Dose and unit, frequency, route of administration

Changes in concomitant therapy must be recorded at each visit. If a change is due to an AE, then this must be reported according to Section 8.4.

7 Discontinuation of study intervention and participant discontinuation/withdrawal

Discontinuation of specific sites or of the study as a whole is detailed in Appendix 1 (Section 10.1.11).

7.1 Discontinuation of study intervention

Study intervention may be discontinued at any time during the study at the discretion of the participant or at the discretion of the investigator for safety, behavioural, compliance or administrative reasons.

Efforts must be made to have participants who discontinue study intervention continue the planned visit schedule and assessments. As a minimum, efforts must be made to have those participants attend the scheduled milestone visits (Visit 3, Visit 5, Visit 8 and Visit 15) on site to ensure continued counselling and data collection. Participants should be informed about the continued scientific importance of their data, even if they discontinue study intervention. Only participants who withdraw consent will be considered as withdrawn from the study. The follow-up visit (Visit 16) should be scheduled 16 weeks after last dose of study intervention. If this coincides with a scheduled visit, assessments pertaining to visit 16 should be performed in addition to the visit assessments. See the flowchart for data to be collected at the milestone visits and follow-up visit and for any further evaluations that need to be completed (Section 1.2).

If the participant does not wish to attend the scheduled visits, efforts should be made to have remaining visits converted to remote contacts, carrying out the protocol assessments deemed feasible to conduct remotely. If a participant is unwilling to attend remaining visits, information about the attempts to follow up with the participant should be documented in the participant's medical record.

The study intervention must be discontinued, if any of the following applies for the participant:
1. Pregnancy
2. Intention of becoming pregnant
3. Initiation of treatment with calcium channel blockers with conduction system effects (e.g., verapamil, diltiazem) or digoxin not required for management of atrial fibrillation with rapid ventricular response
4. Solid organ transplant during the study
5. Simultaneous use of an approved or non-approved investigational medicinal product in another clinical study
6. Safety concern as judged by the investigator

If a participant meets a discontinuation criterion judged by the investigator as reversible, study intervention can be resumed when the discontinuation criterion is no longer met, unless any of the following becomes applicable for the participant in the meantime:
- participant met a discontinuation criterion that is judged by the investigator as irreversible
- participant missed 3 consecutive doses of study intervention

The primary reason for discontinuation of study intervention must be specified in the eCRF, and final trial product (vials with NNC6019-0001) accountability must be performed once the participant has permanently discontinued study intervention. Treatment discontinuation must be registered in the RTSM/IWRS.

7.1.1 Temporary discontinuation of study intervention

If a participant discontinued study intervention temporarily, study intervention can be resumed if considered safe at the investigator's discretion and if the participant does not meet any of the discontinuation criteria (Section 7.1). Similarly, a participant who discontinues study intervention on their own initiative should be encouraged to resume the study intervention if deemed safe by the investigator (see Section 6.1).

A treatment discontinuation and treatment resume must be registered in RTSM/IWRS when a participant discontinues or resumes study intervention. Missed doses are recorded in the eCRF.

7.2 Participant discontinuation/withdrawal from the study

A participant may withdraw consent at any time at his/her own request.

If a participant withdraws consent prior to randomisation, the participant will not be asked to have any follow-up assessments performed. The following data must be collected: Demography, eligibility criteria, date of informed consent, date of screening and the date when participant's participation ended. The end of study form must be completed in eCRF.

If a participant withdraws consent between randomisation and prior to first dosing administration, the participant will not be asked to have any follow-up assessments performed. This withdrawal of consent should be captured in the first dose after randomisation form and the end of study form in eCRF.

If a participant withdraws consent after receipt of study intervention, the investigator must ask the participant if he/she is willing, as soon as possible, to have assessments pertaining to safety performed according to Visit 16. See the flowchart for data to be collected.

Discontinuation of study intervention must be registered in the RTSM/IWRS.

If the participant withdraws consent, Novo Nordisk may retain and continue to use any data collected before such a withdrawal of consent for the purpose of the study or scientific research.

If a participant withdraws from the study, the participant may request destruction of any samples taken and not tested, and the investigator must document this in the medical record and inform Novo Nordisk that samples should be destroyed.

Although a participant is not obliged to give his/her reason(s) for withdrawing, the investigator must make a reasonable effort to ascertain the reason(s), while fully respecting the participant's rights. Where the reasons are obtained, the primary reason for withdrawal must be specified in the eCRF.

7.2.1 Replacement of participants

Sentinel cohort: If a sentinel participant discontinues study intervention or withdraws consent before attending visit 3, the participant will be replaced unless procedures described in Appendix 3 (Section 10.3) are applicable. Sentinel participants will not be replaced if they discontinue study intervention/withdraws consent at or after Visit 3.

If a non-sentinel participant discontinues study intervention or withdraws consent after first dose (Visit 2), the participant will not be replaced.

If a non-sentinel participant withdraws consent or is lost to follow up prior to first dose administration (Visit 2), the participant will be replaced.

7.3 Lost to follow-up

A participant will be considered lost to follow-up if he/she repeatedly fails to return for scheduled visits and is unable to be contacted by the site.

The following actions must be taken if a participant fails to return to the site for a required visit:

- The site must attempt to contact the participant and reschedule the missed visit as soon as possible and counsel the participant on the importance of maintaining the assigned visit schedule and ascertain whether the participant wishes to and/or should continue in the study.
- Before a participant is deemed lost to follow-up, the investigator or designee must make every effort to regain contact with the participant (where possible, at least three telephone calls and, if necessary, a certified letter to the participant's last known mailing address or local equivalent methods). These contact attempts should be documented in the participant's source document.
- Should the participant continue to be unreachable when visit 15 is due, the participant will be considered to have withdrawn from the study with a primary reason of 'lost to follow-up'.
- Site personnel, or an independent third party, will attempt to collect the vital status of the participant within legal and ethical boundaries for all participants randomised. Public sources may be searched for vital status information. If vital status is determined as deceased, this will be documented, and the participant will not be considered lost to follow-up. Sponsor personnel will not be involved in any attempts to collect vital status information.

If a participant is lost to follow-up between randomisation and prior to first dosing administration, the above listed actions are not required. The lost to follow-up should be captured in the first dose after randomisation form and the end of study form in eCRF.

8 Study assessments

The following sections describe the assessments and procedures, while their timing is summarised in the flowchart.

The following general assessments and procedures must be followed in the study:

- Informed consent must be obtained before any study-related activity, see Appendix 1 (Section 10.1.3).
- All screening evaluations must be completed and reviewed to confirm that potential participants meet all inclusion criteria and none of the exclusion criteria. Note: results from both Visit 1 and Visit 1A, when applicable, should fulfil the eligibility criteria.
- The investigator will maintain a screening log to record details of all participants screened and rescreened and to confirm eligibility or record reason for screen failure, as applicable.
- At screening, participants will be provided with a card stating that they are participating in a study and giving contact details of relevant site staff that can be contacted in case of emergency.
- Adherence to the study design requirements, including those specified in the flowchart, is essential and required for study conduct.
- Assessments should be performed according to the standard of care unless otherwise specified in the current section, and all assessments should preferably be performed on the same day. Efforts should be made to limit bias between assessments. When all assessments are performed on the same day, efforts should be made to perform the assessments in the following order:
  1. Clinical outcome assessments (COAs); the following patient reported outcome (PRO) questionnaires are suggested to be performed in the following order:
     - EQ-5D-5L
     - PGI-S for KCCQ
     - PGI-C for KCCQ (when applicable)
     - KCCQ
  2. Electrocardiogram (ECG) and vital signs
  3. Blood sampling
  4. Clinical outcome assessments; the following assessments are suggested to be performed in the following order:
     - 6MWT (must be performed after ECG, first vital sign assessment and blood sampling)
     - PGI-S for walking
     - PGI-C for walking (when applicable)
     - NIS (when applicable)
  5. Other assessments
- If all assessments pertaining to a visit cannot be performed on the same day, it is allowed to split the visit across 2 or more days, provided that all assessments are completed prior to dosing and within the given visit window. The suggested order of the COAs, including the PRO questionnaires as listed above, remains applicable. Specific visit window allowances are applicable to the cardiac MRI and echocardiography. Refer to Section 8.2.3.1 and 8.2.3.2 for the specific visit window details related to the cardiac MRI and echocardiography, respectively.
- First dose must only be administered after assessments related to primary and/or secondary endpoints are completed (e.g., blood samples taken, echocardiography, cardiac MRI and PRO questionnaires completed).

- Review of PRO instruments, ECG, laboratory reports, etc., must be documented in the source documents or the participant's medical record. If clarification of entries or discrepancies in the PRO instruments is needed, the participant must be questioned, and a conclusion made in the participant's source documents. Care must be taken not to bias the participant.
- Repeat samples may be taken for technical issues and unscheduled samples or assessments may be taken for safety reasons. Please refer to Appendix 2 (Section 10.2) for further details on laboratory samples.
- Participants are not required to attend any visits in a fasting state. The time since last meal is collected when blood samples are taken, as applicable.

8.1 Screening

Demography

The following information must be recorded after informed consent at the screening visit:

- Date of birth, unless not permitted by local regulations
- Year of birth
- Age (should be recorded in the RTSM/IWRS)
- Sex
- Race, unless not permitted by local regulations
- Ethnicity, unless not permitted by local regulations

Germany, France, Netherlands: For country-specific requirements, please refer to Appendix 8 (Section 10.8).

Tobacco use

Details of tobacco use must be recorded at screening (visit 1). Smoking is defined as smoking at least one cigarette or equivalent daily. The collected information should include whether the participant smokes or has smoked.

Smoking status information to be collected:

- Never smoked
- Previous smoker (smoking stop date)
- Current smoker

Childbearing Potential

The assessment of women's childbearing potential should be performed at the screening visit and recorded as specified in the flowchart (Section 1.2) and Appendix 4 (Section 10.4).

Documentation can come from the site staff's review of participant's medical records, medical examination, or medical history interview. Documentation of women's child-bearing potential must be recorded in the eCRF.

Central review of imaging diagnostic criteria

Information regarding the specific assessments justifying the diagnostic conclusion of ATTR CM should be reported in the eCRF in a customised ATTR CM medical history form. If the diagnosis is

based on non-invasive diagnostic criteria, it will be required to upload technetium-labelled PYP/DPD/HMDP cardiac scintigraphy including SPECT imaging[42] (confirming that cardiac radiotracer uptake corresponds to the myocardium). Approximately 1 to 2 appointed external medical experts will review the criteria and evaluate the scintigraphy/SPECT imaging. In case a participant does not fulfil the pre-defined centralised diagnostic criteria as assessed by the expert, the participant will not be eligible for the study.

8.2 Efficacy assessments

Planned time points for all efficacy assessments are provided in the flowchart (Section 1.2).

8.2.1 Clinical efficacy laboratory assessments

All protocol-required laboratory assessments, as defined in Appendix 2 (Section 10.2), must be conducted in accordance with the flowchart (Section 1.2) and the laboratory manual.

8.2.1.1 Urine collection

Participants must collect first morning void urine samples in accordance with the flowchart. Urine must be collected in the containers which are to be provided at the previous visit. The investigator should remind the participant to collect first morning void urine samples e.g., via a telephone call or a text message.

Participants must collect two first morning void urine samples:
- one day before the visit
- on the day of the visit

If the participant has not collected the required first morning void urine samples, the participant must be asked to provide new samples (within the visit window) to replace the missed samples.

8.2.2 Clinical outcome assessments

The 6MWT must be performed in accordance with the manual provided by Novo Nordisk. The below clinical outcome assessments test will be performed.

8.2.2.1 6-minute walk test (6MWT)

The 6MWT assesses the distance a participant can walk in six minutes. It is a direct and timed measure of walking ability, which is technically simple, reproducible, and when administrators are well trained, readily standardised. The goal is for the participant to walk as far as possible in six minutes without running. The 6MWT must be performed in accordance with the manual provided by Novo Nordisk.

If it is identified at Visit 1 that Visit 1A will be required, the 6MWT may be omitted at Visit 1.

8.2.2.2 Neuropathy Impairment Score (NIS)

Neuropathy impairment score (NIS) is a clinical assessment that tests muscle strength, reflex activity, and sensation of toes and fingers, and can be used to assess neurologic function in hATTR PN over time. The NIS assessment is only applicable for participants with hATTR CM and will be performed by a trained neurologist or a physician trained in using the NIS. The NIS assessment

should be evaluated, signed and dated by the investigator and filed on the participant's medical record. The assessment should be recorded in the eCRF.

The NIS assessment can be performed within ± 2 weeks of the respective visit, except for the first dosing visit where it can be performed up to 2 weeks in advance (full eligibility needs to be confirmed beforehand).

8.2.2.3 Patient reported outcome (PRO) questionnaires

Participants should be given the opportunity to complete the questionnaires by themselves without interruption. Review of completed PROs must be documented in the source document. The review must be performed by an investigator. The questionnaires take approximately 10 minutes to complete. The below PRO questionnaires will be used.

EuroQoL five dimensions five level (EQ-5D-5L)

The EQ-5D-5L will be used to estimate the impact on participants' health-related quality of life and provides a description of participants' problems by dimensions (descriptive system), a score for overall self-rated health (visual analogue scale (VAS) as well as an index score (EQ-5D-5L index). EQ-5D index score range: 0 to 1 and EQ-5D-VAS: range 0 to 100. A higher score indicates better self-reported health status. If clarification of the test is needed, care should be taken not to bias the participant.

Kansas City Cardiomyopathy Questionnaire (KCCQ)

The KCCQ is a disease-specific health status instrument composed of 23 items that quantify the domains of physical limitation, symptoms, self-efficacy, social limitation, and health-related quality of life limitation from heart failure. The overall summary score and all domains have been independently demonstrated to be valid, reliable, and responsive to clinical change.

Patient Global Impression of Status (PGI-S) and Patient Global Impression of Change (PGI-C)

PGI-S and PGI-C are single-item global rating PRO measures that are used to evaluate the responder threshold. The following PGI-S and PGI-C measures are included:

- Patient Global Impression of Status (PGI-S) for KCCQ version 1.0.
- Patient Global Impression of Change (PGI-C) for KCCQ version 1.0.
- Patient Global Impression of Status (PGI-S) for 6MWT version 1.0.
- Patient Global Impression of Change (PGI-C) for 6MWT version 1.0.

8.2.3 Imaging

The images (cardiac MRI and echocardiography) will be performed at study selected imaging units and images will be analysed by a centralised imaging core laboratory. For standardisation purposes the imaging core laboratory will train the study specific imaging units, as applicable, and the imaging core laboratory will do centralised blinded (blinded to treatment allocation) image analysis/interpretation. The acquisition, display, interpretation, and archiving process of images will be described in an imaging charter and in an imaging manual that will be finalised as supplementary documents before study start. Results from the imaging analyses will be transferred electronically directly from the centralised imaging core laboratory to Novo Nordisk and not shared with site.

8.2.3.1 Cardiac magnetic resonance imaging (MRI)

Cardiac MRI should be performed at the timepoints outlined in the flowchart (Section 1.2) except if a participant has a contraindication to MRI according to local standards. If a participant has a contraindication to MRI, the participant will continue in the study without MRI assessments.

The imaging site should use the same approved MR scanner for all scheduled MR scans. The cardiac MRI scans can be performed ± 2 weeks of the respective visit, except for the first dosing visit where it can be performed up to two weeks in advance (full eligibility needs to be confirmed beforehand). In order to standardise the examination, an MRI site instruction manual will be provided to guide investigators on the image acquisition and the analysis of the planned and optional assessments.

The investigator should ensure that a standard assessment of MR images is performed by a qualified cardiologist/radiologist. In case of any incidental findings, the investigator should be informed and assess if any AEs are to be reported. It is the responsibility of the investigator to refer the participant to further examination and treatment based on the incidental findings as medically indicated. Continued study participation or discontinuation should be considered by the investigator.

To calibrate the MRI, the imaging core laboratory may test the imaging protocol in healthy volunteers. This test will check the MRI settings and the quality of the images generated. Novo Nordisk will not have access to any data generated from the MRI examination on the healthy volunteers and these participants are therefore not considered part of the study.

Measurement of haematocrit for the calculation of ECV should be obtained immediately before the MRI scan, if possible, otherwise within ± 3 days of scanning. If the MRI scan is not done on the same day as a scheduled visit (as outlined in the flowchart in Section 1.2), sites should use an unscheduled laboratory kit for collection of the blood sample for measurement of haematocrit.

8.2.3.2 Echocardiography

A standard echocardiographic examination should be performed at the timepoints outlined in the flowchart (Section 1.2), and the examination can be performed up to 2 days prior to the respective visit. Echocardiographic examination should be performed pre-infusion and the echocardiogram must be assessed locally prior to infusion of trial product. The echocardiography should be interpreted (categorised as normal or abnormal, and, if abnormal, furthermore indicate whether the finding was clinically relevant), signed and dated by the investigator and filed on the participant's medical record. The echocardiography overall evaluation and corresponding outcomes should be documented in source notes and recorded in the eCRF.

Acquisition of echocardiographic images including the analysis should be performed locally by personnel trained in echocardiography. To standardise the examination, an echocardiography site instruction manual will be provided to guide investigators on the image acquisition and the analysis of the planned and optional assessments.

To calibrate the echocardiogram, the imaging core laboratory may test the imaging protocol in healthy volunteers. This test will check the echocardiogram settings and the quality of the images generated. Novo Nordisk will not have access to any data generated from the echocardiographic

examination on the healthy volunteers and these participants are therefore not considered part of the study.

All echocardiography assessments should be performed in accordance with the site instruction manual.

8.3 Safety assessments

Planned time points for all safety assessments are provided in the flowchart (Section 1.2).

Concomitant illness and medical history

A **concomitant illness** is any illness that is already present at the time point from which AEs are collected or found as a result of a screening procedure or other study procedures performed before exposure to study intervention under clinical investigation.

Medical history is a medical event that the participant has experienced in the past. Only relevant medical history should be reported prior to the time point from which AEs are collected.

In case of an abnormal and clinically significant finding fulfilling the definition of medical history or concomitant illness, the investigator must record the finding on the medical history/concomitant illness form.

The following Medical History/Concomitant Illness should be reported in the eCRF:
- History of heart failure
- History of heart rhythm and conduction disturbances
- History of hypotension
- History of peripheral neuropathy
- History of ATTR CM and the exact genetic type if applicable

Any change to a concomitant illness should be recorded during the study. A clinically significant worsening of a concomitant illness should be reported as an AE.

8.3.1 Physical examinations

A physical examination will include assessments of:
- General appearance
- Head, ears, eyes, nose, throat, neck
- Respiratory system
- Cardiovascular system
- Gastrointestinal system incl. mouth
- Musculoskeletal system
- Central and peripheral nervous system
- Skin
- Lymph node palpation
- Endocrine system
- Genitourinary system

Investigators should pay special attention to clinical signs related to previous serious illnesses.

Any abnormal, clinically significant findings prior to start of first dose should be recorded as concomitant illness. Any clinically significant worsening from dosing should be reported as an AE (see Section 8.4).

8.3.2 New York Heart Association (NYHA) classification

The investigator should assess the functional status of the participant based on New York Heart Association (NYHA) classification as specified in the flowchart (Section 1.2). Documentation of the assessment should be recorded in the eCRF.

8.3.3 Body measurements

Body measurements (e.g., height and weight) will also be measured and recorded as specified in the flowchart (Section 1.2).

Body weight

Body weight should be measured with an empty bladder, without shoes and only wearing light clothing on a calibrated scale. Body weight is recorded in kilograms (kg) or pounds (lb).

Height

Height is measured without shoes in centimetres (cm) or inches (in).

8.3.4 Vital signs

Body temperature, pulse rate, as well as systolic and diastolic blood pressure will be assessed and recorded as specified in the flowchart (Section 1.2) and Table 6-2.

Blood pressure and pulse rate

Blood pressure and pulse rate measurements should be preceded by at least 5 minutes of rest for the participant in a quiet setting without distractions (e.g., no use of television, cell phones).

The initial blood pressure and pulse rate measurements at each visit should be assessed sitting. The subsequent measurements at each visit can be assessed sitting or lying. Please see Table 6-2 for timing of measurements.

Blood pressure and pulse rate measurements will be assessed with a completely automated device. Manual techniques must be used only if an automated device is not available.

Blood pressure and pulse rate are collected as specified in the flowchart (Section 1.2).

Blood pressure will consist of 3 systolic and diastolic blood pressure measurements with intervals of at least 1-2 minutes. An additional fourth blood pressure measurement must be performed if the first two readings on systolic or diastolic blood pressure differ by >10 mmHg. No more than four measurements should be performed.

- The last 2 systolic and last 2 diastolic blood pressure measurements should be recorded in the eCRF.

Pulse rate will be measured in connection to the blood pressure measurements.

- The pulse rate for the last 2 measurements should be recorded in the eCRF.

Body temperature

Body temperature should be measured as per local procedure at site with a calibrated thermometer. Body temperature is recorded in degree Celsius (°C) or Fahrenheit (°F) with a precision of one decimal. For details on timepoints for body temperature measurements (Table 6-2). The body temperature measures should be recorded in the eCRF.

8.3.5 Electrocardiograms

12-lead ECG will be obtained as outlined in the flowchart using an ECG machine that automatically calculates the heart rate and measures PR, QRS, QT and QT_c intervals.

The ECG should be interpreted (categorised as normal or abnormal, and, if abnormal, furthermore indicate whether the finding was clinically relevant with a short description, signed and dated by the investigator and filed on the participant's medical record. The ECG measures and corresponding outcomes should be recorded in the eCRF.

Any abnormal clinically relevant findings revealing baseline conditions are to be reported as concomitant illness/medical history in the eCRF. Any clinically significant worsening of a pre-existing condition as well as any new clinically relevant signs, symptoms or disease found as a result of the ECGs conducted after randomisation are to be reported as AEs (please refer to Section 8.4).

Additional ECG recordings can be performed at the investigator's discretion, in which case the reason is to be documented, and an AE reported if applicable.

8.3.6 Cardiac monitoring (only appliable for sentinel participants)

All sentinel participants will be cardiac monitored on both in- and outpatient basis. Cardiac monitoring should be performed at the timepoints outlined in the flowchart (Section 1.2). The cardiac monitoring results should be interpreted (categorised as normal or abnormal, and, if abnormal, furthermore indicate whether the finding was clinically significant), signed and dated by the investigator and filed on the participant's medical record. The cardiac monitoring report over all interpretation and corresponding outcomes should be recorded in the eCRF.
Japan: For country-specific requirements, please refer to Appendix 8 (Section 10.8).

Outpatient 48-hours cardiac monitoring (before randomisation)

During screening, participants will undergo cardiac monitoring (e.g. Holter ECG) on an outpatient basis for at least 48-hours within 2 weeks prior to the randomisation visit. Interpretation and review of the cardiac monitoring report is required prior to randomisation of participants (see Section 5.5).

Inpatient 24-hours cardiac monitoring

When receiving the first dose, participants will be observed under continuous bedside cardiac monitoring for at least 24-hours prior to discharge. Japan: For country-specific requirements, please refer to Appendix 8 (Section 10.8).

Outpatient 6-day cardiac monitoring

On discharge, participants will be monitored using cardiac monitoring (e.g. Holter ECG) on an outpatient basis until 7 days after start of infusion. Japan: For country-specific requirements, please refer to Appendix 8 (Section 10.8).

8.3.7 Clinical safety laboratory assessments

All protocol-required laboratory assessments, as defined in Appendix 2 (Section 10.2), must be conducted in accordance with the laboratory manual and the protocol flowchart.

8.3.8 Pregnancy testing

Women of childbearing potential (WOCBP) should only be included after a negative, highly sensitive pregnancy test (see Appendix 2 [Section 10.2]).

Pregnancy testing should be performed at the timepoints outlined in the flowchart (Section 1.2), as specified in Appendix 2 (Section 10.2).

Pregnancy testing should also be performed whenever a menstruation is missed or when pregnancy is otherwise suspected.

Additional pregnancy testing should be performed during the treatment period, if required locally, refer to Appendix 8 (Section 10.8).

8.4 Adverse events and other safety reporting

The investigator is responsible for detecting, documenting, recording, and following up on events that meet the definition of an AE or SAE.

The definition of AEs and SAEs can be found in Appendix 3 (Section 10.3), along with a description of AEs requiring additional data collection. The definition and description of events for adjudication can be found in Appendix 7 (Section 10.7).

Some AEs require additional data collection on a specific event form. The relevant event(s) are listed below in Table 8-1, together with event(s) for adjudication

Events for adjudication require completion of an adjudication form, please refer to Appendix 7 (Section 10.7).

Table 8-1 AEs requiring additional data collection and events for adjudication

Event type	AE requiring additional data collection	Event for adjudication
Medication error, misuse and abuse	X	
Hypersensitivity reactions	X	
Myocardial inflammation	X	
Cardiac arrhythmia	X	
Death		X
Cardiovascular hospitalisation[a]		X
Urgent heart failure visit not requiring hospitalisation		X

[a]All hospitalisations will be adjudicated

Definitions and reporting timelines for the events mentioned in the above table can be found in Appendix 3 (Section 10.3) and Appendix 7 (Section 10.7) for events requiring adjudication.

8.4.1 Time period and frequency for collecting AE information

All AEs and SAEs must be collected from first administration of trial product under clinical investigation (randomisation visit) and until the follow-up visit in accordance with the flowchart (Section 1.2) or whenever, within the above time period, the site becomes aware of an AE or SAE.

AEs and SAEs are collected from first administration of trial product under clinical investigation (randomisation visit) as no invasive procedures which can give AEs/SAEs are performed during the screening period.

Conditions present prior to the timepoint from which AEs are collected and anticipated day-to-day fluctuations of these conditions, including those identified during screening or during other study-related procedures performed before exposure to study intervention under clinical investigation, will be recorded as medical history/concomitant illness.

AE and SAE reporting timelines can be found in Appendix 3 (Section 10.3). All SAEs must be recorded and reported to Novo Nordisk within 24 hours, and the investigator must submit any updated SAE data to Novo Nordisk within 24 hours of it being available.

Investigators are not obligated to actively seek for AE or SAE in former study participants. However, if the investigator learns of any SAE, including a death, at any time after a participant has discontinued from/completed the study, and the investigator considers the event to be related to the IMP or related to study participation, the investigator must promptly notify Novo Nordisk.

8.4.2 Method of detecting AEs

The method of recording, evaluating, and assessing causality of AE and SAE and the procedures for completing and transmitting SAE reports are provided in Appendix 3 (Section 10.3).

Care should be taken not to introduce bias when detecting AEs and/or SAEs. Open-ended and non-leading verbal questioning of the participant is the preferred method to inquire about events.

8.4.3 Follow-up of AEs

After the initial AE/SAE report, the investigator is required to proactively follow each participant at subsequent visits/contacts. All SAEs should be followed until final outcome of the event or until the participant is lost to follow-up as described in Section 7.3. Further information on follow-up and final outcome of events is given in Appendix 3 (Section 10.3).

8.4.4 Regulatory reporting requirements for SAEs

Prompt notification by the investigator to Novo Nordisk of an SAE is essential so that legal obligations and ethical responsibilities towards the safety of participants and the safety of a study intervention under clinical investigation are met.

Novo Nordisk has a legal responsibility to notify both the local regulatory authority and other regulatory agencies about the safety of a study intervention under clinical investigation. Novo Nordisk will comply with country-specific regulatory requirements relating to safety reporting to the regulatory authority, IRB/IEC, and investigators. This also includes suspected unexpected serious adverse reactions (SUSAR)

An investigator who receives an investigator safety report describing an SAE or other specific safety information (e.g., summary or listing of SAEs) from Novo Nordisk will review and then file it along with the investigator's brochure and will notify the IRB/IEC, if appropriate according to local requirements.

8.4.5 Pregnancy

Details of pregnancies in female participants will be collected after first exposure to IMP and until pregnancy outcome. For details regarding collection and reporting of pregnancy information, please refer to Appendix 4 (Section 10.4).

8.4.6 Cardiovascular and death events

Cardiovascular and death events will be handled and reported according to Section 8.4.

8.4.7 Technical complaints

Technical complaints will be collected for all products listed on the technical complaint form.

Instructions for reporting technical complaints can be found in Appendix 5 (Section 10.5).

In order for Novo Nordisk to perform a complete investigation of reported SAEs, Novo Nordisk might ask the investigator to complete a technical complaint form.

8.5 Pharmacokinetics and pharmacodynamics

8.5.1 Pharmacokinetics

The purpose of measuring plasma NNC6019-0001 levels is to conduct population PK and exposure-response analyses. Single blood samples for measuring plasma concentration of NNC6019-0001 will be drawn on visits specified in the flowchart (Section 1.2). The exact timing (date and time) of obtaining the pharmacokinetic (PK) sample should be recorded on the laboratory requisition form.

Blood samples for PK assessments should be collected, handled, stored, labelled, and shipped according to the description in the laboratory manual supplied by the central laboratory. The bioanalysis of NNC6019-0001 PK will be performed by Novo Nordisk laboratory. NNC6019-0001 PK samples will be stored at the laboratory responsible until final Clinical Study Report (CSR) in case further analysis of the PK samples is required. Details of the bioanalysis will be outlined in a bioanalytical study plan issued by the special laboratory. Bioanalysis of plasma samples for NNC6019-0001 will be carried out using a validated immunoassay.

Residual PK samples should be retained according to Appendix 6 (Section 10.6.4).

8.6 Genetics

Not applicable for this study.

8.7 Biomarkers

Collection of samples for biomarker research is part of this study. The following samples are required and will be collected from all participants in this study:

- blood samples

The detailed sampling regimen is described in the flowchart (Section 1.2) and defined in Appendix 2 (Section 10.2). Biomarkers evaluated in the study include both circulating and imaging biomarkers, sampled at multiple time points throughout the study. The relevant biomarkers directly reflect cardiac status as well as systemic effects related to the target biology.

Some of the biomarkers are also defined as endpoints (Section 3) or assessments (Section 10.2) in this study, and, thus, used for the efficacy and safety evaluation. These include biomarkers related to cardiac status (NT-proBNP and troponin I, GLS, and ECV), inflammation, (hsCRP, IL-6, IL-8, TNF-α, complement C3 and C4), and target biology (misTTR). These pre-defined biomarkers will be analysed prior to database lock (DBL) and reported in the CSR.

Additionally, non-cardiac specific biomarkers related to the target biology, include tetrameric TTR assessments, and levels of retinol binding protein 4 (RBP4) which in patients with hATTR[43] may indirectly reflect degree of stable tetrameric TTR.

Misfolded TTR serves as a potential treatment response biomarker, this has been shown in the FHD study (study NN6019-4965) and in published studies[44], all in participants with hATTR amyloidosis. In relation to the assessment of misfolded TTR, this biomarker holds potential to be both diagnostic and prognostic. In addition, misfolded TTR could be used to monitor treatment response.

Jointly, the abovementioned biomarkers serve as an important part of demonstrating the efficacy, safety, and mode of action of the drug.

The assessment of misfolded TTR will be performed by Novo Nordisk or a special laboratory contracted by Novo Nordisk. The laboratory will provide instructions on sampling, handling of samples, labelling and shipment of samples, which will be detailed in the laboratory manual provided by central lab. Details of the biomarker analyses will be outlined in a biomarker study plan provided by the analysing laboratory.

Residual biomarker samples for misfolded TTR assessment should be retained according to Appendix 6 (Section 10.6.4).

In addition, biosamples are collected for future biomarker analysis. Refer to Section 8.8.3 for further details and Appendix 6 (Section 10.6) for retention.

8.8 Immunogenicity assessments

8.8.1 Anti-NNC6019-0001-antibodies

Anti-drug-antibody samples will be collected according to the flowchart (Section 1.2). All samples must be drawn prior to trial product administration if trial product administration is planned on the sampling day.

Assessment of antibodies against NNC6019-0001 in plasma will be performed by a special laboratory contracted by Novo Nordisk (please refer to Attachment I).

For details on blood sampling, sample preparation and storage, please refer to the laboratory manual.

Analysis for binding anti-NNC6019-0001-antibodies will be performed using a validated anti-drug antibody assay. Confirmed antibody positive samples will be titrated to evaluate the level of the antibody response. Neutralising effect of the antibodies will be evaluated by correlating binding antibody data to PK and PD. Detailed description of the assay methods will be included in an analytical report. Antibody assays will be validated according to international guidelines and recommendations.

Results from the binding anti-drug antibody analysis will be available for the investigator at the end of study upon request.

At the end of the study, the following data will be electronically transferred to the Novo Nordisk database:

- Anti-NNC6019-0001 binding antibodies (positive/negative)
- Anti-NNC6019-0001-antibody titre (numerical)

The investigator will not be able to review the results of antibody measurements in relation to AEs as these are often analysed after LPLV.

For retention of remaining and residual antibody samples, please refer to Appendix 6 (Section 10.6.2).

8.8.2 Assessments in case of suspicion of hypersensitivity to trial product

Participants and investigators will be instructed to detect signs and symptoms of hypersensitivity reactions:

- Local reactions
- Systemic reactions, including anaphylaxis.
- In the event of a hypersensitivity reaction:
- The participant should contact the site for advice on further action as soon as possible.
- Additional data collection will be performed on the event
- Treatment should be provided by the investigator according to local clinical practice.

Additional blood samples and other tests

In the event of an acute severe **systemic** hypersensitivity reaction (i.e., not local reactions), as judged by the investigator, the participant should be called in as soon as possible to have additional blood samples taken in order to analyse the following parameters:

- Tryptase (optimal 0.5 – 2 hours after the hypersensitivity reaction)
- Complement 50
- Anti-NNC6019-0001 binding antibodies
- Anti-NNC6019-0001 IgE antibodies
- Total IgE

Analysis of tryptase and anti-NNC6019-0001 IgE antibodies will be performed by Novo Nordisk, complement 50 and total IgE will be performed by central laboratory, and anti-NNC6019-0001 binding antibodies will be performed by a special laboratory contracted by Novo Nordisk (please refer to Attachment I).

Data from the additional blood samples and tests will be reported in an analytical report and attached to the clinical study report. Furthermore, the results will be included in the narratives of the clinical study report.

For retention of residual hypersensitivity samples, please refer to Appendix 6 (Section 10.6.3).

8.8.3 Human biosamples for future research

Collection of biosamples for future analysis is a component of this study. The samples will be stored in a biobank and allow for future analyses when new knowledge or improved testing technologies may have become available during or after the study. Participation is optional, and participants must sign separate informed consent forms for future research and for genotyping to indicate their participation in the biobank component(s) of the study. Participants cannot take part in the genotyping biobank component only; thus, participants who provide informed consent for genotyping research must also provide informed consent for future research. Participants who do not wish to participate in the biobank component(s) may still participate in the study. Blood and urine samples will be collected according to Appendix 6 (Section 10.6.1) and stored for future use.

Genetic analyses may include analysis of selected genes or genetic markers throughout the genome with the purpose of understanding and predicting response to NNC6019-0001 as well as to understand ATTR CM or other related conditions.

Analyses of circulating biomarkers will measure proteins, lipids, peptides, hormones, metabolites or other non-genetic entities with the purpose of understanding and predicting response to NNC6019-0001 as well as understanding ATTR CM or other related conditions.

The samples may be analysed as part of a multi-study assessment. Results will not be reported to the investigator for assessments of AEs nor will they be part of the clinical study report. The primary objective of the analysis is to investigate on a population level and results are very unlikely to have clinical utility on an individual level. Furthermore, the analyses will be done on pseudonymised data. Therefore, any outcome of the analyses will not be reported directly to participants or sites. The result may be reported in publications, at scientific conferences or to authorities.

The human biosamples for future research will be stored for up to 15 years after end of study at a central laboratory or appropriate storage facility (see Appendix 6 [Section 10.6]).

8.9 Health economics

Not applicable for this study.

9 Statistical considerations

The statistical analysis plan (SAP) will be finalised prior to any interim evaluation, and it will include a more technical and detailed description of the statistical analyses and interim analysis than described in this section.

9.1 Statistical hypotheses

No confirmatory statistical hypothesis testing will be done in this study.

9.1.1 Multiplicity adjustment

As no confirmatory hypothesis will be tested statistically, no adjustment for multiplicity will be done for the two primary endpoints.

9.2 Analysis sets

The following participant analysis sets are defined:

Participant analysis set (PAS)	Description
Full analysis set (FAS)	All randomised participants, except participants who initiate the randomisation registration in RTSM/IWRS but withdraw consent, is withdrawn by the investigator, or is lost to follow-up prior to first dosing administration. Participants will be included in the analyses according to the planned intervention.
Safety analysis set (SAS)	All participants who are exposed to study intervention. Participants will be included in the analyses according to the intervention they actually received.

Abbreviations: FAS = full analysis set; PAS = participant analysis set; SAS = safety analysis set; RTMS/IWRS = Randomisation and Trial Supplies Management System / Interactive Web Response System.

9.3 Statistical analyses

9.3.1 General considerations

Estimated treatment effects will be presented with a 95% confidence interval and a two-sided p-value.

9.3.2 Primary endpoint analysis

The primary endpoints are change in 6MWT and change in NT-proBNP from baseline to week 52.

Analysis addressing the primary estimand

The effect of interest in the primary estimand is regardless of premature discontinuation of study intervention. The primary analysis will be based on the FAS. The following statistical analysis and imputation method is used to address the primary estimand.

Discontinuation of randomised study intervention will be handled by a treatment policy strategy including all post-discontinuation observations. Missing values of 6MWT and NT-proBNP (log-transformed) will be imputed (single or multiple) as described in the estimand section (Table 3-2). Subsequently, values of change from baseline to week 52 will be calculated based on the observed and imputed post-baseline values. The procedure is described in detail below:

First missing values due to death or CV hospitalisation or urgent heart failure visit will be assigned according to Table 3-2.

Thereafter, missing values due to fracture or other reason (Table 3-2) of 6MWT or NT-proBNP (log-transformed) will be multiple imputed sequentially:

- First, intermittent missing post-baseline values are imputed separately for each intervention group using Markov Chain Monte Carlo to generate multiple (1000) copies of the dataset with monotone missing data patterns.
- Next, a stepwise procedure sequentially imputes the missing values for the remaining visits containing missing values.
- At the first visit containing missing values, models are fitted for each copy of the dataset to the observed values for:
    - Pattern 1: Placebo group
    - Pattern 2: NNC6019-0001 10 mg/kg on randomised study intervention
    - Pattern 3: NNC6019-0001 60 mg/kg on randomised study intervention

  The models will include the stratification variable as a factor and as covariates baseline 6MWT or NT-proBNP (log-transformed) and the observed post-baseline assessments for visits prior to the one in question. The estimated parameters, and their variances, are used to impute missing post-baseline values for the visit in question. For 6MWT, a minimum of zero will be specified to prevent imputations below zero.
    - Placebo group: Impute from pattern 1
    - NNC6019-0001 10 mg/kg group and prematurely discontinued randomised study intervention: Impute from pattern 1
    - NNC6019-0001 60 mg/kg group and prematurely discontinued randomised study intervention: Impute from pattern 1
    - NNC6019-0001 10 mg/kg on randomised study intervention: Impute from pattern 2
    - NNC6019-0001 60 mg/kg on randomised study intervention: Impute from pattern 3

  The stepwise procedure is repeated sequentially for Visits 3, Visit 5, Visit 8, and Visit 15 to impute the missing values. If no intermittent missing values exist, multiple copies of the dataset will be generated at the first visit where missing values are present.
  Values of change from baseline to Visit 15 (week 52) will be calculated based on the observed and imputed post-baseline values.
- For each of the complete data sets, change in 6MWT or NT-proBNP (log-transformed) from baseline to Visit 15 (week 52) is analysed using an analysis of variance model with randomised study intervention (NNC6019-0001 (10 mg/kg or 60 mg/kg) vs placebo) and the stratification variable as factors and baseline 6MWT or NT-proBNP (log-transformed) as a covariate.
- The estimates and standard deviations for the dataset copies are pooled to one estimate and associated standard deviation using Rubin's rule. For NT-proBNP, the mean difference on the logarithmic scale will be back-transformed to original scale and reported as a ratio of geometric mean ratios.

9.3.3 Secondary endpoints analysis

9.3.3.1 Supportive secondary endpoints

For details on analyses of additional supportive secondary endpoints, please refer to the SAP.

9.3.4 Exploratory endpoints analysis

For details on analyses of exploratory endpoints, please refer to the SAP.

9.3.5 Other safety analyses

All safety analyses will be made on the safety analysis set. The standard safety assessments (AEs, safety laboratory parameters, vital signs, etc.) will be reported descriptively, including any notable changes of clinical interest in laboratory parameters.

9.3.6 Other analyses

Potential analyses on additional outcomes from the cardiac MRI scans and echocardiographic parameters will be described in the SAP. For other analyses, please also refer to the SAP.

9.3.6.1 Pharmacokinetic and pharmacodynamic modelling

Population PK and exposure-response analysis based on drug concentration and response data from the study will be performed.

The objective of the population PK analysis is to evaluate the effects of pre-specified covariates on drug exposure. The objective of the exposure-response analysis is to investigate the relationship between drug exposure and response and to evaluate the effects of pre-specified covariates on this relationship. A more technical and detailed elaboration of the population PK analysis and exposure-response analysis will be given in a modelling analysis plan (MAP), which will be prepared before DBL.

The population PK and exposure-response analysis will be reported in a separate modelling report, which will not be part of the clinical study report. The individual drug concentration data will be tabulated in the bioanalytical report.

9.4 Interim analysis

An interim evaluation and a partial DBL are pre-planned but may be reconsidered during the study period. It is not considered a protocol deviation if one or more interim evaluation is not performed. An interim evaluation is planned when all participants still in the study have reached the week 24 visit based on all efficacy, safety, and PK data supporting a preliminary selection of the phase 3 dose. The purpose is to obtain advice from regulatory agencies on the overall phase 3 design and the preliminary selected dose to be investigated. The evaluation will primarily be based on the endpoints 6MWT, NT-proBNP, ECV and KCCQ, while simultaneously considering PK data, exposure-response relationships, and the overall safety profile. No change in study design can occur as a consequence of the interim evaluation and the study will not be stopped for either positive efficacy or futility.

A minimal number of Novo Nordisk personnel in an Unblinded Interim Team will be unblinded to perform the interim analyses and interpret the results. From unblinding at the interim evaluation until database lock for the partial DBL, the Unblinded Interim Team cannot be involved in the daily study activities, including but not limited to data cleaning, medical monitoring, safety surveillance, or involved in protocol amendments, updates to existing endpoints, or definition of new endpoints and analyses. Thereafter, the Unblinded Interim Team can again be involved in the study

evaluation. To avoid inducing bias to data collected after the interim evaluation and maintain the integrity of the study, results will only be shared with regulatory authorities and kept confidential to participants, investigators and Novo Nordisk personnel who is not member of the Unblinded Interim Team.

A partial DBL is planned when all participants have completed the end of treatment visit (prior to completion of the follow-up visit for all participants) based on the available efficacy, safety and PK data to confirm the phase 3 dose selected at the interim evaluation. If the evaluation of the selected dose changes from the interim evaluation (if conducted), another dose may be selected. The evaluation will primarily be based on the statistical comparisons of 6MWT, NT-proBNP, ECV and KCCQ specified in the protocol and the SAP, while simultaneously considering PK data, exposure-response relationships, and the overall safety profile. No change in study design can occur as a consequence of this partial DBL evaluation. The regular study team will perform the evaluation, as only follow-up data collection is ongoing.

Further information will be specified in an interim charter or SAP before unblinding.

9.5 Sample size determination

As no confirmatory hypothesis will be tested statistically, the sample size calculation is based on the precision of the comparisons of the primary endpoints.

In the ATTR-ACT[22] study (Figure 4A), a change from baseline of approximately -23 meters was observed for the pooled tafamidis group and -55 meters for the placebo group. With up to 30% of participants expected to be on tafamidis in the present study, a change from baseline in the placebo group of -23*0.3 + -55*0.7 = -45 meters can be expected. Therefore, a 95% confidence interval for the difference between NNC6019-0001 and placebo with a half-width of 45 meters is considered appropriate. With 27 participants in each intervention group and an SD of 75 there is more than 85% probability for obtaining such a confidence interval. The probability of obtaining a half-width of 45 meters on the confidence interval for 6MWT for various sample sizes and standard deviations is shown in Table 9-1.

Based on relative change in NT-proBNP compared to placebo after 52 weeks from the studies ATTR-ACT[22] (approximate 18% reduction), and APOLLO[45] (approximate 44% reduction), a 95% confidence interval with a half-width of 30% reduction between NNC6019-0001 and placebo is considered appropriate. Assuming a coefficient of variation of approximately 0.65 observed in APOLLO[45] it requires 27 participants in each intervention group to obtain such a confidence interval with 80% probability.

Allowing for approximately 20% dropout, it is planned to randomise 99 participants 1:1:1 with 33 participants in each intervention group.

Table 9-1 Probability of obtaining a half-width of 45 meters on the confidence interval for 6MWT for various sample sizes and standard deviations (SD)

	**Participants per intervention group allowing 20% dropout**		
SD	30	33	36
70	85%	96%	100%
75	64%	85%	96%

Abbreviations: SD = standard deviation.

10 Supporting documentation and operational considerations

10.1 Appendix 1: Regulatory, ethical, and study oversight considerations

10.1.1 Regulatory and ethical considerations

This study will be conducted in accordance with the protocol and with the following:

- Consensus ethical principles derived from international guidelines including the Declaration of Helsinki[46] and applicable ICH Good Clinical Practice (GCP) Guideline[47]
- Applicable laws and regulations

The protocol, informed consent form, investigator's brochure (as applicable) and other relevant documents (e.g., advertisements) must be submitted to an IRB/IEC and reviewed and approved by the IRB/IEC before the study is initiated.

Regulatory authorities will receive the clinical trial application, protocol amendments, reports on SAEs, and the CSR according to national requirements.

Any amendments to the protocol will require IRB/IEC approval before implementation of changes made to the study design, except for changes necessary to eliminate an immediate safety hazard to study participants.

Before a site is allowed to start screening participants, written notification from Novo Nordisk must be received.

The investigator will be responsible for:

- providing written summaries of the status of the study annually or more frequently in accordance with the requirements, policies, and procedures established by the IRB/IEC and/or regulatory authorities
- notifying the IRB/IEC of SAEs or other significant safety findings as required by IRB/IEC procedures
- providing oversight of the conduct of the study at the site and adherence to requirements of ICH guidelines, the IRB/IEC, and all other applicable local regulations
- ensuring submission of the CSR synopsis to the IRB/IEC
- reporting any potential serious breaches to the sponsor immediately after discovery

US: For country-specific requirements, please refer to Appendix 8 (Section 10.8).

10.1.2 Financial disclosure

Investigators and sub-investigators will provide Novo Nordisk with sufficient, accurate financial information as requested to allow Novo Nordisk to submit complete and accurate financial certification or disclosure statements to the appropriate regulatory authorities. Investigators are responsible for providing information on financial interests during the course of the study and one year after completion of the study.
Verification under disclosures per Code of Federal Regulations (CFR) of Financial Conflict of Interest.

10.1.3 Informed consent process

The investigator or his/her representative will explain the nature of the study, including the risks and benefits, to the participant and answer all questions regarding the study. This includes the use of an impartial witness where required according to local requirements.

The investigator must ensure the participant ample time to come to a decision whether or not to participate in the study.

Participants must be informed that their participation is voluntary. Participants will be required to sign and date a statement of informed consent that meets the requirements of local regulations, ICH GCP[47] guidelines, Declaration of Helsinki,[46] privacy and data protection requirements, where applicable, and the IRB/IEC or site.

The medical record must include a statement that written informed consent was obtained before any study-related activity and the date when the written consent was obtained. The authorised person obtaining the informed consent must also sign and date the informed consent form before any study-related activity.

The responsibility of seeking informed consent must remain with the investigator, but the investigator may delegate the task to a medically qualified person, in accordance with local requirements.

Participants must be re-consented to the most current version of the informed consent form(s) during their participation in the study.

A copy of the informed consent form(s) must be provided to the participant.

Czech Republic: For country-specific requirements, please refer to Appendix 8 (Section 10.8).

10.1.4 Information to participants during the study

The site will be offered a communication package for the participant during the conduct of the study. The package content is issued by Novo Nordisk. The communication package will contain written information intended for distribution to the participants. The written information will be translated and adjusted to local requirements and distributed to the participant at the discretion of the investigator. The participant may receive a “thank you for your participation letter” after completion of the study. Further, the participant may receive other written information during the study.

All written information to participants must be sent to IRB/IEC for approval/favourable opinion and to regulatory authorities for approval or notification according to local regulations.

10.1.5 Data protection

Participants will be assigned a 6-digit unique identifier, a subject ID. Any participant records or datasets that are transferred to Novo Nordisk will contain the identifier only. No direct identifiers from the participant are transferred to Novo Nordisk.

The participant and any biological material obtained from the participant will be identified by subject ID, visit number and study ID. Appropriate measures such as encryption or leaving out certain identifiers will be enforced to protect the identity of participants as required by local, regional and national requirements.

The participant must be informed about his/her privacy rights, including that his/her personal study-related data will be used by Novo Nordisk in accordance with local data protection law. The disclosure of the data must also be explained to the participant.

The participant must be informed that his/her medical records may be examined by auditors or other authorised personnel appointed by Novo Nordisk, by appropriate IRB/IEC members, and by inspectors from regulatory authorities.

Personal data may be collected from participants due to process requirements from Novo Nordisk's suppliers. This data is needed to ensure that the relevant data analysis for the study can be performed, but will not be part of the data transferred to Novo Nordisk, the assessment of the study endpoints or the clinical study report. A list of any such data values must be kept as part of the study documentation along with an explanation of why it was required.

Spain: For country-specific requirements, please refer to Appendix 8 (Section 10.8).

10.1.6 Committee structure

10.1.6.1 Novo Nordisk safety committee

Novo Nordisk will perform ongoing safety surveillance. If new safety signals are identified, these will be evaluated by an internal safety committee. The safety committee may recommend unblinding of any data for further analysis, and in this case an internal study-independent ad hoc group may be established in order to maintain the blinding of the study personnel.

10.1.6.2 Data monitoring committee

The DMC is an independent, external committee composed of members whose expertise covers relevant specialties including statistics. The DMC is established to review and evaluate accumulated data from the study at predefined time points as well as *ad hoc*. This is done in order to protect the safety of the participants and to evaluate the benefit-risk balance. The DMC will have access to unblinded data, and will provide recommendations on study continuation, modification or termination.

Information regarding responsibilities, procedures and workflow to be used by the DMC are specified in the DMC charter.

10.1.6.3 Steering Committee

A steering committee will provide scientific and operational leadership for the study. The committee will consist of experts from outside Novo Nordisk, and designated Novo Nordisk employees. The committee will operate under a charter agreed with Novo Nordisk.

10.1.6.4 Event adjudication committee

An independent external EAC is established to perform ongoing blinded adjudication of selected AEs and deaths (see Table 8-1 and Appendix 7 [Section 10.7]).

The EAC will evaluate events sent for adjudication using pre-defined definitions and guidelines in accordance with the EAC charter. The evaluation is based on review of pre-defined clinical data collected by the sites. The EAC is composed of permanent members covering all required medical specialities. EAC members must disclose any potential conflicts of interest and must be independent of Novo Nordisk. The EAC will have no authority to impact study conduct, study protocol or amendments. The assessments made by both the event adjudication committee and the investigator will be evaluated and included in the CSR.

10.1.7 Dissemination of clinical study data

Study information will be disclosed at clinicaltrials.gov and novonordisk-trials.com and, if applicable, also on other national or regional study registries. It will be disclosed according to applicable requirements, relevant recommendations or regulations, such as the Declaration of Helsinki,[46] the International Committee of Medical Journal Editors (ICMJE),[48] the Food and Drug Administration Amendment Act (FDAAA),[49] European Commission Requirements[2, 50, 51] and in accordance with Novo Nordisk commitment to clinical transparency. If a participant requests to be included in the study via the Novo Nordisk e-mail contact at these web sites, Novo Nordisk may disclose the investigator's contact details to the participant. As a result of increasing requirements for transparency, some countries require public disclosure of investigator names and their affiliations.

Japan: For country-specific requirements, please refer to Appendix 8 (Section 10.8).

10.1.8 Data quality assurance

10.1.8.1 Case report forms

Novo Nordisk or designee is responsible for the data management of this study including quality checking of the data.

To demonstrate his/her oversight of the collected data, the investigator should sign the eCRF on a regular basis during the conduct of the study as well as at the end of the study, as described in the eCRF completion guideline.

All participant data relating to the study will be recorded on eCRFs unless transmitted electronically to Novo Nordisk or designee (e.g., laboratory data). The investigator is responsible for verifying that data entries are accurate and correct by physically or electronically signing the CRF.

The following will be provided as paper CRFs:
- Pregnancy forms
- Technical complaint forms

The following will be provided as paper CRFs to be used when access to the CRF is revoked or the CRF is temporarily unavailable:

- AE forms
- Safety information forms

Corrections to the CRF data may be made by the investigator or the investigator's delegated staff. An audit trail will be maintained in the CRF application containing as a minimum: the old and the new data, identification of the person entering the data, date and time of the entry and reason for the correction. If corrections are made by the investigator's delegated staff after the date when the investigator signed the CRF, the CRF must be signed and dated again by the investigator.

The investigator must ensure that data is recorded in the CRF as soon as possible, preferably within 5 working days after the visit. Once data has been entered, it will be available to Novo Nordisk for data verification and validation purposes.

10.1.8.2 Monitoring

The investigator must permit study-related monitoring, audits, IRB/IEC review, and regulatory agency inspections and provide direct access to source data documents (original documents, data and records). Direct access includes permission to examine, analyse, verify and reproduce any record(s) and report(s) that are important to the evaluation of the study. If the electronic source data does not have a visible audit trail, the investigator must provide the monitor with signed and dated printouts. In addition, the relevant site staff should be available for discussions at monitoring visits and between monitoring visits (e.g., by telephone).

Study monitors will perform ongoing source data verification of critical data points to confirm that data entered into the eCRF by authorised site personnel are accurate, complete and verifiable from source documents. Study monitors will perform ongoing source data review to ensure that the study is being conducted in accordance with the current approved protocol and any other study agreements, ICH GCP[47] and all applicable regulatory requirements, evaluating the adequacy of critical processes at site for the execution of the protocol, collection of study data, to ensure that the safety and rights of participants are being protected.

Monitoring will be conducted using a risk-based approach including risk assessment, monitoring plans, centralised monitoring (remote assessment of data by Novo Nordisk) and visits to sites.

Quality tolerance limits (QTLs) will be predefined in the relevant monitoring plan to identify systematic issues that can impact participant safety and/or reliability of study results. These predefined parameters will be monitored during the study, and important deviations from the QTLs and remedial actions taken will be summarised in the clinical study report.

An unblinded monitor will visit the study site to ensure that drug handling procedures are adhered to (e.g., that the unblinded pharmacy binder and drug accountability has been completed correctly) and will reconcile trial product accountability.

10.1.8.3 Protocol compliance

Deviations from the protocol should be avoided. If deviations do occur, the investigator must inform the monitor without delay and the implications of the deviation must be reviewed and discussed.

Deviations must be documented and explained in a protocol deviation by stating the reason, date, and the action(s) taken. Some deviations, for which corrections are not possible, can be acknowledged and confirmed via edit checks in the eCRF or via listings from the study database.

10.1.9 Source documents

All data entered in the eCRF must be verifiable in source documentation other than the eCRF.

If source data is entered directly in a paper CRF, each data entry or clear series of data entries must be signed and dated separately by the study staff making the entry.

The original of the completed PROs must not be removed from the site.

Source documents provide evidence for the existence of the participant and substantiate the integrity of the data collected. Source documents are filed at the site. Any source data generated by investigator's subcontractors must be archived and accessible by the site.

Data that is transcribed into the eCRF from source documents must be consistent with the source documents, or the discrepancies must be explained. The investigator may need to request previous medical records or transfer records. Also, current medical records must be available.

It must be possible to verify participant's medical history in source documents, such as participant's medical record.

The investigator must document any attempt to obtain external medical information by noting the date(s) when information was requested, and who was contacted.

Definition of what constitutes source data can be found in a source document agreement at each site. There will only be one source document defined at any time for any data element.

10.1.10 Retention of clinical study documentation

Records and documents, including signed informed consent forms, pertaining to the conduct of this study must be retained by the investigator for 25 years after end of study unless local regulations or institutional policies require a longer retention period. No records may be destroyed during the retention period without the written approval of Novo Nordisk. No records may be transferred to another location or party without written notification to Novo Nordisk.

The investigator must be able to access his/her study documents without involving Novo Nordisk in any way. If applicable, electronic CRF (eCRF) and other participant data will be provided in an electronic readable format to the investigator before access is revoked to the systems supplied by Novo Nordisk. Site-specific CRFs and other participant data (in an electronic readable format or as paper copies or prints) must be retained by the site. A copy of all data will be stored by Novo Nordisk.

Participant's medical records must be kept for the maximum period permitted by the hospital, institution or private practice.

US: For country-specific requirements, please refer to Appendix 8 (Section 10.8).

10.1.11 Study and site closure

Novo Nordisk reserves the right to close the site or terminate the study at any time for any reason at the sole discretion of Novo Nordisk. If the study is suspended or terminated, the investigator must inform the participants promptly and ensure appropriate therapy and follow-up. The investigator and/or Novo Nordisk must also promptly inform the regulatory authorities and IRBs/IECs and provide a detailed written explanation.

Sites will be closed upon study completion. A site is considered closed when all required documents and study supplies have been collected and a site closure visit has been performed.

The investigator may initiate site closure at any time, provided there is reasonable cause and sufficient notice is given in advance of the intended termination.

Reasons for the early closure of a site by Novo Nordisk or investigator may include but are not limited to:

- failure of the investigator to comply with the protocol, the requirements of the IRB/IEC or local health authorities, Novo Nordisk procedures or GCP guidelines
- inadequate recruitment of participants by the investigator
- discontinuation of further study intervention development.

10.1.12 Responsibilities

The investigator is accountable for the conduct of the study at his/her site and must ensure adequate supervision of the conduct of the study at the site. If any tasks are delegated, the investigator must maintain a log of appropriately qualified persons to whom he/she has delegated specified study-related duties. The investigator must ensure that there is adequate and documented training for all staff participating in the conduct of the study. It is the investigator's responsibility to supervise the conduct of the study and to protect the rights, safety, and well-being of the participants.

A qualified physician, who is an investigator or a sub investigator for the study, must be responsible for all study-related medical decisions.

The investigator is responsible for filing essential documents (i.e., those documents which individually and collectively permit evaluation of the conduct of a study and the quality of the data produced) in the investigator trial master file. The documents, including the participant identification code list must be kept in a secure locked facility so that no unauthorised persons can get access to the data.

The investigator will take all necessary technical and organisational safety measures to prevent accidental or wrongful destruction, loss or deterioration of data. The investigator will prevent any unauthorised access to data or any other processing of data against applicable law. This also includes ensuring that no indirect sharing of user credentials for IT systems used in this study takes place (e.g., by not sharing IT equipment with others in a way where user credentials have the

possibility of being shared). The investigator must be able to provide the necessary information or otherwise demonstrate to Novo Nordisk that such technical and organisational safety measures have been taken.

During any period of unavailability, the investigator must delegate responsibility for medical care of participants to a specific qualified physician who will be readily available to participants during that time.

If the investigator is no longer able to fulfil the role as investigator (e.g., if he/she moves or retires), a new investigator will be appointed in consultation with Novo Nordisk.

The investigator and other site personnel must have sufficient English skills according to their assigned task(s).

10.1.13 Indemnity statement

Novo Nordisk carries product liability for its products, and liability as assumed under the special laws, acts and/or guidelines for conducting clinical studies in any country, unless others have shown negligence.

Novo Nordisk assumes no liability in the event of negligence or any other liability of the sites or investigators conducting the study or by persons for whom the said site or investigator are responsible.

Novo Nordisk accepts liability in accordance with country-specific laws, acts and guidelines. France, Spain: For any country specific indemnity requirements supplementing the above, please refer to Appendix 8 (Section 10.8).

10.1.14 Publication policy

The information obtained during the conduct of this study is considered confidential and may be used by or on behalf of Novo Nordisk for regulatory purposes as well as for the general development of the study intervention. All information supplied by Novo Nordisk in connection with this study shall remain the sole property of Novo Nordisk and is to be considered confidential information.

No confidential information shall be disclosed to others without prior written consent from Novo Nordisk. Such information shall not be used except in the performance of this study.

The information obtained during this study may be made available to other investigators who are conducting other clinical studies with the study intervention, if deemed necessary by Novo Nordisk. Provided that certain conditions are fulfilled, Novo Nordisk may grant access to information obtained during this study to researchers who require access for research projects studying the same or related diseases and/or study intervention studied in this study.

Novo Nordisk may publish on its clinical studies website a redacted CSR for this study.

One investigator will be appointed by Novo Nordisk to review and sign the CSR (signatory investigator) on behalf of all participating investigators.

10.1.14.1 Communication of results

Novo Nordisk commits to communicate and disclose results of studies regardless of outcome. Disclosure includes publication of a manuscript in a peer-reviewed scientific journal, abstract submission with a poster or oral presentation at a scientific meeting or disclosure by other means.

The results of this study will be subject to public disclosure on external web sites according to international and national regulations. Novo Nordisk reserves the right to defer the release of data until specified milestones are reached, for example when the CSR is available. This includes the right not to release the results of interim analyses, because the release of such information may influence the results of the entire study.

At the end of the study, one or more scientific publications may be prepared collaboratively by the investigator(s) and Novo Nordisk. Novo Nordisk reserves the right to postpone publication and/or communication for up to 60 days to protect intellectual property.

In all cases, the study results will be reported in an objective, accurate, balanced and complete manner, with a discussion of the strengths and limitations. In the event of any disagreement on the content of any publication, both the investigators' and Novo Nordisk opinions will be fairly and sufficiently represented in the publication.

10.1.14.2 Authorship

Novo Nordisk will work with one or more investigator(s) and other experts who have contributed to the study concept or design, acquisition, analysis or interpretation of data to report the results in one or more publications.

Authorship of publications should be in accordance with the Recommendations for the Conduct, Reporting, Editing and Publication of Scholarly Work in Medical Journals by the International Committee of Medical Journal Editors.[52]

All authors will be provided with the relevant statistical tables, figures, and reports needed to evaluate the planned publication.

Where required by the journal, the investigator from each site will be named in an acknowledgement or in the supplementary material, as specified by the journal.

10.1.14.3 Site-specific publication(s) by investigator(s)

For a multicentre clinical study, analyses based on single-site data usually have significant statistical limitations and frequently do not provide meaningful information for healthcare professionals or participants, and therefore may not be supported by Novo Nordisk. Thus, Novo Nordisk may deny a request or ask for deferment of the publication of individual site results until the primary manuscript is accepted for publication. In line with Good Publication Practice, such individual reports should not precede the primary manuscript and should always reference the primary manuscript of the study.

10.1.14.4 Investigator access to data and review of results

As owner of the study database, Novo Nordisk has the discretion to determine who will have access to the database. Individual investigators will have their own research participants' data and will be provided with the randomisation code after results are available.

10.2 Appendix 2: Clinical laboratory tests

The tests detailed in Table 10-1 and Table 10-2 will be performed by the central laboratory unless otherwise noted.

Additional tests may be performed at any time during the study as determined necessary by the investigator or required by local regulations. Only laboratory samples specified in the protocol should be sent to the central laboratory for analysis; if additional laboratory sampling is needed, e.g., to follow up on AEs, this must be done at a local laboratory.

The central lab will communicate to the investigator abnormal values of parameters not requested in the protocol but identified by the laboratory equipment and/or their processes according to their laboratory standard operating procedures (SOPs). These data will not be transferred to the study database. The investigator should review such values for AEs and report these according to this protocol.

The investigator must review all laboratory results for concomitant illnesses and AEs.

The investigator must keep an overview, e.g., a log, of laboratory samples not handled according to the laboratory manual. In addition, the investigator must keep an overview, e.g., a log, of laboratory samples stored at site.

Human biosamples for future research will be stored as described in Appendix 6 (Section 10.6.1).

US: For country-specific requirements, please refer to Appendix 8 (Section 10.8).

Table 10-1 Protocol-required efficacy laboratory assessments

Laboratory assessments	Parameters
Cardiac biomarkers	• N-terminal pro-brain natriuretic peptide (NT-proBNP)<br>• Troponin I
Inflammatory biomarkers	• High Sensitive C-Reactive Protein (CRP)<br>• IL-6<br>• IL-8<br>• TNF-α<br>• Complement C3<br>• Complement C4
Pharmacokinetics[a]	• NNC6019-0001 plasma concentrations
Other tests	• Misfolded transthyretin[a, b] (misTTR)<br>• Retinol binding protein 4[a] (RBP4)

[a] Results from PK, RBP4 and misTTR will not be made available to investigators during study conduct; [b] misTTR or fragments thereof depending on assay availability and the analysis of misTTR will be performed by Novo Nordisk or a special laboratory contracted by Novo Nordisk.

Table 10-2 Protocol-required safety laboratory assessments

Laboratory assessments	Parameters
Haematology	• Lymphocytes<br>• Eosinophils<br>• Basophils<br>• Monocytes<br>• Neutrophils<br>• Haematocrit<br>• Haemoglobin<br>• Erythrocytes<br>• Leukocytes<br>• Thrombocytes
Biochemistry[a]	• Alanine Aminotransferase (ALT)<br>• Alkaline phosphatase<br>• Aspartate Aminotransferase (AST)<br>• Bilirubin<br>• Creatinine<br>• Potassium<br>• Sodium<br>• Urea<br>• Gamma-Glutamyl Transferase (GGT)<br>• Creatinin Kinase<br>• Glucose<br>• Albumin<br>• Calcium
Coagulation Parameters	• INR<br>• Prothrombin Time<br>• Partial Thromboplastin Time
Lipids	• Cholesterol<br>• High density lipoprotein (HDL) cholesterol<br>• Low density lipoprotein (LDL) cholesterol<br>• Triglycerides
Hormones	• Thyrotropin (TSH)
Serology	• HIV antibody[c]<br>• HIV antigen[c]<br>• Hepatitis B core antibody (HBcAb)<br>• Hepatitis B surface antigen (HBsAg)<br>• Hepatitis C virus antibody[d]
Pregnancy Testing[b]	• Highly sensitive urine human chorionic gonadotropin (hCG) pregnancy test
Urinalysis	• Urine-Albumin to Creatinine Ratio
Other tests	• eGFR calculated by the central laboratory based on the creatinine value using the CKD-EPI equation<br>• Transthyretin (TTR)[e]<br>• In case of systemic hypersensitivity (Section 8.8.2): anti-NNC6019-0001-antibodies[e], anti-NNC6019-0001 IgE[e], total IgE[e], tryptase[e] and complement 50
Antibodies[e]	• Anti-NNC6019-0001-antibodies<br>• Anti-NNC6019-0001-antibody titre
Biosamples for future research	• Whole blood for genetic analysis<br>• Serum and plasma (for analyses of circulating biomarkers)

Laboratory assessments	Parameters
	• Urine spot samples (for analyses of filtered circulating biomarkers)

[a] Details of required actions and follow-up assessments for increased liver parameters including any discontinuation criteria are given in Appendix 3 (Section 10.3) (Hy's Law) and Section 7.1; [b] For women of childbearing potential, as needed, local urine testing will be standard unless serum testing is required by local regulation or IRB/IEC, see Appendix 4 (Section 10.4); [c] Additional HIV serology may be performed if needed; [d] As per current medical standards i.e. hepatitis C virus RNA testing needed only if the anti-hepatitis C virus screening is positive; [e] Results from anti-drug antibodies, tryptase and TTR, will not be made available to investigators during study conduct.

10.3 Appendix 3: Adverse Events and Serious Adverse Events: Definitions and procedures for recording, evaluating, follow-up, and reporting

10.3.1 Definition of AE

An AE is any untoward medical occurrence in a clinical study participant that is temporally associated with the use of IMP, whether or not considered related to the IMP. An AE can therefore be any unfavourable and unintended sign (including an abnormal laboratory finding), symptom or disease (new or exacerbated) temporally associated with the use of an IMP.

Events to be reported as AEs:

- Any abnormal laboratory test results or safety assessments considered clinically significant in the medical and scientific judgment of the investigator, including events that have worsened from prior to the time point from which AEs are collected
- Conditions detected or diagnosed after IMP administration even though it may have been present prior to the time point from which AEs are collected
- Exacerbation/worsening of a chronic or intermittent condition including either an increase in frequency and/or intensity of the condition
- Signs, symptoms or the clinical sequelae of a suspected drug-drug interaction
- Signs, symptoms or the clinical sequelae of a suspected overdose of IMP regardless of intent

A 'lack of efficacy' or 'failure of expected pharmacological action' per se will not be reported as an AE or SAE. Such instances will be captured in the efficacy assessments. However, the signs, symptoms and/or clinical sequelae resulting from lack of efficacy will be reported as AE or SAE if they fulfil the definition.

Events NOT to be reported as AEs:

- Conditions present prior to the time point from which AEs are collected and anticipated day-to-day fluctuations of these conditions. This includes those conditions identified during screening or identified during other study procedures performed before exposure to IMP.
  Note: Conditions present or occurring prior to the time point from which AEs are collected should be recorded as concomitant illness/medical history.
- Medical or surgical procedures (e.g., endoscopy, appendectomy). The condition that leads to the procedure is the AE.
- Medical or surgical procedures not preceded by an AE or worsening of a known condition.

10.3.2 Definition of an SAE

An SAE is any untoward medical occurrence that fulfils at least one of the following criteria:

- **Results in death**
- **Is life-threatening**
  - The term 'life-threatening' refers to an event in which the participant was at risk of death at the time of the event. It does not refer to an event which hypothetically might have caused death, if it were more severe.
- **Requires inpatient hospitalisation or prolongation of existing hospitalisation**
  - Hospitalisation signifies that the participant has been admitted at the hospital or emergency ward for observation and/or treatment that would not have been appropriate in the

physician's office or outpatient setting. Complications that occur during hospitalisation are AEs. If a complication prolongs hospitalisation or fulfils any other seriousness criteria, the event is serious. When in doubt as to whether 'hospitalisation' occurred or was necessary, the AE should be considered serious.

- Hospitalisation for elective treatment (e.g., elective medical or surgical procedures) of a condition that was present prior to the time point from which AEs are collected, and that did not worsen, is not considered an AE.

  Note: Hospitalisations for administrative, study-related, social and convenience reasons do not constitute AEs and should therefore not be reported as AEs or SAEs. Hospital admissions for medical or surgical procedures, planned before study inclusion, are not considered AEs or SAEs

- **Results in persistent or significant disability/incapacity**
  - The term 'disability' means a substantial disruption of a person's ability to conduct normal life functions. This definition is not intended to include experience of relatively minor medical significance, such as uncomplicated headache, nausea, vomiting, diarrhoea, influenza, and accidental trauma (e.g., sprained ankle), that may interfere with or prevent everyday life functions but do not constitute a substantial disruption.
- **Is a congenital anomaly/birth defect**
- **Important medical event:**
  - Medical or scientific judgment should be exercised by the investigator in deciding whether SAE reporting is appropriate in other situations. This includes important medical events that may not be immediately life-threatening or result in death or hospitalisation but may jeopardise the participant or may require medical or surgical intervention to prevent one of the other outcomes listed in the above definition. These events should usually be considered serious and reported as SAEs using the important medical event criterion.
  - The following must be reported as an SAE using the important medical event criterion if no other seriousness criteria are applicable:
    - Suspicion of transmission of infectious agents via IMP
    - Risk of liver injury defined as alanine aminotransferase (ALT) or asparate aminotransferase (AST) >3x UNL and total bilirubin >2x UNL where no alternative aetiology exists (Hy's law)

10.3.3 Description of AEs requiring additional data collection

Adverse events requiring additional data collection (on specific event form)

An AE requiring additional data collection is an AE where Novo Nordisk has evaluated that additional data is needed in the evaluation of safety.

Hypersensitivity reactions
Hypersensitivity is defined as episodes of objectively reproducible symptoms or signs initiated by exposure to a defined stimulus at a dose tolerated by normal persons. Hypersensitivity includes:

- local reactions
- Systemic reactions, including anaphylaxis.

Anaphylaxis is defined as serious hypersensitivity reactions that is rapid in onset and may cause death.

Myocardial inflammation

Newly onset of non-infectious myocarditis.

Cardiac arrhythmia

Cardiac rhythm disturbances, including atrial and/or ventricular tachy- and/or bradyarrhythmia will be collected to assess the risk of cardiac arrhythmia, including:

1. A change or worsening of current arrhythmia
2. The development of a new arrhythmia
3. The development of new/a change or worsening of the sinoatrial and atrioventricular conduction disorders.

Medication error

- A medication error is an unintended failure in the IMP treatment process that leads to, or has the potential to lead to, harm to the participant, such as:
  - administration of wrong drug.
    Note: Use of wrong DUN is not considered a medication error unless it results in administration of wrong drug.
  - wrong route of administration, such as intramuscular instead of subcutaneous
  - accidental administration of a lower or higher dose than intended (including faster or slower infusion time). The administered dose must deviate from the intended dose to an extent where clinical consequences for the study participant were likely to happen as judged by the investigator, although they did not necessarily occur.

Misuse and abuse

- Situations where the IMP is intentionally and inappropriately used not in accordance with the protocol (e.g., overdose to maximise effect)
- Persistent or sporadic, intentional excessive use of an IMP which is accompanied by harmful physical or psychological effects (e.g., overdose with the intention to cause harm)

Note: Medication error, misuse and abuse must always be reported on an AE form and a specific event form must be completed. The AE diagnosis on the AE form must reflect what occurred (e.g., accidental overdose, intentional overdose or other). If the medication error and/or misuse and abuse resulted in a clinical consequence, this must be reported on an additional AE form.

10.3.4 Recording and follow-up of AE and/or SAE

10.3.4.1 AE and SAE recording

The investigator will record all relevant AE/SAE information in the eCRF.

The investigator will attempt to establish a diagnosis of the event based on signs, symptoms, and/or other clinical information. In such cases, the diagnosis (not the individual signs/symptoms) will be documented as the AE/SAE.

When an AE/SAE occurs, it is the responsibility of the investigator to review all documentation (e.g., hospital progress notes, laboratory and diagnostics reports) related to the event.

There may be instances when copies of source documents (e.g., medical records) for certain cases are requested by Novo Nordisk. In such cases, all participant identifiers, with the exception of the subject ID, must be redacted on the copies of the source documents before submission to Novo Nordisk.

For all non-serious AEs, the applicable forms should be signed when the event is resolved or at the end of the study at the latest. For sign-off of SAE-related forms, refer to "AE and SAE reporting via paper CRF" later in this section.

Novo Nordisk products used as concomitant medication: if an AE is considered to have a causal relationship with a Novo Nordisk marketed product used as concomitant medication in the study, it is important that the suspected relationship is reported to Novo Nordisk, e.g., in the alternative aetiology section on the safety information form. Novo Nordisk may need to report this adverse event to relevant regulatory authorities

10.3.4.2 Assessment of severity

The investigator will assess severity for each event reported during the study and assign it to one of the following categories:

- **Mild**: An event that is easily tolerated by the participant, causing minimal discomfort and not interfering with everyday activities.
- **Moderate**: An event that causes sufficient discomfort and interferes with normal everyday activities.
- **Severe**: An event that prevents normal everyday activities.
  Note: An AE that is assessed as severe should not be confused with an SAE. Both AEs and SAEs can be assessed as severe.

10.3.4.3 Assessment of causality

The investigator is obligated to assess the relationship between IMP and the occurrence of each AE/SAE. The investigator will use clinical judgment to determine the relationship.

Relationship between an AE/SAE and the relevant IMP should be assessed as:

- **Probable** - Good reason and sufficient documentation to assume a causal relationship.
- **Possible** - A causal relationship is conceivable and cannot be dismissed.
- **Unlikely** - The event is most likely related to aetiology other than the IMP.

Alternative aetiology, such as underlying disease(s), concomitant medication, and other risk factors, as well as the temporal relationship of the event to IMP administration, should be considered and investigated.

The investigator should use the investigator's brochure for the assessment. For each AE/SAE, the investigator must document in the medical records that he/she has reviewed the AE/SAE and has provided an assessment of causality.

There may be situations in which an SAE has occurred, and the investigator has minimal information to include in the initial report. However, **it is important that the investigator always**

makes an assessment of causality for every event before the initial transmission of the SAE data.

The investigator may change his/her opinion of causality, in light of follow-up information, and update the causality assessment in the eCRF.

The causality assessment is one of the criteria used when determining regulatory reporting requirements

10.3.4.4 Final outcome

The investigator will select the most appropriate outcome:

- **Recovered/resolved**: The participant has fully recovered, or by medical or surgical treatment the condition has returned to the level observed when first documented
- **Recovering/resolving**: The condition is improving, and the participant is expected to recover from the event. This term may also be applicable for AEs ongoing at the time of death (where death was due to another AE).
  Note: For SAEs, this term is only applicable if the participant has completed the follow-up period and is expected to recover.
- **Recovered/resolved with sequelae**: The participant has recovered from the condition but with lasting effect due to a disease, injury, treatment or procedure. If a sequela meets an SAE criterion, the AE must be reported as an SAE.
- **Not recovered/not resolved**: The condition of the participant has not improved, and the symptoms are unchanged, or the outcome is not known. This term may be applicable in cases of chronic conditions, cancer or AEs ongoing at time of death (where death is due to another AE).
- **Fatal**: This term is only applicable if the participant died from a condition related to the reported AE. Outcomes of other reported AEs in a participant before he/she died should be assessed as ‘recovered/resolved’, ‘recovering/resolving’, ‘recovered/resolved with sequelae’ or ‘not recovered/not resolved’. An AE with a fatal outcome must be reported as an SAE.
- **Unknown**: This term is only applicable if the participant is lost to follow-up

10.3.4.5 Follow-up of AE and SAE

The investigator is obligated to perform or arrange for the conduct of supplemental measurements and/or evaluations as medically indicated or as requested by Novo Nordisk to elucidate the nature and/or causality of the AE or SAE as fully as possible (e.g., severe hypersensitivity reactions, Hy’s law). This may include additional laboratory tests or investigations, histopathological examinations, or consultation with other health care professionals.

If a participant dies during participation in the study or during a recognised follow-up period, the investigator should if possible and upon request, provide Novo Nordisk with a redacted copy of the autopsy report including histopathology.

New or updated information should be recorded in the eCRF.

10.3.5 Reporting of SAEs

AE and SAE reporting via CRF

Relevant forms must be completed in the CRF.

For SAEs, initial notification via telephone is acceptable, although it does not replace the need for the investigator to complete the AE and safety information forms within the designated reporting timelines (see Figure 10-1):

- AE form within 24 hours.
- Safety information form within 5 calendar days.
- Both forms should be signed within 7 calendar days after first knowledge by the investigator.
- Specific event form within 14 calendar days.
- For timelines related to events for adjudication, refer also to Appendix 7 (Section 10.7).

If the eCRF is unavailable for more than 24 hours, then the sites will use the paper AE form, and if the eCRF is unavailable for more than 5 calendar days, then the site will use the paper safety information form. The site should enter the SAE data in the eCRF as soon as it becomes available.

The relevant CRF forms (AE and safety information forms) must be forwarded to Novo Nordisk in accordance with Section 10.1.5.

After the study is completed, the study database will be locked, and the CRF will be decommissioned to prevent the entry of new data or changes to existing data. If a site receives a report of a new SAE from a participant or receives updated information on a previously reported SAE after CRF decommission, the site can report this information on a paper AE and safety information form (see below) or to Novo Nordisk by telephone.

Figure 10-1 Decision tree for determining the event type and the respective forms to complete with associated timelines

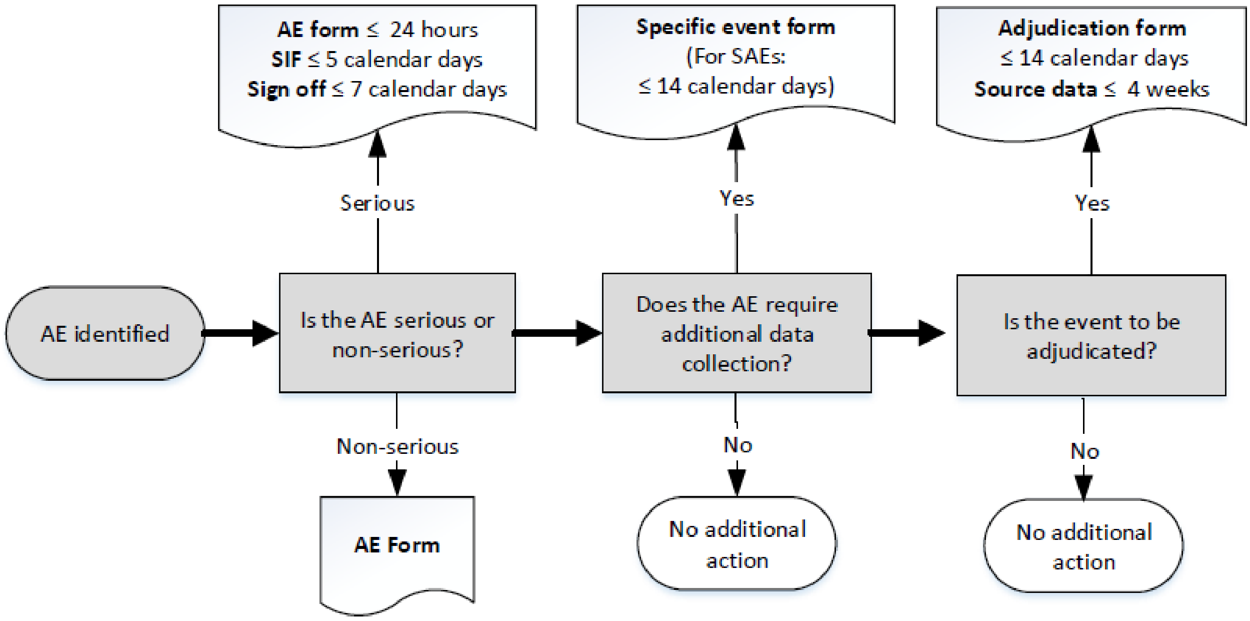

- **Timelines** are from the awareness of an AE.
- **Queries and follow-up** requests to be resolved ≤ 14 calendar days.
- In general data must be recorded in the CRF as soon as possible, preferably within 5 working days (see Appendix 1)

AE: Adverse Events, SAE: Serious Adverse Events, SIF: Safety Information Form

For further information on events for adjudication, refer to Appendix 7 (Section 10.7).
If the event adjudication system (EAS) is not available for document upload, the investigator should ensure that the relevant source documents are collected and saved locally until the EAS is available again.

Contact details for SAE reporting can be found in the investigator trial master file.

10.4 Appendix 4: Contraceptive guidance and collection of pregnancy information

10.4.1 Definitions

Woman of childbearing potential (WOCBP)

A woman is considered fertile following menarche and until becoming postmenopausal unless permanently sterile.
If fertility is unclear (e.g., amenorrhea in adolescents or athletes), and a menstrual cycle cannot be confirmed before first dose of study intervention, additional evaluation should be considered.
It must be recorded in the eCRF whether female participants are of childbearing potential.

Females in the following categories are not considered WOCBP

1. Premenarcheal
2. Females with one or more of the following:
   - Documented total hysterectomy
   - Documented bilateral salpingectomy
   - Documented bilateral oophorectomy

   For females with permanent infertility due to an alternate medical cause other than the above (e.g., Müllerian agenesis, androgen insensitivity), investigator discretion should be applied in determining study enrolment.
3. Postmenopausal female:
   - A postmenopausal state is defined as amenorrhoea for at least 12 months without an alternative medical cause in a female > 45 years of age. Alternative medical causes for amenorrhoea include, but are not limited to, hormonal contraception or hormonal replacement therapy.
   - Females ≥ 60 years of age can be considered postmenopausal.

Females on HRT and whose menopausal status is in doubt are considered of childbearing potential and will be required to use one of the highly effective contraception methods.

Note: Documentation regarding categories 1-3 can come from the site staff's review of participant's medical records, medical examination or medical history interview.

10.4.2 Contraceptive guidance

Male participants

No contraception measures are needed for male participants as the risk of teratogenicity/fetotoxicity caused by transfer of NNC6019-0001 in seminal fluid is unlikely.

Female participants

Female participants of childbearing potential are eligible to participate if they agree to use methods of contraception consistently and correctly. Table 10-3 lists the highly effective methods of contraception allowed. Local regulations may apply.

Portugal, Spain: For country-specific requirements, please refer to Appendix 8 (Section 10.8).

Highly effective contraception should be utilised for a least 16 weeks after last dose of IMP (corresponding to time during treatment and until the end of relevant systemic exposure).

Table 10-3 Highly effective contraceptive methods allowed[53]

Highly effective methods[a] (Failure rate of <1% per year when used consistently and correctly):

- Combined (estrogen- and progestogen-containing) hormonal contraception associated with inhibition of ovulation[b]
  - oral
  - intravaginal
  - transdermal
- Progestogen-only hormone contraception associated with inhibition of ovulation
  - oral
  - injectable
  - implantable
- Intrauterine device (IUD)
- Intrauterine hormone-releasing system (IUS)
- Bilateral tubal occlusion
- Vasectomized partner
  Vasectomized partner is a highly effective contraceptive method provided that the partner is the sole sexual partner of the woman of childbearing potential, and the absence of sperm has been confirmed. If not, an additional highly effective method of contraception should be used. Spermatogenesis cycle is approximately 90 days.
- Sexual abstinence
  Sexual abstinence is considered a highly effective method only if defined as refraining from heterosexual intercourse during the entire period of risk associated with the study intervention. The reliability of sexual abstinence needs to be evaluated in relation to the duration of the study and the preferred and usual lifestyle of the participant.

[a]Contraceptive use by men or women should comply with local regulations regarding the use of contraceptive methods for those participating in clinical studies; [b]If locally required, in accordance with Clinical Trial Facilitation Group (CTFG) guidelines, acceptable contraceptive methods are limited to those which inhibit ovulation as the primary mode of action.

The following methods are not acceptable methods of contraception: Periodic abstinence (calendar, symptothermal, post-ovulation methods), withdrawal (coitus interruptus), spermicides only, and lactational amenorrhoea method (LAM).

10.4.3 Collection of pregnancy information

Female participants who become pregnant

Investigator will collect pregnancy information on any female participant who becomes pregnant while participating in this study.

Information will be recorded on the appropriate form and submitted to Novo Nordisk within 14 calendar days of learning of a participant's pregnancy (see Figure 10-2).

The participant will be followed to determine the outcome of the pregnancy. The investigator will collect follow-up information on participant and neonate which will be forwarded to Novo Nordisk within 14 calendar days. Generally, follow-up will not be required for longer than 1 month beyond the delivery date.

Any termination of pregnancy will be reported, regardless of foetal status (presence or absence of anomalies) or indication for procedure.

While pregnancy itself is not considered to be an AE or SAE, any adverse event in connection with pregnancy or elective termination of a pregnancy for medical reasons will be reported as an AE or SAE. If relevant, consider adding 'gestational', 'pregnancy-related' or a similar term when reporting the AE/SAE.

Pregnancy outcome should be documented in the participant's medical record. Abnormal pregnancy outcome (e.g., spontaneous abortion, foetal death, stillbirth, congenital anomalies and ectopic pregnancy) is considered an SAE. In case of abnormal pregnancy outcome, paternal information should be recorded in the appropriate form after obtaining the necessary signed paternal informed consent.

If the investigator learns of an SAE occurring as a result of a post-study pregnancy which is considered related to the IMP by the investigator, the SAE should be reported to Novo Nordisk as described in Appendix 3 (Section 10.3.)

Figure 10-2 Decision tree for determining the forms to complete for collection of pregnancy information and timelines for reporting – For female participants

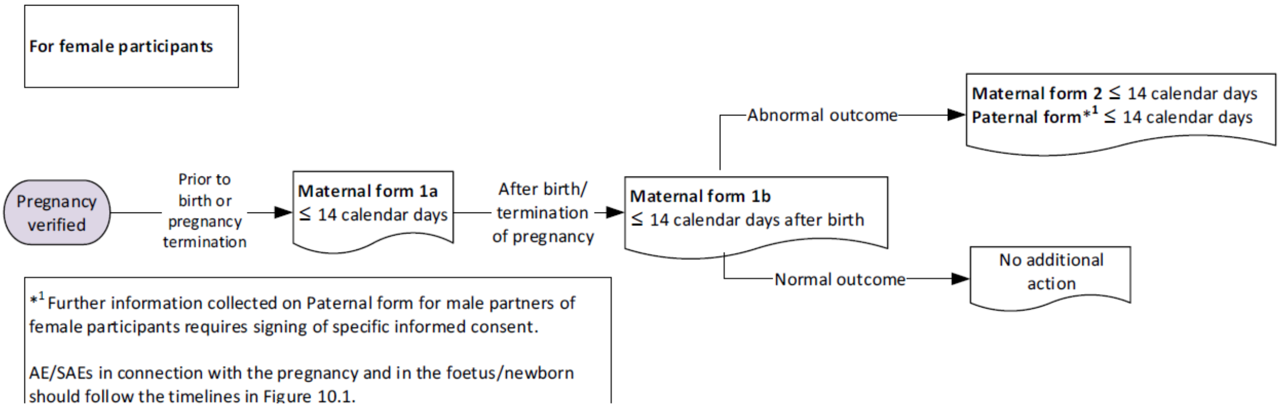

Any female participant who becomes pregnant while participating in the study will discontinue study intervention. Furthermore, study intervention must be discontinued if the female participant intends to become pregnant.

10.5 Appendix 5: Technical complaints: Definition and procedures for recording, evaluation, follow-up and reporting

10.5.1 Definition of technical complaint

A technical complaint is any written, electronic or oral communication that alleges product (medicine or device) defects. The technical complaint may be associated with an AE but does not concern the AE itself.

Examples of technical complaints:
- Problems with the physical or chemical appearance of study interventions (e.g., discoloration, particles or contamination).
- Problems with packaging material including labelling.

Time period for detecting technical complaints

All technical complaints which occur from the time of receipt of the product at site until the time of the last usage of the product must be collected for products predefined on the technical complaint form.

10.5.2 Recording and follow-up of technical complaints

Reporting of technical complaints to Novo Nordisk

For contact details for Customer Complaint Center, please refer to Attachment I.

Technical complaints on products allocated to a participant must be reported on a separate technical complaint form:
1. For products with DUN: One technical complaint form must be completed for each affected DUN.
2. For products without DUN: One technical complaint form must be completed for each batch, code or lot number.

DUN is the same as the term Kit IDs in the systems and manuals.

Timelines for reporting technical complaints to Novo Nordisk

The investigator or the investigator´s unblinded designee must complete the technical complaint form and ensure it is forwarded to Customer Complaint Center, Novo Nordisk, within:
- 24 hours if related to an SAE
- 5 days calendar for all other technical complaints

Follow-up of technical complaints

The investigator is responsible for ensuring that new or updated information will be recorded on the originally completed form.

Collection, storage and shipment of technical complaint samples

The unblinded personnel must collect the technical complaint sample and all associated parts and notify the unblinded monitor within 5 calendar days of obtaining the sample at site. The sample and

all associated parts must be sent as soon as possible to Customer Complaint Center, Novo Nordisk, together with a copy of the completed technical complaint form.

The technical complaint sample should contain the batch, code or lot number and, if available, the DUN (DUN is the same as the term Kit IDs in the systems and manuals). If the technical complaint sample is unobtainable, the reason must be stated on the technical compliant form.

If several samples are shipped in one shipment, the sample and the corresponding technical complaint form should be kept together.

Storage for the technical complaint sample must be done in accordance with the conditions prescribed for the trial product.

10.5.3 Reporting of technical complaints for products not included in the technical complaint form

Technical complaints on products not included in the technical complaint form should be reported to manufacturing holder.

10.6 Appendix 6: Retention of human biosamples for future research

Human biosamples (also in some cases known as human biospecimen or human biological materials) are samples that have been taken from the human body during life or after death. It includes:

- Primary cells, tissues, organs or cell containing fluids of human origin (for example, whole blood, urine, saliva, synovial fluid)
- Cell free fluids of primary human origin (for example, serum and plasma)
- Extracts or derivatives of the above, when derived by purification (for example, DNA, RNA, proteins, membranes, microsomes and other cellular substructures).

10.6.1 Biosamples for future research

Participants who do not wish to contribute with biosamples for storage may still participate in the study. Participants must sign and date a separate informed consent form before biosamples are collected to be stored for future analysis.

In countries where allowed, the study will involve collection of human biosamples for future research to be stored in a central laboratory facility for future use. Serum, plasma, whole blood and urine will be stored, and timing of sampling and amount of material to be stored are specified in Table 10-4.

Table 10-4 Type of material, timing of sampling and amount of material to be stored for future research

Type of material	Time of sampling[a]	Material to be stored
Serum[b]	Randomisation, V3, V5, V8, V15	5 x 0.5 mL aliquots per sample
Plasma[b]	Randomisation, V3, V5, V8, V15	5 x 0.5 mL aliquots per sample
Whole blood[c]	Randomisation, V15	1 x 2.5 mL aliquot per sample
Urine spot samples[d]	Randomisation, V15	2 x 1 mL aliquot per sample

[a] Timing of sampling is also specified in the flowchart (Section 1.2) and Appendix 2 (Section10.2).
[b] Sampled for non-genetic analyses (e.g. hormones, metabolites or similar)
[c] Whole blood collected for analysis of DNA and RNA
[d] For assessment of biomarkers or similar accumulating in urine.

Abbreviations: V = visit

The biosamples will be stored at a central laboratory, at a central storage facility or an analysing laboratory contracted by Novo Nordisk for up to 15 years after end of study. Only relevant Novo Nordisk, consultants, auditors, research organisations or laboratories working for or collaborating with Novo Nordisk as well as storage facility employees will be able to access the stored biosamples and associated data. The biosamples may be transferred to other countries for analysis and will be destroyed at the latest 15 years after end of study.

The analyses of the biosamples for future research are not intended to identify participant-specific findings, but to understand and predict response to NNC6019-0001 and related conditions on a population level.

Analysis will be done on the biosamples and associated data (data relating to the test results or results from the main study).

Novo Nordisk will ensure that third party collaborators live up to the regulations on data protection, see Appendix 1 (Section 10.1.5).

The participant may request the stored biosamples for future research to be destroyed by withdrawing the designated informed consent at any timepoint during and after the study. For samples that have already been analysed, the results can still be used for scientific research and will not be removed from the datafile.

10.6.2 Anti-NNC6019-0001-antibodies samples

Remaining and residual antibody samples (see Section 8.8.1) already collected may be retained after end of study.

- The samples will be stored at Novo Nordisk or a biorepository assigned by Novo Nordisk after end of study and until marketing authorisation approval or until the research project terminates, but no longer than 15 years from the end of study after which they will be destroyed.
- Only relevant Novo Nordisk staff and consultants, auditors, research organisations or laboratories working for Novo Nordisk and biorepository personnel will have access to the stored samples and associated data.
- The samples may be transferred to other countries for analysis, if not prohibited by local regulations, and will be destroyed at the latest 15 years after end of study.
- The identity of study participants will remain confidential, and the samples will be identified only by subject ID, visit number and study identification number. No direct identification of the participant will be stored together with the samples.

The retained samples may be used to:

- Evaluate safety or efficacy aspects that address concerns arising during or after the study.
- Further characterise the antibody responses towards the drug, if required by health authorities or for safety reasons.
- Conduct further analytical method development and validation of antibody assays.
- Genetic analyses will not be performed on these samples.

10.6.3 Hypersensitivity reaction samples

In order to comply with any future requests from health authorities to further characterise the antibody response, antibody samples collected in relation to suspicion of a severe immediate systemic hypersensitivity reaction[54] (Section 8.8.2) may be retained.

The samples will be stored at Novo Nordisk or a Novo Nordisk designated referral central biorepository. The samples might be transferred to other countries, if not prohibited by local regulations. Only Novo Nordisk staff and bio-repository personnel will have access to the stored samples. The samples may be shipped to a contract research organisation (CRO) for analysis.

The samples will be anonymised (identified only by subject ID, visit number, study identification number and sampling date). Confidentiality and personal data protection will be ensured during storage after the end of study and no direct identification of the participant will be stored together with the samples.

Potential further analyses of the samples will not have any consequences for the participant and their relatives. Participants can contact the investigator if they wish to be informed about results derived from stored antibody samples obtained from their own body.

The samples will be stored after end of study and until marketing authorisation approval or until the research project terminates, but no longer than 15 years from end of study after which they will be destroyed.

10.6.4 Pharmacokinetic and pharmacodynamic samples

Residual plasma samples used for bioanalysis of NNC6019-0001 as well as for misfolded TTR biomarker analysis may be stored at Novo Nordisk after finalisation of the CSR. The residual PK samples will only be used for potential NNC6019-0001 assay investigation/validation and exploratory metabolite analysis, if deemed relevant for interpretation of the results within this study. Residual biomarker samples for misTTR analysis may be used for potential further characterisation of biomarkers and assay investigations. Samples will be stored for up to 5 years from end of study, after which the samples will be destroyed. Only Novo Nordisk A/S or relevant special laboratories will have access to the samples.

The samples will be pseudonymized (identified only by a unique sample ID, visit number, study identification number and sampling date). Confidentiality and personal data protection will be ensured during storage after the end of study and no direct identification of the participant will be stored together with the samples.

If an assay investigation or exploratory metabolite investigation is performed using residual PK, and/or biomarker samples, the data derived from this investigation will be documented independently from the CSR.

10.7 Appendix 7: Events requiring adjudication

Event adjudication will be performed in randomised participants. An event for adjudication is a selected AE or death evaluated by an independent external Event adjudication committee (EAC) in a blinded manner, please refer to Table 10-5 for event types in scope.

For details on the EAC, refer to Appendix 1 (Section 10.1.6.4).

Table 10-5 AEs requiring event adjudication

Event type<br>(serious and non-serious AEs)	Description
Death	All cause death
Cardiovascular hospitalisation	All cause hospitalisation
Urgent heart failure visit not requiring hospitalisation	New episode or worsening of existing heart failure leading to an urgent, unscheduled clinic/office/emergency department visit

There are five ways to identify events relevant for adjudication as described below:

1. Investigator-reported events for adjudication: investigator selects the appropriate event type relevant for adjudication (see Table 10-5).
2. AEs reported with fatal outcome
3. AEs reported as requiring hospitalisation
4. AE search (standardised screening): All AEs not reported with an event type relevant for adjudication will undergo screening to identify potential urgent heart failure visit not requiring hospitalisation events for adjudication. Investigators will be notified of these events in the eCRF.
5. EAC-identified events: Unreported events relevant for adjudication identified by the EAC during review of source documents provided for another event for adjudication. Investigators will be notified of these events in the eCRF and has the option to report the EAC-identified event.

For each event relevant for adjudication, an event type specific adjudication form should be completed in the eCRF within 14 days (Figure 10-1).

Copies of source documents should be uploaded to the event adjudication system (EAS) as soon as possible and preferably within 4 weeks (Figure 10-1). In cases where the EAS is not accessible for document upload, the investigator should ensure that the relevant source documents are collected and saved locally until the EAS is available. If no, or insufficient source documents are provided to the adjudication supplier, the investigator can be asked to complete a clinical narrative to be uploaded to the EAS.

If new information becomes available for an event sent for adjudication, it is the responsibility of the investigator to ensure the new information is uploaded to the EAS.

An Event Adjudication Site Manual will be provided to each site detailing which source documents are relevant and how these should be provided to the adjudication supplier. The anonymisation and labelling requirements are also described in the event adjudication site manual.

10.8 Appendix 8: Country-specific requirements

Czech Republic:

- Section 6.1: All participants in the Czech Republic must be monitored at the study site for at least 2 hours (+15 min) after infusion of the 2nd and 3rd doses of trial product. Vital signs to be measured at 10 min (±1 min), 60 min (±10 min) and 2 hours (+15 min) after infusion. From 4th dose and on, all participants must be monitored for at least 90 min (±10 min) after infusion.
- Section 8.3.8: For women of childbearing potential, a pregnancy test (preferably a highly sensitive urine hCG pregnancy test) must be performed at every visit before administration of trial product.
- Appendix 1 (Section 10.1.3). Informed consent process: Participant's electronic signature is not permitted.

France:

- Sections 1.2 and 8.1: Race and ethnic origin can only be collected if purpose of the research is justified. Therefore, in this trial for the central laboratory calculation of the eGFR, information on race (black/white/other) and year of birth will be collected on the laboratory requisition form only.
- Sections 1.2 and 8.1. Date of birth: Only year is collected for the date of birth.
- Appendix 1 (Section 10.1.1). Regulatory and Ethical Considerations: The inclusion of people at risk is not allowed (persons under guardianship or Curatorship). Affiliation to a "sécurité sociale" regime is mandatory.
- Appendix 1 (Section 10.1.13). Indemnity statement: The French Public Health Code article L 1121-10 (law n° 2004-806 of 9 August 2004 art. 88 I,IX, Journal Official of 11 August 2004. "The sponsor is responsible for identification of the harmful consequences of the biomedical the research for the person lending himself thereto and for indemnification of his beneficiaries, except in case of proof, incumbent on it, that the prejudice is not attributable to his fault of the fault of any intervening party, without the sponsor's being entitled to call on acts by a third party or the voluntary withdrawal of the person who had initially consented to cooperating in the research".

Germany:

- Sections 1.2 and 8.1, Participant's full date of birth is not allowed to be collected and must be shortened to year of birth.

Japan:

- Section 6.1: The actual dose of diphenhydramine (or H1 antihistamine) and acetaminophen for premedication may be decided by the investigator in consultation with Novo Nordisk.
- Section 6.1: For this study, drugs used in the clinical study except IMPs are diphenhydramine and acetaminophen. Please refer to local package insert for the latest information.
- Section 6.3.1: Stratification by disease type will not be applied for Japanese participants.
- Sections 6.1 and 8.3.6: Additional safety measures will be applied for Japanese participants:
    - Japanese participants will be dosed after the safety confirmation based on unblinded data evaluation by the external, independent Data Monitoring Committee (DMC) for at least 9 participants completing the 28-days sentinel phase. In addition, intensified safety monitoring will be instituted for the first 6 randomised Japanese participants as outlined below:

- The first 6 randomised Japanese participants will be observed on site under continuous cardiac monitoring for at least 24 hours after start of their first dose. On discharge, participants will be monitored using continuous cardiac monitoring on an out-patient basis until 7 days after start of the infusion. In addition, the first 6 randomised Japanese participants will have weekly visits with safety assessment as defined in the flowchart for Visit 2A, 2B, 2C and 2D during the first 28 days. Thereafter, the first 6 randomised Japanese participants will continue to be dosed Q4W for the full duration of the study.
- Throughout the study, a comprehensive, risk-based medical monitoring will evaluate blinded safety data on a regular basis. In addition, a DMC will review and evaluate accumulated unblinded data (including the Japanese participants) at predefined time intervals as well as ad hoc. The DMC will continue to monitor the trial based on unblinded data until trial closure and will give their recommendation on trial continuation, modification, or termination.

- Section 6.2: Preparation/Handling/Storage/Accountability: The head of the study site or the trial product storage manager assigned by the head of the study site (a pharmacist in principle) is responsible for control and accountability of the trial products.
- Appendix 1 (Section 10.1.7). Dissemination of clinical study data: The study will be registered at www.jrct.niph.go.jp.

Netherlands:

- Sections 1.2 and 8.1: Date of birth: participant's full day of birth can only be used if there is a strong need related to the hypothesis of the study. Generally, only year of birth is to be used.

Portugal:

- Appendix 4 (Section 10.4). Contraception requirements based on the Recommendations related to contraception and pregnancy testing in clinical trials from Clinical Trial Facilitation Group (CTFG).

Spain:

- Appendix 1 (Section 10.1.5). Data protection: This study will be conducted in line with European Regulation (EU) 2016/679 of the European Parliament and of the Council of 27 April 2016 on data protection GDPR.
- Appendix 1 (Section 10.1.13). Indemnity statement: Novo Nordisk accepts liability in accordance with Article 10 "Liability regime" of the Royal Decree 1090/2015 of 4 December.
- Appendix 4 (Section 10.4) and Section 5.2. Contraception requirements based on the Recommendations related to contraception and pregnancy testing in clinical trials from Clinical Trial Facilitation Group (CTFG).

United States:

- Appendix 1 (Section 10.1.1). Regulatory, ethical, and study oversight considerations: FDA form 1572:
  For US sites:
  - Intended for US sites
  - Conducted under the IND
  - All US investigators, as described above, will sign FDA Form 1572

  For sites outside the US:

- Intended for participating sites outside of the US
- Not conducted under the IND
- All investigators outside of the US will not sign FDA form 1572

Novo Nordisk will analyse and report data from all sites together if more than one site is involved in the trial.

- Appendix 1 (Section 10.1.10). Retention of clinical study documentation: In the United States, 21 CFR 312.62(c) and 21 CFR 812.140(d) require 2 years following the date a marketing application is approved for the drug for the indication for which it is being investigated; or, if no application is to be filed or if the application is not approved for such indication, until 2 years after the investigation is discontinued and FDA is notified'.
- Appendix 2 (Section 10.2): Clinical laboratory tests: For haematology samples (differential count) where the test result is not normal, then a part of the sample may be kept for up to two years or according to local regulations.

10.9 Appendix 9: Abbreviations

ADA	anti-drug antibodies
AE	adverse event
ALT	alanine aminotransferase
AST	aspartate aminotransferase
ATTR	transthyretin amyloid
AV	atrioventricular
COA	clinical outcome assessment
COVID 19	Corona virus disease 2019
CM	cardiomyopathy
CRF	case report form
CSR	clinical study report
CTFG	clinical trial facilitation group
CV	cardiovascular
DBL	database lock
DMC	Data Monitoring Committee
DNA	deoxyribonucleic acid
DPD	3,3-diphosphono-1,2-propanodicarboxylic acid
DPS	data points set
DUN	dispensing unit number
EAC	Event Adjudication Committee
EAS	event adjudication system
ECG	electrocardiogram
eCRF	electronic case report form
ECV	extracellular volume
eGFR	estimated glomerular filtration rate
FAS	full analysis set
FDA	U.S. Food and Drug Administration
FDAAA	FDA Amendments Act
FHD	first human dose
GCP	Good Clinical Practice
GLS	global longitudinal strain
hATTR	hereditary ATTR
HFpEF	heart failure with preserved ejection fraction
HIV	Human Immunodeficiency Virus
HMDP	hydroxymethylene diphosphonate

HR	hazard ratio
HRT	hormone replacement therapy
IB	investigator's brochure
ICH	International Council for Harmonisation
IEC	independent ethics committee
IMP	investigational medicinal product
IND	investigational new drug
INR	international normalised ratio
IRB	institutional review board
KCCQ	Change in Kansas City Cardiomyopathy Questionnaire
LPLV	last participant last visit
MAP	modelling analysis plan
MR	magnetic resonance
MRI	magnetic resonance imaging
6MWT	6 Minute Walk Test
NIMP	non-investigational medicinal product
NIS	neuropathy impairment score
NSF	nephrogenic systemic fibrosis
NT-proBNP	N-terminal-pro brain natriuretic peptide
NYHA	New York Heart Association
PAS	participant analysis set
PCD	primary completion date
PD	pharmacodynamics
PGI-S	Patient Global Impression of Status
PIN	prostatic intraepithelial neoplasia
PK	pharmacokinetics
PN	polyneuropathy
PRO	patient reported outcome
PYP	pyrophosphate
RNA	ribonucleic acid
RR	relative risk
RTSM/IWRS	randomisation and trial supplies management system / interactive web response system
SAE	serious adverse event
SAP	statistical analysis plan
SOP	standard operating procedure
SPECT	single-photon emission computed tomography
SUSAR	suspected unexpected serious adverse reaction

TEAE	treatment emergent adverse event
TMM	Trial Materials Manual
TTR	transthyretin
VAS	visual analogue scale
WOCBP	woman of childbearing potential
wtATTR	wild-type ATTR

10.10 Appendix 10: Protocol amendment history

The Protocol amendment summary of changes table for the current protocol version is located directly before the table of contents.

Protocol version 5.0 (28 April 2023)

This amendment is considered to be non-substantial based on the criteria set forth in Article 10(a) of Directive 2001/20/EC of the European Parliament and the Council of the European Union,[2] because it neither substantially impacts the safety or rights of the participants nor the reliability or robustness of the data being generated in the study.

Overall rationale for preparing protocol, version 5.0:

The overall rationale for preparing protocol version 5.0 is to introduce more flexibility for the study sites. It is specified that visits can be split across 2 or more days, provided that all assessments are completed prior to dosing and within the given visit window. In addition, minor changes have been made for completeness and correctness.

Deleted text is written as ~~strikethrough~~ and new text *italic*.

Section # and name	Description of change	Brief rationale
Throughout document	Country-specific requirements for United Kingdom removed throughout the protocol.	United Kingdom will not be included in the study.
1.1 Synopsis	This is an interventional, randomised, multinational, multicentre, three-arm parallel-group, double blind, placebo-controlled study in participants with *hereditary ATTR (*hATTR*)* or *wild-type ATTR (*wtATTR*)* CM	Abbreviations spelled out for clarification
2.1 Study rationale	*In the future NN6019 development programme, the term hereditary ATTR will be updated to variant ATTR (ATTRv) and wild-type ATTR will be abbreviated to ATTRwt.[23, 24] These updated terms will be used in the reporting (CSR) of the current study, NN6019-4940.*<br><br>*23. J Am Coll Cardiol. 2023;81(11):1076-126.*<br>*24. Amyloid. 2020;27(4):217-22.*	To be aligned with international clinical consensus on precise terminology and nomenclature
2.2 Background	The predominant organ involvement for hATTR amyloidosis is either the nervous system ~~(hATTR PN amyloidosis),~~ *termed hATTR polyneuropathy (hATTR PN),* or the heart, *hATTR cardiomyopathy (hATTR CM)* ~~(hATTR CM amyloidosis)~~, although other organ systems are also often involved.<br><br>The predominant effect of wtATTR amyloidosis is on the heart, *wtATTR cardiomyopathy (wtATTR CM)*.	Wording updated for clarification

Section # and name	Description of change	Brief rationale
2.3.1 Risk assessment	Table 2-1 Risk assessment<br><br>Potential risk:<br>~~Pro-arrhythmic risk~~ *Cardiac arrhythmia*	The rationale for changing the naming of the risk from pro-arrhythmia to cardiac arrhythmia is to ensure better data quality. The latter name more precisely describes the event types that needs additional data collection.
5.2 Exclusion criteria	Exclusion criterion 16:<br>International normalised ratio (INR)>1.5 (unless participant is on anticoagulant therapy[c], in which case excluded if INR>3.5)[d].<br><br>Footnotes:<br>[c] *Vitamin K antagonists i.e. warfarin, acenocoumarol etc.;* [d] *Criterion not applicable for participant on therapy with direct-acting oral anticoagulants (DOACs).*	exclusion criteria #16 has been adjusted to accommodate current clinical practice and SMPCs for the relevant anticoagulants. For patients on direct-acting oral anticoagulants. INR is not reliable for assessing the anticoagulation effects of these agents and is not used in clinical practice to monitor anticoagulatory effect of the medication.
6.1 Study interventions administered	*Novo Nordisk will provide infusion/blinding cover bags to EU countries.*<br><br>*Other* auxiliaries needed for preparation and administration of each dose will not be provided by Novo Nordisk.	Approved infusion cover bags are not available for purchase in EU countries outside of DK.
6.2 Preparation, handling, storage and accountability	*All vials should be saved to allow for trial product reconciliation by the unblinded monitor, unless not permissible per site SOP. The site SOP should outline the local process for accountability documentation and destruction policy. The unblinded monitor should perform the reconciliation of the trial product based on the site accountability records.*	Added to accommodate that some pharmacies cannot comply with keeping vials for monitor accountability due to local SOP requirements.
7.2 Participant discontinuation/withdrawal from the study	If a participant withdraws consent ~~as registered in the RTSM/IWRS~~ between randomisation and prior to first dosing administration, the participant will not be asked to have any follow-up assessments performed.	Not registered in the RTSM/IWRS.
7.3 Lost to follow-up	If a participant is lost to follow-up ~~as registered in the RTSM/IWRS~~ between randomisation and prior to first dosing administration, the above listed actions are not required.	Not registered in the RTSM/IWRS.

**Section # and name**	**Description of change**	**Brief rationale**
8 Study assessments	• Assessments should be *performed* ~~carried out~~ according to the standard of care unless otherwise specified in the current section*, and all assessments should preferably be performed on the same day.* Efforts should be made to limit bias between assessments. *When all assessments are performed on the same day, efforts should be made to perform the assessments in the following order:* ~~Assessments should be caried out in the following order:~~<br>1.Clinical outcome assessments *(COAs)*<br>(…)<br><br>• If all assessments pertaining to a visit cannot be performed on the same day, it is allowed to split the visit across 2 or more days, provided that all assessments are completed prior to dosing and within the given visit window. The suggested order of the COAs, including the PRO questionnaires as listed above, remains applicable. Specific visit window allowances are applicable to the cardiac MRI and echocardiography. Refer to Section 8.2.3.1 and 8.2.3.2 for the specific visit window details related to the cardiac MRI and echocardiography, respectively.	To increase flexibility for the sites.
8.2.3.2 Echocardiography	A standard echocardiographic examination should be performed at the timepoints outlined in the flowchart (Section 1.2), *and the examination can be performed up to 2 days prior to the respective visit.*	To increase flexibility for the sites.
8.3.4 Vital signs	*Body* ~~Ear~~ temperature, pulse rate, as well as systolic and diastolic blood pressure will be assessed and recorded as specified in the flowchart (Section 1.2) and Table 6-2.<br><br>Body temperature should be measured *as per local procedure at site* ~~in the ear~~ with a calibrated thermometer.	To increase flexibility for the sites.
8.4 Adverse events and other safety reporting	Table 8-4 AEs requiring additional data collection and events for adjudication<br><br>~~Pro-arrhythmic risk~~ *Cardiac arrhythmia*	The rationale for changing the naming of the risk from pro-arrhythmia to cardiac arrhythmia is to ensure better data quality. The latter name more precisely describes the event types that needs additional data collection.

Section # and name	Description of change	Brief rationale
10.2 Appendix 2: Clinical laboratory tests	Spelling mistake and cross-reference to footnote corrected:<br><br>~~Partiel Tromboplastin Time~~ *Partial Thromboplastin Time*<br><br>Antibodies ~~d~~ *e*	For correctness.
10.2 Appendix 2: Clinical laboratory tests	Footnote e added to total IgE[e], tryptase[e]<br><br>[e] Results from anti-drug antibodies, *tryptase* and TTR, will not be made available to investigators during study conduct.	For correctness
10.3.3 Description of AEs requiring additional data collection	~~Pro-arrhythmic risk~~ *Cardiac arrhythmia*<br><br>Cardiac rhythm disturbances, including atrial and/or ventricular tachy- and/or bradyarrhythmia will be collected to assess the ~~pro-arrhythmic~~ risk *of cardiac arrhythmia*, including:	The rationale for changing the naming of the risk from pro-arrhythmia to cardiac arrhythmia is to ensure better data quality. The latter name more precisely describes the event types that needs additional data collection.
10.3.3 Description of AEs requiring additional data collection	3.The development of new/a change ~~of~~ *or* worsening of the sinoatrial and atrioventricular conduction disorders.	To correct typo
10.9 Abbreviations	10.9 *Appendix 9:* Abbreviations	For correctness
10.9 Abbreviations	*SOP (standard operating procedure)*	New abbreviation introduced.

Protocol version 4.0 (09 Sep 2022), global

This amendment is considered to be substantial based on the criteria set forth in Article 10(a) of Directive 2001/20/EC of the European Parliament and the Council of the European Union[2]

Overall rationale for preparing protocol, version 4.0:

The overall rationale for preparing protocol version 4.0 is to change to lower dose levels. It has been decided to change the doses to 10 mg/kg and 60 mg/kg based on an update of the PK/PD model to accommodate both circulating as well as myocardial PK/PD factors:

- Greater binding affinity of NNC6019-0001 to aggregated misTTR compared to monomeric misTTR
- Lower expected concentration of NNC6019-0001 in cardiac tissue vs plasma

Consequently, the expanded PK/PD model anticipates a greater affinity to the TTR amyloid fibrils in the cardiac tissue compared to the affinity to the primarily monomeric misTTR in circulation. The updated model therefore supports the use of lower doses while still attaining similar levels of effect.

In addition, this version of the protocol includes a correction of inclusion criterion 11, an updated description of premedication, and a specification of required serology assessments.

Deleted text is written as ~~strikethrough~~ and new text *italic*.

Section # and name	Description of change	Brief rationale
Throughout	Dose levels of NNC6019-0001 have been changed: ~~30~~ *10* mg/kg and ~~100~~ *60* mg/kg	See rationale above the table
2.3.1 Risk assessment	Description of premedication has been updated:<br><br>To mitigate risk of hypersensitivity reactions, participants will be treated with histamine (H1) blockers and acetaminophen*/paracetamol* ~~(or paracetamol)~~ prior to receiving NNC6019-0001.	For alignment throughout the protocol
4.3 Justification for dose	Section has been updated with rationale for the selected dose levels of NNC6019-0001 (10 mg/kg and 60 mg/kg)	See rationale above the table
5.1 Inclusion criteria	Inclusion criterion 11 has been corrected:<br><br>Aspartate transaminase (AST) ~~or~~ *and* alanine transaminase (ALT) levels ≤ 2.5 × the upper limit of normal (ULN) ~~or~~ *and* total bilirubin ≤2 × ULN at screening.	For correctness
5.2 Exclusion criteria	Footnote c moved to Section 10.2, Appendix 2 (Table 10-2):<br><br>Evidence of current or chronic hepatitis C virus [e] or hepatitis B virus infection.<br><br>~~[e] As per current medical standards i.e. hepatitis C virus RNA testing needed only if the anti-hepatitis C virus screening is positive.~~	For consistency
6.1 Study interventions administered	Description of premedication has been updated:<br><br>25 mg diphenhydramine (or an equivalent dose of an H1 antihistamine) and 650-*1000* mg acetaminophen*/paracetamol* ~~(or an equivalent paracetamol dose)~~	To comply with different marketed doses of acetaminophen/paracetamol across countries
10.2 Appendix 2: Clinical laboratory tests	Table 10.2: Serology assessments have been specified:<br>• HIV antibody [c]<br>• *HIV antigen* [c]<br>• *Hepatitis B core antibody (HBcAb)*<br>• Hepatitis B surface antigen (HBsAg)<br>• Hepatitis C virus antibody [d]<br><br>[c] *Additional HIV serology may be performed if needed;* [d] *As per current medical standards i.e. hepatitis C virus RNA testing needed only if the anti-hepatitis C virus screening is positive*	For accuracy and to comply with diagnostic criteria for HIV and Hepatitis B testing

Protocol version 3.0 (22 July 2022), global

This amendment is considered to be non-substantial based on the criteria set forth in Article 10(a) of Directive 2001/20/EC of the European Parliament and the Council of the European Union,[2] because it neither significantly impacts the safety nor physical/mental integrity of subjects nor the scientific value of the trial.

Overall rationale for preparing protocol, version 3.0:

The overall rationale for the changes implemented in the amended protocol is to clarify study-related procedures, to address local requirements in Japan and the Netherlands, and to make editorial changes for completeness and correctness.

Deleted text is written as ~~strikethrough~~ and new text *italic*.

**Section # and name**	**Description of change**	**Brief rationale**
Global edit	~~6 MWT~~ changed to *6MWT*	For consistency with literature
1.2 Flowchart	Cross reference to Section 8.1 added for Informed Consent and Demography	For completeness
1.2 Flowchart	Cross reference to footnote 'b' added for cardiac MRI at V3 (week 4).	The number of MRI scans is reduced from 5 to 4 for participants in the main cohort, to reduce the burden on participants and to reduce overall exposure to MRI contrast agent
1.2 Flowchart	Correction of protocol section number from ~~8.9~~ to *8.8.3* and update of description of biosamples to specify blood:<br>Biosamples *(blood)* for Future Analysis Taken<br>Biosamples *(blood)* for Genetic Analysis Taken<br>Urine samples for future Analysis Taken	For correctness
2.1 Study rationale	*NNC6019-0001 is a recombinant product produced using a Chinese hamster ovary-derived cell line.*	Requirement of health authority in Japan
2.3.1 Risk assessment	Name of study intervention in Table 2-1 corrected to NN~~N~~C6019-0001	For correctness
5.2 Exclusion criteria	Cross reference to section number added for exclusion criterion #3:<br><br>Female who is pregnant, breast-feeding or intends to become pregnant or is of childbearing potential and not using highly effective contraceptive method, as defined in Appendix 4 *(Section 10.4)*.	For correctness

**Section # and name**	**Description of change**	**Brief rationale**
6.1 Study interventions administered	Footnote added for Table 6-1:<br><br>[c]*Japan: For country specific requirements, please refer to Appendix 8 (Section 10.8).*	Requirement of health authority in Japan
8 Study assessments	Participants are not required to attend any visits in a fasting state. The time since last meal is collected when blood samples are taken*, as applicable*.	To specify further since time since last meal is not collected for every blood sample
8.2.2.2 Neuropathy Impairment Score (NIS)	*The NIS assessment can be performed within ± 2 weeks of the respective visit, except for the first dosing visit where it can be performed up to 2 weeks in advance (full eligibility needs to be confirmed beforehand).*	To allow flexibility for participants and sites
8.2.3.1 Cardiac magnetic resonance imaging (MRI)	*Measurement of haematocrit for the calculation of ECV should be obtained immediately before the MRI scan, if possible, otherwise within ± 3 days of scanning. If the MRI scan is not done on the same day as a scheduled visit (as outlined in the flowchart in Section 1.2), sites should use an unscheduled laboratory kit for collection of the blood sample for measurement of haematocrit.*	To specify further
8.2.3.2 Echocardiography	*To calibrate the echocardiogram, the imaging core laboratory may test the imaging protocol in healthy volunteers. This test will check the echocardiogram settings and the quality of the images generated. Novo Nordisk will not have access to any data generated from the echocardiographic examination on the healthy volunteers and these participants are therefore not considered part of the study.*	To specify further
8.3.6 Cardiac monitoring (only applicable for sentinel participants)	(Holter ECG) changed to (*e.g.* Holter ECG)	To accommodate that sites may have different systems for out-patient cardiac monitoring
8.3.8 Pregnancy testing	~~Woman~~ *Women* of childbearing potential (WOCBP) should only be included after a negative, highly sensitive ~~urine~~ pregnancy test (see Appendix 2 [Section 10.2]).<br><br>*Pregnancy testing should be performed at the timepoints outlined in the flowchart (Section 1.2), as specified in Appendix 2 (Section 10.2).*<br><br>Pregnancy testing should *also* be performed whenever a menstruation is missed or when pregnancy is otherwise suspected.	To align with flowchart and Appendix 2 (section 10.2)

Section # and name	Description of change	Brief rationale
8.5.1 Pharmacokinetics	eCRF changed to *laboratory requisition form*:<br><br>The exact timing (date and time) of obtaining the pharmacokinetic (PK) sample should be recorded ~~in~~ *on* the ~~eCRF~~ *laboratory requisition form*.	To specify further
8.8.1 Anti-NNC6019-0001-antibodies	~~serum~~ changed to *sample*:<br><br>For details on blood sampling, serum *sample* preparation and storage, please refer to the laboratory manual.	For correctness
8.8.3 Human biosamples for future research	Participation is optional, and participants must sign ~~a~~ separate informed consent *forms for future research and for genotyping* to indicate their participation in the biobank component(s) of the study. *Participants cannot take part in the genotyping biobank component only; thus, participants who provide informed consent for genotyping research must also provide informed consent for future research.*	For clarification
10.2 Appendix 2: Clinical laboratory tests	Footnote 'b' to Table 10-1 updated:<br><br>[b] misTTR or fragments thereof depending on assay availability and the analysis of misTTR will be performed by Novo Nordisk ~~A/S~~ *or a special laboratory contracted by Novo Nordisk*	To specify further
10.8 Appendix 8: Country-specific requirements - France	*Appendix 1 (Section 10.1.1). Regulatory and Ethical Considerations: The inclusion of people at risk is not allowed (persons under guardianship or Curatorship). Affiliation to a "sécurité sociale" regime is mandatory.*	To align with national requirements for France
10.8 Appendix 8: Country-specific requirements - Japan	*Section 6.1: The actual dose of diphenhydramine (or H1 antihistamine) and acetaminophen for premedication may be decided by the investigator in consultation with Novo Nordisk.*<br><br>*Section 6.1: For this study, drugs used in the clinical study except IMPs are diphenhydramine and acetaminophen. Please refer to local package insert for the latest information.*	For clarification and in alignment with GCP requirements in Japan
10.8 Appendix 8: Country-specific requirements – Netherlands	Sections 1.2 and 8.1: Date of birth: participant's full day of birth *can only be used if there is a strong need related to the hypothesis of the study. Generally, only* ~~is not allowed to be collected as part of demography and must be shortened to~~ year of birth *is to be used*.	For correctness to align with national requirements for Netherlands

**Section # and name**	**Description of change**	**Brief rationale**
10.10 Appendix 10: Protocol amendment history	New protocol section added for protocol amendment history	For completeness
11 References	Reference #1 added and references renumbered accordingly throughout the document:<br><br>*1. The European Parliament and the Council of the European Council. Directive 2001/20/EC of the European Parliament and of the Council of 4 April 2001 on the approximation of the laws, regulations and administrative provisions of the member states relating to the implementation of good clinical practice in the conduct of clinical trials on medicinal products for human use. 2001.*	For completeness

Protocol version 2.0:13 July 2022, for Czech Republic

This amendment is considered to be non-substantial based on the criteria set forth in Article 10(a) of Directive 2001/20/EC of the European Parliament and the Council of the European Union,[2] because it neither significantly impacts the safety nor physical/mental integrity of subjects nor the scientific value of the trial.

Overall rationale for preparing protocol, version 2.0:

The overall rationale for the changes implemented in the amended protocol is to address comments received from the Health Authority in Czech Republic.

Deleted text is written as ~~strikethrough~~ and new text *italic*.

6.1 Study interventions administered	Footnotes to Table 6-2 updated:<br><br>***Note**: Czech Republic and Japan: For country specific requirements, please refer to Appendix 8 (Section 10.8).*<br>[a]Also applicable for the first 6 randomised Japanese participants~~; Japan: For country specific requirements, please refer to Appendix 8 (Section 10.8)~~.	Additional safety monitoring requested by Health Authority in Czech Republic
10.8 Appendix 8: Country-specific requirements – Czech Republic	New bullet added:<br><br>• *Section 6.1 All participants in the Czech Republic must be monitored at the study site for at least 2 hours (+15 min) after infusion of the 2nd and 3rd doses of trial product. Vital signs to be measured at 10 min (±1 min), 60 min (±10 min) and 2 hours (+15 min) after infusion. From 4th dose and on, all participants must be monitored for at least 90 min (±10 min) after infusion.*	Additional safety monitoring requested by Health Authority in Czech Republic
10.8 Appendix 8: Country-specific requirements – Czech Republic	New bullet added:<br><br>• *Section 8.3.8: For women of childbearing potential, a pregnancy test (preferably a highly sensitive urine hCG pregnancy test) must be performed at every visit before administration of trial product.*	Clarification on pregnancy testing requested by Health Authority in Czech Republic

11 References

1. The European Parliament and the Council of the European Council of the European Union. Regulation (EU) No 536/2014 of the European Parliament and of the Council of 16 April 2014 on clinical trials on medicinal products for human use, and repealing Directive 2001/20/EC. 27 May 2014.
2. The European Parliament and the Council of the European Council. Directive 2001/20/EC of the European Parliament and of the Council of 4 April 2001 on the approximation of the laws, regulations and administrative provisions of the member states relating to the implementation of good clinical practice in the conduct of clinical trials on medicinal products for human use. 2001.
3. Ruberg FL, Grogan M, Hanna M, Kelly JW, Maurer MS. Transthyretin Amyloid Cardiomyopathy: JACC State-of-the-Art Review. J Am Coll Cardiol. 2019;73(22):2872-91.
4. Spertus JA, Jones PG, Sandhu AT, Arnold SV. Interpreting the Kansas City Cardiomyopathy Questionnaire in Clinical Trials and Clinical Care: JACC State-of-the-Art Review. J Am Coll Cardiol. 2020;76(20):2379-90.
5. Hawkins PN, Ando Y, Dispenzeri A, Gonzalez-Duarte A, Adams D, Suhr OB. Evolving landscape in the management of transthyretin amyloidosis. Ann Med. 2015;47(8):625-38.
6. Emdin M, Aimo A, Rapezzi C, Fontana M, Perfetto F, Seferović PM, et al. Treatment of cardiac transthyretin amyloidosis: an update. Eur Heart J. 2019;40(45):3699-706.
7. Garcia-Pavia P, Rapezzi C, Adler Y, Arad M, Basso C, Brucato A, et al. Diagnosis and treatment of cardiac amyloidosis: a position statement of the ESC Working Group on Myocardial and Pericardial Diseases. Eur Heart J. 2021;42(16):1554-68.
8. Lauppe RE, Liseth Hansen J, Gerdesköld C, Rozenbaum MH, Strand AM, Vakevainen M, et al. Nationwide prevalence and characteristics of transthyretin amyloid cardiomyopathy in Sweden. Open Heart. 2021;8(2).
9. Damy T, Bourel G, Slama M, de Neuville B, Rault C, Charrong P. PCV67 Epidemiology of Transthyretin Amyloid Cardiomyopathy (ATTR-CM) in France: EPACT, a Study Based on the French Nationwide Claims Database Snds. Value in Health. 2020;23:S498-9.
10. Winburn I, Ishii T, Sumikawa T, Togo K, Yasunaga H. Estimating the Prevalence of Transthyretin Amyloid Cardiomyopathy in a Large In-Hospital Database in Japan. Cardiol Ther. 2019;8(2):297-316.
11. Auer-Grumbach M, Rettl R, Ablasser K, Agis H, Beetz C, Duca F, et al. Hereditary ATTR Amyloidosis in Austria: Prevalence and Epidemiological Hot Spots. J Clin Med. 2020;9(7).
12. Lindmark K, Pilebro B, Sundström T, Lindqvist P. Prevalence of wild type transtyrethin cardiac amyloidosis in a heart failure clinic. ESC Heart Fail. 2021;8(1):745-9.

13. Lane T, Fontana M, Martinez-Naharro A, Quarta CC, Whelan CJ, Petrie A, et al. Natural History, Quality of Life, and Outcome in Cardiac Transthyretin Amyloidosis. Circulation. 2019;140(1):16-26.
14. Maurer MS, Bokhari S, Damy T, Dorbala S, Drachman BM, Fontana M, et al. Expert Consensus Recommendations for the Suspicion and Diagnosis of Transthyretin Cardiac Amyloidosis. Circ Heart Fail. 2019;12(9):e006075.
15. Yamamoto H, Yokochi T. Transthyretin cardiac amyloidosis: an update on diagnosis and treatment. ESC Heart Fail. 2019;6(6):1128-39.
16. Pfizer. Vyndaqel ® (tafamidis), EU Summary of product characteristics (SmPC). 2019.
17. Pfizer. Vyndaqel® (tafamidis), US Prescribing Information (PI). June 2021.
18. Ionis Pharmaceutical. Tegsedi™(inotersen), EU summary of product characteristics (SmPC). 2018.
19. Ionis Pharmaceuticals. Tegsedi™ (inotersen), US prescribing information (PI). Oct 2018.
20. Alnylam Pharmaceuticals Inc. Onpattro™ (patisiran), EU Summary of product characteristics (SmPC). 2018.
21. Alnylam Pharmaceuticals Inc. Onpattro™ (patisiran), US prescribing information (PI). Aug 2018.
22. Maurer MS, Schwartz JH, Gundapaneni B, Elliott PM, Merlini G, Waddington-Cruz M, et al. Tafamidis Treatment for Patients with Transthyretin Amyloid Cardiomyopathy. N Engl J Med. 2018;379(11):1007-16.
23. Prothena Biosciences Limited. Clinical trial report (PRX004-101). A phase 1, open-label, dose escalation study of intravenous PRX004 in subjects with amyloid transthyretin (ATTR) amyloidosis. 18 March 2021.
24. Kittleson MM, Ruberg FL, Ambardekar AV, Brannagan TH, Cheng RK, Clarke JO, et al. 2023 ACC Expert Consensus Decision Pathway on Comprehensive Multidisciplinary Care for the Patient With Cardiac Amyloidosis: A Report of the American College of Cardiology Solution Set Oversight Committee. J Am Coll Cardiol. 2023;81(11):1076-126.
25. Benson MD, Buxbaum JN, Eisenberg DS, Merlini G, Saraiva MJM, Sekijima Y, et al. Amyloid nomenclature 2020: update and recommendations by the International Society of Amyloidosis (ISA) nomenclature committee. Amyloid. 2020;27(4):217-22.
26. Merlini G, Bellotti V. Molecular mechanisms of amyloidosis. N Engl J Med. 2003;349(6):583-96.
27. Benson MD, Kincaid JC. The molecular biology and clinical features of amyloid neuropathy. Muscle Nerve. 2007;36(4):411-23.
28. Coelho T, Maurer MS, Suhr OB. THAOS - The Transthyretin Amyloidosis Outcomes Survey: initial report on clinical manifestations in patients with hereditary and wild-type transthyretin amyloidosis. Curr Med Res Opin. 2013;29(1):63-76.

29. Lobato L. Portuguese-type amyloidosis (transthyretin amyloidosis, ATTR V30M). J Nephrol. 2003;16(3):438-42.
30. Lobato L, Rocha A. Transthyretin amyloidosis and the kidney. Clin J Am Soc Nephrol. 2012;7(8):1337-46.
31. Hofmann C, Katus HA, Doroudgar S. Protein Misfolding in Cardiac Disease. Circulation. 2019;139(18):2085-8.
32. Novo Nordisk A/S. Investigator's Brochure, NNC6019-0001, project NN6019, (edition 4). 31 Jan 2022.
33. European Medicines Agency. ICH E9 (R1) addendum on estimands and sensitivity analysis in clinical trials to the guideline on statistical principles for clinical trials. Step 5 (EMA/CHMP/ICH/436221/2017). 17 Feb 2020.
34. American College of Radiology. Nephrogenic systemic fibrosis. ACR Manual On Contrast Media: ACR Committee on Drugs and Contrast Media; 2021. p. 80-8.
35. Oghina S, Josse C, Bézard M, Kharoubi M, Delbarre MA, Eyharts D, et al. Prognostic Value of N-Terminal Pro-Brain Natriuretic Peptide and High-Sensitivity Troponin T Levels in the Natural History of Transthyretin Amyloid Cardiomyopathy and Their Evolution after Tafamidis Treatment. J Clin Med. 2021;10(21).
36. Uszko-Lencer NHMK, Mesquita R, Janssen E, Werter C, Brunner-La Rocca HP, Pitta F, et al. Reliability, construct validity and determinants of 6-minute walk test performance in patients with chronic heart failure. Int J Cardiol. 2017;240:285-90.
37. Law S, Petrie A, Chacko L, Cohen OC, Ravichandran S, Gilbertson JA, et al. Change in N-terminal pro-B-type natriuretic peptide at 1 year predicts mortality in wild-type transthyretin amyloid cardiomyopathy. Heart. 2021.
38. Fontana M, Ćorović A, Scully P, Moon JC. Myocardial Amyloidosis: The Exemplar Interstitial Disease. JACC Cardiovasc Imaging. 2019;12(11 Pt 2):2345-56.
39. Prothena Biosciences. PK/PD report (PRX001-101): A phase 1, open-label, dose escalation study of intravenous PRX004 in subjects with amyloid transthyretin (ATTR) amyloidosis: Pharmacokinetic, phamacodynamic, immunogenicity report. 13 Jan 2021.
40. Shah DK, Betts AM. Antibody biodistribution coefficients: inferring tissue concentrations of monoclonal antibodies based on the plasma concentrations in several preclinical species and human. MAbs. 2013;5(2):297-305.
41. European Commission. The rules governing medicinal products in the European Union. Volume 4. EU guidelines to Good Manufacturing Practice (GMP). Annex 13: Investigational medicinal products (ENTR/F/2/AM/an D(2010) 3374). 03 Feb 2010.
42. Hanna M, Ruberg FL, Maurer MS, Dispenzieri A, Dorbala S, Falk RH, et al. Cardiac Scintigraphy With Technetium-99m-Labeled Bone-Seeking Tracers

for Suspected Amyloidosis: JACC Review Topic of the Week. J Am Coll Cardiol. 2020;75(22):2851-62.

43. Arvanitis M, Koch CM, Chan GG, Torres-Arancivia C, LaValley MP, Jacobson DR, et al. Identification of Transthyretin Cardiac Amyloidosis Using Serum Retinol-Binding Protein 4 and a Clinical Prediction Model. JAMA Cardiol. 2017;2(3):305-13.
44. Jiang X, Labaudinière R, Buxbaum JN, Monteiro C, Novais M, Coelho T, et al. A circulating, disease-specific, mechanism-linked biomarker for ATTR polyneuropathy diagnosis and response to therapy prediction. Proc Natl Acad Sci U S A. 2021;118(9).
45. Solomon SD, Adams D, Kristen A, Grogan M, González-Duarte A, Maurer MS, et al. Effects of Patisiran, an RNA Interference Therapeutic, on Cardiac Parameters in Patients With Hereditary Transthyretin-Mediated Amyloidosis. Circulation. 2019;139(4):431-43.
46. World Medical Association. WMA Declaration of Helsinki - Ethical Principles for Medical Research Involving Human Subjects. Last amended by the 64th WMA General Assembly, Fortaleza, Brazil. Oct 2013.
47. ICH Harmonised Tripartite Guideline. Guideline for Good Clinical Practice E6(R2), Current step 4 version. 09 Nov 2016.
48. De Angelis C, Drazen JM, Frizelle FA, Haug C, Hoey J, Horton R, et al. Clinical trial registration: a statement from the International Committee of Medical Journal Editors. N Engl J Med. 2004;351(12):1250-1.
49. Food and Drug Administration. Food and Drug Administration Amendments Act of 2007 as amended by the Final Rule "Clinical Trials Registration and Results Information Submission". 21 Sep 2016.
50. The European Parliament and the Council of the European Council. Regulation (EC) No 726/2004 of the European Parliament and of the Council of 31 March 2004 laying down Community procedures for the authorisation and supervision of medicinal products for human and veterinary use and establishing a European Medicines Agency, article 57. 30 April 2004.
51. The European Parliament and the Council of the European Council. Regulation (EC) No 1901/2006 of the European Parliament and of the Council of 12 December 2006 on medicinal products for paediatric use and amending Regulation (EEC) No 1768/92, Directive 2001/20/EC, Directive 2001/83/EC and Regulation (EC) No 726/2004, article 41. Official Journal of the European Communities. 27 Dec 2006.
52. International Committee of Medical Journal Editors. Recommendations for the Conduct, Reporting, Editing and Publication of Scholarly Work in Medical Journals; current version available at www.icmje.org.
53. Clinical Trial Facilitation Group (CTFG), Heads of Medicines Agency. Recommendations related to contraception and pregnancy testing in clinical trials. 21 Sep 2020.

54. U.S. Department of Health and Human Services, Food and Drug Administration. Guidance for Industry: Immunogenicity Assessment for Therapeutic Protein Products. August 2015.

Statistical Analysis Plan

Efficacy and safety of NNC6019-0001 at two dose levels in participants with transthyretin amyloid cardiomyopathy (ATTR CM)

Substance: NNC6019-0001

Redacted statistical analysis plan
Includes redaction of personal identifiable information only.

.

Author
[redacted]
Biostatistics Cagrisema & CKAD 5

Table of contents

Page

Table of contents 2

Table of figures 3

Table of tables 4

Version History 5

List of abbreviations 6

1. Introduction 8
1.1 Objectives, endpoints, and estimands 8
1.1.1 Objectives and endpoints 8
1.1.2 Estimands 9
1.2 Study design 11

2. Statistical hypotheses 12
2.1 Multiplicity adjustment 12

3. Analysis sets 13

4. Statistical Analyses 15
4.1 General Considerations 15
4.2 Primary endpoints analysis 15
4.2.1 Definition of endpoints 15
4.2.2 Main analytical approach 15
4.2.3 Sensitivity analysis 17
4.2.4 Supplementary analysis 17
4.3 Secondary endpoints analysis 17
4.3.1 Supportive secondary endpoints 17
4.3.1.1 Supportive secondary efficacy endpoints 17
4.3.1.2 Supportive secondary safety endpoints 18
4.4 Exploratory endpoints analysis 20
4.5 Other Safety Analysis 21
4.6 Other analyses 21
4.6.1 Other derivations and assessments 21
4.6.1.1 Statistical analyses of other assessments at week 24 21
4.6.1.2 Imaging parameters based on imaging charter 21
4.6.1.3 Other assessments 21
4.6.2 Subgroup analyses 22
4.7 Interim Analysis 22
4.8 Data Monitoring Committee (DMC) 22
4.9 Changes to protocol-planned Analysis 22

5. Sample size determination 23

6. Supporting documentation 24

Appendix A : Definition and calculation of endpoints, assessments and derivations 24

Appendix B : Neuropathy impairment score (NIS) and domains 26

7. References 32

Table of figures

Page

Figure 1 Study design 11

Table of tables

Page

Table 1 Objectives and endpoints 8

Table 2 Exploratory endpoints 9

Table 3 Handling of premature discontinuation of randomised study intervention and missing values for various reasons 11

Table 4 NIS questionnaire 26

Version History

This Statistical Analysis Plan (SAP) for study NN6019-4940 is based on the protocol version 6.0 dated 07FEB2024.

**SAP Version**	**Date**	**Change**	**Rationale**
1.0	14-Aug-2024	Not Applicable	Original version

List of abbreviations

6MWT	6 Minute Walk Test
AE	Adverse event
ATTR	Transthyretin amyloid
CRF	Case report form
CSR	Clinical study report
CSS	Clinical summary score
CV	Cardiovascular
CI	Confidence interval
CM	Cardiomyopathy
DBL	Database lock
DMC	Data monitoring committee
EAC	Event Adjudication Committee
ECV	Extracellular volume
eCRF	Electronic case report form
EQ-5D-5L	EuroQoL five dimensions five level
FAS	Full analysis set
FDA	U.S. Food and Drug Administration
GLS	Global longitudinal strain
hATTR	Hereditary ATTR
HF	Heart failure
ICH	International council of harmonisation
KCCQ	Kansas City Cardiomyopathy Questionnaire
MRI	Magnetic resonance imaging
mis-TTR	Misfolded transthyretin
NIS	Neuropathy impairment score

NT-proBNP	N-terminal-pro brain natriuretic peptide
NYHA	New York Heart Association
OSS	Overall summary score
PK	Pharmacokinetics
PGI-S	Patient global impression status
PGI-C	Patient global impression of change
PN	Polyneuropathy
PYE	Participant years of exposure
PYO	Participant years of observation
Q4W	Every 4 Weeks
RTSM/IWRS	Randomisation and trial supplies management system
SAP	Statistical analysis plan
SAE	Serious adverse event
SC	Subcutaneous
TEAE	Treatment emergent adverse event
TTR	Transthyretin
VAS	Visual Analogue scale
wATTR	Wild type ATTR

1. Introduction

This SAP is based on the protocol: Efficacy and safety of NNC6019-0001 at two dose levels in participants with transthyretin amyloid cardiomyopathy (ATTR CM). For primary endpoints statistical analyses and derivations of endpoints presented in this SAP are identical to those described in the protocol, but some additional details have been added. SAP also contains specification of other derivations and analyses. The SAP also contains specifications of additional derivations and analyses in Appendix A, section 6.

1.1 Objectives, endpoints, and estimands

1.1.1 Objectives and endpoints

Table 1 Objectives and endpoints

**Objective**	**Endpoints**		
**Primary**	**Title**	**Time frame**	**Unit**
• To compare the effect of two dose levels of NNC6019-0001 (10 mg/kg and 60 mg/kg) versus placebo on:<br>• change in 6-minute walk test and<br>• change in NT-proBNP<br>from baseline to week 52 in participants with hATTR or wtATTR cardiomyopathy.	Primary		
	Change in 6-minute walk test (6MWT)	From baseline (week 0) to visit 15 (week 52)	Meters
	Change in NT-proBNP	From baseline (week 0) to visit 15 (week 52)	Percentage
**Secondary**	**Title**	**Time frame**	**Unit**
• To compare the effect of two dose levels of NNC6019-0001 (10 mg/kg and 60 mg/kg) versus placebo on:<br>• biomarkers<br>• pharmacodynamic endpoints<br>from baseline to week 52 in participants with hATTR or wtATTR cardiomyopathy.	Supportive Secondary		
	Change in myocardial extracellular volume (ECV)	From baseline (week 0) to visit 15 (week 52)	%-points
	Change in Kansas City Cardiomyopathy Questionnaire (KCCQ) Clinical Summary Score[a](CSS)	From baseline (week 0) to visit 15 (week 52)	Score
	Change in neuropathy impairment score[b] (NIS)	From baseline (week 0) to visit 15 (week 52)	Score
	Change in troponin I	From baseline (week 0) to visit 15 (week 52)	ng/mL

	Change in global longitudinal strain (GLS) on echocardiography	From baseline (week 0) to visit 15 (week 52)	%-points
• To compare the effect of two dose levels of NNC6019-0001 (10 mg/kg and 60 mg/kg) versus placebo on:<br>• safety and tolerability<br>from baseline to week 64 in participants with hATTR or wtATTR cardiomyopathy.	Number of treatment emergent adverse events	From baseline (week 0) to visit 16 (week 64)	Count
	Time to occurrence of all-cause mortality	From baseline (week 0) to visit 16 (week 64)	Weeks
	Number of CV events comprising hospitalisation due to CV events or urgent heart failure visits	From baseline (week 0) to visit 16 (week 64)	Count

[a] Clinical Summary Score (CSS) consists of the Symptom domain and the Physical Limitation domain, additional details on scoring manual are described in "Subject Questionnaire KCCQ Scoring Guide". Scores range from 0 to 100 and lower scores represent more severe symptoms and/or limitations and scores of 100 indicate no symptoms, no limitations, and excellent quality of life; [b] Only applicable for participants with hATTR CM. The total NIS score is graded on a scale of 0–244, with a higher score indicating greater impairment.

Table 2 Exploratory endpoints

Objective	Title	Timeframe	Unit
Exploratory			
• To compare the effect of two dose levels of NNC6019-0001 (10 mg/kg and 60 mg/kg) versus placebo on depletion of plasma misTTR from baseline to week 52 in participants with hATTR or wtATTR cardiomyopathy.	Change in misfolded transthyretin[c] (misTTR)	From baseline (week 0) to visit 15 (week 52)	Percentage
• To compare the effect of two dose levels of NNC6019-0001 (10 mg/kg and 60 mg/kg) versus placebo on change in EQ-5D-5L from baseline to week 52 in participants with hATTR or wtATTR cardiomyopathy.	Change in EQ-5D-5L[d]	From baseline (week 0) to visit 15 (week 52)	Score

[c] Including fragmented TTR depending on assay development. [d] Additional details on scoring manual are described in "Subject Questionnaire EQ-5D-5L User Guide_v3.0"

1.1.2 Estimands

Primary estimand

The primary estimand addresses the following question of interest: What is the effect of two dose levels of NNC6019-0001 (10 mg/kg and 60 mg/kg) versus placebo on change in 6MWT and NT-

proBNP from baseline to week 52, or occurrence of death or CV hospitalisation, in participants with hATTR or wtATTR cardiomyopathy, regardless of premature discontinuation of study intervention.

The primary estimand is defined with the five attributes as defined in ICH E9(R1) addendum2

- Treatment condition: The treatment regimen evaluated is i.v. infusion of NNC6019-0001 Q4W at two dose levels (10 mg/kg and 60 mg/kg) vs placebo, irrespective of use of concomitant medications indicated for ATTR CM or ATTR PN (TTR silencers and stabilisers).
- Population: The treatment effect is assessed for the target population of patients with hATTR or wtATTR CM.
- Variable: The treatment effect is assessed by change in 6MWT and relative change in NT-proBNP from baseline to week 52.
- Remaining intercurrent events (see Table 3):
  Premature discontinuation of randomised study intervention will be handled by a treatment policy strategy including all post-discontinuation observations of 6MWT and NT-proBNP in the analysis and conditional multiple imputation from the placebo arm of missing values.
  - Missing values will be handled as follows:
    - Missing 6MWT values due to all-cause death or CV hospitalisation or urgent heart failure visit will be handled by a composite strategy assigning a single value of 0 meters
    - The risk of missing 6MWT due to fractures or fall-related injuries is assumed unrelated to treatment and missing values will be handled by a hypothetical strategy depending on whether the participant is on the randomised study intervention or has prematurely discontinued.
    - Missing 6MWT values for other than the above reasons will be handled by a hypothetical strategy depending on whether the participant is on the randomised study intervention or has prematurely discontinued.
    - Missing NT-proBNP values due to all-cause death or CV hospitalisation or urgent heart failure visit will be handled by a composite strategy assigning a single value corresponding to the highest observed NT-proBNP value across all participant and visits (including baseline).
    - Missing NT-proBNP values for other reasons will be handled by a hypothetical strategy depending on whether the participant is on the randomised study intervention or has prematurely discontinued.
- Population-level summary:
  - Difference in mean change in 6MWT from baseline between NNC6019-0001 (10 mg/kg or 60 mg/kg) vs placebo.
  - Difference in mean change in NT-proBNP (log-scale) from baseline between NNC6019-0001 (10 mg/kg or 60 mg/kg) vs placebo. The difference will be back transformed to original scale and reported as a ratio of geometric mean ratios.

Table 3 Handling of premature discontinuation of randomised study intervention and missing values for various reasons

		**Premature discontinuation of randomised study intervention**	
**Value**	Endpoint	No	Yes
**Observed**	6MWT<br>NT-proBNP	Use value	Use value
**Missing due to death or CV hospitalisation or urgent heart failure visit**	6MWT<br>NT-proBNP	Assign 0 meters<br>Assign highest observed	Assign 0 meters<br>Assign highest observed
**Missing due to fracture or other reason**	6MWT<br>NT-proBNP	Impute from own group	Impute from placebo group

Rationale for the estimand

The estimand for the two primary endpoints is chosen to best quantify a potential effect on the planned phase 3 primary endpoint considering both efficacy and tolerability. To penalise the phase 3 primary endpoint events as much as possible within sensible limits, it is chosen to assign a single worst-case value of 0 meters for 6MWT and the highest observed value of NT-proBNP, acknowledging that such values may not be directly interpretable.

1.2 Study design

This is an interventional, randomised, multinational, multicentre, three-arm parallel-group, double-blind, placebo-controlled study comparing i.v. NNC6019-0001 Q4W at two dose levels (10 mg/kg and 60 mg/kg) versus placebo in participants with hATTR or wtATTR CM.

For further details please refer protocol section 4.1.

Figure 1 Study design

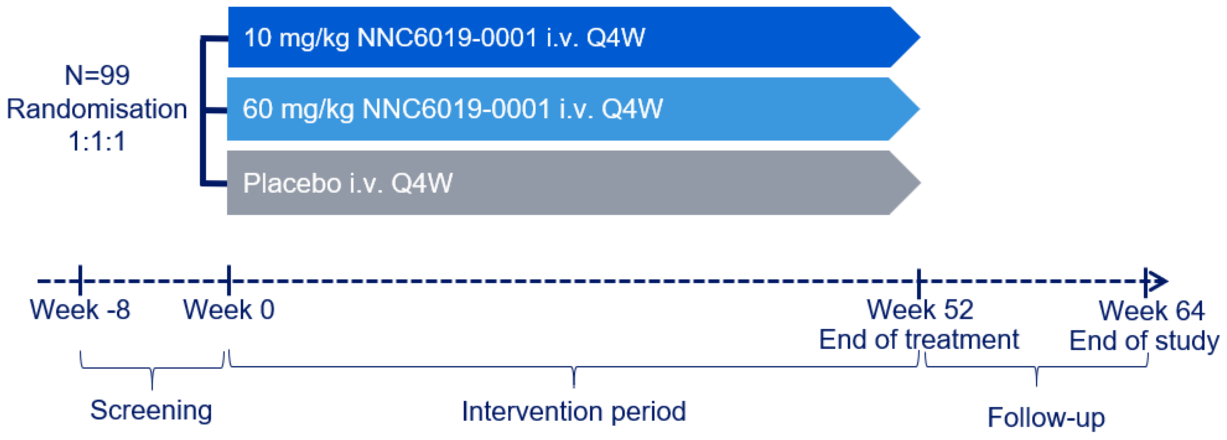

2. Statistical hypotheses

No confirmatory statistical hypothesis testing will be done in this study.

2.1 Multiplicity adjustment

As no confirmatory hypothesis will be tested statistically, no adjustment for multiplicity will be done for the primary endpoints.

3. Analysis sets

The following participant analysis sets are defined:

**Participant analysis set (PAS)**	**Description**
Full analysis set (FAS)	All randomised participants, except participants who initiate the randomisation registration in RTSM/IWRS but withdraw consent, is withdrawn by the investigator, or is lost to follow-up prior to first dosing administration. Participants will be included in the analyses according to the planned intervention.
Safety analysis set (SAS)	All participants who are exposed to study intervention. Participants will be included in the analyses according to the intervention they actually received.

In exceptional cases, participants or observations may be eliminated from the full analysis set. In such case the reasons for their exclusion will be documented before unblinding. The participants and observations excluded from analysis sets, and the reason for this, will be described in the clinical study report.

A review of all profile data will be performed before the DBL decision meeting. The profiles are reviewed by the trial statistician and the project PK team. The project PK team consists of representatives from Non-Clinical and Clinical Assay Sciences, Clinical Pharmacology, Pharmacometrics, Biostatistics. The responsibility of the project PK team is to ensure consistent communication of PK results throughout the project. The project PK team may invite additional members as per need.

The key purpose of the profile review is to agree on samples for re-analysis, data points to be excluded from analyses (if any). If special rules or data exclusions are needed for individual participants, profiles or endpoints it can be agreed at the profile review meetings and documented in DBL decision minutes.

A treatment completer is defined as a participant who will have taken the last dose at visit 14 without permanently discontinuing the trial product.

A study completer will be defined as a participant who will not have withdrawn from the study and will have attended the visit 16.

The following periods will be considered for the data collected:

In-study period : The in-study period is defined as the time interval from date of randomisation to

- Latest date of assessments collected at either the planned end-of-treatment, premature follow-up visit or the planned follow-up visit.
- Withdrawal date for participants who withdraw informed consent.
- Date of the last participant-investigator/site contact as defined by investigator for participants who are lost to follow up.
- Date of death for participants who died before any of the above.

On-treatment period : The on-treatment period starts at the date of first dose of trial product as recorded on the eCRF, and ends at the first date of any of the following :

- The last date on trial product + 112 days (The last administered trial product will be in week 48, and the follow-up is scheduled for week 64, which will correspond to 16 weeks after the last administration of the trial product).
- Premature follow-up date in case of premature treatment discontinuation.
- Planned follow-up date.
- The end-date for the in-study period.

The in-study and on-treatment periods define the participant years of observation (PYO) and participant years of exposure (PYE) respectively, as the total time duration in the periods.

Full analysis set and in-study period will be used for efficacy evaluations whereas safety analysis set and on-treatment period will be used for safety evaluations, unless otherwise specified.

Treatment Emergent Adverse events (TEAE) are defined as events that have onset during the on-treatment period.

4. Statistical Analyses

4.1 General Considerations

Presentation of results from a statistical analysis will include the estimated mean treatment difference (or ratio) presented together with the two-sided 95% confidence interval and the corresponding two-sided p-value.

The numbers 601949401 and 601949402 will be utilized as the seeds for imputations in the exploratory analysis of week 24 and primary analysis of week 52, respectively with regard to the primary endpoints.

Baseline value is defined as latest available assessment at or prior to randomisation. If more than one measurement is associated with the same visit, the earliest measurement is considered eligible. For Vital signs (Pulse, Diastolic and Systolic blood pressure), the baseline assessment will be defined as the mean of the assessments from the randomization visit (V2) observed at the timepoint "Before infusion".

4.2 Primary endpoints analysis

4.2.1 Definition of endpoints

The primary endpoints are change in 6MWT and change in NT-proBNP from baseline to week 52.

4.2.2 Main analytical approach

Analysis addressing the primary estimand

The effect of interest in the primary estimand is regardless of premature discontinuation of study intervention. The primary analysis will be based on the FAS. The following statistical analysis and imputation method is used to address the primary estimand.

Discontinuation of randomised study intervention will be handled by a treatment policy strategy including all post-discontinuation observations. Missing values of 6MWT and NT-proBNP (log-transformed) will be imputed (single or multiple) as described in the estimand section (Table 3). Subsequently, values of change from baseline to week 52 will be calculated based on the observed and imputed post-baseline values. The procedure is described in detail below:

First missing values due to death or CV hospitalisation or urgent heart failure visit will be assigned according to Table 3.

Thereafter, missing values due to fracture or other reason (Table 3) of 6MWT or NT-proBNP (log-transformed) will be multiple imputed sequentially:

- First, intermittent missing post-baseline values are imputed separately for each intervention group using Markov Chain Monte Carlo to generate multiple (1000) copies of the dataset with monotone missing data patterns. If an unexpectedly high amount of missing data is observed, the number of imputations will be increased to 10000.

- Next, a stepwise procedure sequentially imputes the missing values for the remaining visits containing missing values.

- At the first visit containing missing values, models are fitted for each copy of the dataset to the observed values for:

    - Pattern 1: Placebo group

    - Pattern 2: NNC6019-0001 10 mg/kg on randomised study intervention

    - Pattern 3: NNC6019-0001 60 mg/kg on randomised study intervention

The models will include the stratification variable as a factor and as covariates baseline 6MWT or NT-proBNP (log-transformed) and the observed post-baseline assessments for visits prior to the one in question. The estimated parameters, and their variances, are used to impute missing post-baseline values for the visit in question. For 6MWT, a minimum of zero will be specified to prevent imputations below zero.

- Placebo group: Impute from pattern 1

- NNC6019-0001 10 mg/kg group and prematurely discontinued randomised study intervention: Impute from pattern 1

- NNC6019-0001 60 mg/kg group and prematurely discontinued randomised study intervention: Impute from pattern 1

- NNC6019-0001 10 mg/kg on randomised study intervention: Impute from pattern 2

- NNC6019-0001 60 mg/kg on randomised study intervention: Impute from pattern 3

In the above step where imputations will be performed based on the specified patterns, participants who have observations at visit 15 (week 52) but had permanently discontinued treatment before visit 15 (week 52) should be excluded from the imputation process.

The stepwise procedure is repeated sequentially for Visits 3, Visit 5, Visit 8, and Visit 15 to impute the missing values. If no intermittent missing values exist, multiple copies of the dataset will be generated at the first visit where missing values are present.

Values of change from baseline to Visit 15 (week 52) will be calculated based on the observed and imputed post-baseline values.

- For each of the complete data sets, change in 6MWT or NT-proBNP (log-transformed) from baseline to Visit 15 (week 52) is analysed using an analysis of variance model with randomised study intervention (NNC6019-0001 (10 mg/kg or 60 mg/kg) vs placebo) and the stratification variable as factors and baseline 6MWT or NT-proBNP (log-transformed) as a covariate. In case the amount of data for the described model is insufficient for meaningful interpretation or if convergence issues arise, then the model will be simplified by removing the stratification factor.

- The estimates and standard deviations for the dataset copies are pooled to one estimate and associated standard deviation using Rubin's rule. For NT-proBNP, the mean difference on the logarithmic scale will be back-transformed to original scale and reported as a ratio of geometric mean ratios.

4.2.3 Sensitivity analysis

As no confirmatory hypothesis will be tested statistically, no sensitivity analysis is planned for this study.

4.2.4 Supplementary analysis

No supplementary analyses have been planned.

4.3 Secondary endpoints analysis

4.3.1 Supportive secondary endpoints

4.3.1.1 Supportive secondary efficacy endpoints

The following endpoints are defined to investigate the efficacy of two dose levels of NNC6019-0001 (10mg/kg and 60 mg/kg) versus placebo.

- Change in myocardial extracellular volume (ECV)
- Change in Kansas City Cardiomyopathy Questionnaire (KCCQ) Clinical Summary Score (CSS)
- Change in neuropathy impairment score (NIS)
- Change in troponin I
- Change in global longitudinal strain (GLS) on echocardiography

Additional details on derivation of KCCQ-CSS is available in KCCQ scoring manual which can be referred from "Subject Questionnaire KCCQ Scoring Guide".

All the aforementioned endpoints related to change will be derived from the baseline to visit 15 (week 52) time frame.

The automated ECV i.e. 4 Chamber ECV derived from ECV mapping in Circle will be considered as the secondary endpoint and referred as myocardial extracellular volume (ECV).

The secondary efficacy endpoints (Change in ECV, KCCQ, GLS) will be analysed by using a model similar to primary analysis of 6MWT based on observed data (i.e. without imputations) with stratification variable as a factor and the corresponding baseline value as covariate.

Change in Troponin I (log-transformed) from baseline to visit 15 (week 52) will be analysed by using a model similar to primary analysis of NT-proBNP based on observed data (i.e. without imputations) with stratification variable as a factor and the corresponding baseline value (log-transformed) as covariate.

If the data for the aforesaid analysis is deemed insufficient for convergence or interpretation, the initial step will involve simplifying the model by removing the stratification factor. If issues persist despite these adjustments, the analysis will not be carried out due to limited data.

Change in neuropathy impairment score (NIS) will be summarised descriptively. Further details regarding the definitions of the NIS score are provided in Appendix B

4.3.1.2 Supportive secondary safety endpoints

The following endpoints are defined to investigate the safety of two dose levels of NNC6019-0001 (10mg/kg and 60 mg/kg) versus placebo.

- Number of treatment emergent adverse events
- Time to occurrence of all-cause mortality
- Number of CV events comprising hospitalisation due to CV events or urgent heart failure visits

Supportive secondary safety endpoints related to number of treatment emergent adverse events will be summarised descriptively based on data from on-treatment period and safety analysis set.

Time-to-event endpoints, censoring and competing risks :

In this study, we will have two supportive secondary time-to-event endpoints, "Time to occurrence of all-cause mortality" and "Number of cardiovascular events comprising hospitalization due to CV events or urgent heart failure visits". Both the endpoints will be analysed based on EAC confirmed events. Here, if a participant experiences the event of interest during the in-study period, the observation of the time to event is the time from randomisation to EAC-onset date of event. The observation of the time to event is censored if the event of interest does not happen during the in-study period or if the participant is still alive at the end of the observation period. The general assumption for censored observations is that the risk of experiencing an event is not changed by censoring, i.e. an assumption of independent censoring. This is a reasonable assumption for administrative censoring at end-of-study visit.

Time from randomisation to all-cause mortality will be analysed based on in-study period and full analysis set, using a cox proportional hazards model with stratification variable as a factor and treatment as covariate under the assumption of independent censoring. Participants without an event will be censored at either end of study visit (week 64) or participant discontinuation/withdrawal from the study whichever comes first. Ties are handled using the exact method and confidence intervals are based on the Wald test. Furthermore, time from randomisation to EAC-confirmed all-cause death, cumulative incidence plot will be produced with cumulative incidence estimates derived from the Aalen-Johansen estimator. If the data for the aforesaid analysis is deemed insufficient for convergence or interpretation, the initial step will involve simplifying the model by removing the stratification factor. If issues persist despite these adjustments, the analysis will not be carried out due to limited data.

Time from randomisation to EAC-confirmed CV events comprising of cardiovascular hospitalisation or urgent heart failure visits will be analysed based on in-study period and full analysis set, using the Anderson-Gill model with robust standard errors with stratification variable and treatment as factors. Participants without an event will be censored at either end of study visit (week 64) or participant discontinuation/withdrawal from the study or all-cause mortality whichever comes first. Ties are handled using the exact method and confidence intervals are based on the Wald test. If the data for the aforesaid analysis is deemed insufficient for convergence or interpretation, the initial step will involve simplifying the model by removing the stratification factor. If issues persist despite these adjustments, the analysis will not be carried out due to limited data. Further, mean number of EAC-confirmed CV events comprising of cardiovascular

hospitalisation or urgent heart failure visit per participant will be plotted based on in-study period and full analysis set, accounting for all-cause mortality as competing risk. Here cumulative mean number of events are estimated using the Ghosh-Lin estimator[34] of the cumulative mean function for each treatment group.

Dealing with tie scenarios for recurrent events analysis :

For recurrent events analysis of EAC-confirmed CV events comprising of cardiovascular hospitalisation or urgent heart failure we have implemented below specific rules to address tie scenarios, i.e. events occurring on the same date.

If a participant has more than one event then ties will be broken by adding a certain fraction to the relative study day of the next event onwards which are on the same date. This fraction is determined by the number of EAC confirmed events on the same study day.

Also among the events occurring on the same day there is a ranking rule :

- If non fatal EAC-confirmed event(s) occur on the same day as a CV death then the ‘Urgent HF visit without requiring hospitalisation’ should rank highest (among the non fatal EAC-confirmed events(s)) and fatal event should rank lowest. All events will be counted.
- If an ‘Urgent HF visit without requiring hospitalisation’ occurs on same day as any non-fatal EAC-confirmed event, then the ‘Urgent HF visit without requiring hospitalisation’ should rank highest (happened first). All events will be counted.

The below examples illustrate certain scenarios with respect to the rules applied.

In below 3 scenarios, “Start day” and “End day” refers to study day in particular according to the event occurrence and EAC-onset date. “Event” refers to type of EAC-confirmed event. “Order” refers to the sequence in which the events are considered to have occurred on the same day. "Modified start day" and "Modified end day" refer to the adjusted study day that take into account the rules for handling ties. These modified start and end day will be utilized in the statistical analyses and plots.

Scenario 1 :

If a participant had 3 confirmed events in same day.

Start day	End day	Event	Order	Modified start day	Modified end day
0	100	Urgent heart failure	1	0	100
0	100	CV hospitalisation	2	100	100.33
0	100	All-cause death	3	100.33	100.66

As Urgent heart failure event is ranked first, hence the new start and end day is same as 0 to 100.

But as 3 events have same start and end day, 1/3 i.e. 0.33 added to new end day, and so on. The modified start and end day

Scenario 2 :

If a participant had 2 confirmed events in same day.

Start day	End day	Event	Order	Modified start day	Modified end day
0	100	Urgent heart failure	1	0	100
0	100	CV hospitalisation	2	100	100.5

Scenario 3 :

If a participant had 2 confirmed events in same day.

Start day	End day	Event	Order	Modified start day	Modified end day
0	100	Urgent heart failure or CV hospitalisation	1	0	100
0	100	CV hospitalisation	2	100	100.5

4.4 Exploratory endpoints analysis

EQ-5D-5L endpoint refers to both EQ-5D-5L- EQ VAS Score and EQ-5D index score.

All exploratory endpoints will be presented descriptively.

If the assay for misTTR cannot be validated no summary statistics will be provided and the endpoint will not be analysed

Additional details on scoring manual of EQ-5D-5L can be referred from “Subject Questionnaire EQ-5D-5L User Guide_v3.0”.

4.5 Other Safety Analysis

All safety analyses will be made on the safety analysis set. The standard safety assessments (SAEs, AEs, safety laboratory parameters, vital signs, etc.) will be reported descriptively based on the on-treatment period; including any notable changes of clinical interest in laboratory parameters.

4.6 Other analyses

4.6.1 Other derivations and assessments

4.6.1.1 Statistical analyses of other assessments at week 24

Other statistical analyses are performed for the following assessments based on in-study period and full analysis set, by using a model similar to respective primary analysis with same imputation strategy but using only data until week 24.

- Change in 6MWT from baseline to week 24
- Change in NT-proBNP from baseline to week 24

Other statistical analyses are performed for the following assessments based on in-study period and full analysis set, by using a model similar to the respective secondary endpoints analyses based on observed data (without imputation).

- Change in myocardial extracellular volume (ECV)
- Change in Kansas City Cardiomyopathy Questionnaire (KCCQ) Clinical Summary Score (CSS)
- Change in global longitudinal strain (GLS) on echocardiography

Change in Troponin I (log-transformed) from baseline to visit 8 (week 24) will be analysed by using a model similar to secondary endpoint analysis of Troponin I based on observed data (i.e. without imputation) with stratification variable as a factor and the corresponding baseline value (log-transformed) as covariate.

4.6.1.2 Imaging parameters based on imaging charter

Relevant imaging parameters of Echocardiography and Cardiac MRI will be summarised by visits for in-study period and full analysis set based on Imaging charter.

4.6.1.3 Other assessments

The other responder assessments
- Participants improving at least 5, 10 or 15 points in KCCQ clinical summary score (Yes/No)
- Participants worsening at least 5, 10 or 15 points in KCCQ clinical summary score (Yes/No)
- Participants improving at least 5, 10 or 15 points in KCCQ overall summary score (Yes/No)
- Participants worsening at least 5, 10 or 15 points in KCCQ overall summary score (Yes/No)

will be summarised descriptively by visit and treatment groups.

Change in KCCQ overall summary score from baseline to visit 15 (week 52) will be summarised descriptively by visit and treatment groups. Additional details on derivation of overall summary score is available in KCCQ scoring manual which can be referred from “Subject Questionnaire KCCQ Scoring Guide”.

Patient global impression status (PGI-S) and Patient global impression of change (PGI-C) for KCCQ and 6MWT, will be summarised descriptive as per counts based on the categories by visit and treatment groups.

NYHA classification and shifts from one class to another in NYHA classification from baseline to week 24 and week 52 will be summarised by visit and treatment groups.

EAC-confirmed cardiovascular hospitalisation or urgent heart failure visits will be descriptively summarised, including number of participants, events, and rate per 100 participant years of observation along with cause and classification according to EAC-charter.

4.6.2 Subgroup analyses

No subgroup analyses are planned to be reported in the clinical study report.

4.7 Interim Analysis

Refer to protocol section 9.4

4.8 Data Monitoring Committee (DMC)

Refer to protocol section 10.1.6.2

4.9 Changes to protocol-planned Analysis

No changes to protocol-planned analyses have been added.

5. Sample size determination

Please see the protocol section 9.5.

6. Supporting documentation

Appendix A : Definition and calculation of endpoints, assessments and derivations

Type	Title	Time frame	Unit	Details
Primary endpoint	Change in 6-minute walk test (6MWT)	From baseline (week 0) to visit 15 (week 52)	Meters	
Primary endpoint	Change in NT-proBNP	From baseline (week 0) to visit 15 (week 52)	Percentage	Log transformed
Supportive secondary efficacy endpoint	Change in myocardial extracellular volume (ECV)	From baseline (week 0) to visit 15 (week 52)	%-points	
Supportive secondary efficacy endpoint	Change in Kansas City Cardiomyopathy Questionnaire (KCCQ) Clinical Summary Score (CSS)	From baseline (week 0) to visit 15 (week 52)	Score	
Supportive secondary efficacy endpoint	Change in neuropathy impairment score (NIS)	From baseline (week 0) to visit 15 (week 52)	Score	
Supportive secondary efficacy endpoint	Change in troponin I	From baseline (week 0) to visit 15 (week 52)	ng/mL	Log transformed
Supportive secondary efficacy endpoint	Change in global longitudinal strain (GLS) on echocardiography	From baseline (week 0) to visit 15 (week 52)	%-points	
Supportive secondary safety endpoint	Number of treatment emergent adverse events	From baseline (week 0) to visit 16 (week 64)	Count	
Supportive secondary safety endpoint	Time to occurrence of all-cause mortality	From baseline (week 0) to visit 16 (week 64)	Weeks	Cox proportional hazards model, summary measure as Hazard ratio.
Supportive secondary safety endpoint	Number of CV events comprising hospitalisation due to CV events or urgent heart failure visits	From baseline (week 0) to visit 16 (week 64)	Count	Anderson-Gill model with robust estimator, summary measure as Hazard ratio. All-cause death as censored events.
Exploratory endpoint	Change in misfolded transthyretin (misTTR)	From baseline (week 0) to visit 15 (week 52)	Percentage	
Exploratory endpoint	Change in EQ-5D-5L	From baseline (week 0) to visit 15 (week 52)	Score	
Other assessment	Change in 6-minute walk test (6MWT)	From baseline (week 0) to visit 8 (week 24)	Meters	
Other assessment	Change in NT-proBNP	From baseline (week 0) to visit 8 (week 24)	Percentage	Log transformed

Type	Title	Time frame	Unit	Details
Other assessment	Change in myocardial extracellular volume (ECV)	From baseline (week 0) to visit 8 (week 24)	%-points	
Other assessment	Change in Kansas City Cardiomyopathy Questionnaire (KCCQ) Clinical Summary Score (CSS)	From baseline (week 0) to visit 8 (week 24)	Score	
Other assessment	Change in neuropathy impairment score (NIS)	From baseline (week 0) to visit 8 (week 24)	Score	
Other assessment	Change in troponin I	From baseline (week 0) to visit 8 (week 24)	ng/mL	Log transformed
Other assessment	Change in global longitudinal strain (GLS) on echocardiography	From baseline (week 0) to visit 8 (week 24)	%-points	
Other assessment	Participants worsening 5 points or more in KCCQ clinical summary score (Yes/No)	From baseline (week 0) to visit 15 (week 52)	Count of Participants	
Other assessment	Participants worsening 10 points or more in KCCQ clinical summary score (Yes/No)	From baseline (week 0) to visit 15 (week 52)	Count of Participants	
Other assessment	Participants worsening 15 points or more in KCCQ clinical summary score (Yes/No)	From baseline (week 0) to visit 15 (week 52)	Count of Participants	
Other assessment	Participants improving 5 points or more in KCCQ clinical summary score (Yes/No)	From baseline (week 0) to visit 15 (week 52)	Count of Participants	
Other assessment	Participants improving 10 points or more in KCCQ clinical summary score (Yes/No)	From baseline (week 0) to visit 15 (week 52)	Count of Participants	
Other assessment	Participants improving 15 points or more in KCCQ clinical summary score (Yes/No)	From baseline (week 0) to visit 15 (week 52)	Count of Participants	
Other assessment	Participants worsening 5 points or more in KCCQ overall summary score (Yes/No)	From baseline (week 0) to visit 15 (week 52)	Count of Participants	
Other assessment	Participants worsening 10 points or more in KCCQ overall summary score (Yes/No)	From baseline (week 0) to visit 15 (week 52)	Count of Participants	

Type	Title	Time frame	Unit	Details
Other assessment	Participants worsening 15 points or more in KCCQ overall summary score (Yes/No)	From baseline (week 0) to visit 15 (week 52)	Count of Participants	
Other assessment	Participants improving 5 points or more in KCCQ overall summary score (Yes/No)	From baseline (week 0) to visit 15 (week 52)	Count of Participants	
Other assessment	Participants improving 10 points or more in KCCQ overall summary score (Yes/No)	From baseline (week 0) to visit 15 (week 52)	Count of Participants	
Other assessment	Participants improving 15 points or more in KCCQ overall summary score (Yes/No)	From baseline (week 0) to visit 15 (week 52)	Count of Participants	
Other assessment	Change in Kansas City Cardiomyopathy Questionnaire (KCCQ) Overall Summary Score (OSS)	From baseline (week 0) to visit 15 (week 52)	Score	

Appendix B : Neuropathy impairment score (NIS) and domains

The NIS consists of 74 questions which is only applicable for participants with hATTR CM.

These questions are mapped into 10 sum scores based on item groups such as "Cranial Nerves – RIGHT", "Cranial Nerves – LEFT ", "Muscle Weakness - RIGHT", "Muscle Weakness - LEFT", "Reflexes – RIGHT", "Reflexes – LEFT", "Sensation - I. Finger – RIGHT", "Sensation - I. Finger – LEFT", "Sensation - G. Toe – RIGHT", "Sensation - G. Toe – LEFT" which are linked to items listed from 1a to 10d in below Table 4.

Table 4 NIS questionnaire

Response scale is same across Item Group.

Item Group	Item No.	Item description	Response scale(*)
Cranial Nerves – RIGHT	1a	3rd Nerve	0: Normal<br>1: 25% weak<br>2: 50% weak<br>3: 75% weak<br>3.25: Move against gravity
	1b	6th Nerve	
	1c	Facial Weakness	

			3.5: Movement, gravity eliminated<br>3.75: Muscle flicker, no movement<br>4: Paralysis<br>Missing: Limited for other reasons or did not do the activity
	1d	Palate weakness	
	1e	Tongue weakness	
Cranial Nerves – LEFT	2a	3rd Nerve	0: Normal<br>1: 25% weak<br>2: 50% weak<br>3: 75% weak<br>3.25: Move against gravity<br>3.5: Movement, gravity eliminated<br>3.75: Muscle flicker, no movement<br>4: Paralysis<br>Missing: Limited for other reasons or did not do the activity
	2b	6th Nerve	
	2c	Facial Weakness	
	2d	Palate weakness	
	2e	Tongue weakness	
Muscle Weakness - RIGHT	3a	Respiratory	0: Normal<br>1: 25% weak<br>2: 50% weak<br>3: 75% weak<br>3.25: Move against gravity<br>3.5: Movement, gravity eliminated<br>3.75: Muscle flicker, no movement<br>4: Paralysis<br>Missing: Limited for other reasons or did not do the activity
	3b	Neck Flexion	
	3c	Shoulder abduction	
	3d	Elbow flexion	
	3e	Brachioradialis	
	3f	Elbow extension	
	3g	Wrist flexion	
	3h	Wrist extension	
	3i	Finger flexion	
	3j	Finger spread	

	3k	Thumb abduction	
	3l	Hip flexion	
	3m	Hip extension	
	3n	Knee flexion	
	3o	Knee extension	
	3p	Ankle dorsiflexors	
	3q	Ankle plantar flexors	
	3r	Toe extensors	
	3s	Toe flexors	
Muscle Weakness - LEFT	4a	Respiratory	0: Normal<br>1: 25% weak<br>2: 50% weak<br>3: 75% weak<br>3.25: Move against gravity
	4b	Neck Flexion	
	4c	Shoulder abduction	
	4d	Elbow flexion	

	4e	Brachioradialis	3.5: Movement, gravity eliminated<br><br>3.75: Muscle flicker, no movement<br><br>4: Paralysis<br>Missing: Limited for other reasons or did not do the activity
	4f	Elbow extension	
	4g	Wrist flexion	
	4h	Wrist extension	
	4i	Finger flexion	
	4j	Finger spread	
	4k	Thumb abduction	
	4l	Hip flexion	
	4m	Hip extension	
	4n	Knee flexion	
	4o	Knee extension	
	4p	Ankle dorsiflexors	
	4q	Ankle plantar flexors	
	4r	Toe extensors	
	4s	Toe flexors	
Reflexes – RIGHT	5a	Biceps brachii	0: Normal<br><br>1: Decreased<br><br>2: Absent<br><br>Missing: Limited for other reasons or did not do the activity
	5b	Triceps brachii	
	5c	Brachioradialis	
	5d	Quadriceps femoris	
	5e	Triceps surae	
Reflexes – LEFT	6a	Biceps brachii	0: Normal<br><br>1: Decreased<br><br>2: Absent
	6b	Triceps brachii	
	6c	Brachioradialis	

	6d	Quadriceps femoris	Missing: Limited for other reasons or did not do the activity
	6e	Triceps surae	
Sensation - I. Finger - RIGHT	7a	Touch pressure	0: Normal<br><br>1: Decreased<br><br>2: Absent<br><br>Missing: Limited for other reasons or did not do the activity
	7b	Pin-prick	
	7c	Vibration	
	7d	Joint position	
Sensation - I. Finger - LEFT	8a	Touch pressure	0: Normal<br><br>1: Decreased<br><br>2: Absent<br><br>Missing: Limited for other reasons or did not do the activity
	8b	Pin-prick	
	8c	Vibration	
	8d	Joint position	
Sensation - G. Toe - RIGHT	9a	Touch pressure	0: Normal<br><br>1: Decreased<br><br>2: Absent<br><br>Missing: Limited for other reasons or did not do the activity
	9b	Pin-prick	
	9c	Vibration	
	9d	Joint position	
Sensation - G. Toe - LEFT	10a	Touch pressure	0: Normal<br><br>1: Decreased<br><br>2: Absent<br><br>Missing: Limited for other reasons or did not do the activity
	10b	Pin-prick	
	10c	Vibration	
	10d	Joint position	

Definition of total scores by domain and Total NIS score :

"Total Reflexes Neuropathy Impairment Score" is defined as sum of scores from item groups of "Reflexes – RIGHT" (5a-5e), "Reflexes – LEFT" (6a-6e)

"Total Sensation Neuropathy Impairment Score" is defined as sum of scores from item groups of "Sensation - I. Finger – RIGHT"(7a-7d), "Sensation - I. Finger – LEFT"(8a-8d), "Sensation - G. Toe – RIGHT"(9a-9d), "Sensation - G. Toe – LEFT" (10a-10d)

"Total Weakness Neuropathy Impairment Score" is defined as sum of scores from item groups of "Cranial Nerves – RIGHT"(1a-1e), "Cranial Nerves – LEFT "(2a-2e), "Muscle Weakness - RIGHT"(3a-3s), "Muscle Weakness - LEFT"(4a-4s).

The "NIS score", our secondary endpoint of interest, is defined as the sum of Total Reflexes Neuropathy Impairment Score, Total Sensation Neuropathy Impairment Score, and Total Weakness Neuropathy Impairment Score.

If a participant misses any question within a specific item group, then the domain score for that item group will not be defined, and consequently, the "NIS score" will also not be defined.

7. References

1. Food and Drug Administration, CDER. Guidance for Industry. Diabetes Mellitus: Developing Drugs and Therapeutic Biologics for Treatment and Prevention, Draft Guidance. February 2008.
2. European Medicines Agency. ICH E9 (R1) addendum on estimands and sensitivity analysis in clinical trials to the guideline on statistical principles for clinical trials. Step 5 (EMA/CHMP/ICH/436221/2017). 17 Feb 2020.
3. Ghosh D, Lin DY. Nonparametric analysis of recurrent events and death. Biometrics. 2000;56(2):554-62.
4. Ghosh D, Lin D. Marginal regression models for recurrent and terminal events. Statistica Sinica. 2002;12:663-88.
